# Vasomechanic treatment is associated with increased efflux of human brain proteins

**DOI:** 10.1038/s41467-026-78894-y

**Published:** 2026-11-03

**Authors:** Johanna Tuunanen, Katariina Hautamäki, Tommi Väyrynen, Matti Järvelä, Vesa Korhonen, Niko Huotari, Mika Kaakinen, Janne Kananen, Heta Helakari, Lauri Raitamaa, Jari Jukkola, Sanna-Kaisa Herukka, Katariina Laurén, Ulla Salmi, Lauri Eklund, Juha Nikkinen, Maiken Nedergaard, Vesa Kiviniemi

**Affiliations:** 1 Oulu Functional Neuroimaging, Research unit of Health Sciences and Technology, Faculty of Medicine, University of Oulu, Oulu, Finland; 2 Radiology, Department of Diagnostics, Medical Research Center, Oulu University Hospital, Oulu, Finland; 3 Oulu Center for Cell-Matrix Research, Faculty of Biochemistry and Molecular Medicine, Biocenter Oulu, University of Oulu, Oulu, Finland; 4 Clinical Neurophysiology, Department of Diagnostics, Oulu University Hospital, Oulu, Finland; 5 Department of Experimental Medical Science, Lund University, Lund, Sweden; 6 Wallenberg Centre for Molecular Medicine, Lund University, Lund, Sweden; 7 Institute of Clinical Medicine/Neurology, University of Eastern Finland, Kuopio, Finland; 8 Department of Neurology, Kuopio University Hospital, Kuopio, Finland; 9 Department of Oncology and Radiotherapy, Medical Research Center, Oulu University Hospital, Oulu, Finland; 10 Research unit of Health Sciences and Technology, Faculty of Medicine, University of Oulu, Oulu, Finland; 11 Center for Translational Neuromedicine, University of Rochester, Rochester, NY USA; 12 Center for Translational Neuromedicine, University of Copenhagen Faculty of Health and Medical Sciences, Copenhagen, Denmark

**Keywords:** Neuroscience, Medical research, Physiology

## Abstract

An age-related neurovascular impairment in nitric oxide (NO)-mediated vasodilation and reciprocal Piezo1-dependent vascular vasoconstriction may precede the accumulation of soluble brain proteins such as amyloid-β (Aβ) and disruption of brain fluid homeostasis in Alzheimer’s disease (AD). Preclinical studies suggest that restoring these pathways can enhance neurofluid transport and improve cognitive outcomes. In this study (EudraCT 2021-000625-27), we investigated the effects of sublingual NO and Piezo1-targeted whole-body vibrations (WBV_p_) in healthy adults (*n* = 29) using multimodal neuroimaging and blood biomarker analysis. The combined intervention (NO+WBV_p_) was associated with correlating changes in brain fluid dynamics and increased blood levels of soluble brain-derived proteins (Aβ_40&42_, GFAP). These effects increased with age and the magnitude of NO-induced hypotension. The interventions were well tolerated under controlled experimental conditions. Overall, the findings are consistent with transient redistribution of brain-derived proteins toward circulation, suggesting a potential non-invasive approach to increase neurofluidic solute efflux in humans. Pre-specified primary endpoints, changes in circulating brain-derived protein concentrations, brain fluid imaging and brain pulsation measures, were met. Secondary outcomes included physiological analyses.

## Abstract (web summary)

Tuunanen et al. demonstrate a vasomechanic intervention to increase the efflux of soluble human brain proteins, including Aβ, into venous sinuses. The study shows how standard neuroimaging can monitor brain fluid dynamics during preventive efforts targeting proteinopathies such as Alzheimer’s disease.

## Introduction

The efficient removal of soluble metabolites and proteins from interstitial and cerebrospinal fluids (I/CSF) is essential for maintaining brain homeostasis. Impairment of brain clearance pathways has been linked to the earliest stages of neurodegenerative disease, preceding neuronal loss by years or decades^1^. Experimental and clinical studies demonstrate that impaired clearance of soluble amyloid-β (Aβ) and phosphorylated tau protein from the CSF leads to their accumulation within perivascular and interstitial compartments, promoting their aggregation and accelerating neuropathology^1–6^. For example, a murine model of Alzheimer’s disease (AD) exhibits progressive perivascular Aβ deposition accompanied by loss of endothelial low-density lipoprotein receptor-related protein 1 (LRP1), a key transporter mediating Aβ efflux to blood^5^. Elevated soluble Aβ concentrations within CSF further impede perivascular transport, creating a feed-forward cycle in which Aβ transport becomes increasingly impaired^5,6^. Therapeutic strategies that can safely enhance CSF solute clearance therefore represent a promising approach to delaying or preventing the onset of neurodegenerative disease.

Recent findings suggest that CSF efflux can be modulated through physiological mechanisms that are both therapeutically targetable and monitorable in humans. Acute systemic vasodilation caused by hypotensive drugs such as nicardipine and sodium nitroprusside (SNP) has emerged as a potent enhancer of CSF solute efflux in rodents^7^. Nitric oxide (NO) donors such as SNP increase movement of intracisternal tracers from the cranial cavity to the blood, predominantly via cervical lymph nodes, an effect that is accompanied by improved downstream lymphatic function^7,8^. Local NO delivery to neck lymphatic plexuses similarly enhances efflux of intracranial tracers, demonstrating that modulation of vascular tone can directly augment CSF transport along g/lymphatic pathways^8^.

Mechanical stimulation of Piezo1, a mechanosensitive cation channel expressed ubiquitously in all tissues including vascular and lymphatic endothelia, presents a complementary route for modulating neurofluid flow^9^. Recent work shows that even light mechanical stimulation activates Piezo1, increases endothelial NO synthase (eNOS) activity, and restores age-related deficits in cervical lymphatic efflux^9,10^. Piezo1 activation also increases meningeal lymphatic drainage^11,12^ and improves learning and memory in mice^13^.

Although pharmacological Piezo1 agonists are not yet clinically available, mechanical stimulation is an alternative noninvasive way to activate the receptor. Thus, low-frequency (≤40 Hz) whole-body vibration (WBV) improved cognition, brain activity^14,15^ and motor function^16,17^ in humans, and reduced neuroinflammation^18,19^, and Aβ burden^20^ in animal models. In humans, a 40 Hz vibration stimulus of the head modulated brain pulsations and CSF–brain coupling^21^, pointing to a translational pathway for mechanotransductive stimulation.

Because both NO-mediated vasodilation and Piezo1 activation have been shown to enhance CSF efflux, we hypothesized that simultaneously augmenting these functions globally in humans could promote the removal of potentially harmful brain-derived proteins. Specifically, we hypothesized that the intervention would be associated with (i) increasing blood concentrations of brain-derived proteins, (ii) detectable changes in brain T2-weighted dark-fluid (T2DF) magnetic resonance imaging (MRI) signal intensity, and (iii) alterations in brain hydrovascular pulsation dynamics. To address this, we investigated the effects of combined NO-mediated vasodilation and Piezo1-targeted vasoconstrictive mechanical stimulation (WBV_p_), as well as both interventions separately, and examined whether the combined intervention could influence CSF-to-blood solute transport in humans.

We exploited the joint capacities of advanced neurofluidic sensing technology linked to synchronous ultrafast brain pulsation magnetic resonance encephalography (MREG) imaging with previously established T2DF MRI to quantify the changes in neurofluidic drivers of protein-rich fluid shifts, as demonstrated in prior studies used to assess CSF protein burden^22,23^, and to characterize physiological changes in brain water dynamics and macromolecule distributions in healthy volunteers. We combined these measurements with clinical quantification of blood levels of brain-derived proteins that are relevant to neurodegeneration, including Aβ_40_, Aβ_42_, and glial fibrillary acidic protein (GFAP)^24^. Our results indicate that combined NO and WBV_p_ intervention is associated with changes in CSF dynamics and patterns in circulating brain-derived proteins that vary with age and molecular size, potentially reflecting modulation of brain fluid transport processes in humans.

## Results

In this human crossover trial (Supplementary Fig. 1), healthy volunteers (*n* = 29, of whom 15 were female, mean age: 44 ± 10 years; range 27–61 years) underwent two interventions in randomized order and on separate days: mechanotransductive whole-body vibration (WBV_p_, *n* = 27), and the combined protocol of NO-mediated vasodilation with WBV_p_ (NO+WBV_p_, *n* = 26). In addition, we conducted an exploratory NO-only session in a subset of participants (*n* = 12) on a separate day to characterize the acute effects of NO-mediated vasodilation in more detail. This design allowed us to isolate the distinct physiological and blood chemistry effects of each intervention. We also conducted complementary intrathecal tracer studies in mice to provide biological support for the observed phenomena.

### Vasodilation combined to Piezo1-targeted whole-body vibration

**Fig. 1:**
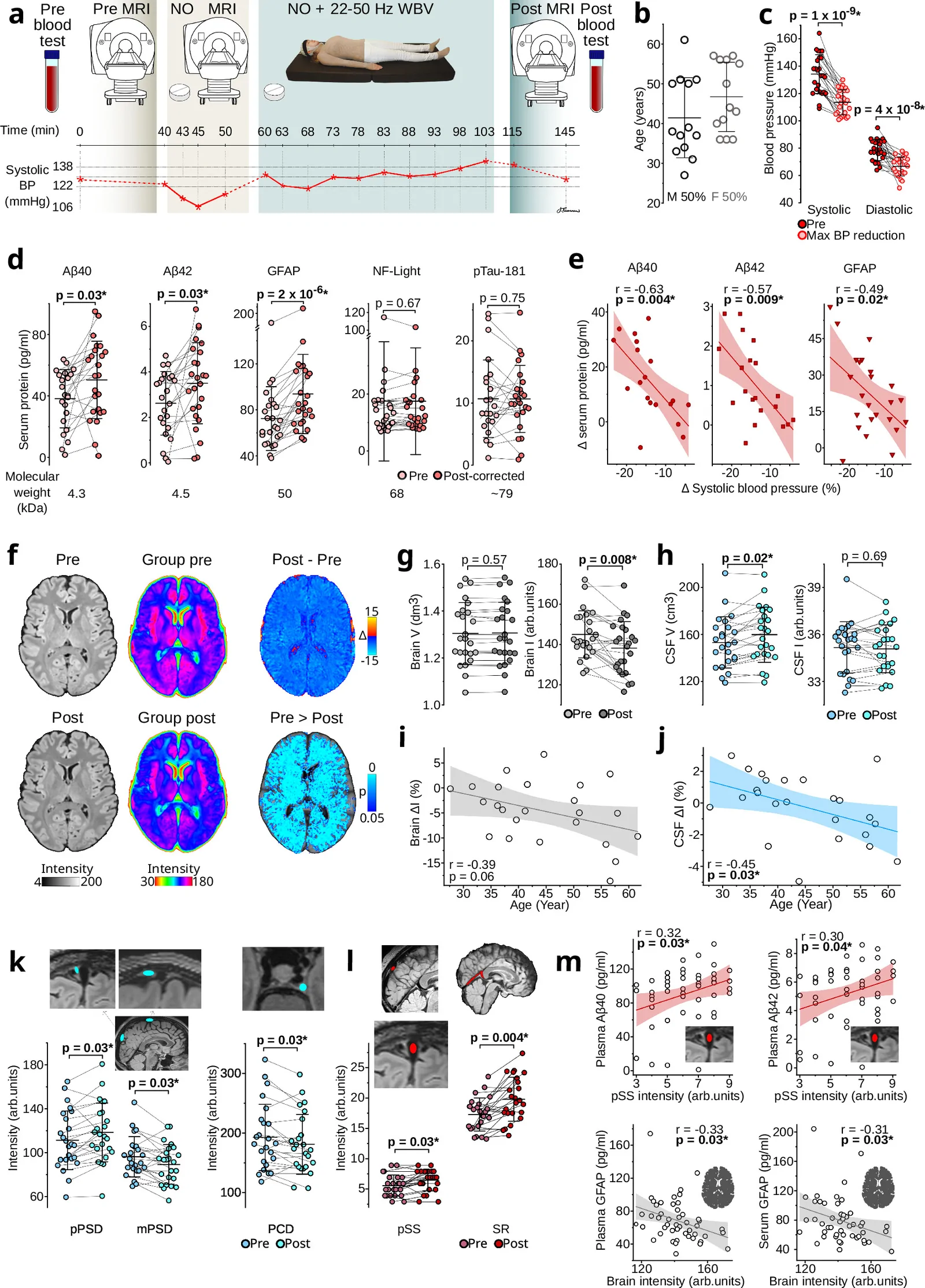
Changes in circulating brain-derived proteins associated with NO-mediated vasodilation combined with Piezo1-targeted whole-body vibration (NO + WBV_p_). **a** Study design of the NO + WBV_p_ condition of the randomized crossover trial, and a timeline chart with systolic blood pressure from a representative subject during the intervention. **b** Age (mean ± SD (standard deviation)) and sex distribution of participants, *n* = 26. **c** The maximal reduction of blood pressure (BP) due to the intervention, mean ± SD, paired two-tailed *t*-test, FDR (false discovery rate) correction, *n* = 25. **d** Venous blood analysis before and after the NO + WBV_p_ intervention, beta-amyloid 40 & 42 (Aβ40, *n* = 22; Aβ42, *n* = 23), glial fibrillary acidic protein (GFAP^#^, *n* = 25), neurofilament-light (NF-Light, *n* = 25), phosphorylated tau-181 (pTau-181, *n* = 23), mean ± SD, paired two-tailed *t*-test (^#^paired-sample Wilcoxon Signed-Rank test), FDR correction. **e** Low molecular weight (<68 kDa) proteins correlation with the individual extent of blood pressure reduction (Pearson’s correlation, linear regression line with 95% CI (confidence intervals), *n*_*Aβ40/Aβ42/GFAP*_ = 19/20/24). **f** T2 dark-fluid (T2DF) images from a representative subject before and after NO+WBV_p_ intervention (Pre, Post), group-averaged color-coded T2DF images (Group pre, Group post), group-averaged change map (Δ, Post – Pre), and statistical voxel-wise difference map (Pre > Post, paired two-tailed *t*-test, family-wise-error-rate correction, *P* < 0.05) due to intervention, *n* = 24. Identical intensity ranges and scaling conventions were applied in all pre and post maps. **g**, **h** Segmented brain and CSF volumes (V) and signal intensities (I) from T2DF images, mean ± SD, paired two-tailed *t*-test, FDR correction, *n* = 24. **i**, **j** Changes in brain and CSF T2DF signal intensities as functions of age (Pearson’s correlation, linear regression line with 95% CI, *n* = 24). **k**, **l** ROI analysis of T2DF images of CSF-filled spaces of the middle vertex parasagittal dura (mPSD), para-cavernous dura (PCD), parasagittal dura space (pPSD), posterior sinus sagittalis (SS^#^) and sinus rectus (SR), mean ± SD, paired two-tailed *t*-test (^#^paired-sample Wilcoxon Signed-Rank test), FDR correction, *n* = 24. **m** Measured blood biomarkers (Aβ40, Aβ42 and GFAP) correlation to T2DF signal intensities of brain and posterior sinus sagittalis (Pearson’s correlation, linear regression line with 95% CI, *n(Aβ40/Aβ42/GFAP)* = 46/47/48). Source data are provided as a Source Data file.

CSF tracer efflux has been shown to increase after NO exposure^7^ and after Piezo1 activation in mice^9,12^. We here tested the hypothesis that the effects of NO-mediated vasodilation therapy combined with WBV_p_ (Fig. 1a) could increase brain protein efflux and secondly explored the physiological effects of such a procedure in 26 healthy participants (mean age: 44 ± 9 years; range 27–61 years, 13 females; Fig. 1b) with our multimodal neuroimaging setup.

Before treatment, systolic/diastolic blood pressure measured from the left brachium was 134 ± 14 / 79 ± 8 mmHg. During combined NO+WBV_p_ intervention, the pressure fell to a nadir of 114 ± 9 / 67 ± 7 mmHg (paired *t*-test, *n* = 25, *t*(24) = 9.8, *P* < 0.0001 / *n* = 25, *t*(24) = 7.9, *P* < 0.0001, Fig. 1c and Supplementary Fig. 2), corresponding to 14.9 ± 6.3% (systolic) and 14.8 ± 9.0% (diastolic) reductions.

To assess changes in circulating markers potentially reflecting CNS-to-blood protein efflux, we collected venous blood samples before and after the intervention. Using the Quanterix Simoa HD-X platform to analyze blood concentrations, we found that the combined intervention significantly increased the low molecular weight (<68 kDa) brain-derived proteins in both serum; Aβ_40_ (pre 38.2 ± 19.0 pg/ml, post hemodilution-corrected 50.5 ± 25.2 pg/ml, paired *t*-test, *n* = 22, *t*(21) = −2.5, *P* = 0.03), Aβ_42_ (pre 2.6 ± 1.4 pg/ml, post-corrected 3.5 ± 1.8 pg/ml, paired *t*-test, *n* = 23, *t*(22) = −2.5, *P* = 0.03), and GFAP (pre 65.2 (56.7–83.5) pg/ml (median (interquartile range)), post-corrected 85.1 (69.7–113.5) pg/ml, paired-sample Wilcoxon signed-rank test, *n* = 25, *W* = 4, *P* < 0.0001) (Fig. 1d and Supplementary Fig. 3a) and plasma samples (Supplementary Fig. 3b). In contrast, the larger neurofilament-light (paired-sample Wilcoxon signed-rank test, *n* = 25, *W* = 139, *P* = 0.67) and phosphorylated tau-181 (paired *t*-test, *n* = 23, *t*(22) = −0.3, *P* = 0.75) proteins were unaltered in serum, suggesting that molecular weight could be a factor in CNS-to-blood protein movement. Moreover, the magnitude of NO-mediated blood pressure reduction during NO+WBV_p_ treatment correlated with the increased serum concentrations of Aβ_40_, Aβ_42_, and GFAP (Fig. 1e and Supplementary Fig. 4, Pearson’s correlation: Aβ_40_: *n* = 19, *r* = −0.63, *P* = 0.004; Aβ_42_: *n* = 20, *r* = −0.57, *P* = 0.009; GFAP: *n* = 24, *r* = −0.49, *P* = 0.02).

S100 calcium-binding protein B (S100B, pre: 0.05 (0.04–0.06) µg/l, post: 0.04 (0.03–0.08) µg/l, paired-sample Wilcoxon signed-rank test, *n* = 25, *W* = 138, *p* = 0.23) showed no significant changes. High-sensitivity C-reactive protein (hs-CRP, pre: 0.81 (0.47–1.23) mg/l, post: 0.79 (0.45–1.2) mg/l, paired-sample Wilcoxon signed-rank test, *n* = 25, *W* = 200, *P* = 0.03) decreased (by approximately 4%), consistent with hemodilution. Together, these findings suggest a good margin of clinical safety. Furthermore, the measured GFAP levels after intervention were markedly lower than reported after severe traumatic brain injury (median: 1924 pg/ml)^25^ or mild traumatic brain injury (mean: 1200 pg/ml)^26^.

To assess treatment effects on cerebral fluid dynamics and water content, we compared 3D T2DF anatomical high-resolution brain MR images acquired before and after the interventions; this approach is commonly used to evaluate brain edema, and more recently also for monitoring the presence of protein in parasagittal glymphatic CSF spaces^22,23^, c.f. Discussion and Methods for details. Here, we used T2DF imaging parameters on a Siemens 3 T scanner matching those in the latter study, which entailed protein concentration calibration of the T2DF signal with respect to free water protein levels^23^.

Following the combined treatment, whole-brain T2DF MRI signal intensity decreased by a mean of 4.6 ± 6.1% (paired *t*-test, *n* = 24, *t*(23) = 3.7, *P* = 0.008; Figs. 1f–g, 5c, g and Supplementary Fig. 6), indicating a reduction in the brain tissue water and/or macromolecule content in the brain, without any detectable change in brain tissue volume. In parallel, the volume of segmented extracerebral free dark CSF fluid increased by 6.5 ± 9.8 ml (paired *t*-test, *n* = 24, *t*(23) = −3.3, *P* = 0.02; Fig. 1h). While the mean CSF signal intensity surrounding the brain did not change significantly, the individual pre vs. post intervention changes did show a significant inverse correlation with age (Pearson’s correlation, *n* = 24, *r* = −0.45, *P* = 0.029; Fig. 1j); participants more than 45 years old exhibited reduced CSF signal intensity more suggestive of CSF water dilution, whereas younger individuals showed an increase in signal intensity more indicative of relative increase in protein efflux into CSF. Brain tissue signal intensity demonstrated a similar trend (Pearson’s correlation, *n* = 24, *r* = −0.39, *P* = 0.062, Fig. 1i), with older participants showing greater post-treatment darkening. Representative brain tissue and CSF segmentation masks are provided in Supplementary Fig. 25a.

Encouraged by the anticorrelated whole-brain and CSF changes, we next examined the dark-fluid MRI signal alterations within individually segmented tissue and fluid compartments to search for specific neurofluidic efflux sites. We performed individual region-of-interest (ROI) analyses to avoid spatial averaging artifacts introduced by MNI standard space registration, and to keep our focus on structures previously implicated in glymphatic solute transport (Fig. 1k, l and Supplementary Fig. 25b).

Within the parasagittal dura (PSD), the middle vertex PSD CSF-filled space (mPSD) showed a significant reduction in signal intensity (pre 97 ± 18 arb. units, post 90 ± 18 arb. units, paired *t*-test, *n* = 24, *t*(23) = 2.7, *P* = 0.03, Fig. 1k), whereas the posterior parts PSD CSF space (pPSD), exhibited an increase (pre 112 ± 27 arb. units, post 119 ± 27 arb. units, paired *t*-test, *n* = 24, *t*(23) = −2.8, *P* = 0.03, Fig. 1k). Signal intensity also decreased deeper down in the CSF-filled space in the para-cavernous dura (PCD, pre 193 ± 56 arb. units, post 182 ± 50 arb. units, paired *t*-test, *n* = 24, *t*(23) = 2.6, *p* = 0.03, Fig. 1k).

In contrast, the T2 dark-fluid MR signal increased within the venous sinuses (Fig. 1l), which may indicate increased protein levels^23^; the intraluminal T2DF signal increased from 5.5 (4.0–7.0) arb. units to 7.0 (5.5–8.0) arb. units (median (IQR)), paired-sample Wilcoxon signed-rank test, *n* = 24, *W* = 28, *P* = 0.03) within the posterior superior sagittal sinus and from 17.3 ± 2.7 arb. units to 19.9 ± 3.6 arb. units (paired *t*-test, *n* = 24, *t*(23) = −4.3, *P* = 0.004) in the sinus rectus. There was no significant change in the medial vertex segment of the sagittal sinus (pre 6.5 ± 2.0 arb. units, post 6.9 ± 2.6 arb. units, paired *t*-test, *n* = 24, *t*(23) = −0.7, *P* = 0.67, Supplementary Fig. 7c). Similarly, signal intensity was unaltered in most CSF areas such as the lateral ventricles, superior cerebellar cisterna adjacent to sinus rectus, fourth ventricles, or subarachnoid CSF space beneath the PSD (Supplementary Fig. 7a), nor were there any changes in midline cervical lymph nodes (pre 127.4 ± 20.4 arb. units, post 127.3 ± 19.9 arb. units, paired *t*-test, *n* = 24, *t*(23) = 0.03, *P* = 0.99, Supplementary Fig. 7b). In summary, the T2DF MRI results suggest changes in the ratio between macromolecule-bound brain water and age-dependent patterns that may reflect redistribution towards posterior and deep venous compartments, without evidence of brain volume loss.

We wanted to analyze the venous T2DF signal intensity change with respect to the quantified blood protein levels to assess whether the venous signals were indeed reflecting direct efflux into the veins. To this end, we performed a pooled analysis combining both baseline pre and post-intervention T2DF and blood Aβ and GFAP data. The results showed that plasma Aβ_40_ and Aβ_42_ concentrations correlated positively with posterior sagittal sinus T2DF signal intensity (Fig. 1m, Pearson’s correlation, Aβ_40_: *n* = 46, *r* = 0.32, *P* = 0.03; Aβ_42_: *n* = 47, *r* = 0.30, *P* = 0.04), with a similar trend observed for serum GFAP (Supplementary Fig. 8, Pearson’s correlation, *n* = 48, *r* = 0.26, *P* = 0.08). In addition, GFAP concentrations in both plasma and serum were inversely correlated with whole-brain T2DF signal intensity (Fig. 1m, Pearson’s correlation, plasma: *r* = −0.33, *P* = 0.03; serum: *r* = −0.30, *P* = 0.03), consistent with coupled changes in brain protein–water balance and circulating GFAP levels after the vasomechanic intervention. Notably, despite NO-induced hemodilution being known to lower plasma protein concentrations^27^, the combined intervention was associated with increased circulating protein levels.

Our supporting mouse experiments with 40 Hz vibration and the NO-donor SNP infusion demonstrated reduced accumulation of infused fluorescent FITC dextran (40 kDa) tracer in dorsal dura mater compared to SNP infusion alone, indicating that 40 Hz vibration reduced the concentration of macromolecules in the dura (Supplementary Fig. 28a).

### Vasomechanic NO+WBV_p_ effects on brain physiology

**Fig. 2:**
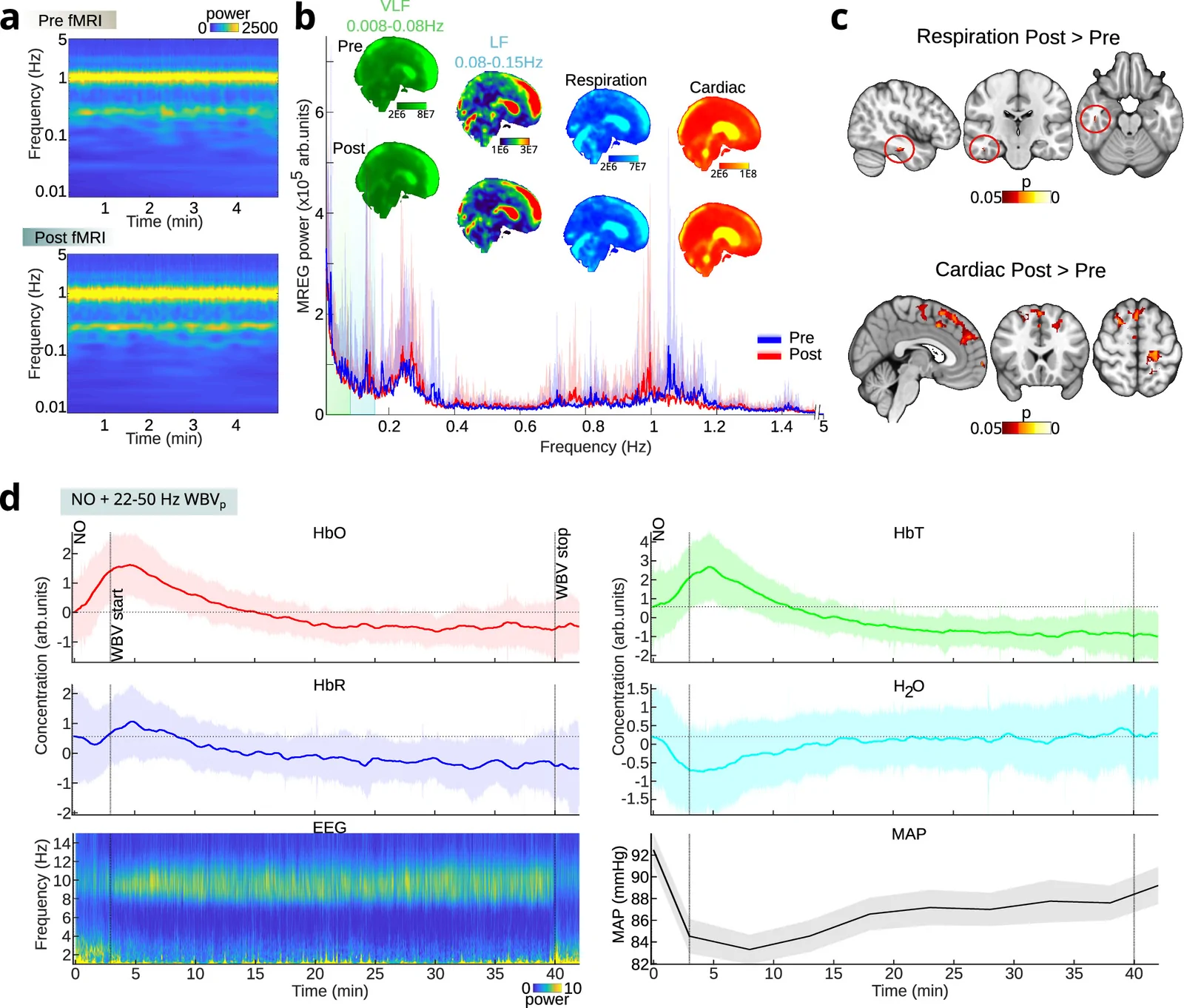
Combined vasomechanic NO + WBV_p_ intervention effects on brain physiology. **a**, **b** Group-averaged spectrogram and global power spectrum (mean + SD) with group mean power maps of very-low (VLF), low (LF), respiration, and cardiac frequencies from brain-wide ultrafast 10 Hz fMRI (MREG) scans (*n* = 23) taken before (Pre) and after (Post) NO + WBV_p_ intervention. Identical intensity ranges and scaling conventions were applied in all pre and post maps. **c** Statistical maps of the voxel-wise comparisons between Pre- and Post-MREG in respiratory (Post > Pre, *n* = 20) and cardiac (Post > Pre, *n* = 23) frequency ranges (paired two-tailed *t*-test, family-wise-error-rate correction, *P* < 0.05). No statistically significant effects were observed in the reverse contrast. **d** Electro- and hydrodynamic brain effects (*n* = 25) with near-infrared spectroscopy (fNIRS) measured at the forehead, and spectrogram of electroencephalography (dcEEG) recordings with simultaneous blood pressure measurements (*n* = 25) during the intervention. Group-averaged fNIRS signals (mean ± SD shown in the plots) of oxygenated blood (HbO), deoxygenated blood (HbR), total hemoglobin (HbT), and brain surface water content (H_2_O) corresponded to blood pressure changes (MAP = mean arterial pressure; mean ± SEM (standard error of the mean)). Source data are provided as a Source Data file.

To assess how the NO+WBV_p_ intervention alters intracranial physiology and the pulsatile driving of brain hydrodynamics, we acquired multimodal whole-brain magnetic resonance encephalography (MREG, 10 Hz) recordings before and after intervention (*n* = 25, Fig. 1a). Following the intervention, respiration-driven brain pulsation power increased in a focal region of the right temporal lobe (Fig. 2a–c and Supplementary Text 1). Cardiovascular pulsation power^28^ was also elevated after the intervention in frontal brain regions (Fig. 2a–c, Supplementary Fig. 9 and Supplementary Text 1). Fifteen minutes after the NO + WBV_p_ intervention, very-low frequency (VLF, 0.008–0.08 Hz) and low-frequency (LF, 0.08–0.15 Hz) vasomotor waves were unchanged compared with baseline (Fig. 2a, b).

Because WBV_p_ is not compatible with MR scanning, we quantified physiological responses during the vasomechanical stimulus outside the scanner using wearable sensing tools: functional near-infrared spectroscopy (fNIRS) over the forehead to measure hemo- (HbR/HbO) and H_2_O hydrodynamic and direct current electroencephalography (dcEEG) to assess electrophysiological responses sensitive to blood-brain barrier (BBB) hydrodynamic permeability during WBV_p_ experiments^29^.

**Fig. 3:**
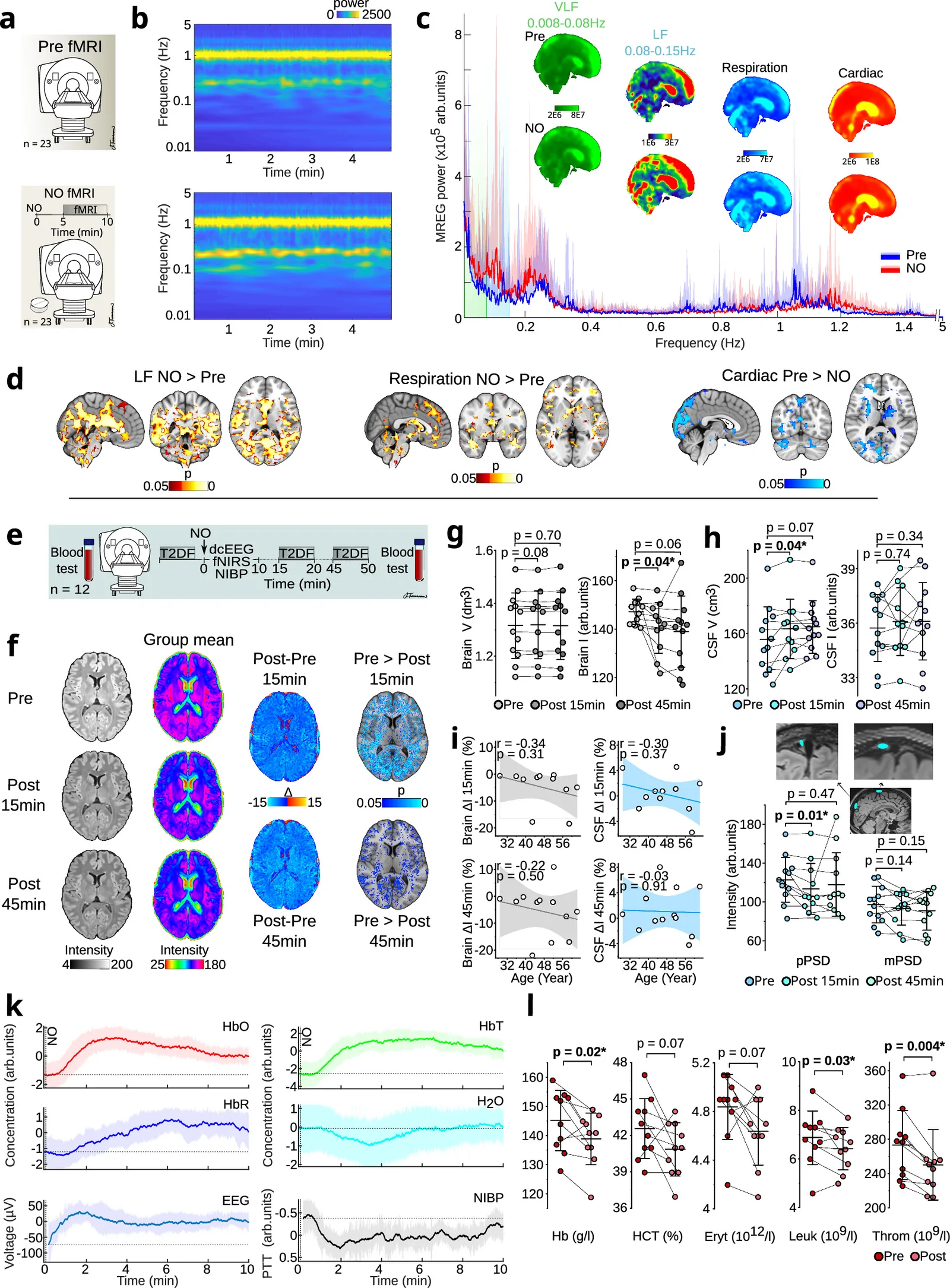
Isolated NO vasodilation (neuro)physiological effects. **a**, NO-only intervention. FMRI (MREG) measurements before (Pre) and during NO-mediated vasodilation. **b**, **c** Group-averaged spectrogram, power spectrum and power maps of very-low-frequency (VLF < 0.08 Hz), low-frequency (LF 0.08–0.15 Hz), respiratory and cardiac frequencies from MREG measurements during NO-mediated vasodilation (*n* = 23). Identical intensity ranges and scaling conventions were applied in all pre- and post-maps. **d** Voxel-wise statistical maps comparing baseline and NO vasodilation MREG (paired two-tailed *t*-test, family-wise-error-rate correction (FWER), *P* < 0.05, *n(LF/Respiration/Cardiac)* = 19/19/23). No statistically significant effects were observed in the reverse contrast. **e** NO-only intervention with serial T2-weighted dark-fluid (T2DF) MRI and blood counts for hemodilution assessment (*n* = 12). **f** T2DF images from a representative subject before and 15 min/45 min after nitroglycerin (Pre, Post 15 min/45 min), group-averaged T2DF images (Group Pre, Group Post 15/45), change map (Δ, Post 15/45 – Pre), and statistical maps (Pre > Post, paired two-tailed *t*-test, FWER *p* < 0.05, *n(Pre/15 min/45 min)* = 12/11/12). Identical intensity ranges and scaling conventions were applied in all pre, post, and subtraction maps. **g**, **h** Segmented brain and CSF volumes (V) and signal intensities (I) from T2DF images, mean ± standard deviation (SD), paired two-tailed *t*-test, *n(Pre/15 min/45 min)* = 12/11/12. **i** Changes in brain and CSF T2DF signal intensities as a function of age (Pearson’s correlation, linear regression line with 95% confidence intervals, *n(15 min/45 min)* = 11/12). **j** ROI analysis of T2DF images of the posterior part of CSF-filled parasagittal dura space (pPSD) and middle vertex parasagittal dura CSF space (mPSD), mean ± SD, paired two-tailed *t*-test, *n(Pre/15 min/45 min)* = 12/11/12. **k** Electro- and hemodynamic brain effects with near-infrared spectroscopy (fNIRS), electroencephalography (dcEEG) and continuous non-invasive blood pressure (NIBP, PTT = pulse transit time) recordings during NO vasodilation. Group-averaged signals (mean ± SD) of oxygenated blood (HbO), deoxygenated blood (HbR), total hemoglobin (HbT), brain surface water content (H_2_O), global dcEEG, and estimated blood pressure (NIBP; mean ± standard error of the mean). *N* = 12. **l** Blood tests before and after NO administration of hemoglobin (Hb), hematocrit (HCT), erythrocytes (Eryt^#^), leukocytes (Leuk), and thrombocytes (Throm^#^), mean ± SD, paired two-tailed *t*-test (^#^paired-sample Wilcoxon Signed-Rank test), *n* = 10. Source data are provided as a Source Data file.

**Fig. 4:**
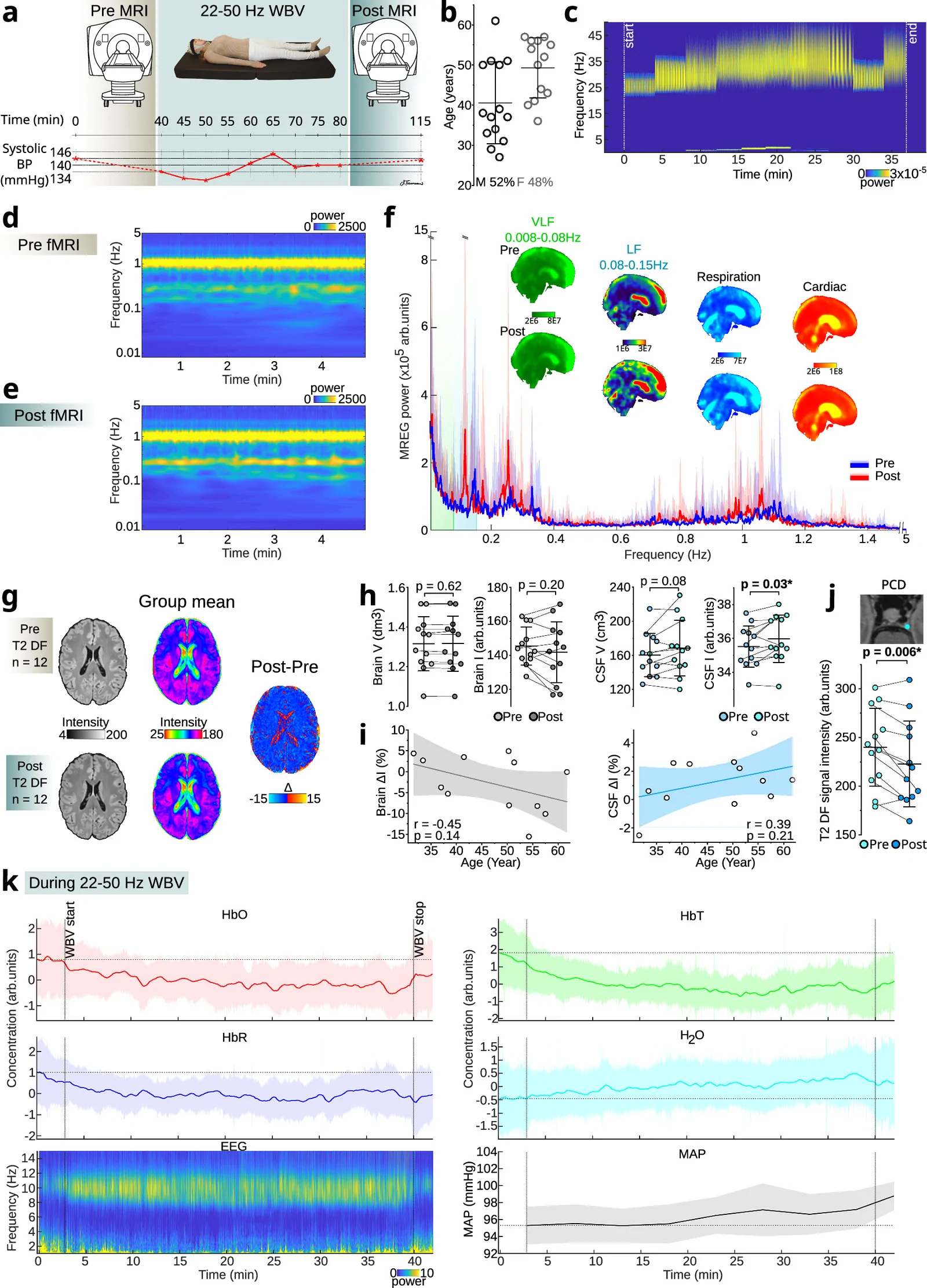
Piezo1-targeted whole-body vibration (WBV_p_) effects on brain physiology and water content. **a** Study design of the WBV_p_ part of the randomized crossover trial, and a representative example of systolic blood pressure during the intervention. **b** Age and sex distribution of participants (*n* = 27). **c** Spectrogram of the accelerometer from the Piezo1-targeted (22–50 Hz) vibration element positioned under the head. **d–f** Group-averaged spectrogram and global power spectrum with group mean power maps of very-low (VLF), low (LF), respiration, and cardiac frequencies from 10 Hz brain-wide fMRI (MREG) measurements (*n* = 25) before (Pre) and after (Post) WBV_p_ intervention. No statistically significant changes were observed in the voxel-wise comparisons. Identical intensity ranges and scaling conventions were applied in all pre and post maps. **g** T2 dark-fluid (T2DF) images from a representative subject before (Pre) and after (Post) intervention, group-averaged color-coded T2DF images (Group pre, Group post), and group-averaged change map (Δ, Post – Pre). Identical intensity ranges and scaling conventions were applied in all pre and post maps. There were no statistically significant voxel intensity changes between the pre- and post-intervention scans. *N* = 12. **h** Segmented brain and CSF volumes (V) and signal intensities (I) from T2DF images, mean ± standard deviation (SD), paired two-tailed *t*-test, *n* = 12. **i** Change in brain and CSF T2DF signal intensities as a function of age (Pearson’s correlation). Graphs indicate linear regression line with 95% confidence intervals, *n* = 12. **j** ROI analysis of T2DF signal intensity in the para-cavernous dura CSF-filled space (PCD), mean ± SD, paired two-tailed *t*-test, *n* = 12. **k** Electro- and hydrodynamic brain effects measured with near-infrared spectroscopy (*n* = 22) at the forehead, and spectrogram of electroencephalography (EEG, *n* = 25) with simultaneous blood pressure recordings (*n* = 27) during the intervention. Group-averaged signals (shown as mean ± SD in the plots) of oxygenated blood (HbO), deoxygenated blood (HbR), total hemoglobin (HbT), brain surface water content (H_2_O), and blood pressure (MAP = mean arterial pressure; mean ± standard error of the mean). Source data are provided as a Source Data file.

At the onset of intervention, there was a rapid rise in oxygenated hemoglobin (HbO) along with a slower and smaller increase in deoxygenated hemoglobin (HbR), consistent with NO-mediated vasodilation (Figs. 2d and 3k). These hemodynamic effects coincided with reductions in mean arterial pressure (MAP) and brain water content (H_2_O). MAP did not recover to baseline during the intervention, whereas H_2_O gradually returned to baseline after 37 min (Fig. 2d). The combined hemo-hydrodynamic profile matched simple NO-mediated vasodilation during the first ~10 min (Fig. 3k). Thereafter, concurrent WBV_p_ effects (Fig. 2d), also seen in the WBV_p_-alone experiment (Fig. 4k), began to counteract the NO-driven changes, reversing the hemodynamic and hydrodynamic trends (Figs. 2d, 3k and 4k). Notably, the combined treatment evoked a temporary H_2_O response distinct from that with WBV_p_ alone; while WBV_p_-alone caused a continuous rise in H_2_O accompanied by increased MAP (Fig. 4k), the combined NO+WBV_p_ intervention produced only a transient decrease in H_2_O, with subsequent recovery. Overall, the combined intervention seemingly reflects a temporal summation of NO and vibration effects, with a characteristic difference in brain water dynamics (Figs. 2–4).

During WBV_p_, dcEEG showed increased alpha power (8–12 Hz), likely reflecting eye closure and relaxed drowsiness, as participants kept their eyes closed for ~45% of the period with vibration. Low-frequency activity (<4 Hz) decreased during vibration and re-emerged immediately afterward.

### NO vasodilation (neuro)physiological effects

To separately quantify the intracranial physiological responses to acute NO vasodilation alone, we acquired 10 Hz fMRI (MREG) during an initial NO phase of the combined NO+WBV_p_ intervention visit (Fig. 1a) in twenty-three subjects (Fig. 3a). NO produced arterial vasodilation accompanied by systemic blood pressure reduction in both MAP and continuous non-invasive blood pressure (NIBP) measurements (Figs. 2d, 3k and Supplementary Fig. 2). This vasodilation increased power and reduced the frequency of the brain vasomotor wave ~0.1 Hz LF peak (Fig. 3b–d and Supplementary Fig. 12). VLF power <0.08 Hz remained unchanged, despite the expectation that ubiquitous NO vasodilation should suppress vasomotion. NO also increased the respiratory (Fig. 3b–d and Supplementary Fig. 13) and decreased cardiovascular (Fig. 3b–d and Supplementary Fig. 14) brain pulsation power.

To assess acute NO-mediated vasodilation changes in greater detail, we performed a dedicated exploratory sub-study of T2DF MRI, dcEEG, fNIRS and NIBP recordings in twelve participants (mean age: 46 ± 10 years; range 27–61 years, 6 females) on a separate study day (Fig. 3e).

**Fig. 5:**
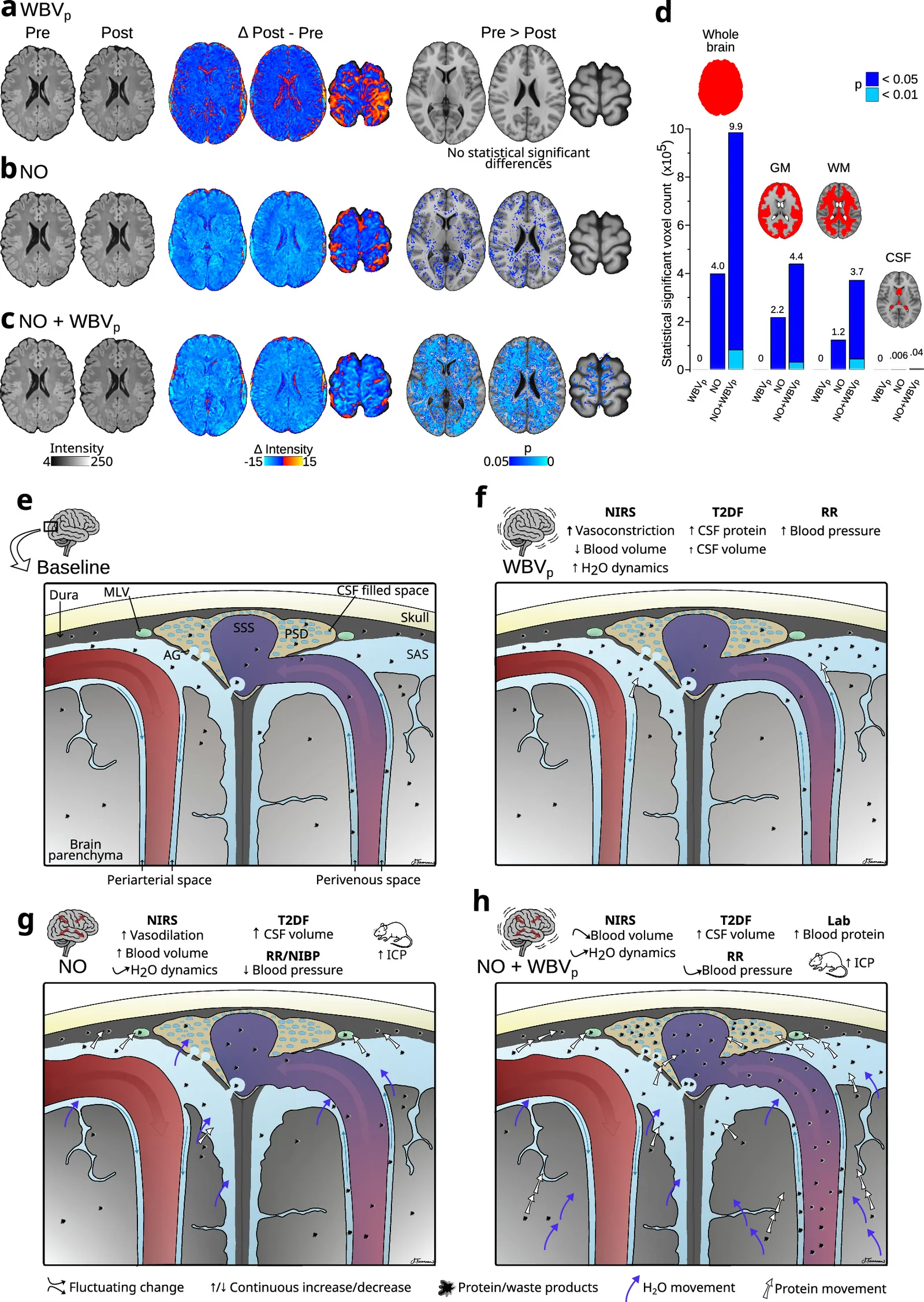
Spatial extent of T2DF MRI signal changes and schematic representation of mechanisms associated with fluid, vascular, and protein redistribution processes across investigated interventions. T2 dark-fluid (T2DF) images from a representative subject before (Pre) and after (Post) intervention, group-averaged change maps (Δ, Post – Pre), and statistical voxel-wise difference maps (Pre > Post, paired two-tailed *t*-test, family-wise-error-rate correction *P* < 0.05, *n* = 12) of **a** WBV_p_, **b** NO and **c** combined NO + WBV_p_ intervention. Identical intensity ranges and scaling conventions were applied in all pre, post-, subtraction and difference maps. **d** Quantified spatial extent of the significantly altered T2DF intensity changes in whole-brain and segmented gray matter (GM), white matter (WM) and cerebrospinal fluid (CSF) per intervention. Illustrative diagrams of (**e**), baseline, and the main effects with postulated mechanisms in (**f**). Piezo1-targeted whole-body vibration (WBV_p,_ Fig. 4) induces mainly slowly emerging vasoconstriction, elevating blood pressure (BP) and increasing CSF volume, with a brighter T2DF signal indicating higher protein content. **g** NO vasodilation (NO, Fig. 3) drops BP, increases 0.1 Hz vasomotor waves in arteries and respiratory pulsations in veins, similar to sleep, which also increases solute transport^29,44,46^. NO increases intracranial pressure (ICP)^7^, resulting in removal of brain water along with proteins into CSF and to posterior parasagittal dura (PSD) and venous sinuses, relatively more from the GM. Given the quantified NO-induced hemodilution on blood counts (Fig. 3l)^27^, the stable venous T2DF signal (Supplementary Fig. 17) suggests a relative elevation of blood proteins, as hemodilution normally reduces protein concentrations. **h** The combined (NO+WBV_p_, Figs. 1 and 2) intervention induces, in addition to the NO and WBV_p_ effects on pulsations driving brain solute dynamics, a longer-lasting BP drop and altered water hydrodynamics followed by slower BP increase. Brain neurofluidic redistributions occur over a markedly wider area, particularly in deeper WM, resulting in brain-wide protein/water removal into posterior PSD and venous sinuses. Notably, the linear correlations of blood Aβ_40&42_ concentrations (Fig. 1m) with the increased T2DF signal in posterior venous sinuses despite NO-induced hemodilution indicate simultaneous laboratory and imaging evidence suggestive of an increase in posterior venous protein permeability as compared to NO intervention alone. NIRS = near-infrared spectroscopy, SAS = subarachnoid space, SSS = superior sagittal sinus, ML = meningeal lymphatic vessel, AG = arachnoid granulation.

The electro/hydrodynamic measures indicated that the NO vasodilation alone produced effects resembling the early part of the combined vasomechanic intervention. Whole-brain T2DF MRI signal intensity decreased by 4.8 ± 6.8% at 15 min post-administration (paired *t*-test, *n* = 11, *t*(10) = 2.3, *P* = 0.04, Fig. 3f, g and Supplementary Fig. 10). Over the entire 45-min measurement period, mean T2DF signal intensity continued to decline by 5.5 ± 9.1%, showing a trend toward significance (paired *t*-test, *n* = 12, *t*(11) = 2.1, *P* = 0.06, Figs. 3f, g, 5b and Supplementary Fig. 11). Brain tissue volume remained unchanged (Fig. 3g), whereas CSF volume again had increased by 7.0 ± 9.7 ml at 15 min (paired *t*-test, *n* = 11, *t*(10) = −2.4, *P* = 0.04) and 9.2 ± 15.6 ml at 45 min (paired *t*-test, *n* = 12, *t*(11) = −2.0, *P* = 0.07, Fig. 3h).

T2DF signal intensity in CSF did not change significantly (Fig. 3h), and neither the brain nor CSF signal intensity correlated with age after NO-mediated vasodilation (Fig. 3i). The posterior PSD CSF-filled space (Fig. 3j) displayed an acute decrease in signal intensity at 15 min (paired *t*-test, *n* = 11, *t*(10) = 3.0, *P* = 0.01), consistent with a possible vasodilation-induced reduction in the local macromolecule/water ratio. Further ROI analyses showed no significant changes in sagittal sinus, lymph nodes, or the CSF compartments (Supplementary Figs. 15–17).

NO induced a transient hypotension (Fig. 3k) that was accompanied by an immediate 117 ± 98 µV DC shift in the dcEEG, followed by a delayed rise in arterial HbO with a temporal profile closely resembling the NIBP and dcEEG curve outlines (Fig. 3k). A slower, transient reduction in brain H_2_O content emerged, which was followed by a gradual venous HbR increase. At 15 and 45 min after NO-alone administration, T2DF MRI recapitulated the global brain signal reductions observed with the combined NO+WBV_p_ intervention (Fig. 3f, g).

Because NO administration induces a hemodilution that usually decreases blood protein concentrations^27,30^, we quantified dilution effects after the 0.5 mg sublingual dose (Fig. 1d, e). Basic blood count before and after NO showed decreases in hemoglobin (4.2 ± 4.7 \%), hematocrit (3.8 ± 5.9%), erythrocytes (4.0 ± 5.6%), leukocytes (5.8 ± 7.8%), and thrombocytes (8.3 ± 5.3%), corresponding to an estimated 5.2% blood-water dilution (Fig. 3l). The correction factor was applied uniformly to all analytes and was used only in the analyses presented in Fig. 1d, e. We also confirmed that the NO-mediated vasodilation by SNP in anesthetized mice promoted a significant 40 kDa FITC dextran removal from CSF to blood (Supplementary Fig. 28b).

### Piezo1-targeted whole-body vibration

Mechanical low-frequency Piezo1 activation enhances g/lymphatic solute flow^31^. To isolate the physiological effects of Piezo1-targeted (modulated in the low-frequency range between 22–50 Hz, c.f. Fig. 4c) WBV_p_, twenty-seven participants (mean age: 45 ± 10 years; range 27–61 years, 13 females; Fig. 4b) underwent measurements of brain pulsation power, water content, and hemodynamic and electrophysiological responses (Fig. 4a).

During WBV_p_, MAP showed an upward trend accompanied by an increase in brain H_2_O content and a decrease in total blood volume (HbT, arterial HbO and venous HbR) measured by fNIRS (Fig. 4k). Participants kept their eyes closed for ~48% of the 37 min vibration time and the dcEEG alpha power during the vibration-alone attained approximately 12% higher power than in the combined NO+WBV_p_ intervention (Fig. 2d). There was also a reduction in low-frequency power (<4 Hz), which was less pronounced than in the combined NO + WBV_p_.

T2DF MRI, which was available for twelve participants, showed a trend toward increased CSF volume (paired *t*-test, *n* = 12, *t*(11) = −1.9, *P* = 0.08, Figs. 4g, h and 5a), whereas brain tissue volume and intensity remained stable. Whole-brain CSF intensity increased significantly (paired *t*-test, *n* = 12, *t*(11) = −2.4, *P* = 0.03, Fig. 4h), which may reflect an increase in small-molecule content within the cortical CSF spaces; ROI analysis, as in the earlier intervention (Fig. 1k, l), revealed no significant changes in the lateral ventricles, superior cisterna, fourth ventricles, or parasagittal CSF space (Supplementary Fig. 9). Likewise, ROI analysis detected no significant changes in sinus, lymph nodes, or PSD (Supplementary Figs. 20 and 21).

Change in brain tissue T2DF signal intensity following WBV_p_ showed a non-significant correlation with age (Pearson’s correlation, *n* = 12, *r* = −0.45, *P* = 0.14, Fig. 4i), with older participants tending to exhibit greater signal reduction (i.e., darker brain tissue). Likewise, CSF intensity tended to increase more in older individuals (Fig. 4i). There was a significant intensity decrease in the para-cavernous dura CSF-filled space (PCD, pre 239.8 ± 38.2 arb. units, post 222.8 ± 42.1 arb. units, paired *t*-test, *n* = 12, *t*(11) = 3.4, *P* = 0.006, Fig. 4j). Although MAP rose during active WBV_p_ (Fig. 4k), follow-up blood pressure measured 30 min later in 15 subjects showed a modest decline relative to baseline (systolic 134 ± 14 → 131 ± 12 mmHg, *P* = 0.2; diastolic 82 ± 7 → 77 ± 7 mmHg, *P* = 0.06).

Across VLF, LF, respiratory or cardiovascular brain pulsation powers, there were no statistically significant voxelwise differences between pre- and post-WBV_p_ scans (Fig. 4d–f and Supplementary Fig. 18).

### The combined vasomechanic intervention resulted in more spatially extensive T2DF MRI signal changes than individual treatments

To enable a direct comparison of spatial effects across interventions, T2DF MRI data were analyzed in a matched subset of participants (*n* = 12) with available scans for NO and the combined NO+WBV_p_ condition, while WBV_p_-only data were derived from a partially overlapping cohort (Fig. 5). The voxel-wise magnitude of T2DF MRI signal intensity change was comparable between the NO and combined vasomechanic interventions (Fig. 5b, c). However, the spatial extent of signal changes was substantially larger in the combined condition, corresponding to approximately 2–6 times the total affected volume observed across the individual NO and WBV_p_ interventions (Fig. 5d). In addition to the empirical data, a schematic illustration is included to summarize the main findings and their proposed physiological interpretation (Fig. 5e–h).

### Sham intervention effects on T2 dark-fluid image

To control for potential effects of repeated scanning and prolonged supine positioning, a sham intervention cohort (*n* = 13; mean age 39.7 ± 10.9 years; range 26–67 years; 6 females) was examined using identical T2DF MRI acquisition parameters. Participants were scanned before and after a 45-min resting period without active intervention, corresponding to the WBV_p_ treatment duration.

No statistically significant changes were observed in T2DF signal intensity between pre- and post-sham scans (Supplementary Fig. 22). However, inspection of the difference images indicated a tendency toward increased signal intensity following the resting period, in contrast to the signal reductions observed after the active interventions. These findings suggest that prolonged supine positioning alone may be associated with a slight increase in brain water-related signal, whereas the vasomechanic interventions were associated with opposite signal changes.

### Treatment side effects

The interventions were feasible and acceptably tolerated under controlled experimental conditions, although transient adverse effects were observed: Mild adverse effects were reported following NO administration, with four participants reporting headache and three reporting nausea. In one case, the study was discontinued due to vomiting following NO administration. All reported symptoms resolved during the observation period within 30 min after active intervention. No participants reported discomfort associated with the whole-body vibration procedure. There were no serious adverse events.

## Discussion

In this study, we investigated the hypothesis that human brain solute and protein efflux into the systemic circulation could be enhanced through combined vasomechanic interventions that dynamically modulate cerebral cerebrofluidic dynamics in healthy volunteers. We investigated the individual effects of vasodilatory nitric oxide (NO) and mechanotransductive whole-body vibration targeting Piezo1 receptors (WBV_p_) in separate experiments, and in their vasomechanic (NO + WBV_p_) combination whether they could modulate human brain fluid dynamics and influence circulating levels of brain-derived proteins. The principal findings show that the combined vasomechanic intervention was associated with (i) increased venous blood concentrations of low-molecular-weight proteins (Aβ40, Aβ42, and GFAP) proportional to NO-mediated blood pressure drop; (ii) changes in dark-fluid T2DF MRI signals reflected hydrodynamic changes across the brain, dural CSF-filled spaces, and importantly within the venous sinuses in proportion to venous blood Ab protein increase and age, and, (iii) physiological responses reflecting altered neurovascular and hydrodynamic pulsations driving cerebral solutions in a way similar to sleep. Together, these observations are consistent with a transient redistribution of cerebral fluids and brain-derived macromolecules into blood circulation.

Mechanistically, the combined vasomechanic intervention was associated with widespread and age-dependent changes in neurofluid dynamics, reflected by coordinated alterations in global brain tissue, CSF, and posterior PSD and venous sinus signals on dark-fluid MRI, which coincided with hemo- and hydrodynamic signatures detected by simultaneous fNIRS monitoring. NO alone induced, as expected, MAP reduction, cerebral hyperemia, blood-water dilution, and a slowing and amplification of 0.1 Hz vasomotor and respiratory pulsations, accompanied by net CSF expansion and decreased dark-fluid MRI signal intensity, consistent with reduced water/macromolecule ratios.

In contrast, whole-body low-frequency modulated vibration targeting Piezo1 receptors (WBV_p_) gradually increased systemic MAP, elevated brain CSF-water fNIRS signals with higher intensity T2DF, and reduced brain HbO_2_ levels and para-cavernous dural signals. Taken together, the findings indicate that combining pharmacological vasodilation with mechanical vasoconstrictive stimulation is associated with complementary vascular and CSF transport processes and may facilitate human brain solute efflux.

Endothelial deficiency of NO promotes AD pathology^32^, and conversely, a large Taiwanese cohort study recently reported a reduced risk of dementia in individuals treated with NO-based vasodilators^33^, consistent with other clinical evidence that strict blood pressure control lowers the risk of transition from mild cognitive impairment to dementia^34,35^. Mechanistically, NO-based vasodilation produces a drop in systemic blood pressure, which increases ICP and enhances brain tracer efflux^7^. This pressure-dependent stimulation of solute clearance provides a plausible physiological mechanism for the potential preventative effect of NO therapy on neurodegeneration.

Soluble Aβ_40_ is thought to be the most neurotoxic amyloid species in early AD, and is consequently a favored target for interventive treatment^36^. At the same time, increased CSF concentrations of Aβ_40&42_ directly impede glymphatic transport, producing a feed-forward deficit in solute clearance^6^. Aging further exacerbates this via reduced endothelial LRP1-mediated Aβ transport and loss of AQP4 polarization at astrocytic endfeet, together leading to diminished Aβ removal to blood^5,37^. In this context, the present findings suggest that soluble Aβ species may be mobilized toward the blood circulation under combined vasomechanic intervention (Fig. 1). There is some early evidence in support of such an efflux of interstitial solutes into the venous blood flow^38^. The present vasomechanic intervention results suggest a feasible, non-invasive approach to counteract the age-related decline in brain protein efflux, which might conceivably prove to be protective against AD and other age-dependent neurodegenerative proteinopathies. However, given the apparent molecular size selectivity (<68 kDa, Fig. 1d), this effect may preferentially concern soluble proteins prior to their aggregation into larger complexes.

Previously, the physiological state most strongly linked to efficient glymphatic transport was sleep; CSF tracer efflux and removal of Aβ increase during natural sleep^39–41^, while sleep deficit in humans reduces Aβ clearance^41–43^. During natural sleep, orexin-dependent reductions in central noradrenergic tone and blood pressure^44,45^ induce robust vasomotor and respiratory pulsations to promote solute efflux^28,29,44,46,47^. NO intervention appears to recapitulate key elements of this sleep-associated physiology; it lowers blood pressure (Fig. 3k and Supplementary Fig. 2), and importantly also amplifies ~0.1 Hz vasomotor brain waves and respiratory pulsations (Fig. 3b–d)^46,48^, which may contribute to the observed enhancement of protein efflux into blood.

Piezo1 channels in the cerebrovascular endothelium are activated by mechanical forces and counteract excessive arterial vasodilation by inducing vasoconstriction^31^. Notably, continuous pharmacological activation of endothelial Piezo1 has been linked to learning and memory impairments in rodents, whereas increased astrocytic Piezo1 activity promotes cognitive function^13,31^, highlighting their target-specific relevance to neurocognition. In the present study, modulated WBV_p_ evoked a gradual reduction in cerebral blood flow together with an increase in MAP (Fig. 4k), consistent with a reactive vasoconstrictive response that is in keeping with the occurrence of Piezo1 activation. A precise low-frequency modulation of the vibration pattern is essential for this approach, as Piezo1 channels rapidly desensitize during tonic, prolonged stimulation^49^. Pharmacological agonists of Piezo1, such as Yoda1, remain ineffective for brain applications because they fail to cross the BBB^50^. Piezo1 agonist drugs also cause tonic activation of endothelial Piezo1, which induces a long-lasting vasoconstriction that can attenuate the important vasodilation responses coupled to neuronal activity, ultimately leading to cognitive decline in mice^31^. Thus, we used herein a slowly modulated Piezo1 stimulation to induce non-continuous, oscillatory vasoconstrictions, similar to the vasomotor waves recently shown to be in direct proportion with the amount of brain solute efflux^44^.

Mechanotransductive vibration therapies have repeatedly been shown to improve learning, cognition, and motor function across multiple species^14–19,51^. Low-frequency vasomotor brain pulsations^44^ drive hydrodynamics over the BBB, especially in sleep^29^, as well as low pressure mechanical stimulation of the cervical lymphatic plexus in mice increases CNS solute efflux^9^. In contrast to pharmacological approaches, the WBV_p_ used in this study enabled a controlled, low pressure whole-body Piezo1 activation in a low-frequency oscillating pattern, which may further augment solute efflux (Fig. 4h). Importantly, as the mechanical activation of Piezo1 in blood and lymphatic vessels further stimulates eNOS-driven NO production^9,10,12,52^, their combined re-awakening by external vasomechanic intervention seems to offer a mechanistic basis for the spatially greater enhancement of brain hydrodynamics and solute efflux seen with the present vasomechanic intervention (Figs. 1 and 5).

Ageing introduces several convergent deficits that degrade brain fluid transport, including reduced eNOS activity, diminished AQP4 expression^12,53^ and endothelial Piezo1 function^9^, which together can impair brain neurofluidic functions such as the (g)lymphatic efflux^54^. These changes may help explain the age dependency observed in the present hydrodynamic responses (Fig. 1i, j and Supplementary Fig. 5), although given the modest sample size, especially in the separate NO and WBV_p_ studies, these age-related observations should be interpreted cautiously. Whole-body vibrations may also boost NO production via eNOS activation, thereby promoting multiple other neuroprotective pathways^55^. Within the brain, Piezo1 activation has also been shown to mitigate Aβ-induced defects in microglial motility and phagocytosis and improve AD-related cognitive dysfunction^56^.

Based on the observed CSF and deep para-cavernous dural T2DF MRI changes (Figs. 1k and 4j), we propose that WBV_p_ can affect not only cortical glymphatic pathways, but also the deeper brain efflux routes from the white matter that may be associated with dural and basal cavernous outflow systems^57,58^. In rodents, CSF clearance is associated with the dura mater, which contains lymphatic vessels and blood-collecting venous sinuses. Substances present in CSF may be absorbed into the dura mater, where they are drained by transit into blood and lymph. We have recently shown that accumulation of a 40 kDa fluorescent tracer from CSF into the dorsal dura mater is significantly increased upon lowering blood pressure in mice^7^. Interestingly, combining the WBV with SNP-induced blood pressure lowering significantly reduced the amount of the same tracer in dura, while increasing its transit to blood (Supplementary Fig. 28), thus suggesting complementary action promoting dural clearance in posterior regions similar to the current results in human posterior venous sinuses (Fig. 1l). A recent study indicated that solute efflux from the human brain can also occur partly through perineural routes, as seen in rodents^59^, whereas paraspinal CSF pathways may represent major contributors to net efflux^60^.

We used the T2 dark-fluid (a.k.a. FLAIR-based) MRI signal intensity analysis applied earlier by other groups for investigating brain solute transport within glymphatic PSD interfaces^22,23,57^. T2DF is a very important sequence in clinical neuroimaging, where tissue-bound water has high signal intensity due to the proximity of water ^1^H to macromolecules like proteins. Less constricted water molecules distal from macromolecules in neurofluidic compartments such as blood and CSF are labeled dark in T2DF MRI by a precisely timed inversion pulse, rendering dark the freely precessing water ^1^H signal. In clinical imaging, an acutely increased T2DF signal is often related to brain tissue edema, and darkening of the T2DF signal typically reflects a reversal of such pathology after a successful therapeutic intervention. The current T2DF MRI results need to be interpreted differentially depending on the particular brain compartment: whereas darkening in tissue may mainly reflect a reduced amount of bound water ^1^H signal, darkening in neurofluidic compartments with normally low signal compartments is more indicative of increased water concentration in the relative absence of macromolecules such as proteins.

As demonstrated in previous protein phantom model verification study with a similar 3 T scanner using identical TR, TI and TE sequence parameters, T2DF signal intensity in free-water solutions increases with increasing protein concentration^23^. Accordingly, reduced T2DF signal intensity is consistent with a relative reduction in protein content or protein-to-water ratio. One of the most prominent findings of the vasomechanic intervention was the whole-brain darkening of T2DF MRI signal extending into deep structures (Figs. 1f and 3f). Here, darkening of the whole human T2DF image may in theory reflect either a reduced amount of tissue-bound water resulting in less ^1^H protons to give MR signal, a reduced soluble protein concentration affecting the water molecules less, or both phenomena. In light of the quantified hemodilution, the increased soluble protein level in the blood laboratory results, in addition to the convergent fNIRS hydrodynamic brain sensing data, these results may most likely reflect the concerted redistribution of soluble proteins along with removal of brain water, i.e., solutes, from the CSF compartment (Figs. 1f–h and 3h)^30^ into the blood (Fig. 3l).

As NO-induced hemodilution normally causes a drop in blood protein concentration^27^, the opposing increase we detected in low molecular weight soluble brain-derived proteins (<68 kDa) in blood (Fig. 1d) strongly suggests an increased brain-derived solute efflux directly into the blood. This may well be accompanied by a transient BBB-related permeability effect. The dcEEG level that typically reflects BBB permeability further suggests that BBB permeability could have been increased^61^. Notably, T2DF signal intensities in the posterior venous sinus showed a linear correlation with quantified circulating blood protein concentrations (Fig. 1m), linking the imaging findings more directly to blood biomarkers, suggesting a more posterior compartmentalized efflux mechanism in humans.

The low-intensity T2DF compartments of posterior venous sinuses and adjacent PSD regions showed increased T2DF intensity following the vasomechanic intervention, supporting the notion that enriched local protein concentration increased the T2DF signal, as indicated by Albayram et al. (2022) in the PSD spaces^23^ (Fig. 1k, l). In contrast, the more frontally-positioned parasagittal/para-cavernous dural CSF regions displayed reduced, i.e., an even darker T2DF signal, consistent with simultaneous water dilution that is suggestive of either increased protein removal or increased dilution of CSF water (Figs. 1k and 4j).

To the best of our knowledge, differential solute efflux according to cranial position has not been extensively characterized in humans, and the physiological and anatomical mechanisms underlying these observations remain unclear. There could possibly be anatomical and cerebrofluidic pressure-dependent differences in the BBB and PSD permeability favoring efflux in dorsal regions. Mice have regionally differential basal meningeal lymphatic vessels and valves that are permissive to greater tracer efflux compared to dorsal meningeal vessels^62^. Furthermore, in the supine position there is greater hydrostatic pressure towards the posterior areas, which, together with the increased ICP during arterial vasodilation (Figs. 1 and 3) could favor posterior solute drainage in the compartmentalized subarachnoid space CSF towards the posterior venous sinuses in the human cranium^22,63,64^.

Furthermore, the middle vertex PSD CSF-filled space showed quite a persistent T2DF signal alteration, with a mean interval between measurements of 9.4 months (range 0.5–22.3 months, Supplementary Fig. 23), although most of the T2DF signal intensity changes were transient over much of the brain. This seems to be in line with the notion that these superior regions may have inherently slower solute in/outflow processes, as already suggested by Eide and co-workers in humans^22^. Here, we show that changes in PSD solute content, once established, appear to persist for months. This slow nature of solute exchange could be related to hydrostatic effects in the low-pressure environment of venous drainage. Even in the recumbent position, hydrostatic pressure within the highest vertical part of the superior sagittal sinus remains relatively elevated compared with lower venous sinuses, which could in theory promote pressure-driven solute transport toward posterior regions, consistent with the findings shown in Figs. 1 and 3.

In theory, the observed treatment-evoked T2DF changes could reflect technical Rician noise in low-intensity T2DF signal regions resulting from differences in subject positioning or T2-related flow effects between scans. However, such noise artifacts should be present uniformly in a given area of the T2DF image. Instead, the T2DF changes were highly compartment-specific and not present uniformly in the brain, as would be in the Rician noise case. Neighboring supracellar cistern and other low-intensity CSF areas showed no changes, while adjacent sinus rectus showed increased T2DF signal intensity, which argues against the interpretation of Rician noise artifact (Figs. 1h, l, 3h, 4h and Supplementary Figs. 7, 15, 17, 19, 21).

Increased CSF flow velocity can also increase T2DF signal intensity in narrowed CSF spaces. However, most global brain changes indicate a darkening of the T2DF signal. We have previously shown that an increase in brain water flow velocity should coincide with a simultaneous increase in pulsation power within both brain tissue and CSF^29,45,48^. First of all, during NO-induced vasodilation, MREG power, particularly in the vasomotor and respiratory frequency ranges, increased over wide brain areas. In these regions, the T2DF signal would be expected to increase rather than exhibit the observed darkening, if the signal changes were caused by increased flow. Following the combined intervention, most pulsation power changes had subsided, suggesting that the observed T2DF signal increases in the posterior venous sinuses and other regions are more likely related to increased blood protein content than to increased flow velocity. Furthermore, the T2DF signal intensity increases were observed only in the posterior venous sinuses and PSD regions, with absent changes in the middle vertex sagittal sinus area after the vasomechanic intervention (Fig. 1k, l). The detected cardiovascular pulsation power increases sparsely in the frontal brain areas are non-overlapping and relatively distant from the posterior venous sinuses (Fig. 2c).

The extent of increased brain solute efflux of Aβ & GFAP in healthy humans closely followed the magnitude of the blood pressure drop and age (Fig. 1e and Supplementary Fig. 5)^41^. The simultaneously acquired fNIRS signal confirmed that brain water content indeed tracked the blood pressure drop during NO vasodilation (Fig. 2d). The changes in brain arterial HbO and slower venous HbR shifts were counteracted by the brain surface H_2_O signal, mostly reflecting subarachnoid CSF, in keeping with the Monro-Kellie principle governing intracranial fluid compartment dynamics.

Taken together, the convergent T2DF MRI, physiological brain sensing data and blood biomarker findings are consistent with a redistribution of brain-derived proteins toward the venous blood compartment induced by the combined vasomechanic intervention. While these results may suggest the presence of enhanced solute movement from brain to blood circulation, they should be interpreted cautiously in light of our currently limited mechanistic knowledge of the solute clearance pathway. The complementary murine experiments provide further biological support for the overall framework, rather than directly validating the specific mechanisms inferred from the human data. We conclude that the multimodal findings are consistent with increased human brain solute efflux following combined NO-mediated vasodilation and Piezo1-targeted mechanotransductive stimulation, which was safely implemented under controlled experimental conditions.

## Methods

All human data were collected between 11/2021–10/2023. The study protocol was approved by the National Committee on Medical Research Ethics (Tukija), Finnish Medicines Agency (Fimea) and the Regional Ethics Committee of the Northern Ostrobothnia Hospital District, and is registered to the EU Clinical Trials Register (clinicaltrialregister.eu, EudraCT number 2021-000625-27). The study was carried out in accordance with the Medical Device Regulation (EU 2017/745), European Commission Directives on Clinical Trials (2001/20/EC) and Good Clinical Practice (2005/28/EC), Finnish National Legislation on Clinical Trials on Medicinal Products (488/1999) and Medicines (395/1987), ethical guidelines of the Helsinki Declaration and the General Data Protection Regulation (EU 2016/679).

### Participants

The study involved 29 healthy volunteers (mean age: 44 ± 10 years; 15 females, 52%). Participants were recruited through email advertisements distributed to the personnel mailing lists of the University of Oulu and Oulu University Hospital. Inclusion criteria required participants to be healthy volunteers not using regular medication, except contraceptives or thyroid hormone replacement therapy (thyroxine). Exclusion criteria were pregnancy or breastfeeding, migraine or recurrent headache, substance or alcohol abuse, smoking or using tobacco products, known allergy to glyceryl trinitrate or other nitrates, current use of erectile dysfunction medication (sildenafil, vardenafil, or tadalafil), or contraindications for magnetic resonance imaging (including metal foreign bodies, severe claustrophobia, or electronic implants). Prospective participants contacted researchers voluntarily, underwent an initial phone interview, and subsequently attended an enrollment visit. During the enrollment visit, participants received detailed information about the study, voluntarily signed informed consent, and underwent medical screening by a physician to confirm their eligibility and ensure adequate health status for study participation. Participants were instructed to abstain from caffeinated beverages (e.g., coffee, energy drinks) and alcohol for at least 12 h prior to each study visit. Participants did not receive financial compensation for study participation.

### Study design

Eligible participants completed two intervention visits in randomized order: (1) NO-mediated vasodilation with whole-body vibration (NO+WBV_p_) and (2) Piezo1-targeted whole-body vibration (WBV_p_). After the first visit, each participant crossed over to the alternate intervention, resulting in a randomized crossover design. A washout period of at least seven days separated the visits. The study flow is summarized in the CONSORT diagram (Supplementary Fig. 1).

To investigate the acute effect of NO-mediated vasodilation (NO) alone in more detail (i.e., without WBV_p_), an additional NO-only session was conducted in a subset of participants (*n* = 12). This session was performed as an exploratory sub-study and was not part of the randomized crossover design. Accordingly, analyses involving this session were considered exploratory and interpreted separately from the primary endpoint family.

In addition, a separate supplementary cohort (*n* = 13) underwent a sham intervention without NO-mediated vasodilation or whole-body vibration. This cohort was used to assess changes in T2DF MRI signal intensity in the absence of active intervention and was analyzed in an exploratory manner, separately from the primary endpoint family.

Given the multimodal and exploratory design, we defined a primary endpoint family consisting of (i) circulating concentrations of brain-derived proteins, (ii) T2DF signal intensity changes and (iii) brain pulsation changes due to interventions. All other analyses were considered exploratory and interpreted accordingly. Methods for the mouse study are presented in Supplementary Text 3.

The study protocol included additional assessments such as the PAL memory test, sleep questionnaire, and BDI-21, results of which are not included in the present analysis. These metrics were obtained to ensure general health status and suitability of participants, whereas the current manuscript focuses on physiological, imaging, and blood biomarker outcomes.

### Study drug

Sublingual nitroglycerin (glyceryl trinitrate, GTN, 0.5 mg per tablet; Nitroglycerin Orifarm®, Orifarm Healthcare a/s) was used as the study drug to cause vasodilation. GTN is an organic nitrate vasodilator that undergoes bioactivation to release nitric oxide (NO), which stimulates soluble guanylate cyclase in vascular smooth muscle cells, increasing intracellular cyclic guanosine monophosphate levels and thereby inducing smooth muscle relaxation. This mechanism produces potent vasodilation in arteries, with a predominant effect on venous capacitance vessels and significant dilation of large arteries (including the coronary arteries). When administered sublingually, GTN is rapidly absorbed through the oral mucosa, achieving peak plasma concentrations in approximately 2–5 min. The drug’s onset of action is thus almost immediate, and its hemodynamic effects persist for about 30–60 min. In clinical practice, sublingual GTN is widely used for the acute relief of angina pectoris.

In the combined NO+WBV_p_ intervention, nitroglycerin was administered as two sublingual 0.5 mg doses. The first dose was given during MRI acquisition, and the second dose was administered approximately 20 min later during the WBV_p_ treatment period. In the NO-only intervention, a single sublingual 0.5 mg nitroglycerin dose was administered.

### Whole-body vibration equipment

The Piezo1-targeting^49^ of the direct whole-body mechanotransductive stimulation was achieved by specific modulation of the 22–50 Hz whole-body vibration. The stimulus intensity modulation was carried out such that the Piezo1 mechanoreceptors would not saturate as they do with continuous tonic stimulation, but rather aiming to engage maximal triggering stimulus responses to > 20 Hz stimuli after a refractory period, as shown in Fig. 4c. The stimulus was delivered with a commercially available whole-body vibration device (Neurosonic Mobile Mattress Basic, Oy Neurosonic Finland Ltd).

### Multimodal data acquisition and preprocessing

Multimodal data were acquired simultaneously using magnetic resonance encephalography (MREG), MRI-compatible functional near-infrared spectroscopy (fNIRS), continuous non-invasive blood pressure (NIBP), cuff-based blood pressure monitoring, and direct-current electroencephalography (dcEEG). All modalities were synchronized using scanner trigger pulses^65^. Data were collected during a resting-state protocol, with subjects awake and fixating their gaze on a cross displayed on a screen. All data were acquired at Oulu University Hospital, Finland.

Preprocess steps and analyses were performed using MATLAB (R2024b, The MathWorks, Natick, MA), Functional MRI of the Brain Software Library (FSL, version 6.0.7.16)^66^ and Analysis of Functional NeuroImages (AFNI; version 18.0.05)^67^.

#### (f)MRI data acquisition and preprocessing

MRI data were acquired using a Siemens MAGNETOM Vida 3 T scanner (Siemens Healthineers AG, Erlangen, Germany) equipped with a 64-channel head coil. High-resolution anatomical T2 dark-fluid (T2DF) weighted images were obtained using a standard Siemens DF space imaging in the sagittal plane (repetition time (TR) = 5000 ms, echo time (TE) = 387 ms, inversion time (TI) = 1800 ms, field-of-view (FOV) = 240 mm, flip angle (FA) = 120°, voxel size (VS) = 0.45 × 0.45 × 0.9 mm^3^).

Dark-Fluid (T2DF) MRI is one of the most important clinical MRI sequences as it is sensitive to edematous brain tissue water, which is detected as high signal intensity. The high T2DF signal stems from an increased amount of water-^1^H surrounding brain macromolecules such as proteins. These sequences use an inversion pulse applied at a specific inversion time (in humans at 37 °C, TI = 1800 ms), which nullifies signal from free-moving, unbound CSF water ^1^H protons in DF images, whereas water affected by spin interactions with macromolecules such as proteins has a shorter T1 time and therefore gives higher signal. Once the excess tissue water from an acute tissue edema resolves, the high T2DF signal darkens back to normal levels. In our study, the DF images were used to quantify changes in the brain tissue protein/water ratio caused by the interventions targeting vascular and perivascular structures.

The DF MRI was recently shown to also be advantageous for high-precision estimation of key glymphatic structures like the parasagittal dura (PSD)^22^. As shown in a free water phantom experiment by Albayram et al. (2022)^23^, increasing levels of protein (or other macromolecules) within freely moving distilled water increases the dark-fluid signal intensity. In the free-moving CSF and blood that appear as dark fluids in T2DF MRI, an increase in the signal may reflect an increase in the amounts of macromolecules, such as soluble proteins, within the dark fluid. Although T2 flow effects can also increase the DF signal in fast-flowing narrow CSF spaces such as the narrow aqueducts and sulci, we avoided such narrowed spaces in our 3D ROI analyses by choosing broader CSF areas. Darkening tissue DF signal reflects a reduced amount of brain tissue water ^1^H in association with macromolecules, i.e., either free water ^1^H giving the MR signal, or associations with macromolecules changing the T1 relaxation of the ^1^H, or both.

For further analysis, we used the FSL Brain Extraction Tool (BET) with neck and bias field correction for brain extraction from T2DF images. To confirm optimal quality, the extracted brain images were visually inspected.

T2-weighted images were obtained using Siemens space imaging in the sagittal plane (TR = 3200 ms, TE = 412 ms, FOV = 240 mm, FA = 120°, and VS = 0.94 × 0.94 × 0.9 mm^3^) for defining lymph nodes and CSF-filled spaces within the dura from T2DF images (Supplementary Fig. 24). T1-weighted images were obtained using a 3D MPRAGE sequence (TR = 1900 ms, TE = 2.49 ms, inversion time = 900 ms, FOV = 240 mm, FA = 9°, and VS = 0.94 × 0.94 × 0.9 mm^3^) for registration and for defining lymph nodes from T2DF images (Supplementary Fig. 24). FSL BET with neck and bias field correction was used to extract the brain from the T1 images for registration of functional data to Montreal Neurological Institute (MNI152) space.

An ultrafast magnetic resonance encephalography (MREG) sequence was used to capture physiological brain pulsations at a temporal resolution of 10 Hz. MREG consists of a 3D single-shot stack-of-spirals sequence that under-samples k-space, enabling rapid whole-brain coverage^68^. The scanning parameters were: repetition time (TR) = 100 ms, echo time (TE) = 36 ms, flip angle (FA) = 25°, field of view (FOV) = 192 mm, voxel size (VS) = 3 × 3 × 3 mm^3^, and scanning time 5 min. Images were reconstructed using L2-Tikhonov regularization with *λ* = 0.1, determined via the L-curve method, and using a MATLAB-based reconstruction tool provided by the sequence developers^69^.

To minimize T1-relaxation effects, the first 180 time points were removed from the MREG data. MREG data were preprocessed with the FSL pipeline using high-pass filtration with a cut-off frequency of 0.008 Hz, and FSL MCFLIRT for motion correction. Additionally, the motion artifacts were minimized by excluding data that exceeded the following parameters: mean relative motion > 0.06 mm, mean absolute motion > 0.4 mm, or absolute motion in any timepoint > 2 mm. MREG data were spatially smoothed with a 5 mm full width at half maximum Gaussian kernel. Any remaining motion-produced spikes were removed with AFNI. Finally, the MREG data were registered into MNI152 standard space using FSL for further analysis.

#### EEG and physiological data acquisition and preprocessing

Electroencephalographic (EEG) signals were acquired using a 32-channel MR-compatible direct-current dcEEG (Brain Products GmbH, Gilching, Germany). DcEEG recordings were obtained with a BrainCap MR EEG cap (EasyCap GmbH, Herrsching, Germany), in which Ag/AgCl electrodes were positioned according to the international 10–20 system. An electrolyte gel (ABRALYT HiCl, EasyCap GmbH) was applied between the electrodes and the scalp to ensure stable contact and to minimize impedance, which was maintained below 10 kΩ for all electrodes. DcEEG signals were recorded during MRI scanning using Brain Vision Recorder (Brain Products GmbH), and during WBV_p_ stimulation using the NeuroOne system (Bittium Corporation, Oulu, Finland), with a sampling rate of 5 kHz in both settings. Prior to measurements, we performed a visual inspection of signal quality. During MRI, the acquired dcEEG data (during NO intervention, *n* = 12) were preprocessed with Brain Vision Analyzer (v.2.1, Brain Products) to remove gradient and ballistocardiographic artifacts through template subtraction^70^. Bad channels were identified by computing with MATLAB the standard deviation and maximum amplitude of each channel’s high-pass filtered (cutoff frequency 0.1 Hz) time series; channels with both SD > 2 and maximum amplitude > 1000 μV were excluded (0−1 channels per recording, 0.8% overall). Filtered data were used only for identification of bad data to retain the DC level. Because the polarity of DC shifts in EEG differs between individuals^71^, we inverted the raw signals (multiplied by -1) so that slow shifts appear as upward (positive) deflections (0–31, 49.9%). After that, data were downsampled to 500 Hz, and a linear/quadratic trend was removed with MATLAB.

For dcEEG data acquired during the WBV_p_ mechanical stimulus, bad channels were identified by computing the standard deviation and maximum amplitude of each channel’s band-pass filtered (0.1–22 Hz) time series; channels with both SD > 2 and maximum amplitude > 1000 μV were excluded (NO + WBV_p_ intervention: 0–4 channels per recording, 3.2% overall; WBV_p_ intervention: 0–3 channels per recording, 3.1% overall). Next, the raw data were preprocessed in MATLAB using low-pass filtering with a cut-off frequency of 22 Hz to remove the vibration artifact.

Physiological signals were recorded in synchrony with fMRI^65^, including respiration with a scanner respiratory belt and heart rate with scanner fingertip photoplethysmography (PPG). Individual cardiorespiratory frequencies were calculated in MATLAB. Cuff-based blood pressure (systolic/diastolic) was recorded with an anesthesia monitor (GE Datex-Ohmeda Aestive 5).

#### (f)NIRS data acquisition and preprocessing

FNIRS data were collected using an MRI-compatible fiber-optic system^65^ at a sampling rate of 800 Hz. High-power LEDs generated monochromatic light at wavelengths of 690, 830, and 980 nm. FNIRS optodes were attached to each subject’s forehead (right and left) at the source-detector distance of 3 cm to reach the brain cortex^72^. Raw measured signals were processed to quantify, with MATLAB, the concentrations of oxygenated brain blood (HbO), deoxygenated brain blood (HbR), and cerebral CSF water below the skull (H_2_O) using a modification of the Beer-Lambert law^72^. The total hemoglobin (HbT) signal was calculated as a sum of HbO and HbR signals. Afterward, the group means (and standard deviation) was calculated for the normalized right and left signals.

#### NIBP data acquisition and preprocessing

Continuous non-invasive blood pressure (NIBP) was measured using a fiber-optic accelerometer system optimized for MRI compatibility with a sampling rate of 1 kHz^65^. Two sensors were attached to the sternum over the aortic valve and carotid artery to sense skin movements caused by cardiovascular pulses. Pulse transit times were calculated between the sensors for each recording.

### Multimodal data analysis

#### fMRI data analysis

For the MREG recordings, we used complex Morlet wavelets in wavelet convolution to calculate the time-frequency spectrogram^29^ with a fixed number of wavelet cycles (*N* = 8) and logarithmic frequency ranging from 0.01–5 Hz with 50 equal steps. To reduce computational cost, we randomly selected 5% (3405 voxels) of brain voxels.

Global fast Fourier transform (FFT) power density maps were calculated using AFNI for the full-band MREG data, followed by application of FSL to calculate the average FFT spectrum of all brain voxels. The sums of VLF, LF, individual respiratory, and cardiac powers were calculated with FSL and AFNI.

1. The VLF range of 0.008–0.08 Hz was chosen to obtain the maximum possible coverage of the very-low frequencies, without overlapping with LF and respiratory frequencies.
2. The LF (0.08–0.15 Hz) band was selected to capture low-frequency ~0.1 Hz vasomotor waves without overlapping with respiration.
3. Respiratory and cardiac ranges and peak frequencies were obtained from individual FFT spectra without harmonics or overlapping with other physiological frequency bands (Supplementary information Text 1). Frequencies identified from the MREG data were additionally double-checked against the scanner’s physiological signals (respiration belt and photoplethysmogram) solely for validation purposes, to ensure correct frequency identification (Supplementary Fig. 26).

Paired statistical tests excluded datasets exhibiting physiological frequency overlap, specifically for cases in which the respiratory frequency range overlapped into the VLF band. The resulting sample sizes for each comparison are reported in Supplementary Fig. 27.

#### Anatomical T2 dark-fluid MRI data analysis

1. Whole-brain voxelwise comparison: T2DF brain-extracted and bias-field corrected volumes were compared between pre- and post-intervention scans.
2. CSF/brain-tissue segmentation: coarse two-class segmentation masks (CSF vs brain tissue) were generated with FSL for brain-extracted and bias-field-corrected T2DF images. Masks were refined using FSL with threshold values < 70 DF intensity (arb. units) defined as CSF and > 70 arb. units defined as brain tissue. All masks were visually inspected for quality and anatomical validity. A representative example of the masks can be found in Supplementary Fig. 25. Next, we calculated the mean intensity values and volumes with FSL.
3. Region-of-interest (ROI) analysis: ROI analysis was performed on T2DF images to obtain the individual mean intensity within each ROI before and after the interventions. For the original T2DF images, ROIs were delineated individually for each subject and scan using a diagnostic DICOM  medical image viewer Neaview, Vitec Neagen Co, with anatomical guidance from co-registered T1- and T2-weighted images (Supplementary Figs. 24 and 25). To ensure accurate longitudinal comparison, ROIs were placed in identical anatomical slices and orientation between pre- and post-intervention images. For each ROI, the largest possible anatomically valid region was selected to maximize signal stability and minimize voxel-level variability. Adjacent anatomical structures (sagittal sinus and parasagittal dura CSF-filled spaces) were delineated within the same slice. The following ROIs were manually defined: subarachnoid CSF beneath the parasagittal dura, middle/vertex parasagittal dura CSF-filled space, posterior parasagittal dura CSF-filled space, para-cavernous dura CSF-filled space, posterior and middle sagittal sinus, and superficial cervical lymph nodes. In addition, ROIs for lateral ventricles, superior cisterna, fourth ventricle, and sinus rectus were generated using FSL (FSLeyes) with threshold-based masks for brain-extracted and bias-field-corrected T2DF images. These masks were defined individually for each subject and applied identically to both pre- and post-intervention images (Supplementary Fig. 25).

#### EEG data analysis

For dcEEG acquired during vibration, we used complex Morlet wavelets in wavelet convolution to calculate a time-frequency spectrogram^29^ with a fixed number of wavelet cycles (*N* = 8) and a frequency range from 0.1 to 22 Hz in equal 220 steps for each dcEEG channel. We next calculated the group average dcEEG spectrogram, and then the corresponding alpha power (8–12 Hz). For dcEEG acquired during MRI, we calculated group means (and SD).

#### NIBP data analysis

PTT is inversely related to blood pressure and can be used for cuffless blood pressure estimation^73^. Generally, as blood pressure increases, the arterial walls become stiffer, leading to faster pulse wave velocity and shorter PTT. From each PTT data set, we calculated a moving average in MATLAB, and subsequently the group means (and standard error of the mean).

### Blood tests

Venous blood was drawn immediately before and after the NO+WBV_p_ intervention. Participants underwent the pre-intervention blood sampling prior to any active intervention procedures. The interval between the pre-blood sample and the pre-T2DF MRI acquisition was approximately 1 h, during which time participants were prepared for the measurements (e.g., instrumentation and setup) without exposure to active interventions. The post-intervention blood sample was collected approximately 10–15 min after the post-T2DF MRI acquisition, corresponding to approximately 1.5 h after sublingual NO (0.5 mg) administration. EDTA-plasma and serum were separated and aliquoted into Sarstedt polypropylene tubes, frozen and stored at –70 °C, and shipped to the Eastern Finland Biomarker laboratory for analysis. Biomarkers were quantified on Quanterix’s Single Molecule Array (Simoa) HD-X Analyzer (Quanterix, Billerica, MA, USA) using the Neurology 4-PlexE assay (Aβ_40_, Aβ_42_, GFAP, NF-light) and the pTau181 Advantage V2.1 assay^24^. Serum results are presented in Fig. 1, with concordant plasma results provided in Supplementary Fig. 3. In subsequent blood biomarker correlation analyses, only statistically significant correlations are illustrated in the figures. All tested correlations, including non-significant results, are provided in the Supplementary Information and Source Data file. For safety monitoring, the serum hs-CRP and S100B levels were measured by Nordlab (Oulu, Finland).

To evaluate the potential NO-induced hemodilution, a complete blood count (including hemoglobin, hematocrit, red and white blood cells and platelets) was obtained immediately before and after the NO intervention (*n* = 10). The pre-intervention blood sample was collected approximately 1 h prior to imaging, during which time no active intervention was performed and only preparatory procedures were carried out. The post-intervention blood sample was collected approximately 1 h after sublingual NO (0.5 mg) administration. The analyses were performed by Nordlab (Oulu, Finland). A detailed description of the derivation and calculation of the hemodilution correction factor is provided in Supplementary Information Text 2.

### Statistical analysis

Voxelwise comparisons between brain pulsation power maps and T2DF maps were performed by a two-sided paired *t*-test using a non-parametric threshold-free permutation test (5000 permutations) with FSL’s randomize^74^, and were corrected for the family-wise error rate (FWER) at a significance level of *P* < 0.05.

For the blood pressure data, segmented and ROI-based T2DF data, and blood results, we used the Shapiro-Wilk test to examine data normality. For normally distributed data, we applied a paired *t*-test, and for non-normally distributed data, the Paired-sample Wilcoxon signed-rank test, using OriginPro 2023b for the statistical analyses.

To control for the number of false positives among significant results of segmented and ROI analysis (*n* = 24) and blood results (*n* = 25) in the NO+WBV_p_ condition, we used Benjamini-Hochberg false discovery rate (FDR) correction^75^ with MATLAB.

The significance threshold for all tests was *P* < 0.05. All data were reported as mean ± standard deviation (SD) unless otherwise indicated. Non-normally distributed data were reported as median (interquartile range, IQR). In Fig. 1e, outliers in the Pearson’s correlation analysis (>2.5 SD, 3 Ab40 & −42 and 1 GFAP) were masked before the final correlation analysis (Supplementary Fig. 4). The resulting sample sizes for each multimodal analysis are reported in Supplementary Fig. 22.

### Reporting summary

Further information on research design is available in the Nature Portfolio Reporting Summary linked to this article.

## Supplementary information

Reporting Summary

Supplementary Information

Transparent Peer Review file

## Source data

Source Data

## Data Availability

Large source datasets are available at https://github.com/johannatuunanen/Vasomechanic_treatment_is_associated_with_increased_efflux_of_human_brain_proteins. Sharing of individual-level MRI/fMRI data is governed by the Finnish Act on the Secondary Use of Health and Social Data and the European Union’s General Data Protection Regulation (GDPR), which restricts access when there is a risk of re-identification. Accordingly, data sharing is considered case-by-case upon reasonable request. Pseudonymized individual MRI/fMRI datasets may be shared with qualified academic collaborators after ethical review and data protection assessment, and subject to an approved data usage agreement. Due to these legal constraints, the original raw MRI/fMRI datasets cannot be deposited in a public repository. Requests should be directed to the corresponding author (J.T., V.Ki.). The timeframe for response to such requests is within 1–3 months. Source data are provided with this paper.
