# Supplementary material for: Vasomechanic treatment is associated with increased efflux of human brain proteins
Source: Nat Commun. 2026 Nov 3;17:11164. doi: 10.1038/s41467-026-78894-y (PMC13731658; doi:10.1038/s41467-026-78894-y)
Supplement: Supplementary file 1 [file 41467_2026_78894_MOESM1_ESM.pdf]

nature portfolio

Corresponding author(s): Johanna Tuunanen, Vesa Kiviniemi

Last updated by author(s): Sep 10, 2026

Reporting Summary

Nature Portfolio wishes to improve the reproducibility of the work that we publish. This form provides structure for consistency and transparency in reporting. For further information on Nature Portfolio policies, see our Editorial Policies and the Editorial Policy Checklist.

Statistics

For all statistical analyses, confirm that the following items are present in the figure legend, table legend, main text, or Methods section.

n/a	Confirmed	
☐	☒	The exact sample size (*n*) for each experimental group/condition, given as a discrete number and unit of measurement
☐	☒	A statement on whether measurements were taken from distinct samples or whether the same sample was measured repeatedly
☐	☒	The statistical test(s) used AND whether they are one- or two-sided<br>*Only common tests should be described solely by name; describe more complex techniques in the Methods section.*
☒	☐	A description of all covariates tested
☐	☒	A description of any assumptions or corrections, such as tests of normality and adjustment for multiple comparisons
☐	☒	A full description of the statistical parameters including central tendency (e.g. means) or other basic estimates (e.g. regression coefficient) AND variation (e.g. standard deviation) or associated estimates of uncertainty (e.g. confidence intervals)
☐	☒	For null hypothesis testing, the test statistic (e.g. *F*, *t*, *r*) with confidence intervals, effect sizes, degrees of freedom and *P* value noted<br>*Give P values as exact values whenever suitable.*
☒	☐	For Bayesian analysis, information on the choice of priors and Markov chain Monte Carlo settings
☒	☐	For hierarchical and complex designs, identification of the appropriate level for tests and full reporting of outcomes
☒	☐	Estimates of effect sizes (e.g. Cohen's *d*, Pearson's *r*), indicating how they were calculated

Our web collection on statistics for biologists contains articles on many of the points above.

Software and code

Policy information about availability of computer code

Data collection	Siemens VIDA 3T MRI systems, MREG fMRI sequence, functional near-infrared spectroscopy (fNIRS), non-invasive blood pressure (NIBP) devices, a 32-channel EEG (Brain Products GmbH, Gilching, Germany & NeuroOne system Bittium Corporation, Oulu, Finland), anesthesia monitor (GE Datex-Ohmeda Aestive 5), and Simoa HD-X Analyzer (Quanterix, Neurology 4-PlexE and pTau181 Advantage V2.1).
Data analysis	Data analyses were performed using MATLAB (MathWorks, R2024b), FSL (FMRIB Software Library, version 6.0.7.16), AFNI (version 18.0.05), OriginPro (2023b), Brain Vision Analyzer(v.2.1, Brain Products).

For manuscripts utilizing custom algorithms or software that are central to the research but not yet described in published literature, software must be made available to editors and reviewers. We strongly encourage code deposition in a community repository (e.g. GitHub). See the Nature Portfolio guidelines for submitting code & software for further information.

Data

Policy information about availability of data

All manuscripts must include a data availability statement. This statement should provide the following information, where applicable:
- Accession codes, unique identifiers, or web links for publicly available datasets
- A description of any restrictions on data availability
- For clinical datasets or third party data, please ensure that the statement adheres to our policy

Source data are provided with the paper. Sharing of individual-level MRI/fMRI data is governed by the Finnish Act on the Secondary Use of Health and Social Data and the European Union's General Data Protection Regulation (GDPR), which restricts access when there is a risk of re-identification. Accordingly, data sharing is

considered case-by-case upon reasonable request. Pseudonymized individual MRI/fMRI datasets may be shared with qualified academic collaborators after ethical review and data protection assessment, and subject to an approved data usage agreement. Due to these legal constraints, the original raw MRI/fMRI datasets cannot be deposited in a public repository. Requests should be directed to the corresponding author (J.T., V.Ki.). The timeframe for response to such requests is within 1-3 months.

Research involving human participants, their data, or biological material

Policy information about studies with human participants or human data. See also policy information about sex, gender (identity/presentation), and sexual orientation and race, ethnicity and racism.

Reporting on sex and gender	Sex was recorded as a biological variable and is reported throughout the manuscript. The study included healthy adult volunteers (15 females and 14 males). No gender-related variables were collected or analyzed. Owing to the limited sample size, sex-stratified analyses were not performed.
Reporting on race, ethnicity, or other socially relevant groupings	Race, ethnicity, and other socially relevant groupings were not recorded or analyzed in this study. These variables were not part of the study design or objectives.
Population characteristics	The study included healthy adult volunteers recruited from the Oulu region, Finland. Participants were screened for eligibility according to predefined inclusion and exclusion criteria. Demographic characteristics, including age and sex, are reported in the manuscript. The main randomized crossover cohort consisted of 29 healthy participants aged 23–65 years.
Recruitment	Recruitment was conducted via an announcement sent to the email lists of the University of Oulu and the Northern Ostrobothnia Hospital District. Written informed consent was obtained from all participants. Participants did not receive financial compensation for study participation. As participation was voluntary, some self-selection bias cannot be excluded, and the study population may represent individuals with greater interest in health and research than the general population. The study was conducted in a relatively small cohort of healthy volunteers, which may limit generalizability to patient populations. A randomized crossover design was used to reduce inter-individual variability and potential allocation bias.
Ethics oversight	The study protocol was approved by National Committee on Medical Research Ethics (Tukija), Finnish Medicines Agency (Fimea) and Regional Ethics Committee of the Northern Ostrobothnia Hospital District.

Note that full information on the approval of the study protocol must also be provided in the manuscript.

Field-specific reporting

Please select the one below that is the best fit for your research. If you are not sure, read the appropriate sections before making your selection.

[x] Life sciences [ ] Behavioural & social sciences [ ] Ecological, evolutionary & environmental sciences

For a reference copy of the document with all sections, see nature.com/documents/nr-reporting-summary-flat.pdf

Life sciences study design

All studies must disclose on these points even when the disclosure is negative.

Sample size	This study was designed as an exploratory pilot study investigating the physiological effects of vasomechanic interventions in healthy human volunteers. No formal a priori sample size calculation was performed. Sample size was determined based on feasibility, participant availability, and previous experience from related physiological imaging studies. The primary randomized crossover cohort comprised 29 participants. Additional exploratory NO-only (n = 12) and sham-control (n = 13) cohorts were analyzed separately.
Data exclusions	Data exclusions were predefined and based on modality-specific data quality criteria. For some analyses, participant numbers differed across modalities owing to missing measurements, technical failures, motion artefacts, or insufficient data quality. The exact sample size for each analysis is reported in the Results section and corresponding figure legends.
Replication	The study used repeated within-subject measurements in a randomized crossover design. The main findings were supported across multiple independent measurement modalities, including T2DF MRI, blood biomarkers, fNIRS, and MREG. Additional NO-only and sham-control cohorts provided independent supporting observations. No findings failed reproduction within the study dataset.
Randomization	Participants were allocated by simple randomization to determine the order of the two study visits (A or B). After completing the first intervention, all participants underwent the alternate intervention, resulting in a randomized crossover design. No blinding was implemented during data collection or analysis.
Blinding	Blinding was not used during data acquisition. Manual ROI analyses were performed with knowledge of the intervention condition because accurate anatomical identification of specific dural, venous, and CSF structures required reference to the complete imaging dataset. Therefore, blinding was not feasible for these analyses. Automated image-processing and statistical pipelines were applied identically to all datasets, minimizing the potential for observer bias.

Reporting for specific materials, systems and methods

We require information from authors about some types of materials, experimental systems and methods used in many studies. Here, indicate whether each material, system or method listed is relevant to your study. If you are not sure if a list item applies to your research, read the appropriate section before selecting a response.

Materials & experimental systems

n/a	Involved in the study
☐	☐ Antibodies
☐	☐ Eukaryotic cell lines
☐	☐ Palaeontology and archaeology
☐	☒ Animals and other organisms
☐	☒ Clinical data
☐	☐ Dual use research of concern
☐	☐ Plants

Methods

n/a	Involved in the study
☐	☐ ChIP-seq
☐	☐ Flow cytometry
☐	☒ MRI-based neuroimaging

Antibodies

Antibodies used: *Describe all antibodies used in the study; as applicable, provide supplier name, catalog number, clone name, and lot number.*

Validation: *Describe the validation of each primary antibody for the species and application, noting any validation statements on the manufacturer's website, relevant citations, antibody profiles in online databases, or data provided in the manuscript.*

Eukaryotic cell lines

Policy information about cell lines and Sex and Gender in Research

Cell line source(s): *State the source of each cell line used and the sex of all primary cell lines and cells derived from human participants or vertebrate models.*

Authentication: *Describe the authentication procedures for each cell line used OR declare that none of the cell lines used were authenticated.*

Mycoplasma contamination: *Confirm that all cell lines tested negative for mycoplasma contamination OR describe the results of the testing for mycoplasma contamination OR declare that the cell lines were not tested for mycoplasma contamination.*

Commonly misidentified lines (See ICLAC register): *Name any commonly misidentified cell lines used in the study and provide a rationale for their use.*

Palaeontology and Archaeology

Specimen provenance: *Provide provenance information for specimens and describe permits that were obtained for the work (including the name of the issuing authority, the date of issue, and any identifying information). Permits should encompass collection and, where applicable, export.*

Specimen deposition: *Indicate where the specimens have been deposited to permit free access by other researchers.*

Dating methods: *If new dates are provided, describe how they were obtained (e.g. collection, storage, sample pretreatment and measurement), where they were obtained (i.e. lab name), the calibration program and the protocol for quality assurance OR state that no new dates are provided.*

☐ Tick this box to confirm that the raw and calibrated dates are available in the paper or in Supplementary Information.

Ethics oversight: *Identify the organization(s) that approved or provided guidance on the study protocol, OR state that no ethical approval or guidance was required and explain why not.*

Note that full information on the approval of the study protocol must also be provided in the manuscript.

Animals and other research organisms

Policy information about studies involving animals; ARRIVE guidelines recommended for reporting animal research, and Sex and Gender in Research

Laboratory animals: 2-3 months old C56 Bl6/N (Charles River) female mice were used in all experiments.

Wild animals: The study did not involve wild animals

Reporting on sex: Only female mice were used in the animal experiments. The study was not designed to evaluate sex differences, and sex-specific analyses were therefore not performed.

Field-collected samples	Animals were housed in temperature (21 ± 2 ºC) and humidity (40 %) controlled rooms with a 12 h light/12 h dark cycle. Teklad Global Rodent diet (Harlan Teklad, USA) and untreated tap water were offered ad libitum.
Ethics oversight	The protocol and animal care were in accordance with the Finnish and European legislation and were approved by the national Project Authorization Board (license numbers ESAVI/41363/2019 and ESAVI/2362/04.10.07/2017). Experiments involving mice are performed under permission from the National Animal Experiment Board following the regulations of the EU and national legislation (EU Directive 2010/63/EU, the European Convention ETS123).

Note that full information on the approval of the study protocol must also be provided in the manuscript.

Clinical data

Policy information about clinical studies

All manuscripts should comply with the ICMJE guidelines for publication of clinical research and a completed CONSORT checklist must be included with all submissions.

Clinical trial registration	EudraCT number: 2021-000625-27
Study protocol	The study protocol is available from the corresponding author upon reasonable request. Key protocol details are provided in the Methods section and Supplementary Information. In addition, the full study protocol was submitted to Nature Communications as part of the original manuscript submission.
Data collection	Data were collected at Oulu University Hospital, Finland. First posted date on the registry: 21.05.2021, Start of recruitment: 01.11.2021, First subject first visit: 29.11.2023, Last subject last visit: 10.10. 2023, End of study: 31.12.2023
Outcomes	Predefined outcome measures included circulating brain-derived protein biomarkers, T2 dark-fluid (T2DF) MRI signal intensity changes, and physiological brain pulsation measures. Additional analyses were considered exploratory and are described in the manuscript and Supplementary Information.

Dual use research of concern

Policy information about dual use research of concern

Hazards

Could the accidental, deliberate or reckless misuse of agents or technologies generated in the work, or the application of information presented in the manuscript, pose a threat to:

No	Yes	
☐	☐	Public health
☐	☐	National security
☐	☐	Crops and/or livestock
☐	☐	Ecosystems
☐	☐	Any other significant area

Experiments of concern

Does the work involve any of these experiments of concern:

No	Yes	
☐	☐	Demonstrate how to render a vaccine ineffective
☐	☐	Confer resistance to therapeutically useful antibiotics or antiviral agents
☐	☐	Enhance the virulence of a pathogen or render a nonpathogen virulent
☐	☐	Increase transmissibility of a pathogen
☐	☐	Alter the host range of a pathogen
☐	☐	Enable evasion of diagnostic/detection modalities
☐	☐	Enable the weaponization of a biological agent or toxin
☐	☐	Any other potentially harmful combination of experiments and agents

Plants

Seed stocks

Report on the source of all seed stocks or other plant material used. If applicable, state the seed stock centre and catalogue number. If plant specimens were collected from the field, describe the collection location, date and sampling procedures.

Novel plant genotypes

Describe the methods by which all novel plant genotypes were produced. This includes those generated by transgenic approaches, gene editing, chemical/radiation-based mutagenesis and hybridization. For transgenic lines, describe the transformation method, the number of independent lines analyzed and the generation upon which experiments were performed. For gene-edited lines, describe the editor used, the endogenous sequence targeted for editing, the targeting guide RNA sequence (if applicable) and how the editor was applied.

Authentication

Describe any authentication procedures for each seed stock used or novel genotype generated. Describe any experiments used to assess the effect of a mutation and, where applicable, how potential secondary effects (e.g. second site T-DNA insertions, mosiacism, off-target gene editing) were examined.

ChIP-seq

Data deposition

☐ Confirm that both raw and final processed data have been deposited in a public database such as GEO.

☐ Confirm that you have deposited or provided access to graph files (e.g. BED files) for the called peaks.

Data access links
May remain private before publication.

For "Initial submission" or "Revised version" documents, provide reviewer access links. For your "Final submission" document, provide a link to the deposited data.

Files in database submission

Provide a list of all files available in the database submission.

Genome browser session
(e.g. UCSC)

Provide a link to an anonymized genome browser session for "Initial submission" and "Revised version" documents only, to enable peer review. Write "no longer applicable" for "Final submission" documents.

Methodology

Replicates

Describe the experimental replicates, specifying number, type and replicate agreement.

Sequencing depth

Describe the sequencing depth for each experiment, providing the total number of reads, uniquely mapped reads, length of reads and whether they were paired- or single-end.

Antibodies

Describe the antibodies used for the ChIP-seq experiments; as applicable, provide supplier name, catalog number, clone name, and lot number.

Peak calling parameters

Specify the command line program and parameters used for read mapping and peak calling, including the ChIP, control and index files used.

Data quality

Describe the methods used to ensure data quality in full detail, including how many peaks are at FDR 5% and above 5-fold enrichment.

Software

Describe the software used to collect and analyze the ChIP-seq data. For custom code that has been deposited into a community repository, provide accession details.

Flow Cytometry

Plots

Confirm that:

☐ The axis labels state the marker and fluorochrome used (e.g. CD4-FITC).

☐ The axis scales are clearly visible. Include numbers along axes only for bottom left plot of group (a 'group' is an analysis of identical markers).

☐ All plots are contour plots with outliers or pseudocolor plots.

☐ A numerical value for number of cells or percentage (with statistics) is provided.

Methodology

Sample preparation

Describe the sample preparation, detailing the biological source of the cells and any tissue processing steps used.

Instrument

Identify the instrument used for data collection, specifying make and model number.

Software

Describe the software used to collect and analyze the flow cytometry data. For custom code that has been deposited into a community repository, provide accession details.

Cell population abundance | *Describe the abundance of the relevant cell populations within post-sort fractions, providing details on the purity of the samples and how it was determined.*

Gating strategy | *Describe the gating strategy used for all relevant experiments, specifying the preliminary FSC/SSC gates of the starting cell population, indicating where boundaries between "positive" and "negative" staining cell populations are defined.*

☐ Tick this box to confirm that a figure exemplifying the gating strategy is provided in the Supplementary Information.

Magnetic resonance imaging

Experimental design

Design type	Randomized crossover intervention study combined with exploratory NO-only and sham-control sub-studies. Resting-state physiological and MRI measurements were acquired before and after intervention.
Design specifications	Participants underwent pre- and post-intervention MRI acquisitions. The primary randomized crossover study included NO+WBVp and WBVp interventions separated by a washout period of at least 7 days. Additional NO-only and sham-control cohorts were analyzed separately.
Behavioral performance measures	N/A

Acquisition

Imaging type(s)	Structural MRI T1, T2 and T2 dark-fluid (T2DF) MRI, and functional MRI magnetic resonance encephalography (MREG).
Field strength	3 tesla
Sequence & imaging parameters	MRI acquisition parameters for T2DF MRI, MREG, T1-weighted, and T2-weighted sequences are reported in the Methods section (MRI acquisition).
Area of acquisition	whole brain

Diffusion MRI ☐ Used ☒ Not used

Preprocessing

Preprocessing software	Image preprocessing was performed using FSL (FMRIB Software Library), MATLAB, and custom processing pipelines as described in the Methods section.
Normalization	Not performed.
Normalization template	Not performed.
Noise and artifact removal	Standard preprocessing included bias-field correction.
Volume censoring	Not performed.

Statistical modeling & inference

Model type and settings	Voxelwise paired comparisons and ROI-based analyses were performed to assess intervention-related changes between pre- and post-intervention measurements. Correlation analyses were conducted using Pearson's correlation coefficient where appropriate. Statistical significance was accepted at the .05 level (two-tailed).
Effect(s) tested	Intervention-related changes in T2DF signal intensity and physiological fMRI measures.

Specify type of analysis: ☐ Whole brain ☐ ROI-based ☒ Both

Anatomical location(s) | Whole-brain statistical analyses were conducted following spatial normalization to MNI space. Anatomical localization was based on subject-specific anatomical MRI images. ROI analyses combined automated threshold-based segmentation using FSL with anatomically guided delineation based on corresponding T1-weighted and T2-weighted images. For manually defined regions, ROIs were identified using a diagnostic DICOM viewer with simultaneous reference to T2DF, T1-weighted, and T2-weighted images to ensure accurate separation of venous, dural, CSF, and brain tissue compartments.

Statistic type for inference (See Eklund et al. 2016)	Whole-brain analyses used voxelwise randomise statistical testing with FWER correction. ROI analyses used paired t-tests, Wilcoxon signed-rank tests, and Pearson correlation analyses as appropriate with FDR correction.
Correction	FWER, FDR

Models & analysis

n/a	Involved in the study
☒	☐ Functional and/or effective connectivity
☒	☐ Graph analysis
☒	☐ Multivariate modeling or predictive analysis
