# Supplementary material for: Vasomechanic treatment is associated with increased efflux of human brain proteins
Source: Nat Commun. 2026 Nov 3;17:11164. doi: 10.1038/s41467-026-78894-y (PMC13731658; doi:10.1038/s41467-026-78894-y)
Supplement: Supplementary file 2 [file 41467_2026_78894_MOESM2_ESM.pdf]

Supplementary information for

Vasomechanic treatment is associated with increased efflux of human brain proteins

Johanna Tuunanen[1,2]*, Katariina Hautamäki[1,2], Tommi Väyrynen[1,2], Matti Järvelä[1,2], Vesa Korhonen[1,2], Niko Huotari[1,2], Mika Kaakinen[3], Janne Kananen[1,4], Heta Helakari[1,2], Lauri Raitamaa[1,2], Jari Jukkola[3,5,6], Sanna-Kaisa Herukka[7,8], Katariina Laurén[1,2], Ulla Salmi[1,2], Lauri Eklund[3], Juha Nikkinen[9,10], Maiken Nedergaard[11,12], Vesa Kiviniemi[1,2,3]*

1. Oulu Functional Neuroimaging, Research unit of Health Sciences and Technology, Faculty of Medicine, University of Oulu, Oulu, Finland
2. Radiology, Department of Diagnostics, Medical Research Center, Oulu University Hospital, Oulu, Finland
3. Oulu Center for Cell-Matrix Research, Faculty of Biochemistry and Molecular Medicine, Biocenter Oulu, University of Oulu, Oulu, Finland.
4. Clinical Neurophysiology, Department of Diagnostics, Oulu University Hospital, Oulu, Finland
5. Department of Experimental Medical Science, Lund University, Lund, Sweden
6. Wallenberg Centre for Molecular Medicine, Lund University, Lund, Sweden
7. Institute of Clinical Medicine/Neurology, University of Eastern Finland, Kuopio, Finland.
8. Department of Neurology, Kuopio University Hospital, Kuopio, Finland.
9. Department of Oncology and Radiotherapy, Medical Research Center, Oulu University Hospital, Oulu, Finland
10. Research unit of Health Sciences and Technology, Faculty of Medicine, University of Oulu, Oulu, Finland
11. Center for Translational Neuromedicine, University of Rochester, Rochester, NY, USA
12. Center for Translational Neuromedicine, University of Copenhagen Faculty of Health and Medical Sciences, Copenhagen, Denmark

*Corresponding authors:
Johanna Tuunanen
johanna.tuunanen@oulu.fi
Vesa Kiviniemi
vesa.kiviniemi@oulu.fi

Supplementary information Text 1

Brain pulsation ranges from MREG

Cardiorespiratory frequencies identified from MREG closely correspond to simultaneously recorded physiological measurements[1–6]. Individual respiratory and cardiac ranges and peak frequencies were extracted from subject-specific MREG periodograms while avoiding harmonics and overlap with other physiological frequency bands. From the MREG signal frequency spectrum, respiratory and cardiac minima, maxima, and peak values were identified (Supplementary Fig. 26) and used to calculate individual cardiorespiratory frequency ranges. The lower and upper bounds of each frequency range were determined based on the points at which the spectral power deviated from the noise floor, while ensuring separation from adjacent physiological bands, their harmonics, and respiratory-induced heterodyne components surrounding the cardiac frequency range.

Fast Fourier Transform (FFT) from scanner's physiological respiration belt and photoplethysmogram signals were calculated using MATLAB. Frequencies identified from the MREG data were then double-checked

against these physiological signals solely for validation purposes, to ensure the accuracy of frequency identification (Supplementary Fig. 26).

Vasomechanic nitric oxide mediated vasodilation with Piezo1-targeted whole-body vibration (NO & WBV_p)

Compared to baseline, the vasomechanic NO+WBV_p intervention increased the spectral respiration range (Pre: 0.19 ± 0.05 Hz, Post: 0.21 ± 0.05 Hz, $p = 0.04$, n = 20) but did not alter respiration rate (Pre and Post: 15 ± 4 b/min, $p = 0.97$, $n = 25$). Heart rate also decreased (Pre: 62 ± 6 b/min, Post: 58 ± 8 b/min, $p = 0.001$, $n = 25$), but the width of the cardiac range was not significantly changed (Pre: 0.25 ± 0.06 Hz, Post: 0.28 ± 0.05 Hz, $p = 0.051$, $n = 23$).

Nitric oxide (NO) mediated vasodilation

NO-mediated vasodilation reduced respiratory rate (15 ± 4 b/min to 13 ± 4 b/min, $p = 0.007$, $n = 26$) and increased heart rate (from 62 ± 6 b/min to 66 ± 9 b/min, $p = 9.6 \times 10^{-6}$, $n = 26$) compared to baseline. There were no significant effects on spectral respiration range (Pre: 0.19 ± 0.05 Hz, during NO 0.20 ± 0.06 Hz, $p = 0.363$, $n = 19$) or cardiac range (Pre: 0.25 ± 0.06 Hz, during NO 0.27 ± 0.07 Hz, $p = 0.164$, $n = 23$).

Piezo1-targeted whole-body vibration (WBV_p)

Compared to baseline, WBV_p interventions did not markedly alter the spectral respiration range (Pre: 0.20 ± 0.06 Hz, Post: 0.20 ± 0.05 Hz, $p = 0.43$, $n = 20$) and respiration rate remained stable (Pre 15 ± 4 vs. Post 15 ± 4 breaths/min, $p = 0.75$, $n = 27$). Heart rate decreased slightly (61 ± 7 → 59 ± 8 beats/min, $p = 0.04$, $n = 27$) and the spectral range of cardiac rate increased (Pre: 0.23 ± 0.04 Hz, Post: 0.25 ± 0.05 Hz, $p = 0.02$, $n = 25$) significantly during the WBV_p intervention.

Supplementary information Text 2

Hemodilution correction factor: To derive a study-specific hemodilution correction factor, the relative percentage changes in blood parameters (hemoglobin, hematocrit, erythrocytes, leukocytes, and thrombocytes) were calculated for each participant, and the mean percentage decrease across parameters was used as an estimate of blood dilution. This yielded an average reduction of 5.2%, which was applied as a global hemodilution correction factor.

The correction factor was applied uniformly to all analytes and was used only in the analyses presented in Figures 1d–e. Corresponding uncorrected values are provided in Supplementary Figure 3. Importantly, the main findings remained qualitatively unchanged, as increases in circulating proteins were also observed in the uncorrected analyses.

Supplementary information Text 3

Methods for mice study

Mouse study design

2-3 months old C56 Bl6/N (Charles River) female mice were used in all experiments. Animals were housed in temperature (21 ± 2 ºC) and humidity (40 %) controlled rooms with a 12 h light/12 h dark cycle. Teklad Global Rodent diet (Harlan Teklad, USA) and untreated tap water were offered ad libitum. Mice were anaesthetized with subcutaneous injection of ketamine (75 mg/kg) and xylazine (10 mg/kg) (KX), with supplementation of anaesthesia every 30 minutes during the entire experiment. Subcutaneous lidocaine (10 mg/kg) was administered to surgical sites 5 min before operations. The protocol and animal care were in accordance with the Finnish and European legislation and were approved by the national Project Authorization Board (license numbers ESAVI/41363/2019 and ESAVI/2362/04.10.07/2017). Experiments involving mice are performed under permission from the National Animal Experiment Board following the regulations of the EU and national legislation (EU Directive 2010/63/EU, the European Convention ETS123). The study was not designed to evaluate sex differences, and sex-specific analyses were therefore not performed.

Cisterna magna injections, combination and NO-mediated vasodilation with SNP treatment

The femoral vein and cisterna magna (CM) cannulation and tracer infusion[7]: The femoral vein was exposed through an incision on skin and cannulated with a BTPE 50 polyethylene tubing filled with physiological saline supplemented with 500 IU/ml heparin. Next, BTPE-10 tubing, connected to a 30G dental needle, was inserted in the cisterna magna (CM) and fixed in place with superglue and dental acrylic. 4 µl (25 mg/ml) of 40 kDa of fluorescein isothiocyanate-dextran (Sigma, FD40S-250MG), was infused into the subarachnoid space at a rate of 2µl/min, driven by a microinjector (Legato130, KD Scientific). **Combination treatment:** After the CM-cannula was sealed with a cauterizer to prevent backflow, animals were placed onto a vibrating mattress on a supine position. A heating lamp was placed above the animal to keep their body temperature ~37ºC. Sodium nitroprusside (SNP) (Sigma, 10 µg/kg/min) was infused into the femoral vein for 15 min at a rate of 10 µl/min with or without 40 Hz vibration. Euthanasia was performed with an overdose of KX, followed transcardial perfusion with ice-cold 1x PBS supplemented with 500 IU/ml heparin. The dura mater from the dorsal part of the skull was collected, flash frozen in liquid nitrogen, and stored at -80 ºC. **NO-mediated vasodilation treatment:** 40 kDa FITC-dextran (FD40) injection into the CM was followed by blood sampling from the femoral vein 5 min later, with an i.v. infusion of SNP or saline (Control) starting 2 min after the first blood sampling and continuing for 40 min, after which the final blood sampling was performed. SNP (Sigma, PHR1423-1G) (5 µg/kg/min) was infused into the femoral vein at the rate of 5 µl/min for 40 minutes. The control group received 0.9% NaCl (saline) i.v. at a similar rate. Blood was collected and centrifuged at 1100 x g for 10 min and supernatant (serum) stored at -70 °C.

Fluorescence measurements from serum and tissue

The tissue samples and serum were thawed, weighed, and immersed in PBS and homogenized for 5 min at 50 Hz with a homogenizer (Qiagen, Tissue Lyzer LT) and stainless-steel beads. After centrifugation at 12,400 g for 1 hour at room temperature, the supernatant was collected and diluted in PBS. All diluted samples were assayed in triplicate using a 96-well plate. Serial dilutions of 40/4 kDa FITC dextran was used for making a standard curve. Fluorescence of FITC-dextran was measured with fluorometer ((PerkinElmer, VICTOR3V 1420 Multilabel counter) with excitation/emission at 485nm/535nm. Serum and dura mater samples without tracer were used as negative controls.

Supplementary Figures

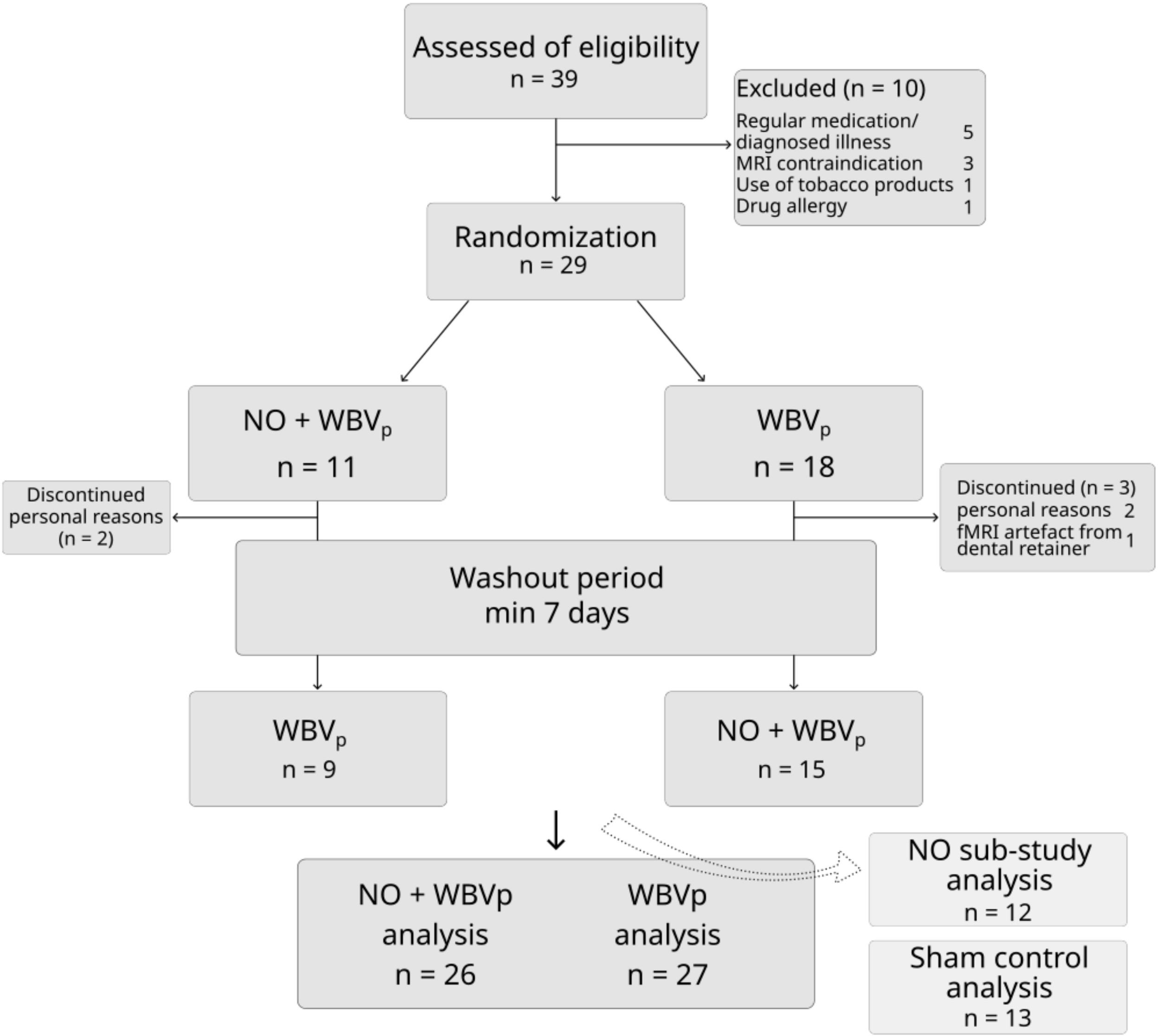

Supplementary Figure 1. CONSORT (Consolidated Standards of Reporting Trials) flow diagram of randomized crossover design and exclusion of subjects. Twenty-nine participants were included in the study, of whom 26 completed the combined NO-mediated vasodilation with whole-body vibration (NO+WBV_p) intervention and 27 completed the whole-body vibration (WBV_p) intervention in randomized order on separate study visits. In addition, a subset of participants (*n* = 12) took part in an exploratory NO-only (NO) sub-study on a separate experimental day. A separate sham control cohort (*n* = 13) was included to assess the effects of prolonged supine positioning and repeated MRI acquisition without active intervention.

Vasomechanic nitric oxide-mediated vasodilation with Piezo1-targeted whole-body vibration (NO & WBV_p)

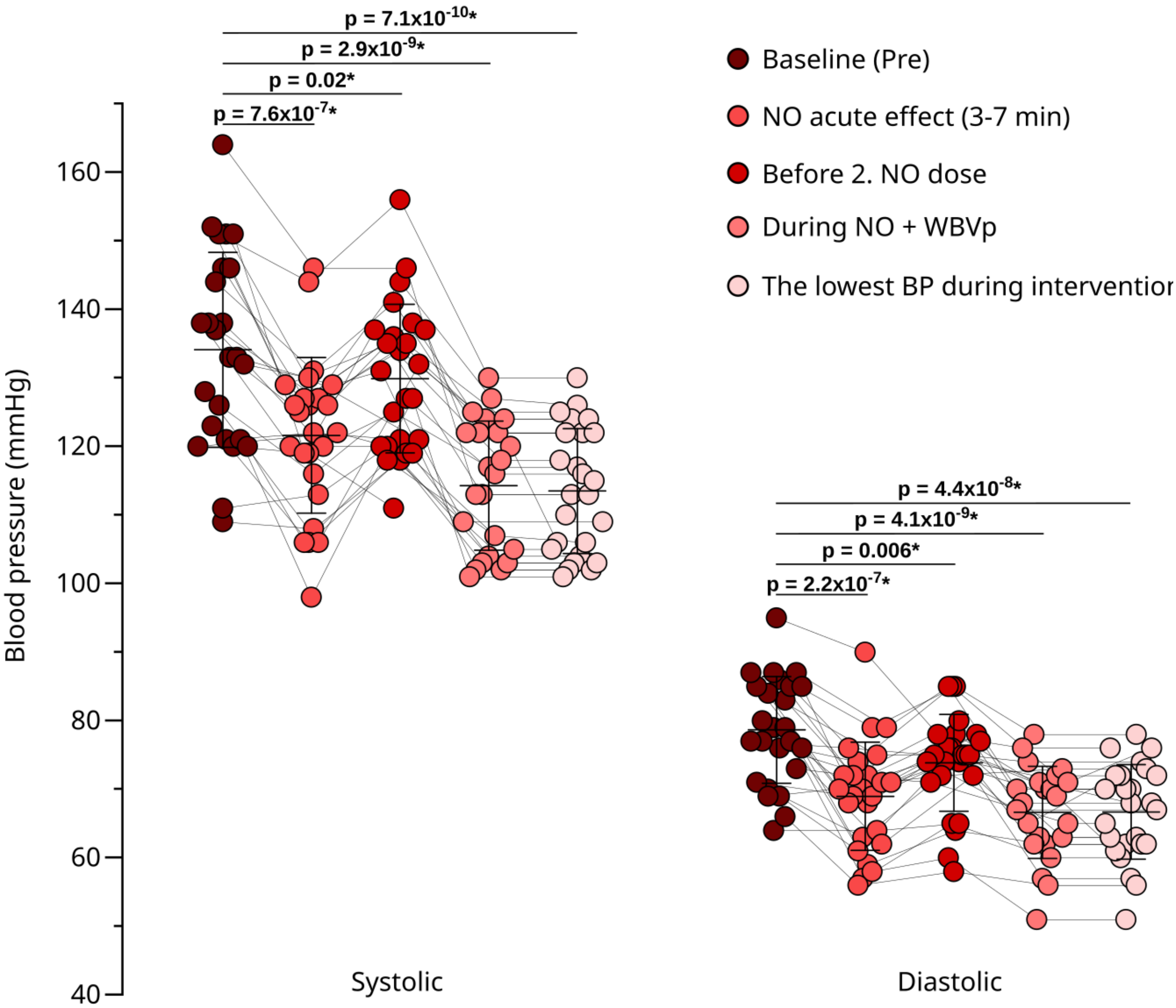

Supplementary Figure 2. Measured blood pressure (systolic, diastolic) during NO+WBVp session. Mean (± standard deviation), paired two-tailed *t*-test, *n* = 25. NO = nitric oxide. Source data are provided as a Source Data file.

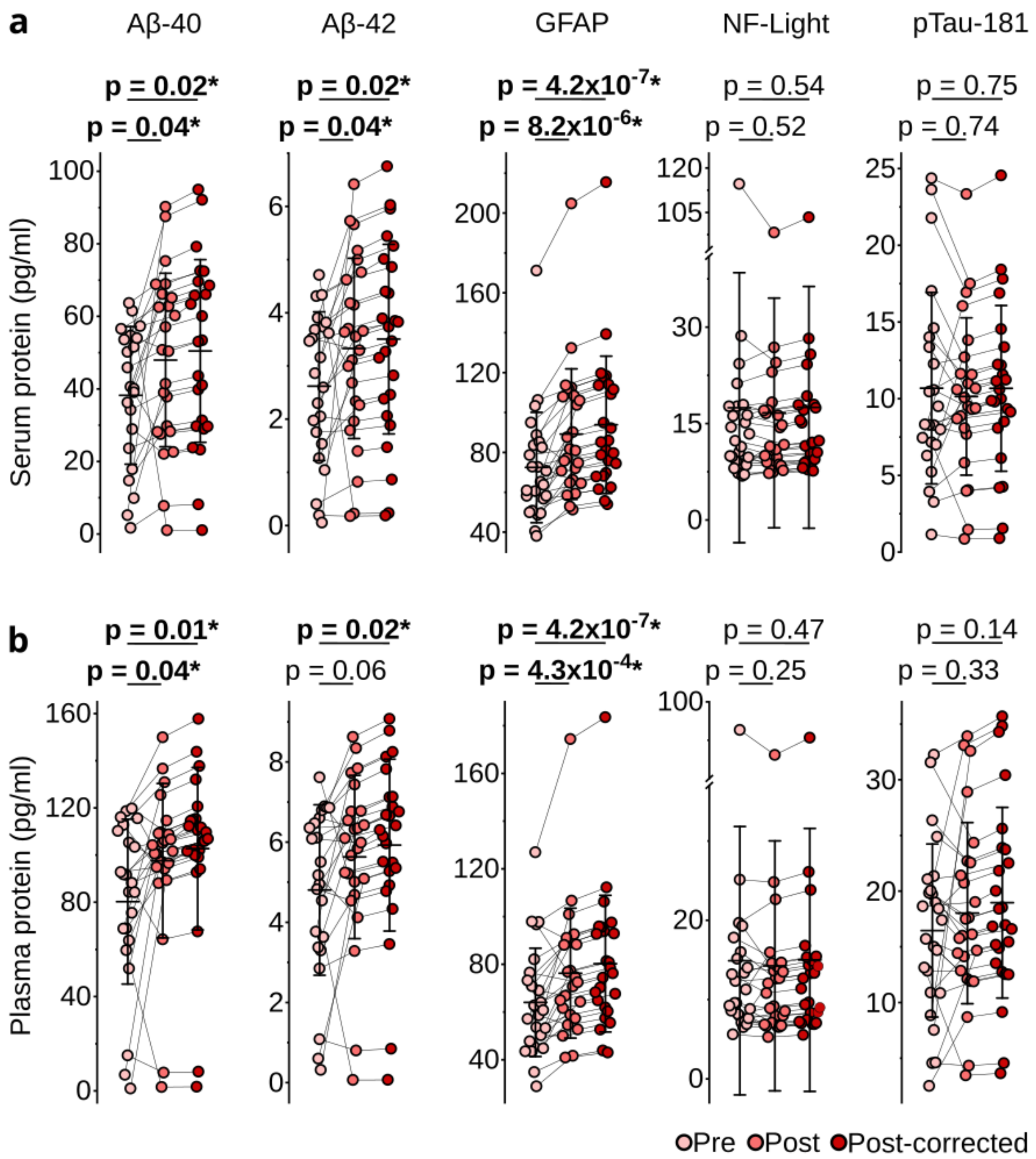

Supplementary Figure 3. Effects of NO+WBVp intervention on serum and plasma brain-derived protein levels. a. Serum ($n_{A\beta40}$ = 22, $n_{A\beta42}$ = 23, n_{GFAP} = 25, $n_{NF\text{-}Light}$ = 25, $n_{pTau181}$ = 23) and b. plasma ($n_{A\beta40}$ = 23, $n_{A\beta42}$ = 23, n_{GFAP} = 25, $n_{NF\text{-}Light}$ = 25, $n_{pTau181}$ = 25) concentrations of brain-derived proteins before (Pre) and after (Post) NO+WBV$_p$ intervention, with post-intervention values additionally shown after hemodilution correction (Post-corrected). Mean (± standard deviation), paired two-tailed *t*-test. Aβ40 & Aβ42 = beta-amyloid 40 & 42, GFAP = glial fibrillary acidic protein#, NF-Light = neurofilament-light#, pTau-181 = phosphorylated tau-181. #Paired-sample Wilcoxon signed-rank test was used for comparison. Source data are provided as a Source Data file.

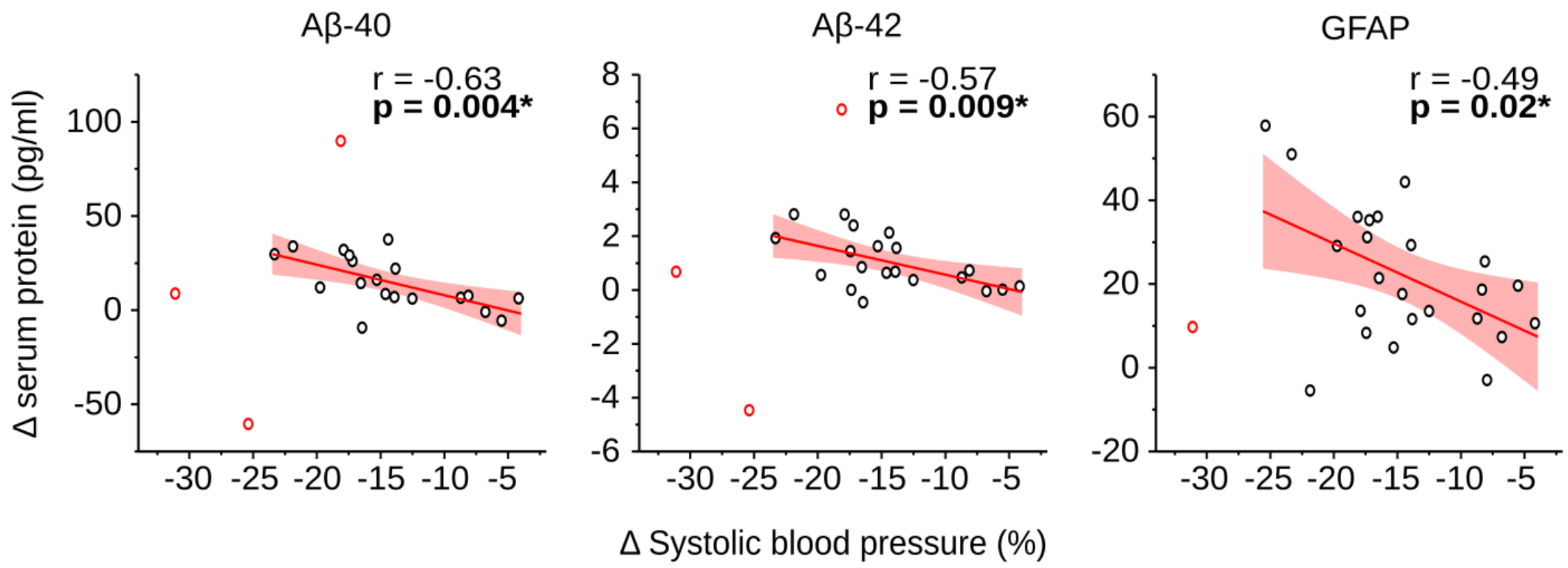

Supplementary Figure 4. Correlations between changes in circulating brain-derived proteins and the maximal extent of blood pressure reduction during the NO+WBV$_p$ intervention. Changes (Δ, post - pre) in serum beta-amyloid 40 & 42 (Aβ40, Aβ42) and glial fibrillary acidic protein (GFAP) concentrations correlate with the individual maximal blood pressure decrease (Pearson's correlation, linear regression line with 95% confidence intervals, $n_{A\beta40/A\beta42/GFAP}$ = 19/20/24). This figure replicates the analysis shown in Fig. 1e, with the addition of values masked from the final analysis (shown in red circles). Source data are provided as a Source Data file (Source Data Figure 1e).

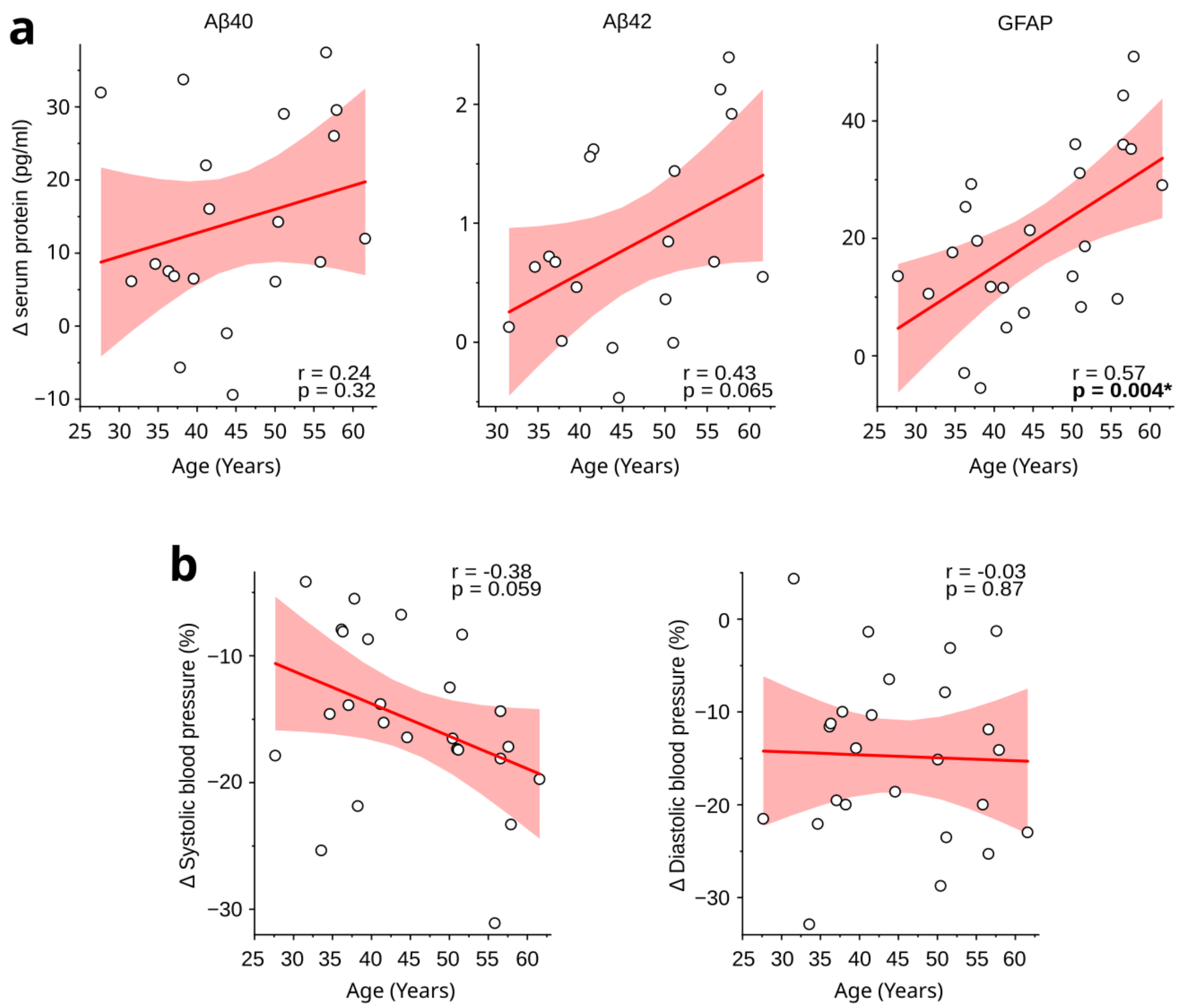

Supplementary Figure 5. Age-dependent changes in blood biomarkers and blood pressure following NO+WBVp intervention. a. Changes (Δ, post - pre) in serum beta-amyloid (Aβ40, Aβ42) and glial fibrillary acidic protein (GFAP) as a function of age (Pearson's correlation, linear regression line with 95% confidence intervals (CI), $n_{A\beta40/A\beta42/GFAP}$ = 20/19/24) in NO+WBV$_p$ intervention. b. Changes in maximal reduction of systolic and diastolic blood pressure during NO+WBV$_p$ intervention as functions of age (Pearson's correlation, linear regression line with 95% CI, $n_{SYS/DIAS}$ = 25/25). Source data are provided as a Source Data file.

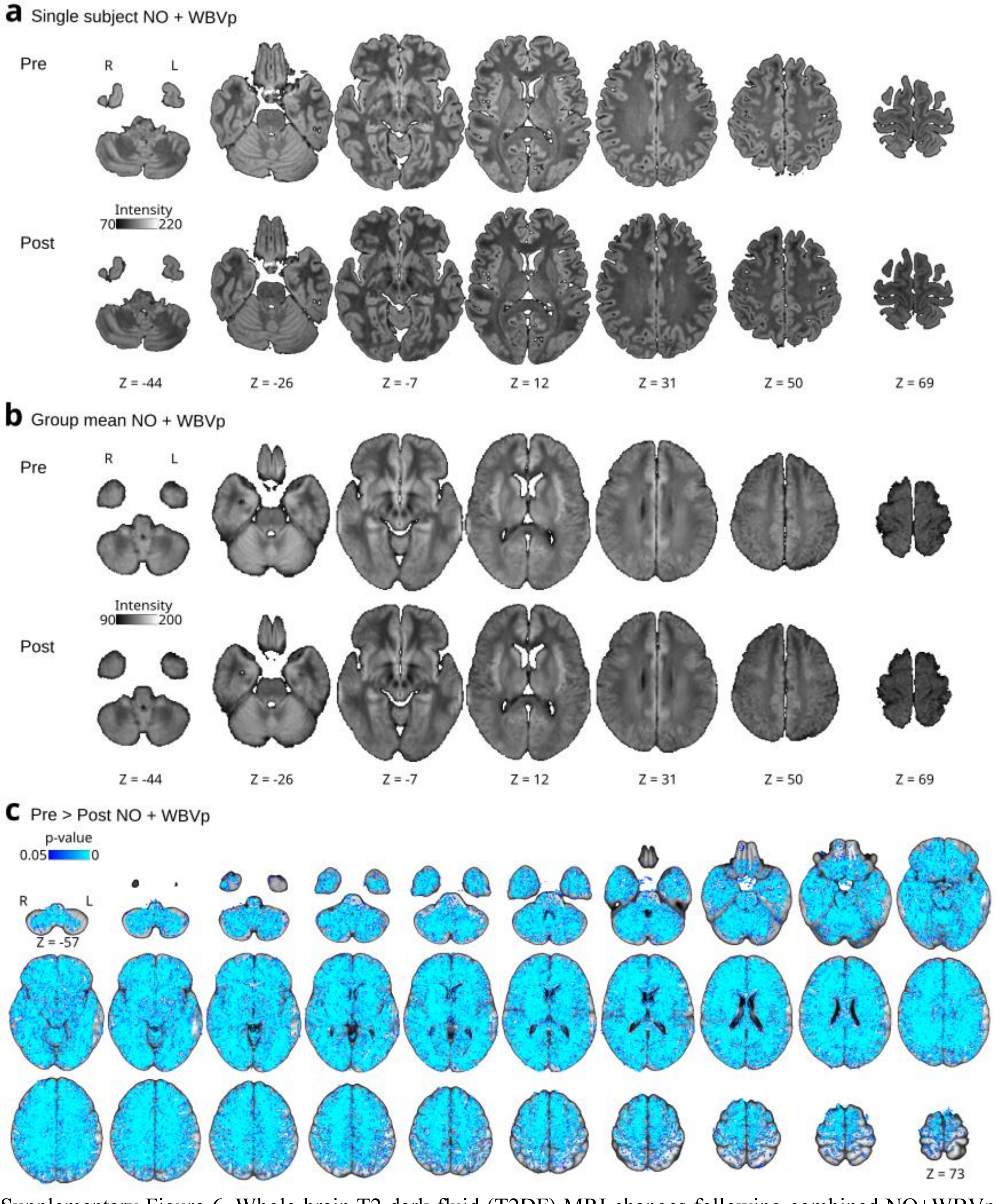

Supplementary Figure 6. Whole-brain T2 dark-fluid (T2DF) MRI changes following combined NO+WBVp intervention. Axial slices of T2DF image intensities in a. single representative subject, and b. group mean average (n = 24) T2DF images before (Pre) and after (Post) the combined vasomechanic NO+WBV$_p$ intervention. c. Statistical analysis result maps of the Post vs. Pre T2DF intensity maps (paired two-tailed t-test, family-wise-error-rate correction (FWER), $p < 0.05$, n = 24) indicating a significant whole-brain level signal change from the NO+WBV$_p$ intervention. R = right, L = left, Z = axial plane MNI152 coordinate.

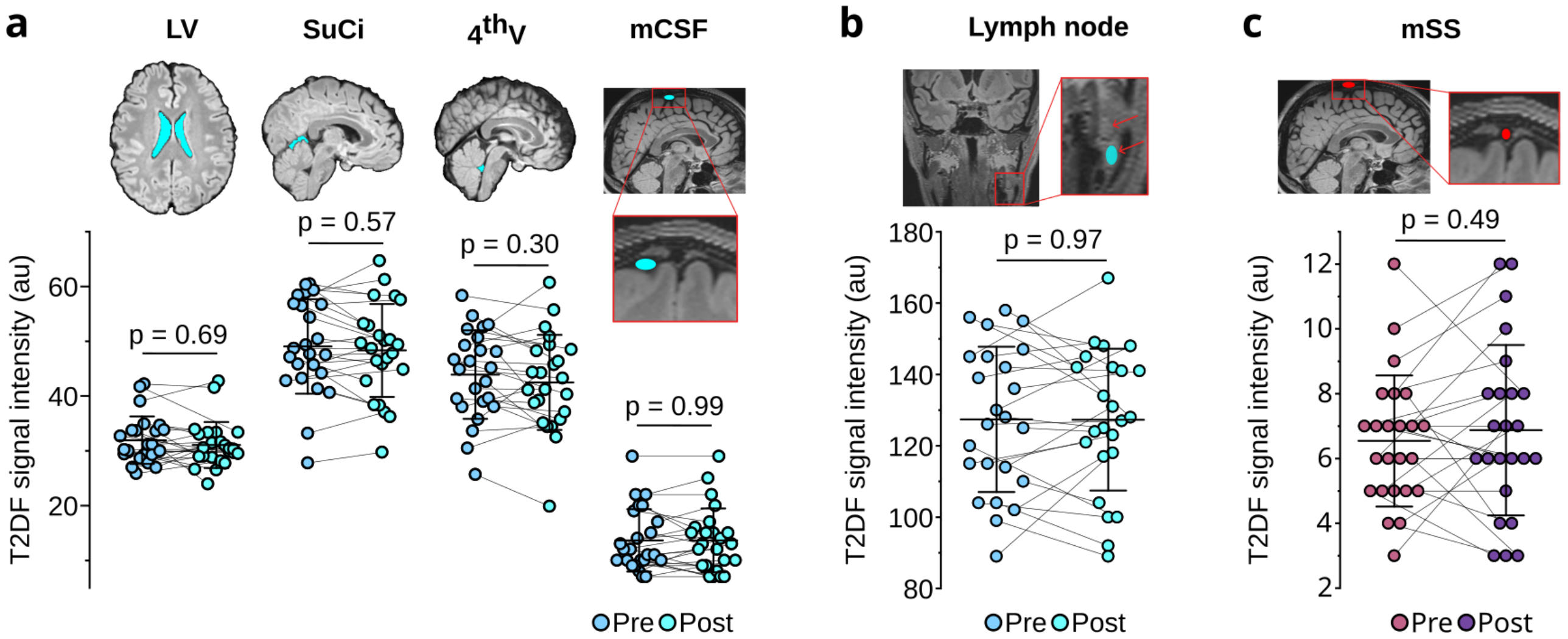

Supplementary Figure 7. ROI analyses of T2DF signal intensity in CSF spaces, cervical lymph nodes, and superior sagittal sinus following combined NO+WBVp intervention. Analyses of T2 dark-fluid (T2DF) images of a. lateral ventricles (LV#), superior cisterna (SuCi), 4th ventricle (4thV), middle parasagittal CSF space (mCSF#), b. cervical lymph node and c. middle/vertex superior sagittal sinus (mSS) before (Pre) and after (Post) NO+WBV$_p$ intervention showed no significant changes. Mean (± standard deviation), paired two-tailed *t*-test, *n* = 24. #Paired-sample Wilcoxon signed-rank test was used for comparison. au = arbitrary units. Source data are provided as a Source Data file.

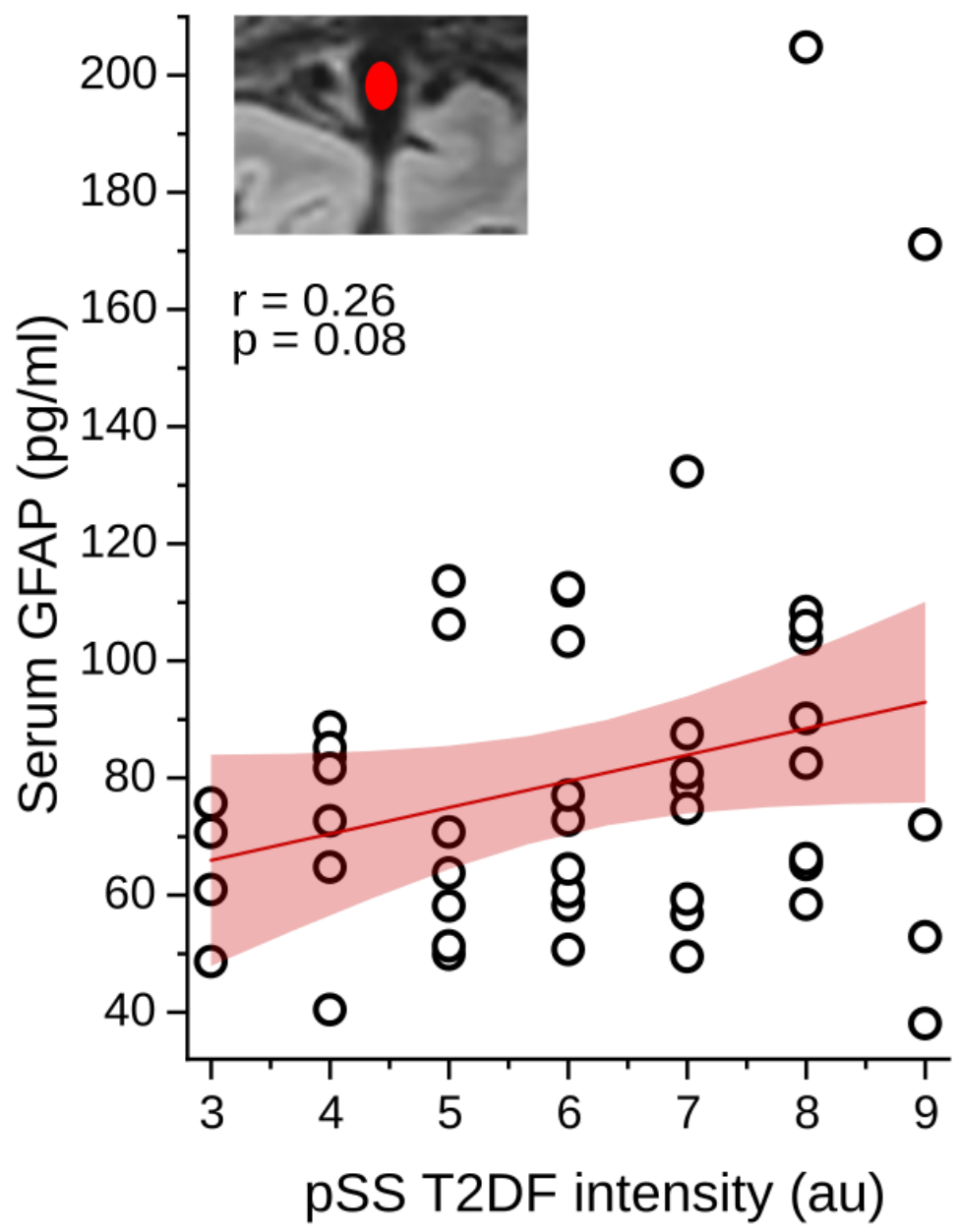

Supplementary Figure 8. Association between serum GFAP concentration and posterior superior sagittal sinus T2DF signal intensity. Measured serum glial fibrillary acidic protein (GFAP, *n* = 48) before and after the NO+WBV$_p$ intervention showed a trend toward a linear correlation with T2-weighted dark-fluid (T2DF) signal intensity in the posterior superior sagittal sinus (pSS) (Pearson's correlation, *p* = 0.08, linear regression line with 95% confidence intervals). au = arbitrary units. Source data are provided as a Source Data file.

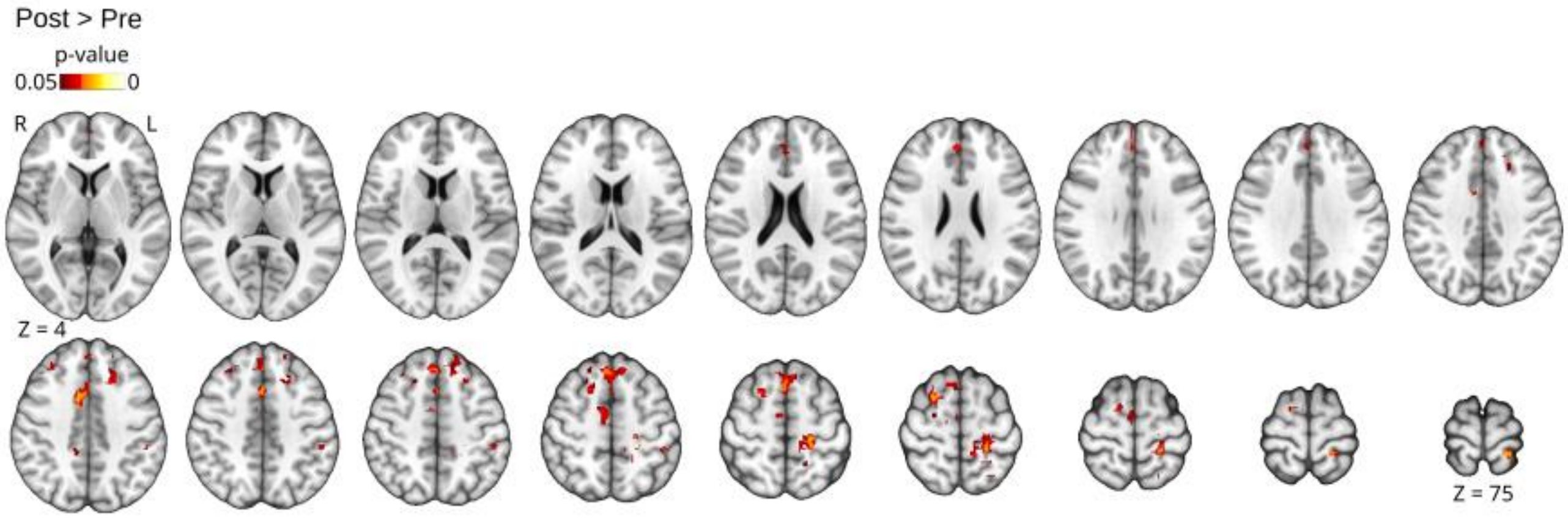

Supplementary Figure 9. Spatial distribution of the cardiac brain pulsation power differences following the combined intervention. Statistical maps of NO+WBV_p (n = 23) comparing Pre and Post intervention measurements (paired two-tailed t-test, family-wise-error-rate correction (FWER), $p < 0.05$). R = right, L = left, Z = axial plane MNI152 coordinate.

Nitric oxide (NO) mediated vasodilation

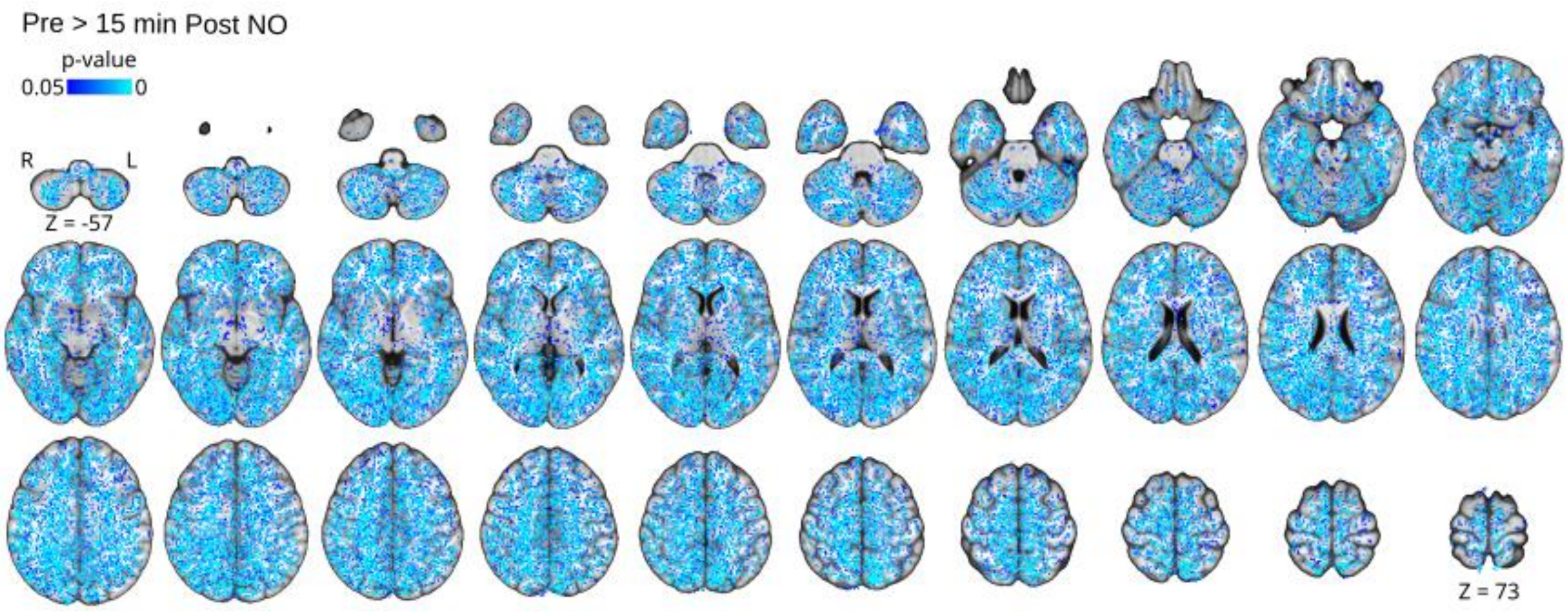

Supplementary Figure 10. Spatial distribution of T2 dark-fluid signal intensity differences following NO intervention. Statistical maps of NO ($n = 11$) comparing Pre and 15 min Post-NO administration measurements (paired two-tailed t-test, family-wise-error-rate correction (FWER), $p < 0.05$). R = right, L = left, Z = axial plane MNI152 coordinate.

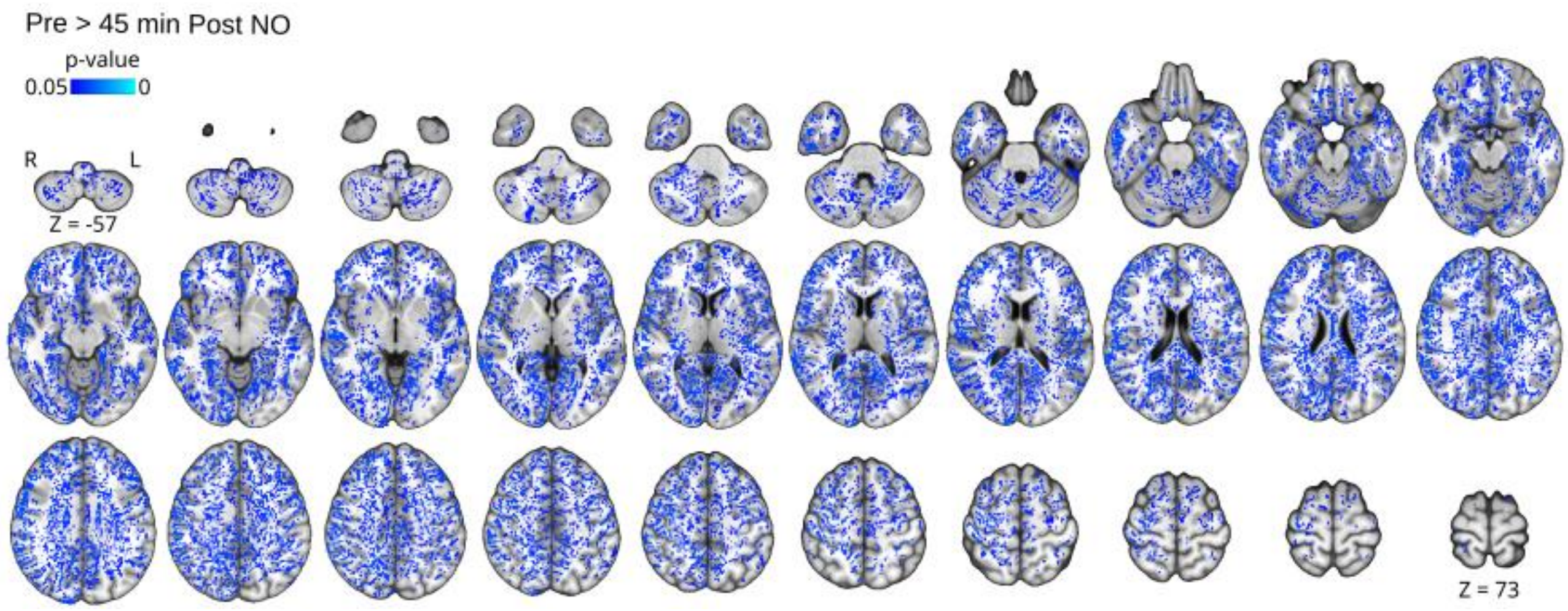

Supplementary Figure 11. Spatial distribution of T2 dark-fluid signal intensity differences following NO intervention. Statistical maps of NO ($n = 12$) comparing Pre and 45 min Post-NO administration measurements (paired two-tailed t-test, family-wise-error-rate correction (FWER), $p < 0.05$). R = right, L = left, Z = axial plane MNI152 coordinate.

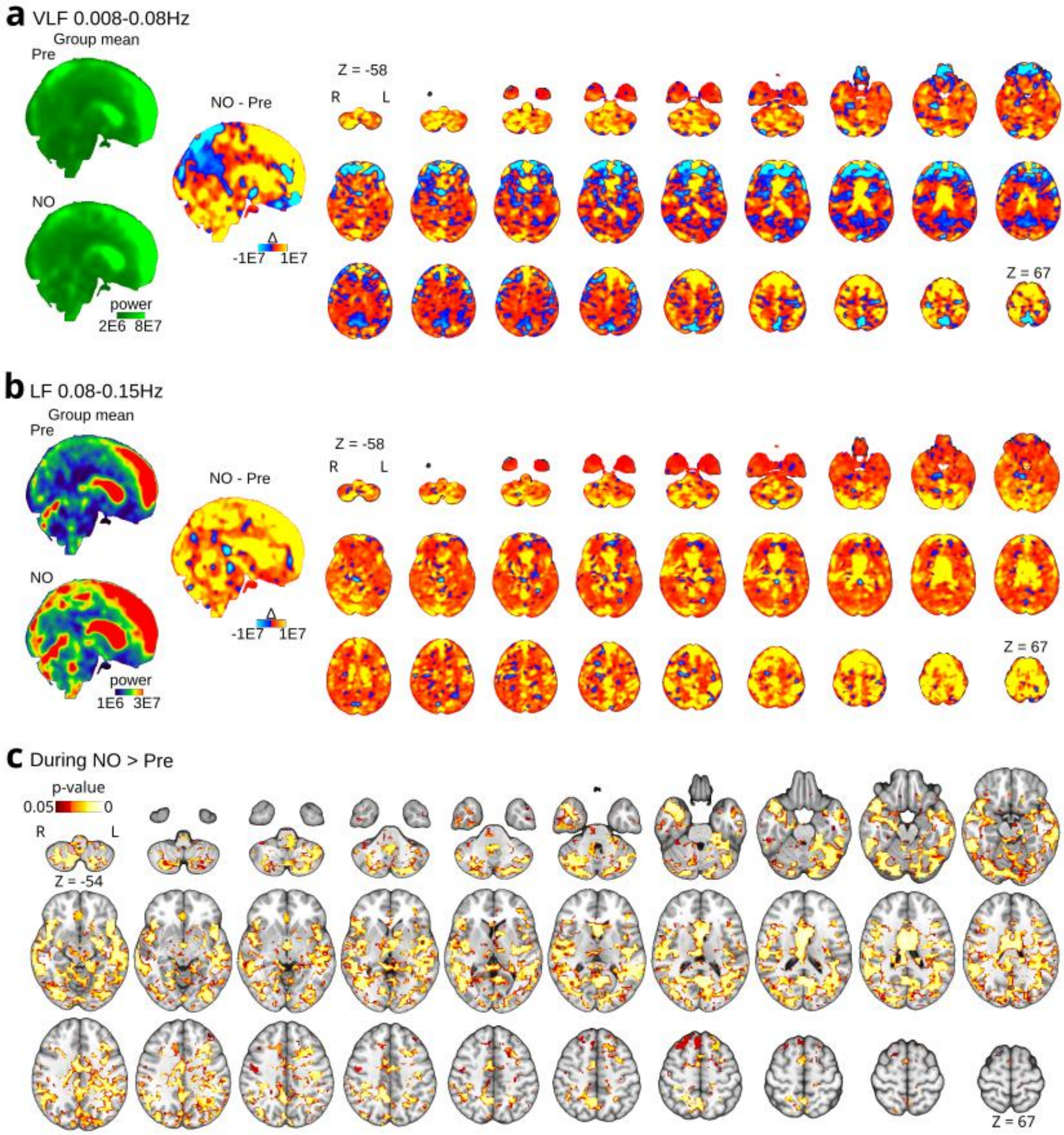

Supplementary Figure 12. MREG very-low and low frequency brain pulsations during NO-mediated vasodilation. a. Group mean and subtraction (during NO - Pre) maps of MREG very-low frequency (VLF, 0.008 – 0.08 Hz) brain pulsations for the NO phase of the combined NO+WBV_p intervention visit (*n* = 21) shown for Pre- and NO conditions. b. Group mean and subtraction (during NO - Pre) maps of MREG low frequency (LF, 0.08 – 0.15 Hz) brain pulsations for the NO phase of the combined NO+WBV_p intervention visit (*n* = 19) shown for Pre- and NO conditions. c. Spatial distribution of the low frequency (LF, 0.08-0.15 Hz) brain pulsation power difference maps (paired two-tailed *t*-test, family-wise-error-rate correction (FWER), $p < 0.05$) for the NO phase of the combined NO+WBV_p intervention visit (*n* = 19) between Pre and during NO conditions. R = right, L = left, Z = axial plane MNI152 coordinate.

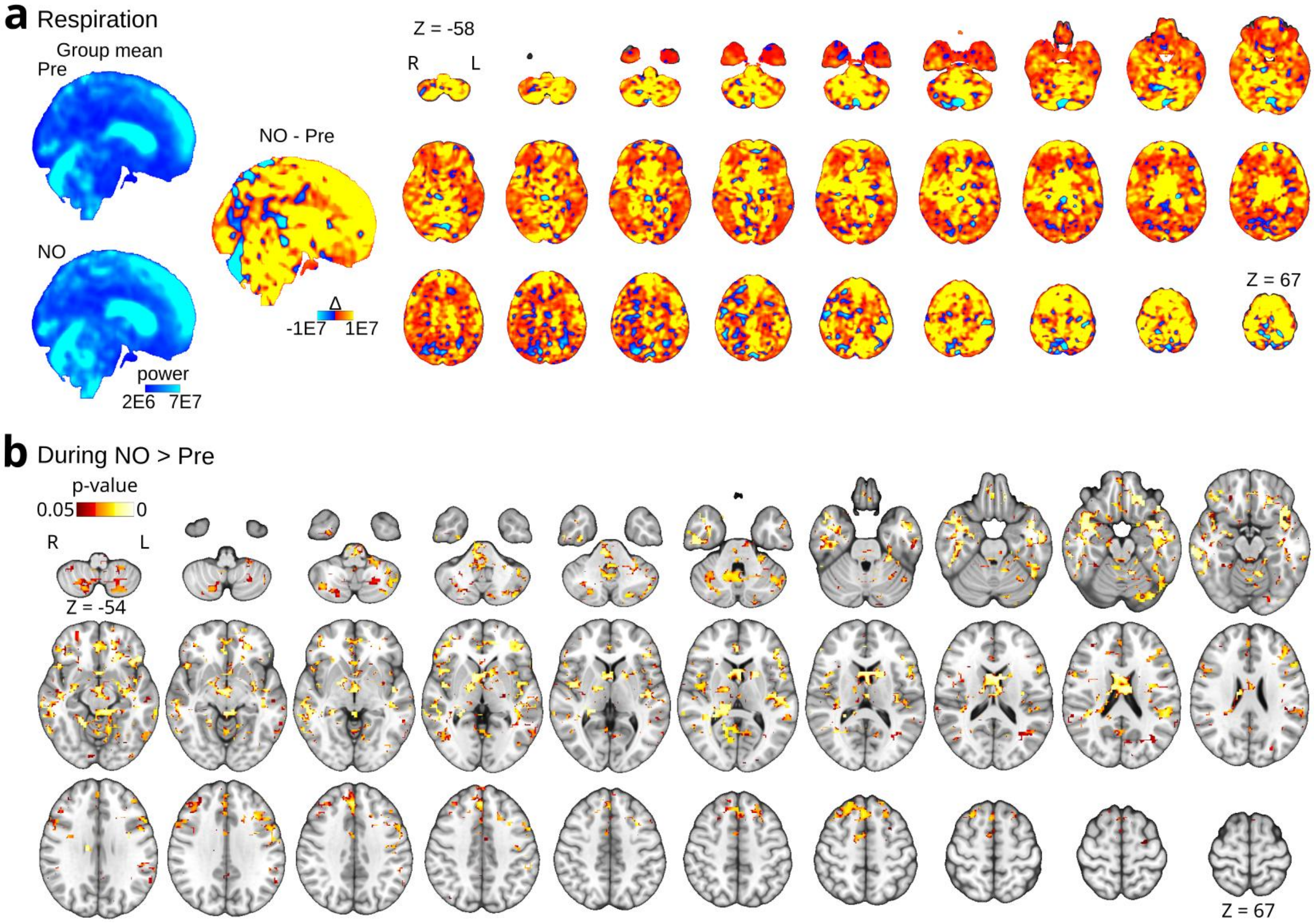

Supplementary Figure 13. Respiratory brain pulsation power changes during the NO-mediated vasodilation. a. Group mean and subtraction (during NO - Pre) maps of MREG individual respiratory frequency brain pulsations for the NO phase of the combined NO+WBV_p intervention visit (n = 19) shown for Pre- and NO conditions. b. Spatial distribution of the respiratory pulsation brain power difference maps (paired two-tailed t-test, family-wise-error-rate correction (FWER), $p < 0.05$) of the NO phase of the combined NO+WBV_p intervention visit (n = 19) between Pre and during NO conditions. R = right, L = left, Z = axial plane MNI152 coordinate.

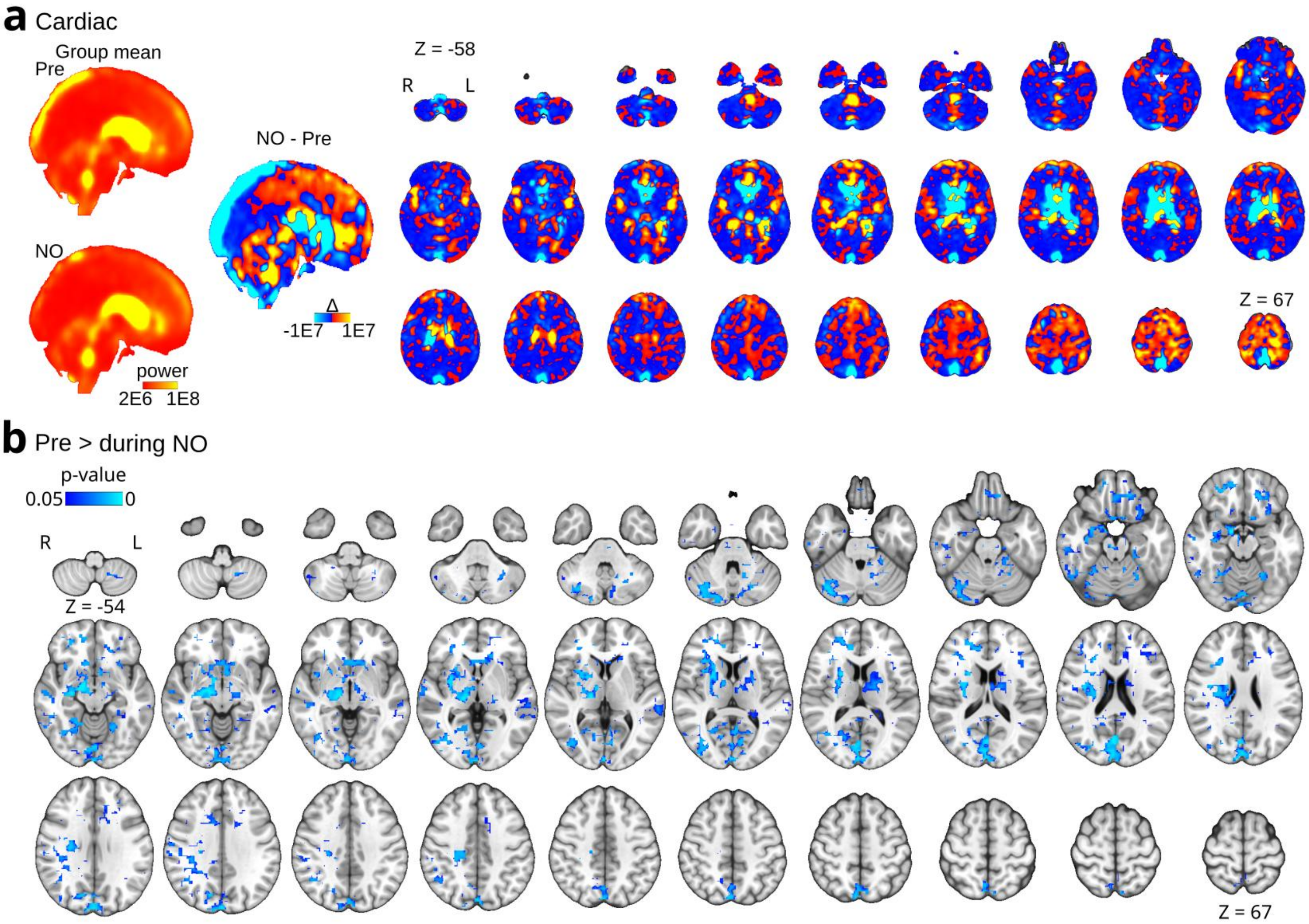

Supplementary Figure 14. Cardiac brain pulsation power changes during the NO-mediated vasodilation. a. Group mean and subtraction (during NO - Pre) maps of MREG individual cardiac frequency brain pulsations for the NO phase of the combined NO+WBV$_p$ intervention visit (n = 23) shown for Pre and NO conditions. b. Spatial distribution of the cardiac pulsation brain power difference maps (paired two-tailed t-test, family-wise-error-rate correction (FWER), $p < 0.05$) of the NO phase of the combined NO+WBV$_p$ intervention visit (n = 23) between Pre and during the NO condition. R = right, L = left, Z = axial plane MNI152 coordinate.

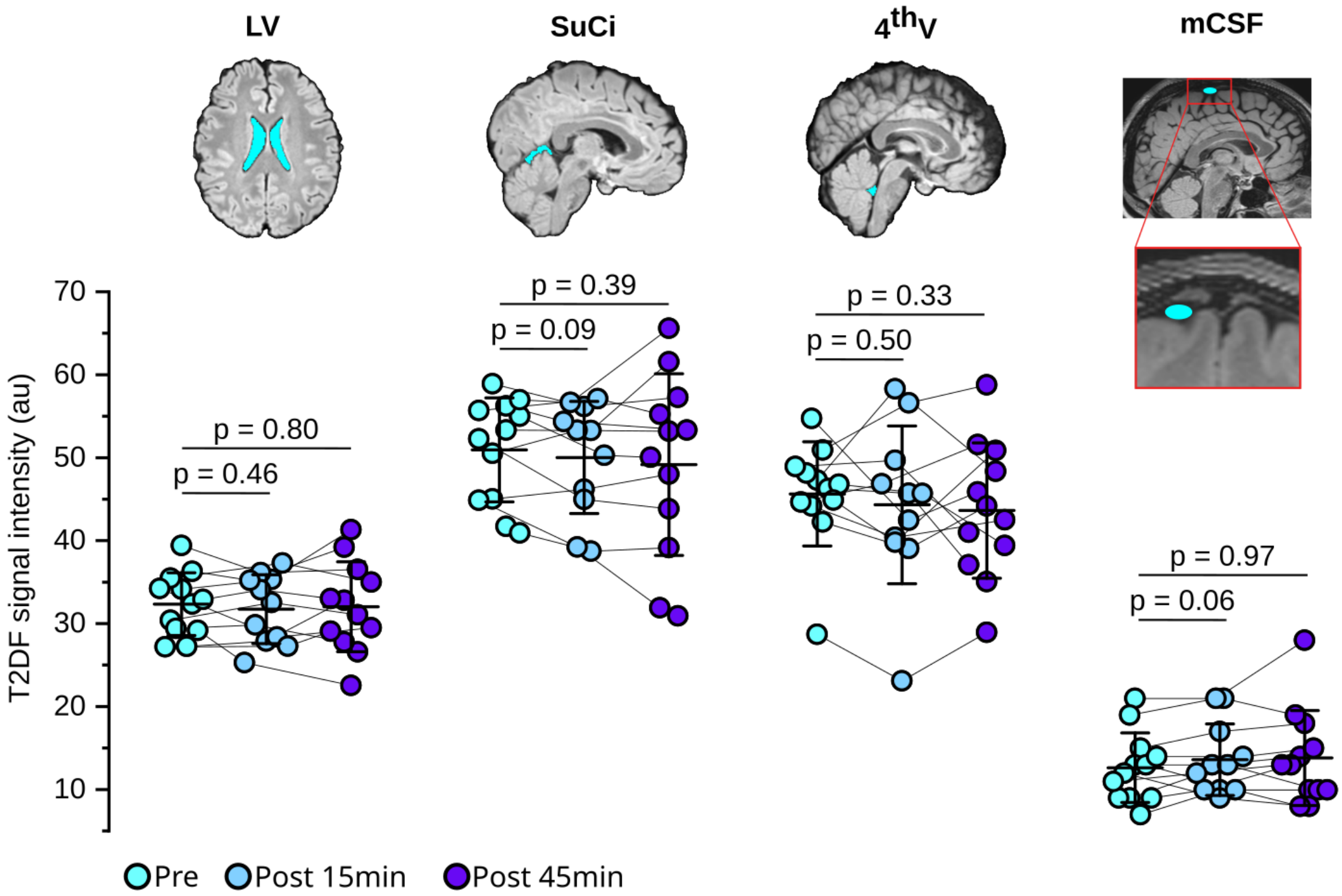

Supplementary Figure 15. ROI analysis of T2DF signal intensity in CSF spaces following NO administration. Analysis of T2 dark-fluid (T2DF) images of lateral ventricles (LV), superior cisterna (SuCi), 4th ventricle (4thV#), and middle parasagittal CSF space (mCSF) before (Pre, $n = 12$) and after NO administration at 15 min (Post 15 min, $n = 11$) and 45 min (Post 45 min, $n = 12$). Mean (± standard deviation), paired two-tailed *t*-test. #Paired Wilcoxon signed-rank test. au = arbitrary units. Source data are provided as a Source Data file.

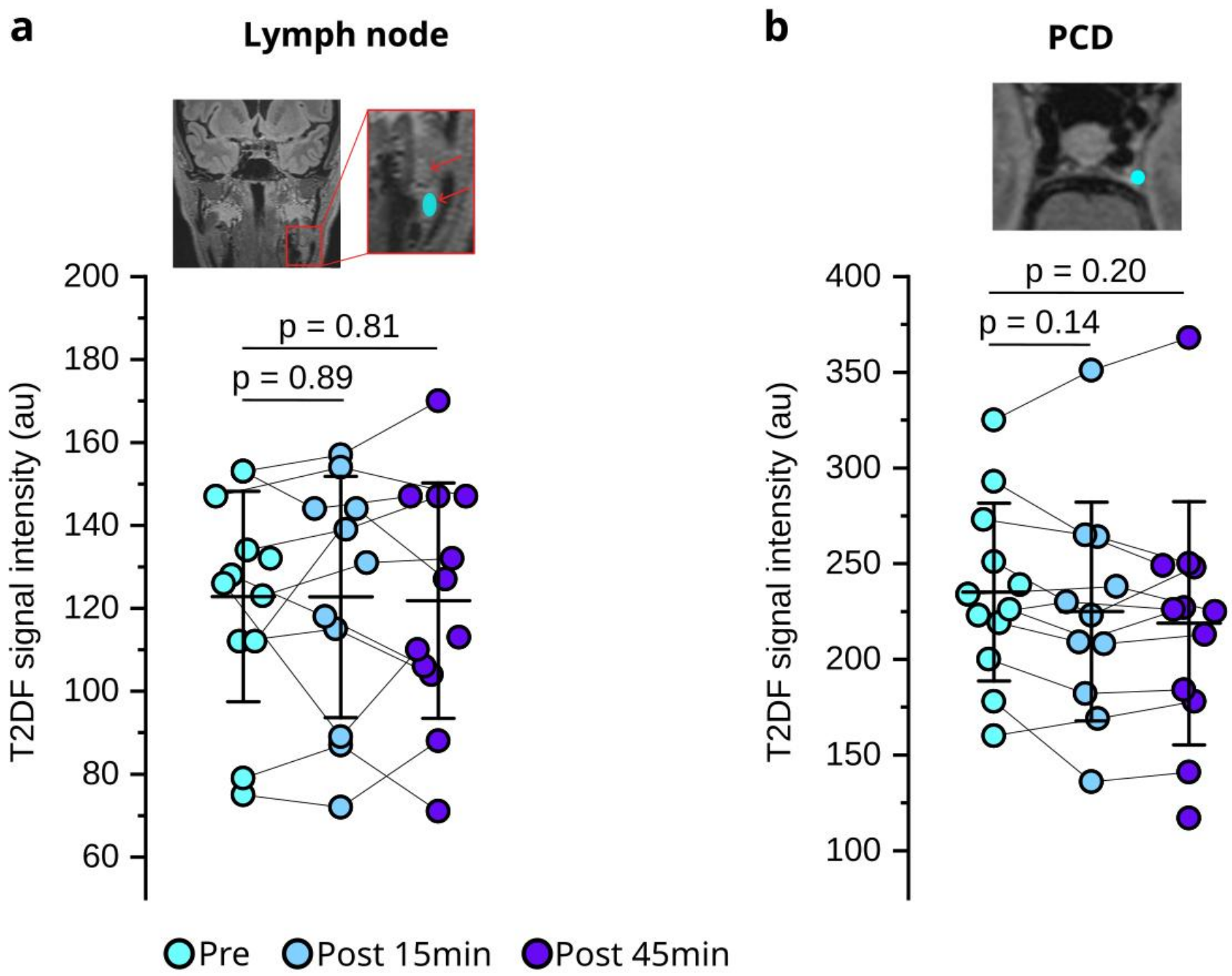

Supplementary Figure 16. ROI analysis of T2DF signal intensity in cervical lymph nodes and para-cavernous dura CSF spaces following NO administration. Analysis of T2 dark-fluid (T2DF) images of a. superficial cervical lymph nodes and b. para-cavernous dura CSF-filled spaces (PCD) before (Pre, $n = 12$) and after NO administration at 15 min (Post 15 min, $n = 11$) and 45 min (Post 45 min, $n = 12$). Mean (± standard deviation), paired two-tailed *t*-test. au = arbitrary units. Source data are provided as a Source Data file.

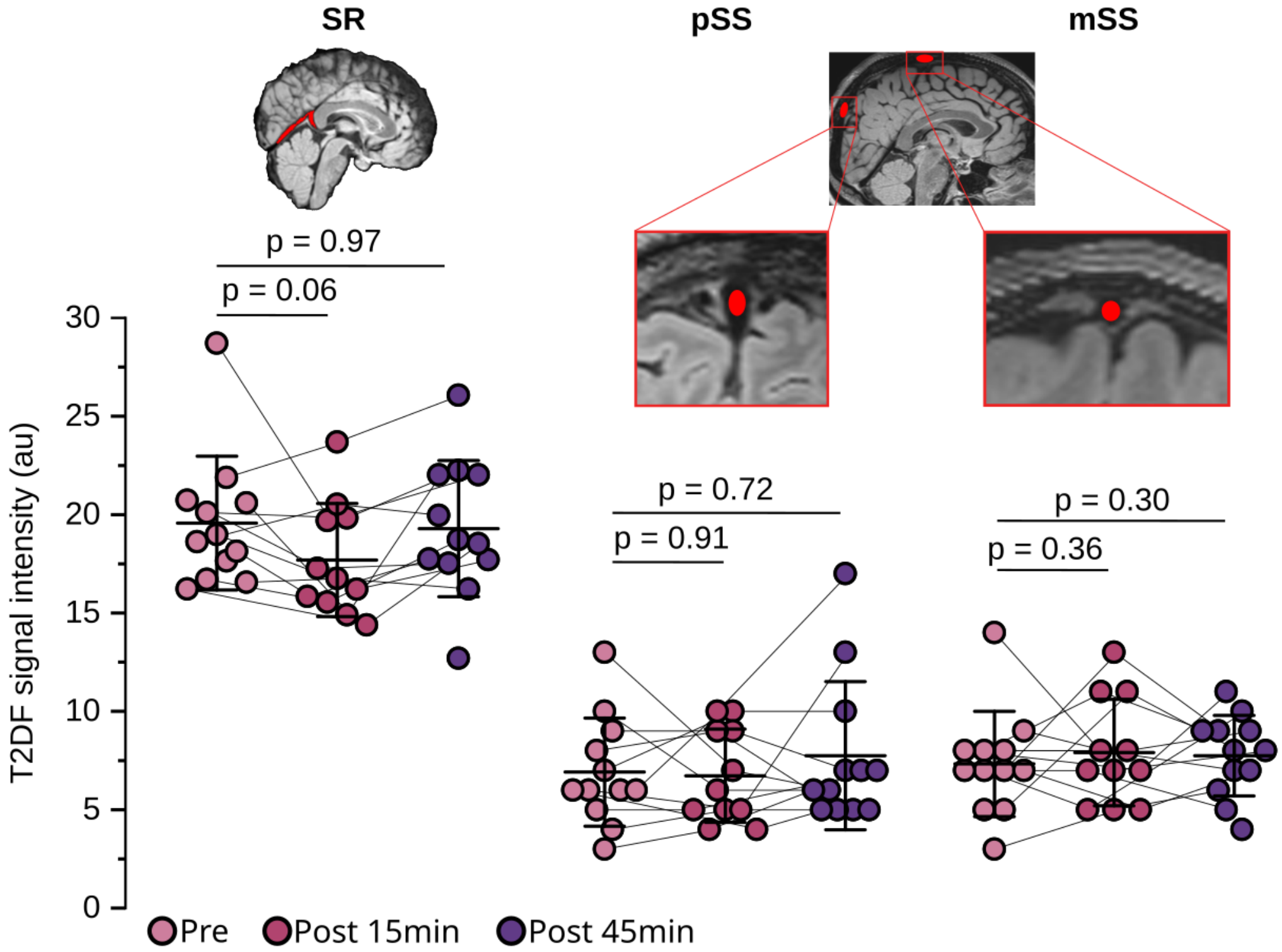

Supplementary Figure 17. ROI analysis of venous sinus T2DF signal intensity following NO administration. Analysis of T2 dark-fluid (T2DF) images of sinus rectus (SR$^{\#}$), posterior sagittal sinus (pSS$^{\#}$), and middle/vertex superior sagittal sinus (mSS) before (Pre, n = 12) and after NO administration at 15 min (Post 15 min, n = 11) and 45 min (Post 45 min, n = 12). As the blood underwent dilution according to the laboratory findings (cf. Fig 3l), the diluted blood would be expected to exhibit lower T2DF signal intensity. However, the absence of the expected T2DF signal decrease suggests increased protein content in the blood, compensating for the increase in free water. This interpretation is further supported by the Simoa laboratory findings (Fig. 1d). Thus, the net darkening of the whole brain is unlikely to be explained by increased brain tissue blood volume and more likely reflects a reduction in brain water and/or protein content during the NO condition. Mean (± standard deviation), paired two-tailed t-test. $^{\#}$Paired Wilcoxon signed-rank test. au = arbitrary units. Source data are provided as a Source Data file.

Piezo1-targeted whole-body vibration (WBV_p)

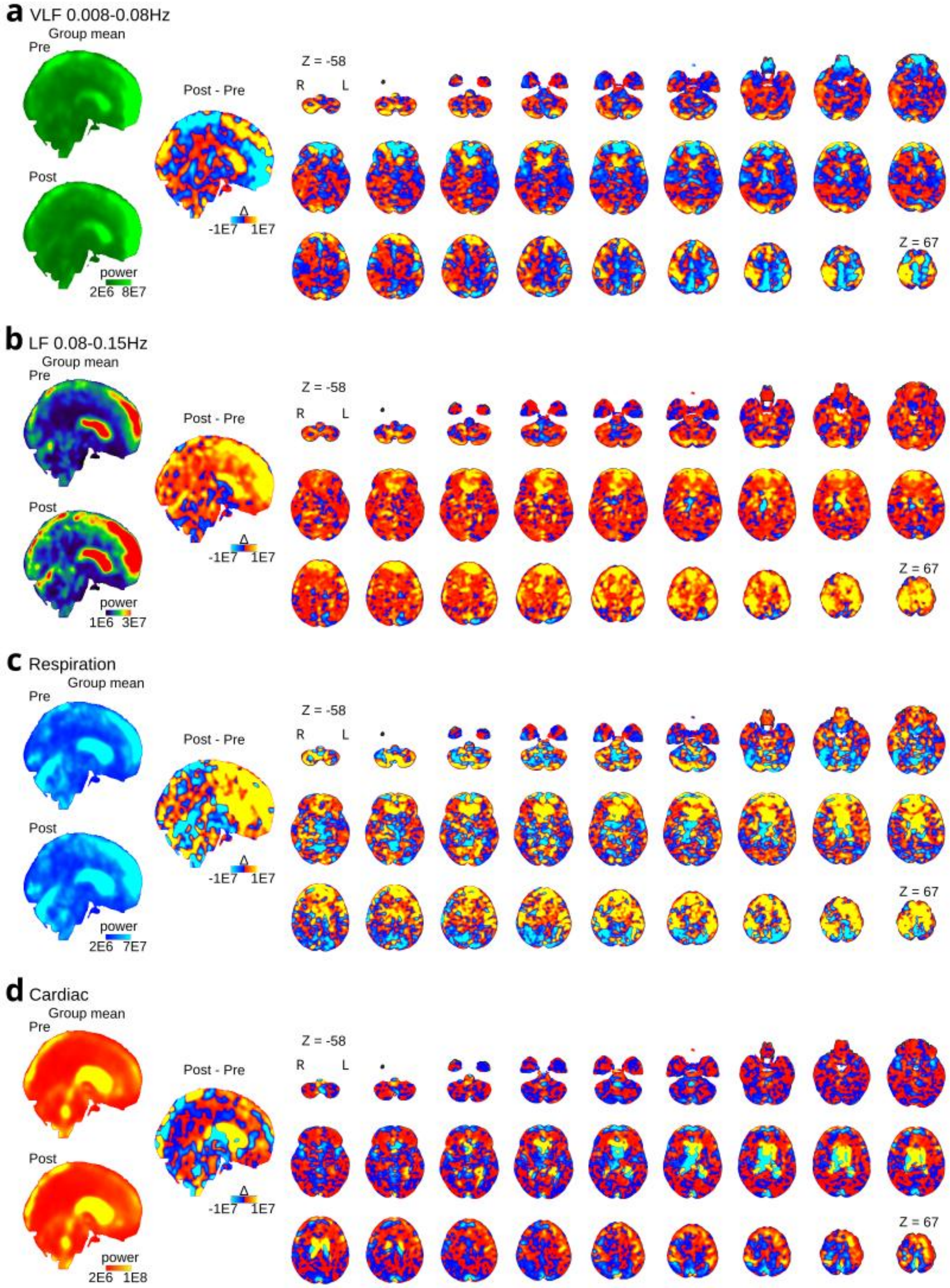

Supplementary Figure 18. Changes in MREG brain pulsation power following WBVp intervention. Group mean and subtraction (Post - Pre) maps of MREG brain pulsations during the whole-body vibration intervention (WBV_p, n = 25). a. very-low frequency (VLF, 0.008-0.08 Hz); b. low frequency (LF, 0.08-0.15 Hz); c. individual respiratory frequency; and d. individual cardiac frequency.

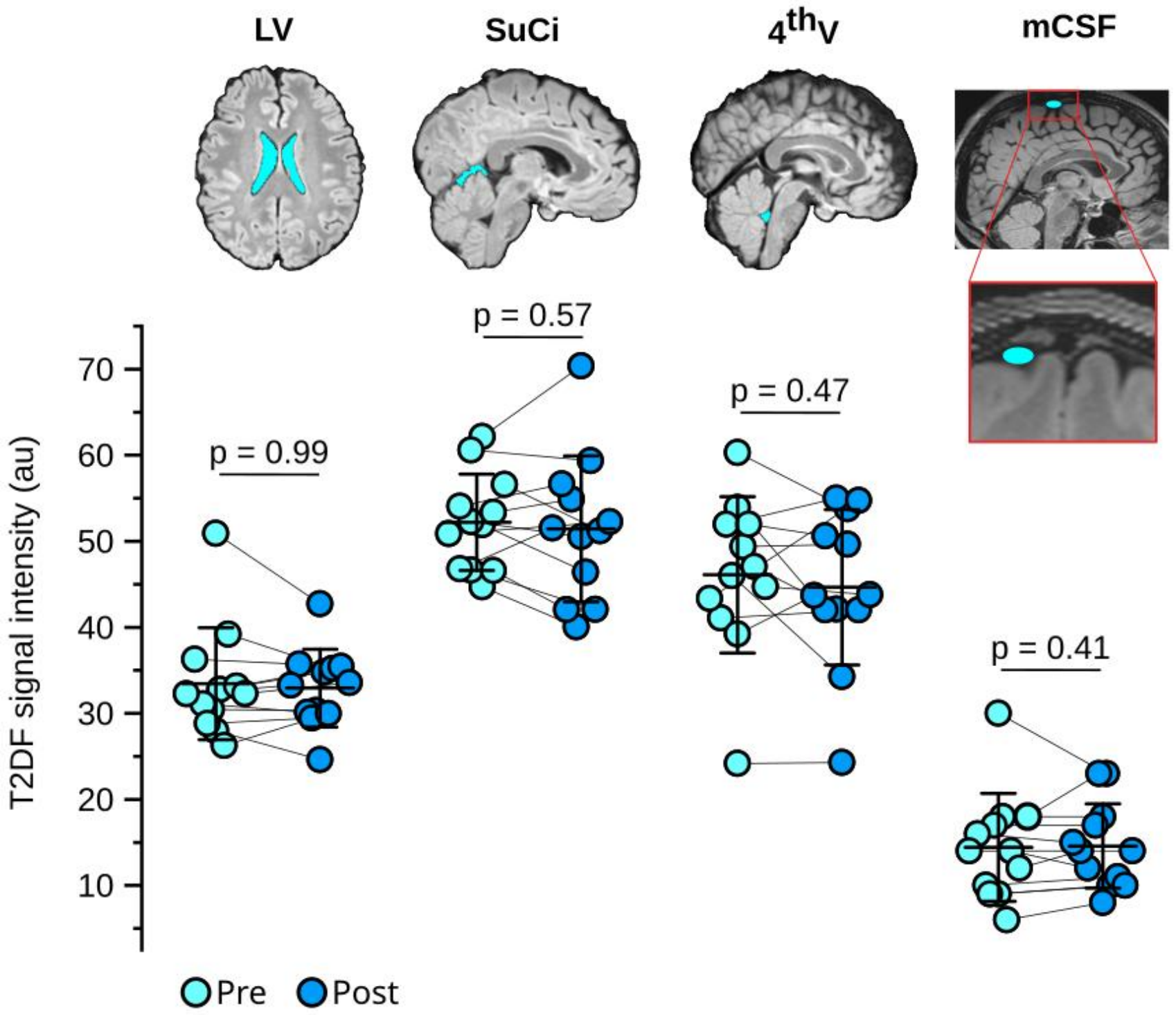

Supplementary Figure 19. ROI analysis of T2DF signal intensity in CSF spaces following WBV_p intervention. Analysis of T2 dark-fluid (T2DF) images of lateral ventricles ($LV^{\#}$), superior cisterna (SuCi), 4th ventricle (4^{th}V), and middle parasagittal CSF space (mCSF) before (Pre) and after (Post) Piezo1-targeted whole-body vibration intervention (WBV_p). Mean (± standard deviation), paired two-tailed *t*-test, *n* = 12. #Paired Wilcoxon signed-rank test. au = arbitrary units. Source data are provided as a Source Data file.

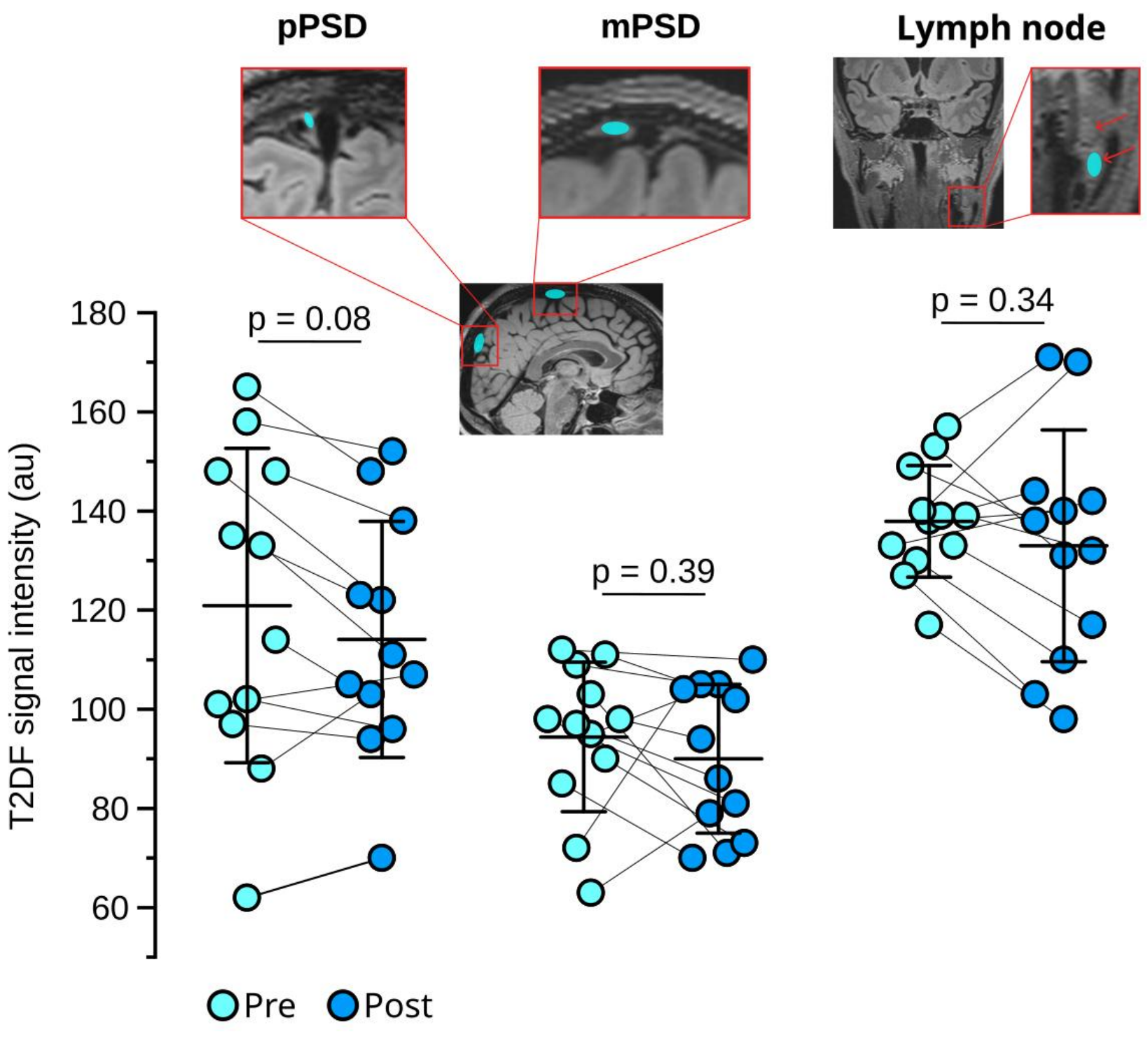

Supplementary Figure 20. ROI analysis of T2DF signal intensity in parasagittal dura spaces and cervical lymph nodes following WBV_p intervention. Analysis of T2 dark-fluid (T2DF) images of posterior parasagittal dura CSF-filled space (pPSD), middle/vertex parasagittal dura CSF-filled space (mPSD), and superficial cervical lymph nodes before (Pre) and after (Post) Piezo1-targeted whole-body vibration intervention (WBV_p). Mean (± standard deviation), paired two-tailed *t*-test, *n* = 12. au = arbitrary units. Source data are provided as a Source Data file.

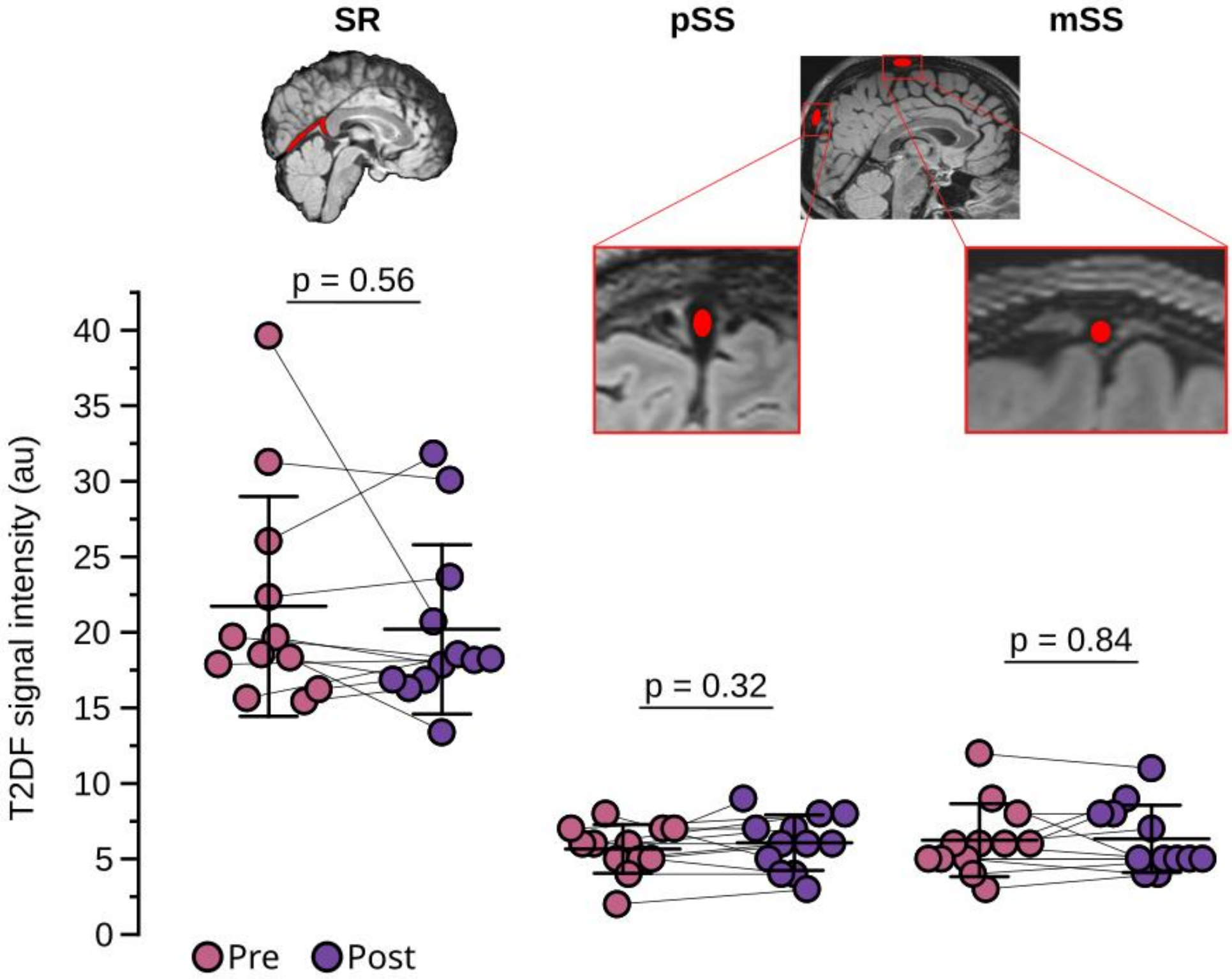

Supplementary Figure 21. ROI analysis of T2DF signal intensity in venous sinuses following WBV_p intervention. Analysis of T2 dark-fluid (T2DF) images of sinus rectus ($SR^{\#}$), posterior sagittal sinus (pSS) and middle/vertex superior sagittal sinus (mSS) before (Pre) and after (Post) Piezo1-targeted whole-body vibration intervention (WBV_p). Mean (± standard deviation), paired two-tailed *t*-test, $n = 12$. $^{\#}$Paired Wilcoxon signed-rank test. au = arbitrary units. Source data are provided as a Source Data file.

Sham intervention effects on T2 dark-fluid image

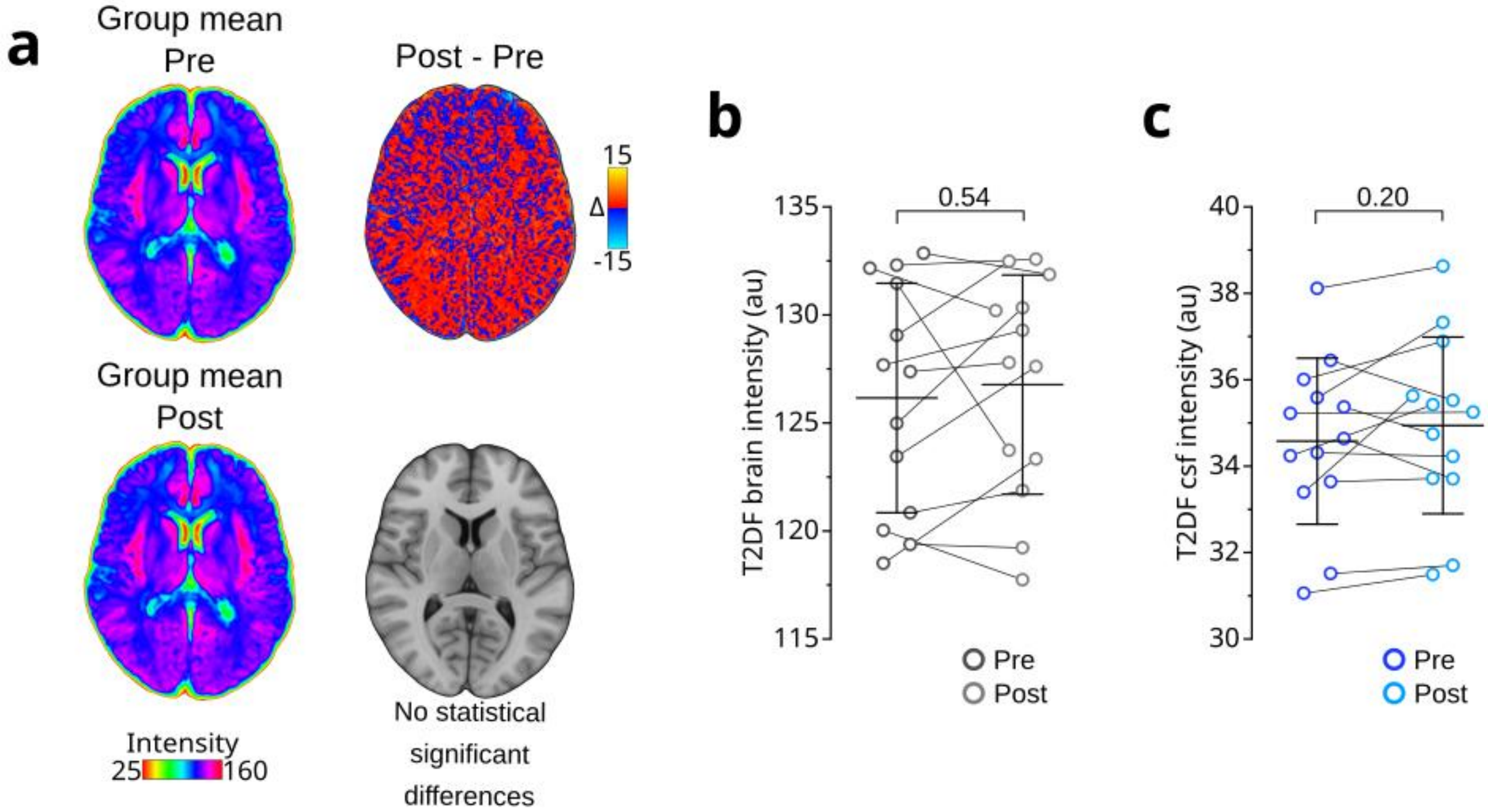

Supplementary Figure 22. Sham control intervention. a. Group mean T2-weighted dark-fluid (T2DF) MRI images at pre- and post-rest with corresponding subtraction (Δ, Post − Pre) maps. No statistically significant changes were observed in whole-brain T2DF signal intensity (two-sided paired *t*-test, family-wise-error-rate correction (FWER), $p < 0.05$). b. Whole-brain T2DF signal intensity before and after the sham condition. Mean (± standard deviation), paired two-tailed *t*-test. c. Cerebrospinal fluid (CSF) T2DF signal intensity before and after the sham condition. Mean (± standard deviation), paired two-tailed *t*-test. Each line represents an individual subject (n = 13). Although no significant differences were detected, subtraction maps indicate a tendency toward increased signal intensity following the resting period. The protocol consisted of repeated scanning before and after a 45-minute supine resting period without active intervention, matching the WBV_p treatment duration. au = arbitrary units. Source data are provided as a Source Data file.

Longitudinal analysis of T2DF signal intensity

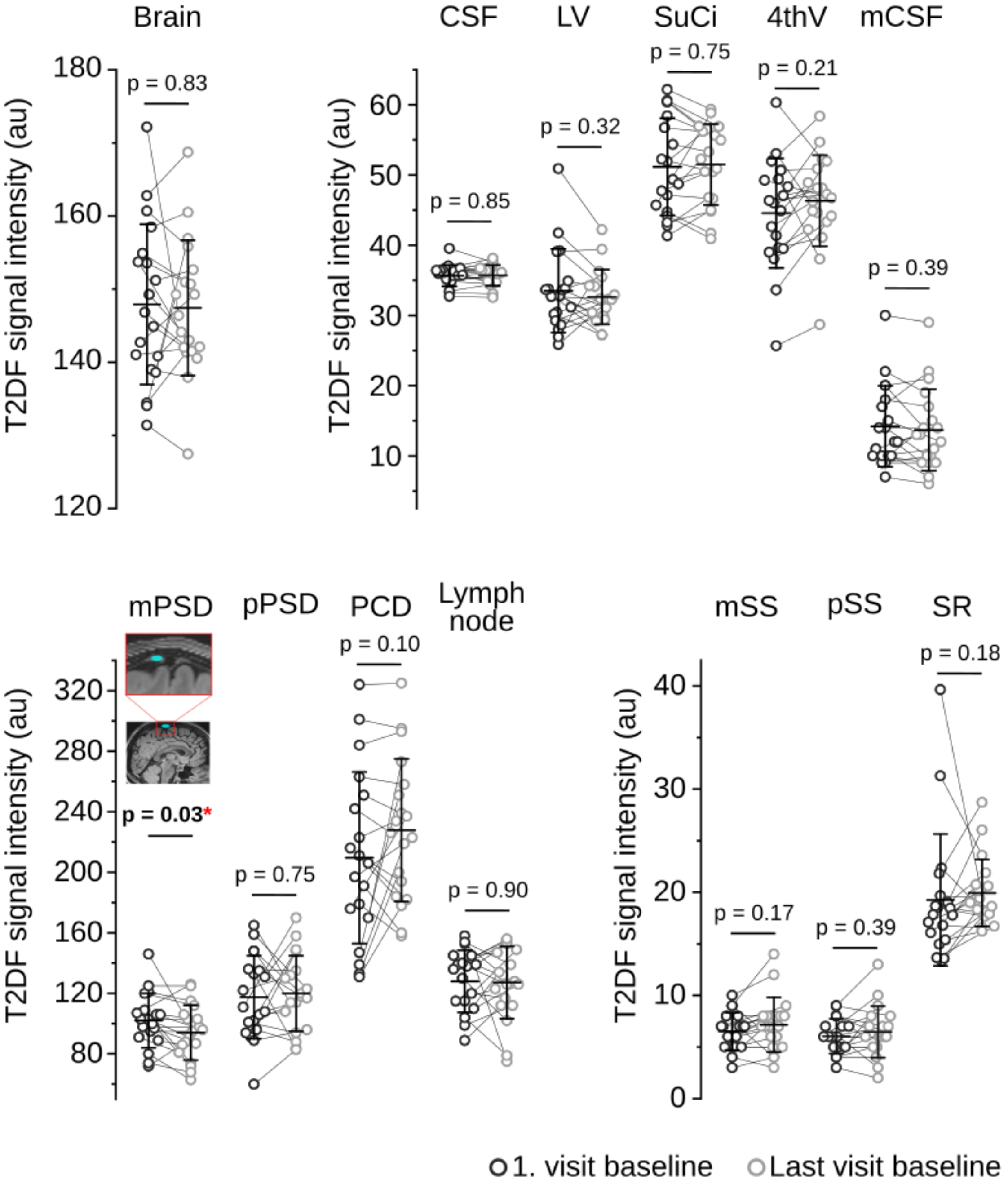

Supplementary Figure 23. Longitudinal stability of T2DF MRI signal intensity across study visits. T2-weighted dark-fluid (T2DF) signal intensity was quantified from regions of interest defined on the baseline image of the first visit and compared with the baseline image of the last visit (n = 19; mean interval 9.4 months, range 0.5–22.3 months). No significant changes were observed in whole-brain or cerebrospinal fluid (CSF) T2DF signal intensity between visits. Among individual regions, only the middle parasagittal dura CSF-filled space (mPSD) showed a significant decrease in signal intensity (p = 0.03), whereas all other regions remained stable. Data are shown for the following regions: Segmented whole-brain tissue (Brain), global cerebrospinal fluid (CSF), lateral ventricles (LV#), superior cisterna (SuCi), fourth ventricle (4thV), middle CSF space (mCSF#), middle parasagittal dura CSF-filled space (mPSD), posterior parasagittal dura CSF-filled space (pPSD), para-cavernous dura CSF-filled space (PCD), lymph node, middle/vertex sagittal sinus (mSS), posterior sagittal sinus (pSS), and sinus rectus (SR#). All scatter plots show means, with whiskers extending to one standard deviation. Each line represents an individual subject. Comparisons were performed using paired two-tailed t-tests. #Paired-sample Wilcoxon signed-rank test was used for comparison. au = arbitrary units. Source data are provided as a Source Data file.

Material and methods

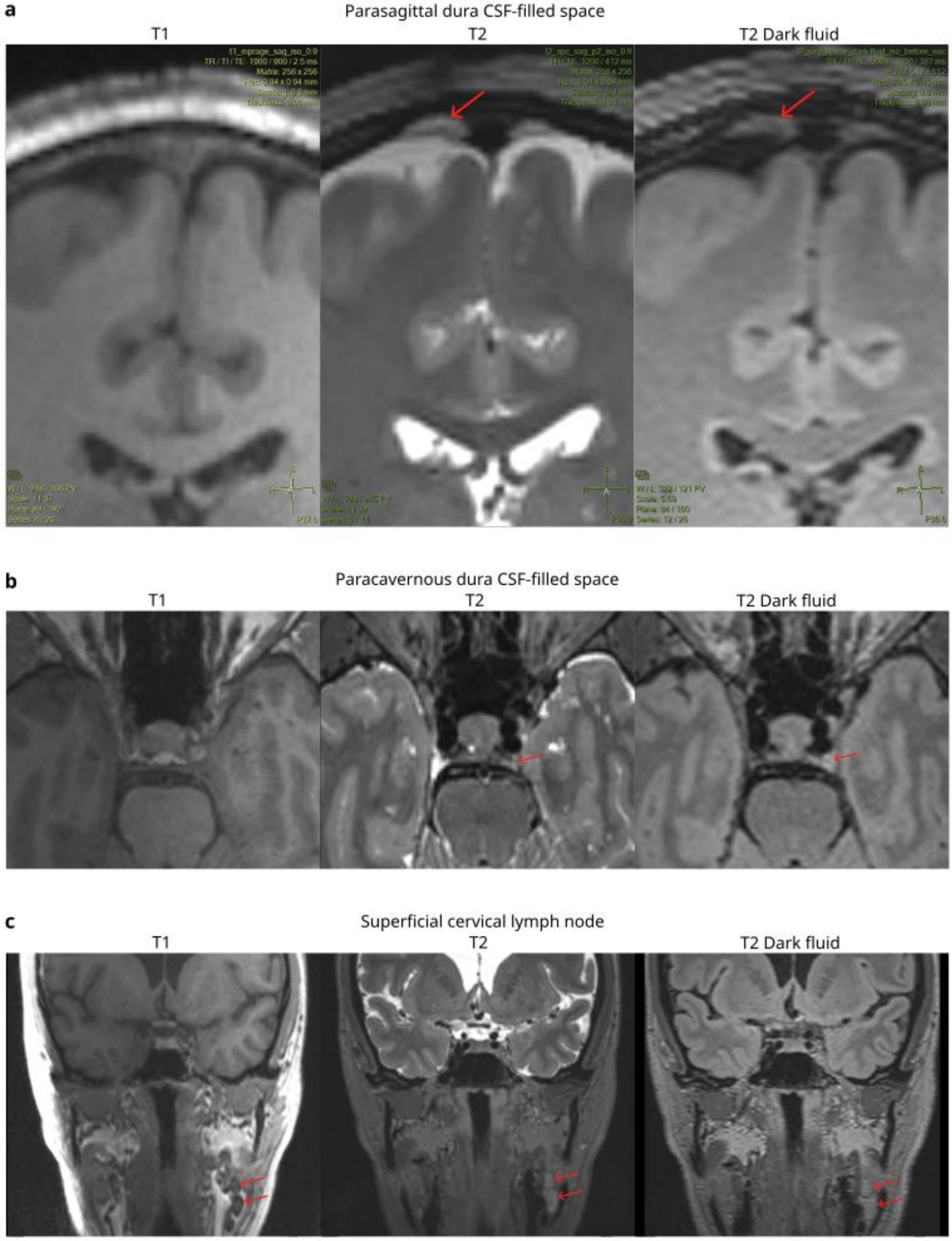

Supplementary Figure 24. ROI identification using T1- and T2-weighted images. Representative images to define a. parasagittal dura CSF-filled space, b. para-cavernous dura CSF-filled space, and c. superficial cervical lymph nodes from T2 dark-fluid images using T1- and T2-weighted images.

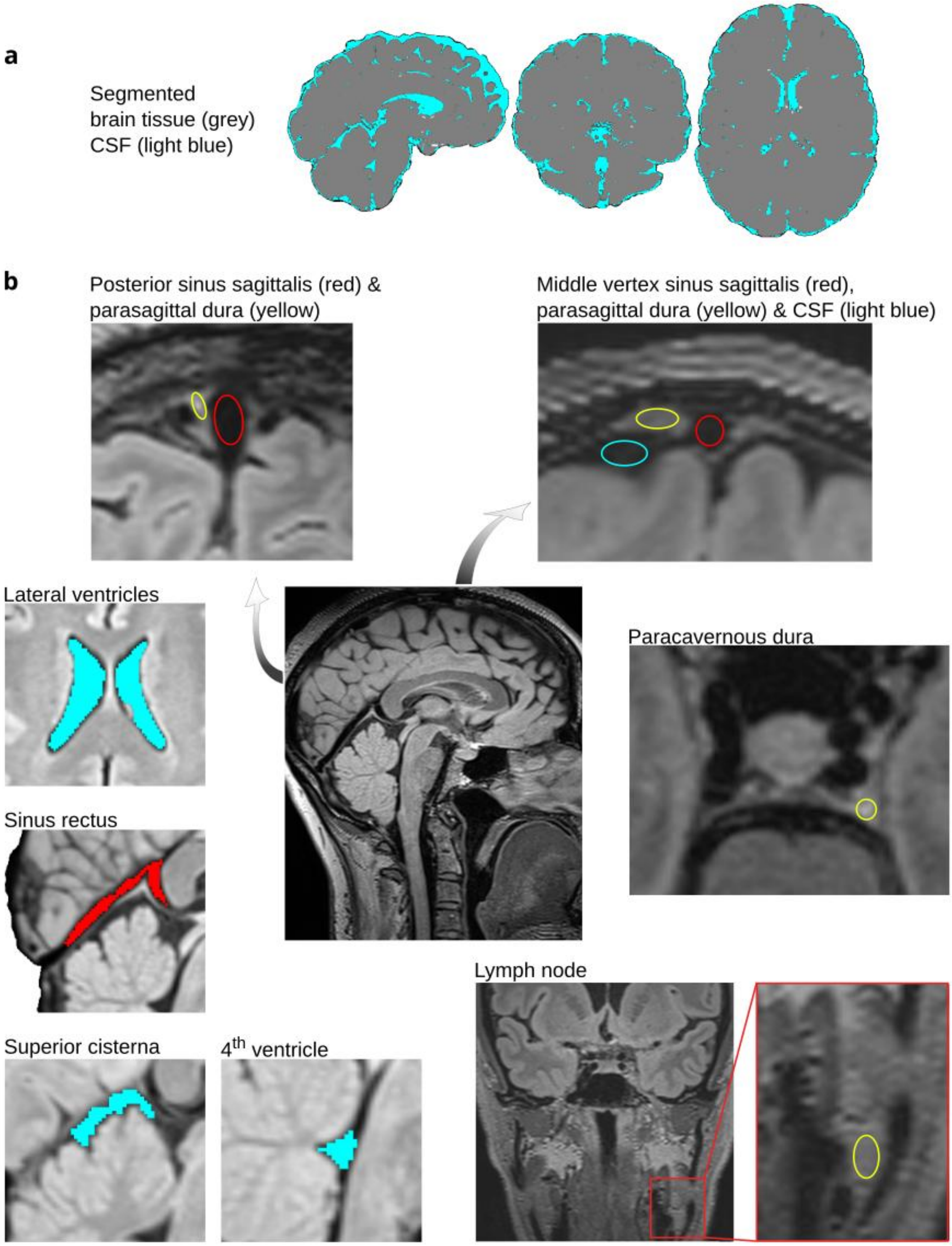

Supplementary Figure 25. Segmentation and ROI definition. a. Representative segmentation of brain tissue (grey) and cerebrospinal fluid (CSF, light blue) from a single participant from T2-weighted dark-fluid (T2DF) image. b. Representative examples of individually defined regions of interest (ROIs) from a single participant. Individual ROIs of posterior sinus sagittalis and parasagittal dura CSF-filled space, middle/vertex sinus sagittalis, parasagittal dura CSF-filled space and CSF, paracavernous dura CSF-filled space and lymph node were manually delineated on T2DF MRI images using a diagnostic DICOM viewer, guided by co-registered

T1- and T2-weighted images to ensure accurate anatomical localization of CSF-filled dural spaces and lymphatic structures. For each subject and scan, ROIs were drawn individually in matched anatomical slices and orientation to ensure direct comparability between pre- and post-intervention images. Anatomical regions were selected to maximize ROI size within each structure while avoiding partial volume effects, and identical ROI placement was maintained between time points within subjects. Adjacent structures (sinus sagittalis and parasagittal dura CSF spaces) were defined within the same slice. Individual ROIs of lateral ventricles, fourth ventricle, superior cisterna, and sinus rectus were generated using FSL-based thresholding and masking tools, and the same masks were applied to both pre- and post-intervention images for each subject.

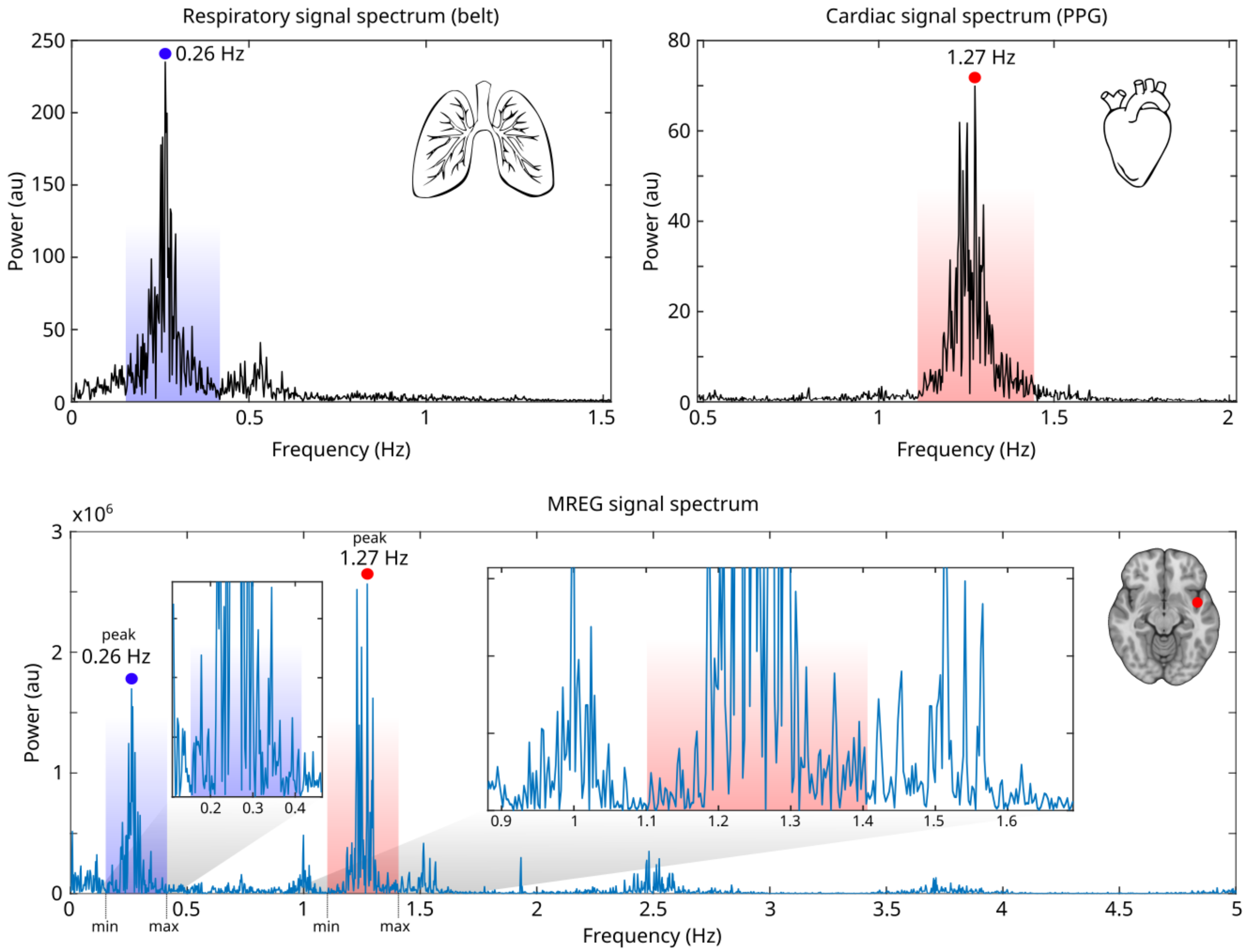

Supplementary Figure 26. Validation of cardiorespiratory frequency estimation between MREG and physiological measurements. Representative power spectra of respiratory (belt) and cardiac (photoplethysmogram, PPG) signals showing individual peak frequencies and frequency ranges (minimum to maximum). Corresponding MREG signal spectra are shown below, demonstrating alignment of respiratory and cardiac peaks with the independently measured physiological signals. Shaded areas indicate the frequency ranges used for subsequent analysis. au = arbitrary units. Source data are provided as a Source Data file. Illustration: Johanna Tuunanen, University of Oulu.

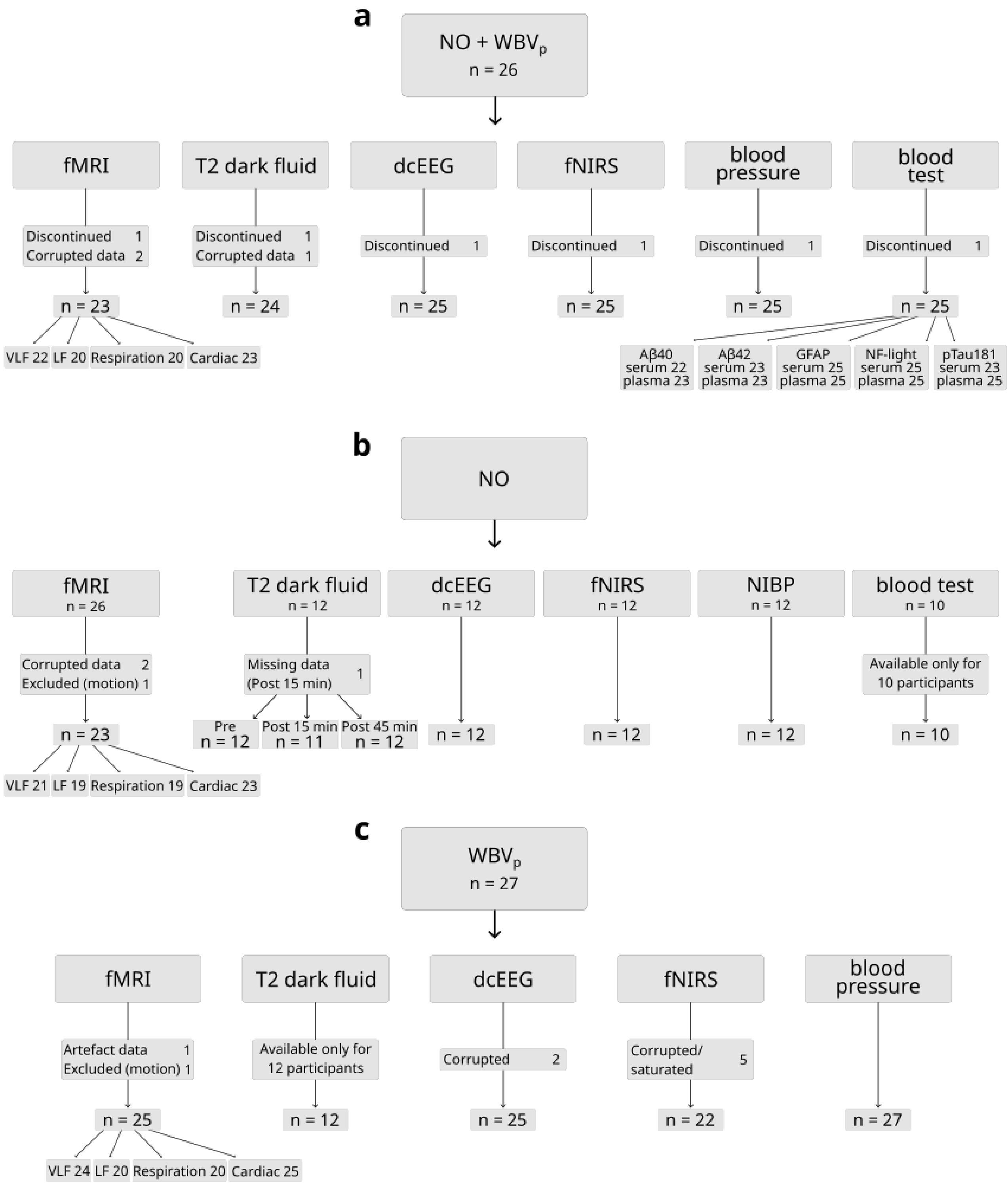

Supplementary Figure 27. Availability of multimodal datasets across interventions and study design. a. Combined NO-mediated vasodilation with Piezo1-targeted whole-body vibration ($NO+WBV_p$) intervention within the randomized crossover study (n = 26), showing the number of available datasets for each modality (fMRI, T2DF MRI, dcEEG, fNIRS, blood pressure, and blood biomarkers). b. NO-mediated vasodilation (NO) condition. fMRI data (n = 23) were acquired during the NO phase of the combined $NO+WBV_p$ intervention visit in the crossover study. Additional multimodal NO data (T2DF MRI, dcEEG, fNIRS, NIBP, blood tests; n = 12) were obtained from a separate exploratory NO-only sub-study conducted on a different experimental day. c. Piezo1-targeted whole-body vibration (WBV_p) intervention within the randomized crossover study (n = 27), showing modality-specific data availability after exclusion of corrupted or incomplete datasets. Across all panels, the figure illustrates dataset availability for each modality, including exclusions due to motion, artefacts, or incomplete recordings.

Mouse study

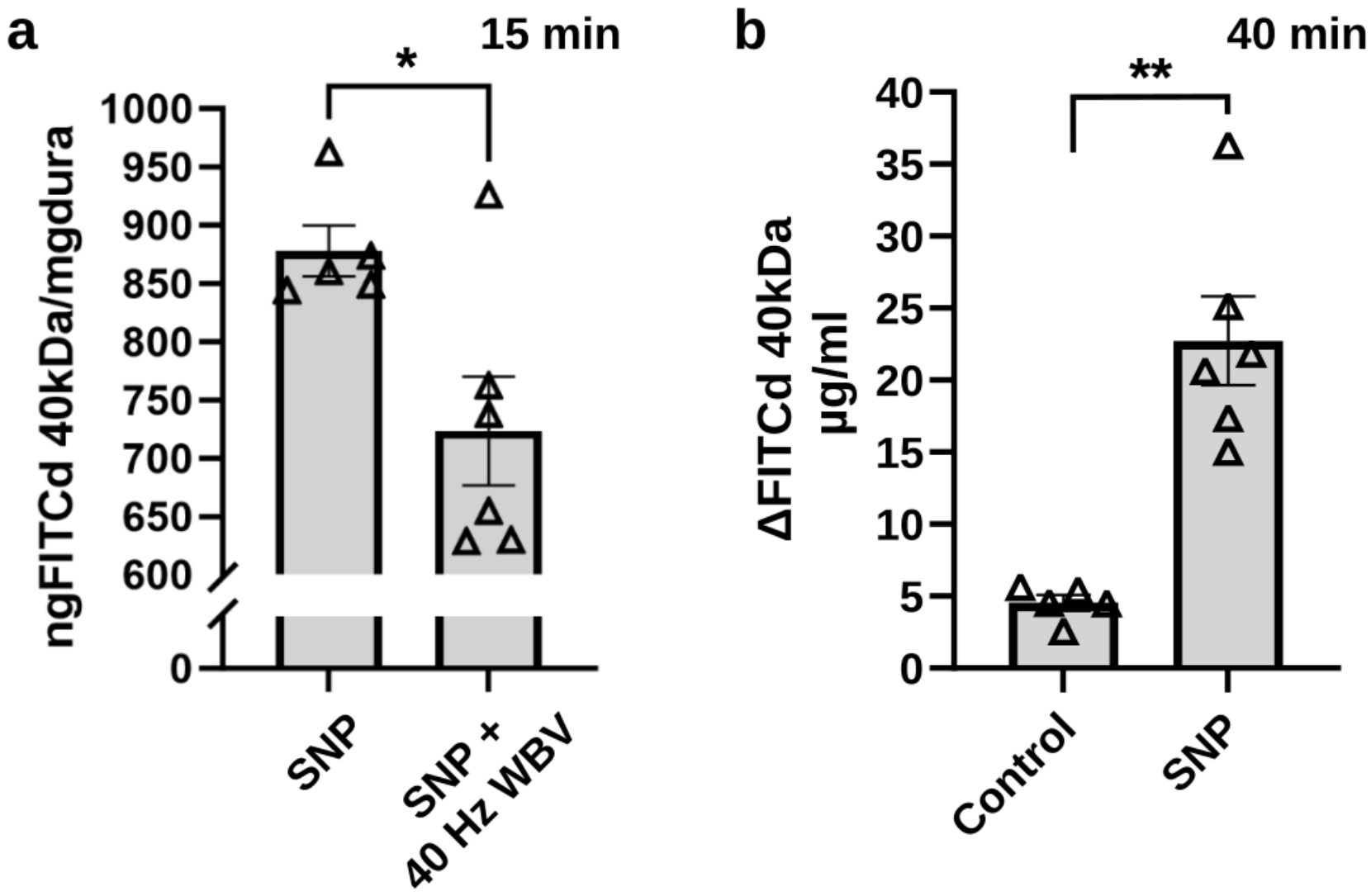

Supplementary Figure 28. Complementary intrathecal tracer studies in mice. a. Vasodilation with sodium nitroprusside (SNP) combined with 40 Hz vibration treatment (WBV, $n = 6$) for 15 min results in significantly less tracer (FITC-dextran 40 kDa) accumulation in dorsal dura mater compared to mice treated with SNP alone ($n = 5$). Unpaired two-tailed *t*-test with Welch's correction, *$p = 0.0199$, mean ± standard error of mean (SEM) b. 40 kDa FITC-dextran concentration increased in blood during 40-min NO-mediated vasodilation with SNP ($n = 6$) compared to mice treated with saline (Control, $n = 5$). Unpaired two-tailed *t*-test with Welch's correction, **$p = 0.0017$, mean ± SEM. Source data are provided as a Source Data file.

Supplementary references

1. Haverinen, J. *et al.* Heightened respiratory brain pulsations indicate fluid dynamic dysfunction in early multiple sclerosis. *Journal of Cerebral Blood Flow & Metabolism* **46**, 1453802 (2026).
2. Järvelä, M. *et al.* Increased very low frequency pulsations and decreased cardiorespiratory pulsations suggest altered brain clearance in narcolepsy. *Communications Medicine* **2**, 122 (2022).
3. Järvelä, M. *et al.* Orexin effect on physiological pulsations of the human brain. *Proceedings of the National Academy of Sciences* **122**, (2025).
4. Kananen, J. *et al.* Increased interictal synchronicity of respiratory related brain pulsations in epilepsy. *Journal of Cerebral Blood Flow & Metabolism* **42**, 1840–1853 (2022).
5. Tuovinen, T. *et al.* The variability of functional MRI brain signal increases in Alzheimer's disease at cardiorespiratory frequencies. *Sci. Rep.* **10**, 21559 (2020).
6. Tuunanen, J. *et al.* Cardiovascular and vasomotor pulsations in the brain and periphery during awake and NREM sleep in a multimodal fMRI study. *Front. Neurosci.* **18**, (2024).
7. Jukkola, J. *et al.* Blood pressure lowering enhances cerebrospinal fluid efflux to the systemic circulation primarily via the lymphatic vasculature. *Fluids Barriers CNS* **21**, 12 (2024).
