# Supplementary material for: Vasomechanic treatment is associated with increased efflux of human brain proteins
Source: Nat Commun. 2026 Nov 3;17:11164. doi: 10.1038/s41467-026-78894-y (PMC13731658; doi:10.1038/s41467-026-78894-y)
Supplement: Supplementary file 3 [file 41467_2026_78894_MOESM3_ESM.pdf]

nature portfolio

Peer Review File

Vasomechanic treatment is associated with increased efflux of human brain proteins

Corresponding Author: Ms Johanna Tuunanen

Parts of this Peer Review File have been redacted as indicated to remove third-party material.

This file contains all reviewer reports in order by version, followed by all author rebuttals in order by version.

Version 0:

Reviewer comments:

Reviewer #1

(Remarks to the Author)
This paper investigated NO-mediated vibration modulation to brain glymphatic system based on human studies. Effects of the vibration modulation by NO, WBV, and NO_WBV were compared and discussed. Results showed a synergistic enhancement of brain fluid transport, and markedly increased the efflux of soluble proteins into the bloodstream. This paper is well-written and the vibration modulation of the brain glymphatic system is promising for clinical translation in the intervention of neurodegenerative diseases. I only have a few suggestions for improvement of the manuscript:
1.It is interesting to see the brain volume decreased and the CSF volume increased significantly as shown in Fig. 1g-h. Does this mean WBV can produce physical changes of the brain? Some explanations would be helpful.
2.Could you please explain the increase and decrease patterns of the intensity at different locations in Fig. 1 k-m? If efflux was enhanced, should they be all increased in terms of signal intensity?
3.Is it possible that the significant intensity decreases of the CSF space for the WBV as shown in Fig. 4k may due to the down modulation of the blood flow?
4.The wide frequency range for vibration stimulation overtime as shown in Fig. 4c is a bit complex. It will be ideal to have single frequency analysis. Since it's a pilot human study, some discussion would be helpful as there are many frequency-specific studies showing the modulation effects as mentioned in the introduction section.
5.The mouse experiment showed promising results with 40 Hz. To be consistent with the methods used for human in this study, would it be helpful to have the same WBV application to the mouse to have a comparison?
6.Signal intensity of the T2DF images were used for evaluation of the efflux. There are many MR imaging modalities that can be used for quantification, e.g. ASL, DTI. This can be considered in future studies.

Reviewer #2

(Remarks to the Author)
This is a substantial and timely study that addresses an important question in human brain clearance physiology through an ambitious multimodal design integrating physiological monitoring, blood biomarkers, and MRI based readouts. The human dataset is valuable, the overall experimental concept is compelling, and several aspects of the work are potentially important for the broader translational literature in neurodegeneration and brain fluid biology. At the same time, a number of conceptual, methodological, and reporting issues should be addressed before the principal conclusions can be supported at the level currently stated. In particular, the manuscript would benefit from a more disciplined alignment between the data and the mechanistic claims, together with several clarifications that would improve analytical transparency and overall interpretability.

Major Comments:

1. The manuscript presents interesting and potentially important evidence that the combined intervention is associated with increased circulating Aβ40, Aβ42, and GFAP together with imaging changes consistent with altered neurofluid dynamics. However, the central interpretation currently extends beyond what the data can firmly support, particularly where these findings are discussed as evidence of direct enhancement of brain to blood protein clearance. The distinction between

association and mechanism should be drawn more carefully throughout the Results and Discussion. In particular, the authors should consider alternative explanations for the observed biomarker shifts, including systemic redistribution, transient barrier related effects, and other hemodynamic influences associated with the intervention. As presented, the dataset is clearly suggestive, but the strongest causal language should be moderated unless supported by additional orthogonal evidence.

2. The dark fluid MRI analysis is central to the interpretation of the study and directly informs the broader conclusions regarding clearance related physiology and brain protein redistribution. In this manuscript, the imaging findings serve not simply as supportive observations, but as the principal framework through which the post intervention biomarker changes are linked to altered fluid and macromolecular behavior within the brain and adjacent dural compartments. This aspect of the work therefore warrants a more fully developed methodological and conceptual discussion. The use of a high resolution non contrast dark fluid, FLAIR type acquisition is an important strength, particularly in the context of human studies where repeated assessment may be required. At the same time, the interpretive scope of these signal changes should be defined with greater precision. Specifically, the Discussion would benefit from a more explicit account of how the observed dark fluid alterations should be understood in relation to changes in water and macromolecular distribution, rather than being carried too quickly toward route level mechanistic inference. Strengthening this section would materially improve the manuscript by aligning the imaging results more closely with the biological claims being made and by clarifying the broader relevance of this MRI framework to the study of human brain clearance physiology.

3. The conclusion that the combined intervention exerts a synergistic effect is currently stronger than the statistical evidence presented. Although the combined condition appears to produce larger effects than the individual intervention arms, descriptive comparison across conditions is not sufficient to establish synergy. This point should therefore be addressed more formally, either through an explicit interaction analysis or through more restrained wording that refers to a greater combined effect rather than confirmed synergy. As written, the current interpretation overstates what the comparative analysis can support.

4. The statistical framework requires clearer definition in light of the exploratory, multimodal structure of the study. In particular, the manuscript would benefit from a more explicit specification of the primary endpoint, or primary endpoint family, together with a clearer account of how variation in sample size across modalities and analyses was handled. The study is presented within a pilot framework, yet the manuscript integrates multiple physiological, imaging, and biomarker readouts across partially overlapping cohorts. Under these conditions, a more clearly articulated analytical hierarchy is needed to distinguish hypothesis driven outcomes from broader exploratory observations and to improve the overall interpretability of the results.

5. The alignment between the clinical protocol and the final intervention description should be clarified more carefully, particularly with respect to nitroglycerin dosing and timing. The protocol describes a pilot design in which nitroglycerin was administered around imaging and treatment periods under blood pressure based constraints, and because the key physiological and biomarker findings are tightly linked to hypotensive responses, it would be important for the final manuscript to state explicitly how the analyzed NO and combined intervention arms were operationally implemented, whether the dosing structure was identical across participants, and whether any protocol refinements occurred during the course of the study.

6. The safety and tolerability statements should be framed with greater precision. Although no serious adverse events were reported and the study appears feasible under monitored conditions, the manuscript also documents headache, nausea, and one discontinuation due to severe nausea. The wording should therefore be moderated accordingly, with the intervention described more specifically as feasible and acceptably tolerated in a closely supervised short term study of healthy volunteers. A clearer presentation of adverse events by intervention condition and severity would further improve the clinical transparency of the report.

7. The murine experiments add useful biological support to the study, but their translational role should be defined with greater precision. In the current form of the manuscript, the animal data occasionally come close to being presented as mechanistic validation of the clearance route inferred from the human imaging findings. A more disciplined framing would be preferable. Specifically, the murine results are better positioned as complementary experimental support for the broader biological model rather than direct validation of the precise human pathway inferred from the present data. This distinction is important in the meningeal lymphatic and glymphatic field, where broad conceptual overlap across species coexists with substantial differences in anatomy, scaling, drainage predominance, and route visibility. Clarifying this point would strengthen the translational argument while preserving the importance of the animal work.

8. The age related findings are intriguing and potentially important, but I would suggest presenting them more clearly as exploratory unless their robustness can be further established. The cohort size is still modest, the age range is limited, and the main manuscript notes outlier masking in at least one correlation analysis. Under these conditions, the age dependent interpretation should be phrased carefully and ideally supported with fuller sensitivity analyses and transparent display of all data points.

9. The central biological interpretation of the manuscript rests heavily on rapid post intervention changes in circulating biomarkers, and this aspect of the study would benefit from further analytical support. In particular, the interpretation would be strengthened by a more explicit assessment of biomarker kinetics and concordance, including closer integration of serum and plasma findings, fuller accounting for hemodilution related variables, and clearer subject level comparison between biomarker shifts and the corresponding physiological or imaging changes. If additional post intervention time points are

available, these would be especially informative. If not, the mechanistic interpretation should be correspondingly moderated.

Minor Comments

1. The presentation of participant numbers should be made more consistent across the manuscript. Although the supplement provides a CONSORT style overview and modality specific availability, the main text would be easier to follow if the reasons for moving from 29 enrolled participants to smaller analytic subsets were stated more explicitly at the first relevant point in the Results.

2. The nomenclature of the intervention arms should be standardized throughout the manuscript and figure legends. At present, WBVp, WPVp, NO+WBVp, and NO+WPVp appear interchangeably. A uniform terminology would improve clarity, particularly when comparing the isolated and combined intervention conditions.

3. The manuscript would benefit from a careful language edit for precision and fluency. There are several small but noticeable issues in the current text, including “of who 15 females,” “combined to Piezo1 targeted whole body vibration,” “At 15 ans 45 min,” “with the any of the whole body vibration procedure,” and “not significantly unaffected.”

4. The statistical terminology should be revised where “two sample paired t test” is used in the Methods and figure legends. If the analyses were conducted within subjects, they should simply be described as paired t tests.

5. The Methods would benefit from a more explicit account of the timing of blood sampling and MRI acquisition relative to the intervention period in each study arm. This is particularly important because the interpretation of both the biomarker and dark fluid MRI findings depends closely on temporal proximity to the physiological response.

6. A brief justification should be provided for treating serum as the principal matrix for the core biomarker analyses while presenting plasma data in the supplement. Since both are shown, this distinction should be clarified.

7. Because hemodilution is central to the interpretation of the biomarker results, the Methods should state more explicitly how the correction factor was derived, whether it was applied uniformly across analytes, and whether the main conclusions were robust to uncorrected versus corrected analyses.

8. The ROI based dark fluid analyses would be easier to evaluate if the Methods provided slightly more detail regarding ROI delineation. In particular, it would be helpful to clarify whether raters were blinded to condition, whether ROIs were drawn independently on each scan or propagated across time points, and whether any internal consistency checks were performed.

9. The pre scan standardization procedures described in the protocol, including avoidance of coffee, energy drinks, and alcohol before study visits, appear relevant to the vascular and physiological readouts and should be reported in the main Methods section.

10. The protocol includes additional assessments such as the PAL memory test, sleep questionnaire, and BDI 21, whereas the manuscript focuses primarily on physiological, imaging, and blood biomarker outcomes. If these measures were prespecified but are outside the scope of the present report, a brief clarification in the Methods or supplement would help avoid uncertainty regarding their role in the overall study design.

11. The Results and Discussion would read more clearly if the distinction between global segmented dark fluid changes and ROI specific signal changes were maintained more consistently. At several points, the interpretation moves rapidly between whole brain, CSF, dural, and venous compartments, which makes the imaging narrative harder to follow.

12. The figure legends should report sample size more consistently at the panel level. In several figures, different panels appear to derive from different analytic subsets, but the corresponding n is not always immediately apparent for each comparison.

13. The figures are scientifically rich, but several panels are visually dense. In particular, figures that combine whole brain maps, ROI quantification, and physiological traces would benefit from a more orderly internal progression, so that the reader is guided from global observations to regional quantification and then to physiological support.

14. In Figure 1, the representative anatomical insets and ROI illustrations are useful but relatively small given the interpretive importance of the parasagittal dural and venous regions. Enlarging these panels, if feasible, would improve anatomical clarity.

15. Figure 5 is useful as a summary figure, but the legend should distinguish more explicitly between experimentally observed findings and interpretive schematic elements. This would allow the figure to function more effectively as a synthesis panel without conflating measured data with proposed mechanism.

16. The color scale conventions used for the pre, post, and difference maps should be clarified more explicitly in the figure legends. Where visual comparison across panels is central to interpretation, it should be immediately clear whether identical intensity ranges and scaling conventions were applied.

17. Several figure legends are highly compressed relative to the complexity of the panels they describe. In the more data

dense figures, particularly Figures 1, 3, and 4, a slightly more structured legend format would improve readability and reduce ambiguity regarding which statistical comparisons correspond to which panels.

Reviewer #3

(Remarks to the Author)
In the manuscript “Vasomechanic treatment increases efflux of human brain proteins” the authors Tuunanen et al study the combined delivery of nitric oxide (NO) and whole body vibrations (WBVp) in healthy adults (n = 29) on brain fluid dynamics and 27 CNS-to-blood protein efflux. The authors conclude that this intervention is a promising method for enhancing clearance of amyloid from the brain. Brain clearance in humans is still ill-understood and any study showing significant changes is therefore of importance. Unfortunately, this study has a sub-optimal design and the readout techniques can be challenged. I would certainly more information to be able to fully appreciate their experiments.
My main concerns are:
1. The design of the experiments is sub-optimal. The NO condition was later tested in another cohort, the WBVp could not be done while in the MRI, and the study lacks a proper control condition. The lack of a control condition makes pre-post analysis difficult to interpret, as repeated MRI is known to induce changes in stress within participants. Or were all participants experienced MRI volunteers?
2. The use of quantitative T2 dark fluid employed as by the authors, would need further validation. Signal intensities in MRI are easily influenced by e.g. preparation phases (power optimalization, frequency determination) as well as by post-processing (bias-field correction). Moreover, in the CSF the intensities are near zero, where Rician noise issues come into play. Without more convincing validation, I cannot be certain that the observed changes can indeed be interpreted as a changes in molecular content of CSF. This is a major point and would need more experiments.
3. In the MREG spectrograms, it is quite clear that cardiac and respiratory frequencies are quite drastically changed during the interventions. It is unclear whether their post-processing accurately corrects for this. The text is too vague (ranges and peaks were obtained from individual FFT spectrums; frequencies were double checked with the physiological recordings of the scanner), please explain exactly how this was done. Also, unclear why the scanner recordings were not used as an independent way to determine the frequency ranges. Now, the authors are double dipping: first use the spectrum to determine the frequency range and then integrate the same spectrum over that ranger. It is also unclear whether the person doing the post-processing was blinded towards the condition and whether pre- and post were scored immediately after each other. More information is needed and a second observer would be nice to prove objectivity of the measurements.
4. I find the cartoons of Figure 5 rather speculative and it would need more text to fully explain why certain arrows are drawn: i.e. for each process depicted in the cartoon, clearly indicate on what data/finding that is based. This field suffers from too many diagrams, with little data underlying the schemes.
5. Please subtract pre- and post power maps in figure 3c and 4f and show these alongside the pre- and post maps.
6. Please connect dots in all figures (pre - post) for each participant.
7. In all figures, always display both positive and negative effects (e.g. Figure 2C).
8. Please discuss in much more detail how CSF motion could also influence T2 dark fluid signal intensities.

Version 1:

Reviewer comments:

Reviewer #1

(Remarks to the Author)
The authors have addressed all my questions. Mechanical stimulation provides a new and helpful way for brain modulation. I recommend acceptance of this paper.

Reviewer #2

(Remarks to the Author)
Thank you for the comprehensive and thoughtful revision. I have carefully reviewed the response letter and the revised manuscript. The authors have addressed the major conceptual, methodological, and reporting concerns in a substantive manner, and the manuscript has improved considerably.

The interpretation is now more appropriately aligned with the evidence. The removal of the synergy claim, the clearer distinction between the randomized crossover study and the exploratory cohorts, the definition of the primary endpoint family, and the improved reporting of sample sizes have strengthened the analytical framework. The expanded descriptions of intervention timing, hemodilution correction, serum and plasma findings, ROI delineation, adverse events, and the complementary role of the murine studies have also improved transparency and reproducibility. The additional analyses linking posterior venous sinus T2DF signals with circulating biomarkers provide valuable convergent support for the overall interpretation.

I am satisfied that my principal concerns have been addressed. Before publication, I recommend only two limited clarifications. First, the title and final conclusion remain somewhat more definitive than the careful interpretation presented throughout the revised manuscript. Slightly more cautious wording would improve consistency with the observational and

exploratory nature of the human findings. Second, the statement that only statistically significant biomarker correlations are illustrated should be revised or accompanied by confirmation that all tested correlations are available in the Source Data or Supplementary Information.

These are minor points that do not require additional experiments. Overall, the authors have responded constructively and have produced a substantially stronger manuscript.

Reviewer #3

(Remarks to the Author)
I would like to thank the authors for this extensive resubmission: this was very informative. I have no further concerns.

Open Access This Peer Review File is licensed under a Creative Commons Attribution 4.0 International License, which permits use, sharing, adaptation, distribution and reproduction in any medium or format, as long as you give appropriate credit to the original author(s) and the source, provide a link to the Creative Commons license, and indicate if changes were made.
In cases where reviewers are anonymous, credit should be given to 'Anonymous Referee' and the source.
The images or other third party material in this Peer Review File are included in the article's Creative Commons license, unless indicated otherwise in a credit line to the material. If material is not included in the article's Creative Commons license and your intended use is not permitted by statutory regulation or exceeds the permitted use, you will need to obtain permission directly from the copyright holder.
To view a copy of this license, visit https://creativecommons.org/licenses/by/4.0/

Answers to reviewers:

Reviewer #1

This paper investigated NO-mediated vibration modulation to brain glymphatic system based on human studies. Effects of the vibration modulation by NO, WBV, and NO_WBV were compared and discussed. Results showed a synergistic enhancement of brain fluid transport, and markedly increased the efflux of soluble proteins into the bloodstream. This paper is well-written and the vibration modulation of the brain glymphatic system is promising for clinical translation in the intervention of neurodegenerative diseases. I only have a few suggestions for improvement of the manuscript:

1.**It is interesting to see the brain volume decreased and the CSF volume increased significantly as shown in Fig. 1g-h. Does this mean WBV can produce physical changes of the brain?**

A: We cordially thank the reviewer for pointing this out; indeed we originally had some explanations but due to restrictions on word count we had to leave them out.

The observed changes should not be interpreted as evidence that WBV induces permanent structural or physical alterations of the brain. Rather, our data suggest transient physiological changes in tissue fluid compartment distributions via vascular dynamics.

Importantly, WBV alone does not produce the marked changes shown in Fig. 1g–h. The effects of WBV alone are presented in Fig. 4, where only modest changes are observed, including significant alterations in selected regional CSF measures (cavernous sinus dura and total brain CSF in the T2-derived fluid (T2DF) signal). The NIRS measurements further indicate a vasoconstrictive trend, reflected by reductions in HbO, HbR, and HbT, accompanied by an increase in the cortical water (H₂O) signal.

The more pronounced findings in Fig. 1g–h are observed following the combined NO+WBV intervention. Under this condition, the total brain volume remains unchanged, whereas the tissue T2DF signal decreases, suggesting a physiological redistribution of water and/or protein content rather than tissue loss. At the same time, segmented CSF volumes increase significantly, and the changes in CSF signal intensity show an age-dependent correlation. Taken together, these findings are more consistent with transient shifts in fluid compartments and vascular physiology than with permanent structural changes of the brain.

Some explanations would be helpful?

A: Our previous research on animal models lead to the development of vasomechanic human intervention[1,2] based on the observation of vasodilation leading into injected CSF tracer efflux. The NO vasodilation alone also does remove fluid from the brain, as measured with both T2DF MRI and hemodilution of total blood cell counts. After adding of the WBV increases the effect becomes into a significantly larger over most of the brain volume based on T2DF MRI data.

In the combined NO+WBV we could detect marked changes in the composition of the brain, CSF and venous tissues detected with three independent measures, i.e. Simoa laboratory kit, MR images and fNIRS sensing brain neurofluidics. These simultaneously quantified measures all point out to the same direction that indeed brain loses some of its water content along with proteins into blood directly. Importantly, the hemodilution should reduce the protein amounts but instead it increases Ab and GFAP found in CSF.

We believe that the NO introduces the vasodilation due to arterial smooth muscle relaxation by elevating cGMP in smooth muscle and by competitively stopping cytochrome C function and mitochondrial respiration chain causing arterial vasodilation.

First of all, the mechanotransductive slow stimulus has also been shown to increase lymphatic vessel constriction in the paraspinal areas increasing also glymphatic solute efflux[3].

The WBV activates mechantransductive piezo-1 receptors causing cation inflow into endothelial cells, astrocytes and neurons, having opposing effects on cognition in astrocytes vs. endothelial cells. Tonic medical stimulation of piezo-1 receptors of endothelial cells in mice reduces cognitive capabilities[4] but favourable in astrocytes[5]. Thus the modulated oscillatory WBV activation was preferred as it also mimics the main slow vasomotor drive of g/lymphatic efflux, as explained later on in question 4.

Our JPND Good Wibes consortia research has shown that the infraslow vasomechanic pulsations increase during sleep[6–9] and are a major determinant of the brain solute tracer efflux even down to urine in bladder level. Vasomotion of the neck of the lymphatic vessels increase g/lymphatic efflux [3]. Furthermore, once the head trauma-pain induced sympathetic tone increase was countered with the anti-adrenergic PPA medication, it restarted neck lymphatic vessel vasomotion improving significantly the brain edema removal, neurocognitive performance and recovery after brain trauma in mice[10].

The NO itself induces two changes for the brain neurofluidics: the water and blood perfusion pressure towards the brain interstitium reduces, and, also the intracranial pressure increases as blood volume (that usually is under autoregulatory control of smooth muscle of the arteries and arterioles) increases during NO vasodilation. Thus, the normal net flow of water towards the brain tissue reduces and the dropping blood pressure increases vasomotor wave amplitude and reduces frequency[11,12] ,manuscript Figure 3 a-b. After the NO vasodilation wears off at 10-15 minutes or so, the slowly modulated WBV starts to cause gentle vasoconstrictive waves, which increases the NO hydrodynamic effect 2-3 x wider, c.f. Fig 5 B now on the new version.

This in our mind leads to two things: i) reduced arterial blood water pressure towards the perivascular space and astrocytes (and maybe interstitium also) added to increase in compressive ICP from vasodilation during NO leads to the measured blood pressure dependent removal of proteins into the blood along with the removed water out of the brain (I/)CSF. Notably also the perineural efflux can be increased but one might expect it to be slower than direct vascular route. But in both cases, the results suggest that there may be a blood pressure dependent mechanism(s) embedded within the BBB for solute efflux indeed as the reviewers here accurately point out. The lowered arterial pressure towards the tissue reduces together with the increased ICP from vasodilation may increase efflux of solutes and proteins start to leak out of the brain into CSF and/or venous blood flow.

The sagital sinus BBB (c.f. Fig 1 below from our electron microscopy figure from sinus sagital vacuoles[2], where we show our previous EM image indicating that in the reduced blood pressure case the amount of vacuoles outside the brain increases taking HRP tracer with it. Our results suggest that within the autoregulation boundaries the reduction of the BP enhances a pressure dependent mechanism of Ab removal.

REDACTED

Figure 1 illustrating the CSF to sagital sinus electron microscopy data indicating HRP entry into the blood brain barrier of the SS after vasodilative treatment [2]*(Jukkola Jari, Investigation of treatments to enhance cerebrospinal fluid flow in the brain : blood pressure lowering and whole-body vibration Thesis 2022 Fig 26, page 76,ISSN 2343-3868,* [*https://oulurepo.oulu.fi/handle/10024/10321*](https://oulurepo.oulu.fi/handle/10024/10321) *limited availability due to pre-journal publication thesis).*

Ii) secondly, a similar hydrodynamic pressure mechanism occurs in normal sleep, where also BP drops and ICP increases[13–15]. Our EU JPND consortia has just shown that in sleep the noradrenaline (NA) driven BP drop followed by slow oscillations in NA levels also increases vasomotor waves [6]. Regionally the increased vasomotor waves then start to drive local interstitial electrical activity and hydrodynamics in NREM 1-2 sleep based on synchronous MREG BOLD, infraslow dcEEG and water fNIRS measurements [7]. Sleep also speeds up water molecule flow through the brain tissue in the areas of increased vasomotor wave drive of electrofluidics[8]. The same increasing vasomotor waves drive brain electro/hydrodynamics has also been shown in animals there also the tracer removal increased from brain all the way into bladder within our JPND collaboration by Nedergaard's group[6,7].

Sleep also increases the other main brain pulsation mechanisms of respiratory pulsation power and speed[8,9,13–15]. There also unpublished research poster from our JPND consortia (submitted and under re-review) indicating that locus coeroleus noradrenaline controls also pH/respiratory activity modulating also respiratory brain pulsations (Oxford 2026 Glymphatics conference poster session). Similar to the sleep changes, the NO reduces BP and increases both vasomotor waves and respiratory brain pulsations markedly simulating a natural, sleep like biophysiological mechanism for increasing brain solute removal during the vasomechanic therapy.

Iii) Thirdly, the increased intracranial pressure causes more proteins to leave over the BBB, as shown here in the illustration supporting the idea of more optimal vacuoles for Ab removal in low BP, vs. reduced Ab removal in high BP[2].

Investigation of treatments to enhance cerebrospinal fluid flow in the brain : blood pressure lowering and whole-body vibration

iv) Due to the reviewer critique here, we also conducted more deeper pooled analysis of T2DF MRI data vs. venous blood ptotein concentrations. We detected that the *increasing* protein levels in the venous blood were in fact linearly correlated with both measured brain tissue (GFAP) and venous blood T2DF MRI intensity levels, c.f Fig 2 below.

The T2 DF MRI data reflects water bound to macromolecules like proteins while the free CSF and blood water is darkened by inverse TI pulse. Thus the blood serum and plasma Aβ40 & Aβ42 protein values both correlating with the venous df image intensity indicate an increased blood protein amount after the NO+WBV vasomechanic therapy directly into the veins.

Also the amount of GFAP in the blood was reversely correlate with the brain tissue image intensity indicating less astrocytic GFAP protein binding water in the brain in addition to the removal of water giving the MR signal in the tissue as well.

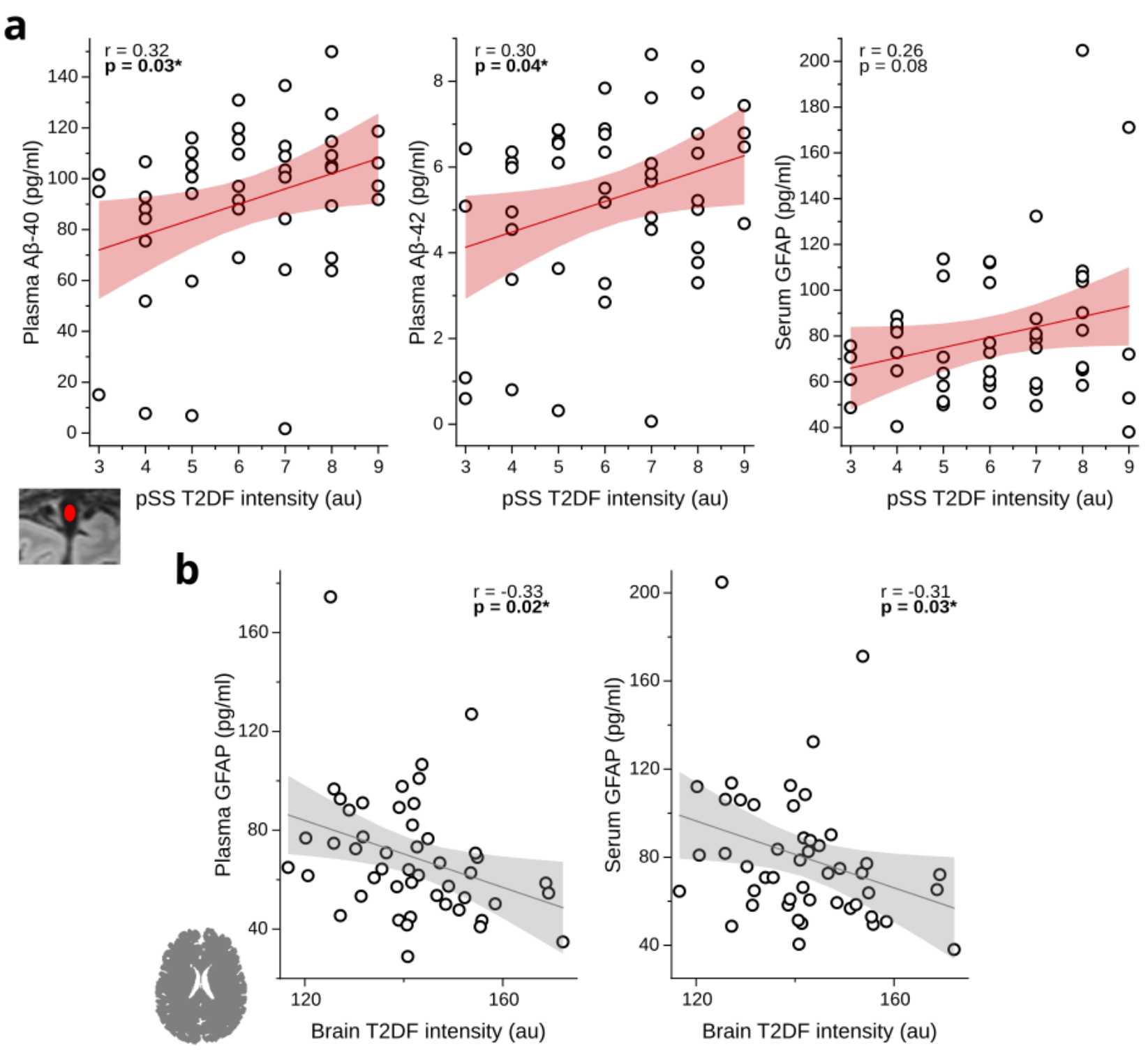

Fig .2. a) Pooled analysis combining baseline pre and post vasomechanic intervention data of Aβ 40 and Aβ 42 plasma concentrations from Simoa HDX lab measurements vs. T2DF MRI sagital sinus image intensity showing linear correlation with both proteins and a trend with GFAP. b) The brain derived GFAP protein plasma and serum concentrations both are inversely correlated to whole brain T2df signal intensity indicating that the whole brain protein/water relation shifts is coupled to the GFAP that is being moved into the bloodstream in significant amounts during the vasomechanic intervention. Notably, the plasma protein levels normally should be lowered by the NO induced hemodilution (Dupuis 1990), but in combined vasomechanic treament the opposite occurs.

We now added the following short explanation on the issue also in the combined result image figure 5 in addition to the beginning of the discussion:

*Illustrative diagrams of **e**. baseline and the main effects with postulated mechanisms on **f**. Piezo1-targeted whole-body vibration (WBV_p, Fig 4) induces mainly slowly emerging vasoconstriction elevating BP and increasing CSF with brighter DF signal indicating higher protein content. **g.** NO vasodilation (NO, Fig 3) drops BP, increases vasomotor waves, respiratory pulsations in a sleep like fashion (Helakari 2022,2023, Haugland 2025, Väyrynen 2026) and ICP(Jukkola et al.2024) resulting in removal of brain water along with proteins into CSF, PSD and venous blood relatively more from the gray matter (GM). Despite the known hemodilution after NO on blood counts(Dubois), the stable DF signal intensity within the veins indicates relatively elevatedlevels of protein in venous blood as hemodilution should lower the DF signal within veins. **h.** combined (NO+WBV_p Fig's 1-2) interventions induce in addition the solo NO and WBVp effects alone a longer lasting BP drop followed by slow BP increase. The brain neurofluidic redistributions occur over a markedly wider area especially in deeper white matter (WM) solute resulting in strong brain protein removal into posterior PSD and venous sinuses. Notably, the a linear correlations of measured blood Ab40 and Ab42 concentrations in Fig 1 m with the posterior venous sinuses increasing DF signal despite the NO hemodilution indicates simultaneous laboratory and imaging evidence strongly suggesting of increase in posterior venous protein concentration as compared to NO intervention alone.*

Also :

Agonist drugs also cause tonic activation of endothelial Piezo1, which induces a long-lasting vasoconstriction that attenuates the important vasodilation responses coupled to neuronal activity leading to cognitive decline in mice[31]. Thus, slowly modulated Piezo1 stimulation was used to induce oscillatory vasoconstrictions, similar to vasomotor waves recently shown to be directly proportional to the amount of brain solute efflux (Haugeland 2025).

Could you please explain the increase and decrease patterns of the intensity at different locations in Fig. 1 k-m? If efflux was enhanced, should they be all increased in terms of signal intensity?

A: Thank you for pointing this out, this is a key point raised by all reviewers, that we were unable to explain in relevantly accurate form within the word limits indeed. But actually now with the reviewer motivation and quite frankly help on the the issue we were able to find references that could help in explaining the anatomical differences. Also as this is a novel finding, we may not be currently able to fully explain it alltogether, we can only speculate why these results are quite localized. But basically the solute efflux seems to be occuring more int he dorsal venous areas situated at the lowest areas of the brain in supine position of the head while laying on the MRI scanner. And no changes were detectable in the CSF ventricles in the middle of the brain that suggest a differential efflux mechanism altogether in the cortex vs. deep down.

From the MR imaging perspective the T2 DF MRI has to be interpreted always in relation to the tissue compartment at hand; the free unbound water is dark due to precisely targeted T1pulse some 1800 ms after the rf pulse, while water molecules close to macromolecules including proteins in the brain tissue are bright. Usually pathology increases the brain tissue dfMRI brightness due to water influx in edema and/or gliosis.

In our data the increase in the otherwise dark fluid signal in areas of free water signal in neurofluidic areas like venous blood and CSF, means either an increase in protein amount, but also a reduction in water concentration in fluid spaces could (in theory) be the case. More precisely the relative amount of protein/water increases when fluid signals get brighter. And vice versa, when the CSF becomes darker, the less protein there is per water molecule. After NO, the blood water amount increases also as shown by the hemodilution effect detected by Arend and colleagues [16], re-analyzed and shown also in our data in Fig 3 l.

For the brain tissue filled with molecule bound bright water, a darkening usually in clinical imaging means a reduction in brain tissue edema water after successful therapy. In our study the both the

NO and combined NO+WBV vasomechanic intervention especially remove molecule bound tissue water. However, as it is a protein/water relation that governs T2DF MRI intensity in tissue, also a reduction in brain protein like the brain specific GFAP (and Ab) can also explain the signal reduction. And in our case the removed GFAP levels in plasma and serum also correlated with whole brain 2TDF intensity.

All this was already shown excellently in the Albayram [17] paper phantom experiment:

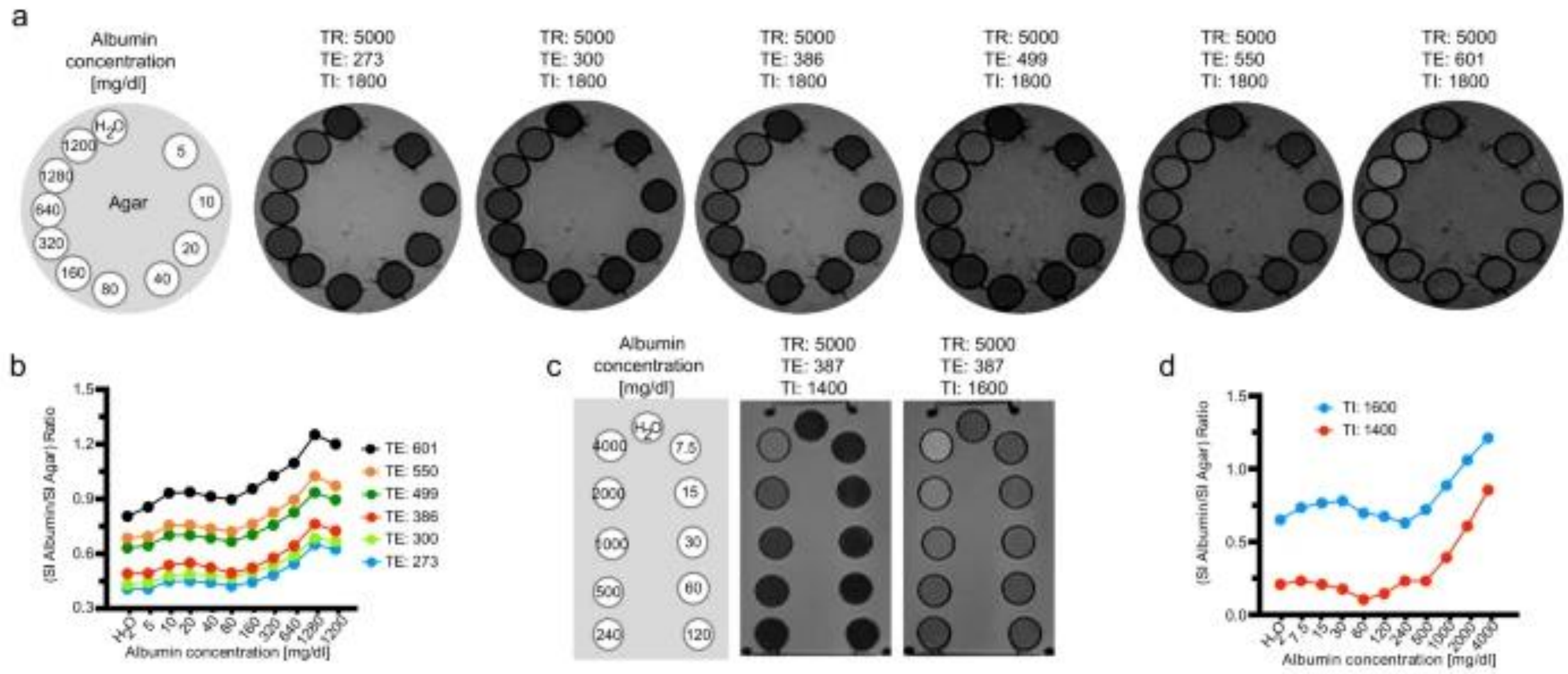

Figure 3. Ablayram protein concentration phantom study indicating how the amount of protein determines the T2DF intensity. Importantly, we used identical T2DF MRI sequence parameters (TR5000, TE 386, TI 1800 ms)[17], which verifies that the protein concentration in the fluids dictates the results also in our studies together with the verification of in vivo protein correlation to the T2DF MR image intensity above in Fig 2.

However, there are indeed changes that are quite localized and thanks to the reviewers comment we did find that there are some references of spatial differences at least for basal vs. dorsal meningeal lymphatatics in mice[18]. In the central CSF areas like ventricles do not seem to have any significant T2DF change in the CSF spaces, except for the deep basal pericavernous dura in the combined NO+WBV vasomechanic intervention and during the WBV alone.

Another mechanism could be small differences head alignment within paired intra-individual comparisons but the lack of rim like changes at the tissue/CSF/gas interfaces typical to MRI data misalignment, opposite sham experiment results and multiple compartments like intensity based ventricles as well as hand drawn neck lymph nodes showing no changes in tissue T2DF signal suggests that the detected results are not explained by small differences in head orientation nor technological but induced are by the interventions.

The grand total efflux seems to be more of a cortical phenomenon suggesting that it could be a more glymphatic phenomenon, or, the deeper structures around CSF may behave differently as suggested by Ahn & co-workers based on mice experiments[18]. The changes are also localized more in posterior venous sinuses like sagital and rectus sinus and posterior parasagital dura, while more anterior structures like middline sagital sinus had no change. The parasagittal dural PSD T2DF signal intensity was dropping – and as now also found out by the deepended investigtions after the reviewer comments- these changes seem to persist over months (9.4 +/-   months) as compared to original non-intervention baselines.

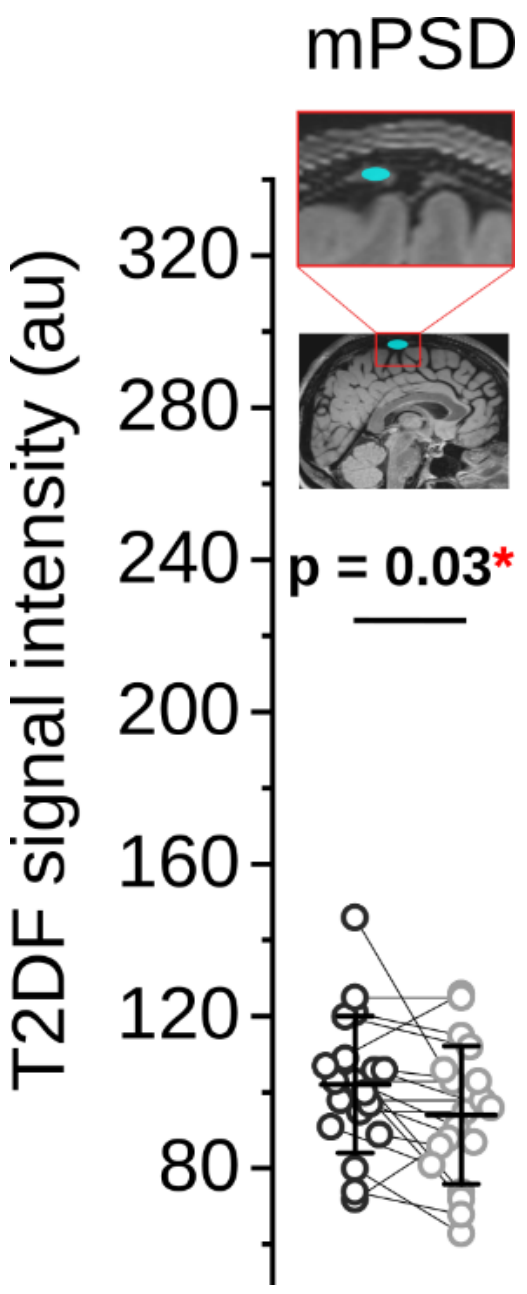

Figure 4.

One explanation to the findings could be the results detected by Eide and Ringstad, who showed that that the slow tracer accumulations may be focused more in the posterior areas[19,20] along with increasing cortical vein inflow towards the back. In supine position there could also be some hydrostatic sedimentation issue together with the idea that somehow the clearance is faster/stronger in front and changes are detected in the posterior areas, the dura could be more permeable at the back. The protein shift along PSD could be somehow compartmentalized towards the back of the brain Møllgård SLYM membrane also as suggested by Nedergaard[21].

Now added explanation:

“We used the dark-fluid (FLAIR-based) MRI signal intensity analysis implicated earlier by other groups for investigating brain solute transport within glymphatic PSD interfaces[22,23,58]. T2DF is a very important sequence in clinical neuroimaging, where tissue-bound water has high signal intensity due to proximity of water ^{1}H proximity to macromolecules like proteins. Less constricted water molecules distant from macromolecules in neurofluidic compartments such as blood and CSF are labelled dark in T2DF MRI by a precisely timed inversion pulse rendering freely precessing water ^{1}H signal dark.

In clinical imaging an acutely increased T2DF signal is often related to arising from brain tissue edema and darkening of T2DF signal typically reflects a reversal of such pathology after a successful therapeutic interventions. The current T2DF MRI results need to the interpreted differentially based on the brain compartment at hand: while in tissue the darkening may reflect mostly reduced amount of bound water ^{1}H signal, while an increase in normally low signal neurofluidic compartments T2DF suggests increased amount of water molecules close to macromolecules such as proteins.

As shown by a previous protein phantom model verification study with a similar 3T scanner using identical TR, TI and TE sequence parameters, the measured T2DF darkening of reflects reduced protein/water ratio and vice versa with increasing signal in free water solutions[23]. One of the most prominent findings of the vasomechanic intervention was a whole-brain darkening of T2DF MRI signal extending into deep structures (Fig. 1f, 3f). Here, darkening of whole human T2DF image may in theory reflect either reduced amount of tissue bound water resulting in less ^{1}H protons to

give MR signal, reduced amount of soluble proteins affecting the watermolecules less, or actually both. In light of the quantified hemodilution, an increase in soluble protein level increase in the blood laboratory results, in addition to the convergent fNIRS hydrodynamic brain sensing results, the most likely change in the brain is a combined redistribution of soluble proteins along with removed brain water, i.e. solutes, from the CSF compartment (Fig. 1f-h, 3h) into blood constituents (Fig 3l).

As normally the NO-induced hemodilution causes a drop in blood protein concentration (Dupuis et al. 1990), the opposing increase we detected in low molecular weight soluble brain derived proteins (< 68 kDa) in blood (Fig. 1d) strongly suggests an increased brain-derived solute efflux directly into the blood. This may well be accompanied with transient BBB related permeability effect, as also seen in the mouse model (Fig. S28b). The dcEEG level that reflects BBB permeability further suggests that BBB permeability could be increased (Kiviniemi et al., 2017 Plos One). It is also of particular interest that two posterior venous sinus T2DF signal intensities showed a linear correlation with quantified circulating blood protein concentrations (Fig. 1m), linking the imaging findings more directly to blood biomarkers, suggesting a more posterior compartmentalized efflux mechanism in humans.

The low intensity DF compartments of posterior venous sinuses and adjacent PSD regions showed increased DF intensity following the vasomechanic intervention, suggesting the idea of enriched local protein concentration increasing the DF signal, as indicated by Albayram et al. in the PSD spaces(2022) (Fig. 1k-l) (Albayram et al. 2022). In contrast, the more frontally in the medial sagittal sinus and parasagittal/para-cavernous dural CSF regions displayed reduced, i.e. an even darker T2DF signal, consistent with simultaneous water dilution suggestive of either increased protein removal or increased dilution of CSF water (Fig. 1k, 4k).

To the best of our knowledge, this detection of differential solute efflux based on cranial position in humans is a novel finding and thus currently we do not have direct knowledge on the structural mechanical underpinnings of such differences in humans. There could be anatomical and cerebrofluidic pressure-dependent differences in the BBB and PSD permeability favouring dorsal area efflux. Mice have been shown to have differential basal meningeal lymphatic vessel structure and valves function that allows increased tracer efflux compared to dorsal meningeal vessels (Ahn et. al. 2019). In the supine position there is also more hydrostatic pressure towards the posterior areas, which, together with the increased ICP during arterial vasodilation (Fig's 1 & 3) could in compartmentalized subarachnoid space CSF favor posterior solute drainage towards the posterior venous sinuses in human cranium (Eide et al. Nat Com 2020; 2024, Møllgård et al., 2023).

Furthermore, the middle vertex PSD CSF-filled space showed quite a persistent DF signal alteration, with a mean interval between measurements of 9.4 months (range 0.5–22.3 months, Fig. S23), although majority of the T2DF signal intensity changes were transient over most parts of the brain. This seems to be in line with the notion that these vertical areas may have slowest solute in/outflow processes as already suggested by Eide and co-workers in humans(Eide&Ringstad 2020). Here we show that once there is a change in the PSD solute content, the changes in these areas also seem to persist for months. This slow nature of solute exchange could be partially related to hydrostatic effects in the low pressure environment of venous drainage. Even in recumbent positions hydrostatic pressure of the vertical sagittal sinus is relatively elevated compared to lower venous sinuses, which in theory should direct the pressure driven solute flow towards be posterior areas as shown predominantly to occur in Fig's 1 & 3.

In theory, the T2DF changes could be due to technical Rician noise in low-intensity T2DF signal areas due to altered positioning between scans or T2 flow differences between scans. However, such noise artifacts should be present uniformly in a given area of the T2DF image. Here, however, the T2DF changes were highly compartment specific, and not present uniformly in the brain as

should be in the Rician noise case. Neighboring supracellar cistern as well as other central CSF low intensity CSF areas show no changes, while adjacent sinus rectus dark fluid signal shows increases that are linearly correlating with the venous blood both Aβ's, which argues against the Rician noise artefact possibility (Fig, 1h&l, 3h, 4i, S7, S15, S17, S19, S21).

The increased CSF flow speed can also increase T2 DF signal intensity in narrowed CSF areas. However, most global brain changes indicate darkening of T2DF signal. The T2FD signal intensity increases in posterior venous sinuses and PSD areas after vasomechanic intervation do not overlap with any MREG pulsation power increases. We have previously shown that an increase in brain water flow speeds should coincide with simultaneous power increase in the brain tissue and CSF (Elabasy et al., 2025, Järvelä 2025, Väyrynen 2026). During the NO vasodilation the MREG power of especially vasomotor and respiratory pulsations was increased in wide areas; however in these areas the T2DF signal should increase instead of the detected darkening if the signal changes were due to increased flow. After the combined intervention, the pulsation power changes were mostly gone, and thus the T2DF signal increase posterior sinuses and other areas as well sound to be more related to increased blood protein content rather than flow speed increase, c.f Fig 1 m."

3.Is it possible that the significant intensity decreases of the CSF space for the WBV as shown in Fig. 4k may due to the down modulation of the blood flow?

A: Indeed the flow of water molecules in T2DF MRI can sometimes increase signal intensity in narrow sulci due to fast flow, in theory also reduced flow could be involved. However, these changes are rarely reported in the clinical literature and are not routinely detected in clinical practice. However, the segmented CSF spaces in the whole brain had increased intensity in 4 l-image, only the parasagital dura PSD in 4k-image showed a drop.

Regarding the flow drop, at least MREG pulsation powers just prior to the T2DF MRI scans were not altered in these areas as compared to baseline suggesting minimal flow changes first of all. Also there should not be much flow changes anymore at the time of the 4K figure as it was taken a minutes after the WBV therapy, and importantly the MREG data taken just before shows no significant changes in pulsations at least. We also tried to avoid blood vessels in the DF MRI using 3D T1w and T2 spc images as not to image blood flow but rather the dura there in the cavernous dura based on avoiding carotid arteries and by minimizing the apparent venous effects that indeed are present in the cavernous sinus. However, it has to be said that absolute certainty from separating cavernous sinus dura vs. venous ROI gathering may be difficult to obtain due to both imaging technique and anatomical reasons. Even if we had used i.v. Gd enhanced techniques, the dura would also enhance making it virtually impossible to separate from cavernous sinus. Also recent 4D PC venograms may not reach this level of spatial accuracy. We do not such 7T phase-imaging or other techniques that could differentiate venous sinuses by their very slow flow profiles.

So in theory a downregulation of the venous flow could be the involved but the lack of pulsation power drop talks against it and usually df image data only shows increased signal intensity due to faster flow. We have previously detected increased water flow speeds during power increases in human brain, suggesting that the DF signal should increase rather than darken in the brain. The absent signs of flow speed increases in venous sinuses also talk in favour of protein concentration increase in them instead of flow speed.

Now added text:

The increased CSF flow speed can also increase T2 DF signal intensity in narrowed CSF areas. However, most global brain changes indicate darkening of T2DF signal. The T2FD signal intensity increases in posterior venous sinuses and PSD areas after vasomechanic intervation do not overlap with any MREG pulsation power increases. We have previously shown that an increase in brain water flow speeds should coincide with simultaneous power increase in the brain tissue and CSF (Elabasy et al., 2025, Järvelä 2025, Väyrynen 2026). During the NO vasodilation the MREG power of especially vasomotor and respiratory pulsations was increased in wide areas; however in these areas the T2DF signal should increase instead of the detected darkening if the signal changes were due to increased flow. After the combined intervention, the pulsation power changes were mostly gone, and thus the T2DF signal increase posterior sinuses and other areas as well sound to be more related to increased blood protein content rather than flow speed increase, c.f Fig 1 m.

4. The wide frequency range for vibration stimulation overtime as shown in Fig. 4c is a bit complex. It will be ideal to have single frequency analysis. Since it's a pilot human study, some discussion would be helpful as there are many frequency-specific studies showing the modulation effects as mentioned in the introduction section.

A: This is indeed the case, but the wide range and modulation of the WBV is deliberate and based on blood pressure driven research over a decade from the Neurosonic company pioneer. We chose to use a modulated stimulus of different frequencies, that was found have beneficial BP in multiple subjects on trial based testing by the manufacturer (currently non-published information). The developer of the Neurosonic WBV tool finetuned the tool development focusing on BP and individual feedback measures.

But the reviewer is absolutely correct that these issues need to studied in a spectral manner, as, for instance, the piezo-1 receptor alone response to vibration works as follows:

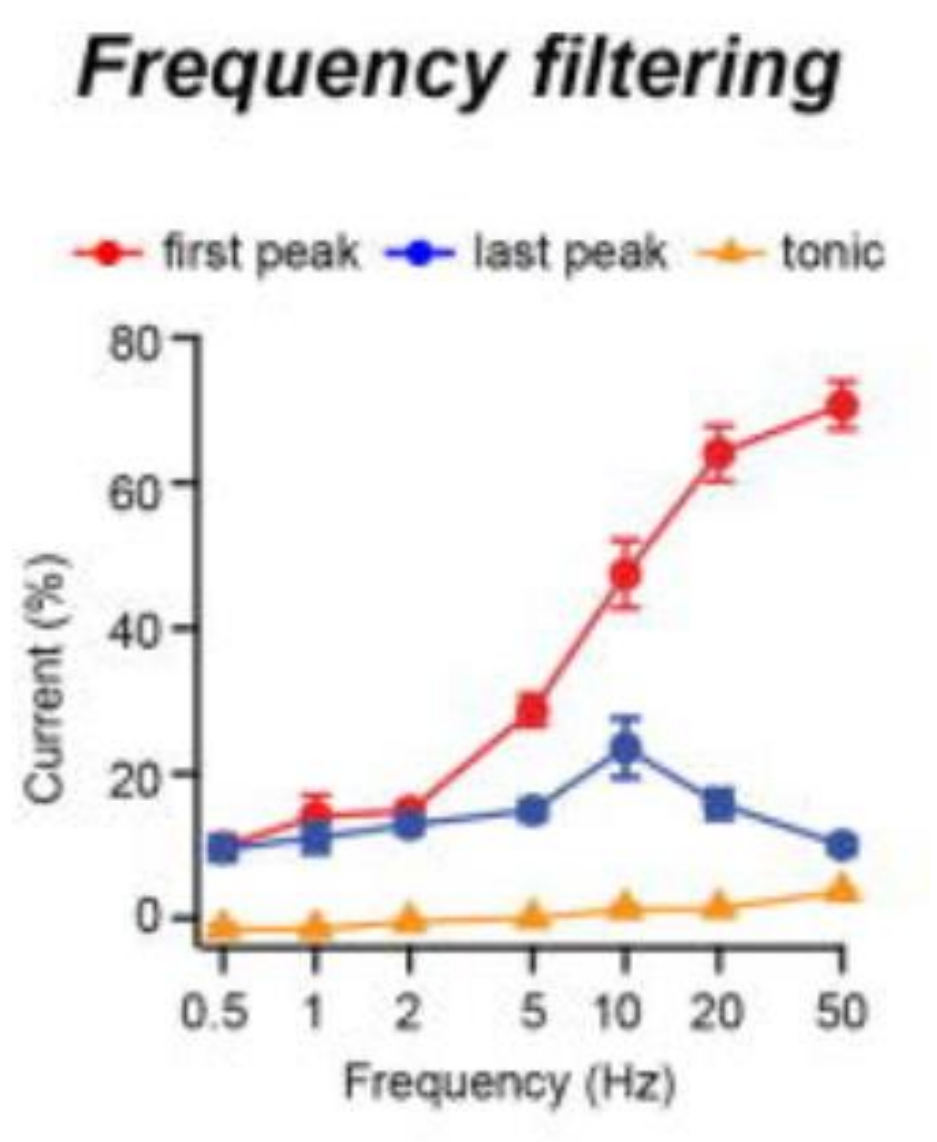

Figure 5. Efffect of tonic and first onset mechanotransductive vibration on Piezo-1 receptor by Lewis et al., 2017 with elevating with increasing frequency on first response.

As one can see Lewis & co-workers excellent article [22]image above that indeed the 1st peak stimulation response to a frequency continues to grow clearly till 50 Hz, so there is no preferred frequency actually, even though the 40 Hz has become a popular one for some reason, maybe due to gamma range upper neuronal limit? What is also noteworthy is the tonic stimulation yellow line; it does not do much activation of the piezo-1, therefore we modulated both the frequency and

amplitude to get to 1st peak activations as often as possible. Also continuous Piezo-1 stimulation of endothelial cells by Yoda1 medication is harmful for mice cognition.

The modulation is actually the very point of the therapy; we used a single frequency in our previous animal studies where we did not see much results, only some in the dura. Furthermore, the modulation of both the amplitude and and frequency could increase the piezo-1 receptor targeting due to avoidance of receptor habituation. In addition, as explained above in question 1, the slow modulation of piezo-1 stimulus was deliberately used to mimic vasomotor waves to maximize natural pulsatile efflux mechanisms that are shown to be most effective drivers of g/lymphatic efflux [3,6,7,10].

However, we definitely agree with the reviewer that we need to look more closely to what happens DURING each frequency envelope and modulation on the dcEEG and NIRS data available on EACH WBV treatment. And indeed, that is work we have already started looking at and indeed there are some quite nice preliminary findings that we intend to analyze further and hopefully publish after this article. But there is so much work there that falls out of the scope of the current study.

Also we chose not to stimulate only the head&neck areas as many others do, but rather the whole body, which proved out to be a very good idea based on what Steven Proulx said on 2026 Oxford Glymphatics meeting that the actually the whole CNS, also the peripheral nerves seem to take part in the neurofluidic flow efflux along all peripheral spinal nerves, not just cranial nerves[23] by Rzeplinski et al. This may also increase the efflux in the peripheral areas, but it may not explaing the relatively fast increase in Ab & GFAP plasma measures.

5.The mouse experiment showed promising results with 40 Hz. To be consistent with the methods used for human in this study, would it be helpful to have the same WBV application to the mouse to have a comparison?

A: In our translational experience the mouse results were not directly translatable to human results due to some differences that we do not yet fully understand. While humans responded very clearly to the combined vasomechanic stimuli, the mice responded to the WBV stimuli mostly on the dural areas. However, the animals were in anesthesia so that is an obvious difference. So using the animal data may not be directly translatable here.

But indeed, as mentioned above, we aim to look at the different frequency bands separately on humans in our next study; also their order and modulation should be investigated more thoroughly in the future. And how other stimuli like NO effect these results. But all in all, the more variable stimulation seems to be beneficial for the solute efflux. And as there are other mechanoreceptors like TRP, etc.., these frequencies should indeed be more carefully examined over a spectrum of them really. Literature on these interactions at receptor specific level is sparce currently.

6.Signal intensity of the T2DF images were used for evaluation of the efflux. There are many MR imaging modalities that can be used for quantification, e.g. ASL, DTI. This can be considered in future studies.

A: Absolutely so; and it is very easy agree with reviewer also here since we just applied for an EU BrainHealth Partnership grant together with the leading glymphatic imaging researchers including diffusivity measure CSF-STREAM and elastography DENSE, MREG and human golden standard Gd+ MRI Oslo pioneers to do vendor agnostic approaches on scanning human brain neurofluidics.

Also planning to scan proteins with CEST, MRF T1 &T2 maps in addition to ASL and DTI. Maybe SWI and MR spectroscopy too if time and patients/volunteers allow. We have direct plans now with partial funding to commence GdMRI research with Oslo researchers on MREG verification analytics within the Lundbeck funded Nordic Center for Glymphatic Biology.

But in this article we do have here quite a bit of multimodal results as pointed out by the second reviewers here. But absolutely this is indeed the plan for the future, would love to join these masterclass researchers.

Reviewer #2

This is a substantial and timely study that addresses an important question in human brain clearance physiology through an ambitious multimodal design integrating physiological monitoring, blood biomarkers, and MRI based readouts. The human dataset is valuable, the overall experimental concept is compelling, and several aspects of the work are potentially important for the broader translational literature in neurodegeneration and brain fluid biology. At the same time, a number of conceptual, methodological, and reporting issues should be addressed before the principal conclusions can be supported at the level currently stated. In particular, the manuscript would benefit from a more disciplined alignment between the data and the mechanistic claims, together with several clarifications that would improve analytical transparency and overall interpretability.

Major Comments:

1. The manuscript presents interesting and potentially important evidence that the combined intervention is associated with increased circulating Aβ40, Aβ42, and GFAP together with imaging changes consistent with altered neurofluid dynamics. However, the central interpretation currently extends beyond what the data can firmly support, particularly where these findings are discussed as evidence of direct enhancement of brain to blood protein clearance. The distinction between association and mechanism should be drawn more carefully throughout the Results and Discussion. In particular, the authors should consider alternative explanations for the observed biomarker shifts, including systemic redistribution, transient barrier related effects, and other hemodynamic influences associated with the intervention. As presented, the dataset is clearly suggestive, but the strongest causal language should be moderated unless supported by additional orthogonal evidence.

A: We thank the reviewer for pointing out this very important and quite helpful point indeed; we were indeed a bit too much carried away with our interpretations and expressions based on the quite astonishing results. Now we tone down the discussion considerably over the causal relationships indeed and try to be holding more on solid evidence ground. As there is an absence of a method directly scanning brain tissue protein waste removal in humans, we can only speculate in some of the issues absolutely; thus we indeed toned down our causality speculations.

On the other hand with the helping guidance of the reviewer comments, we were also able to find out more orthogonal evidence on the brain tissue compartment T2DF signal relation with serum and plasma protein relations as shown here in the response and in the new text that was edited quite extensively.

We introduce now the mentioned idea of systemic redistribution based on the latest findings of Steven Proulx on Oxford meeting of the whole CNS redistributive involving the peripheral nerves as well in the CSF efflux into discussion[23].

We go on also introducing the suggested idea of transient barrier effects at the end of the vasomechanic chapter as we have recently shown that indeed the BBB in sleep alters regionally and seems to reverse neuro and hydrovascular coupling during sleep in order to flush the brain interstititium[7] as the astrocytes lining the BBB glia limitans with their endfeet do actually shrink by noradrenaline[24]. opening up the outer glia limitans BBB barrier most likely.

Now added: "Notably, the combined treatment evoked a temporary H_2O response distinct from that with WBV_p alone; while WBV_p-alone caused a continuous rise in H_2O accompanied by

increased MAP (Fig. 4g), the combined NO+WBV_p intervention produced only a transient decrease in H_2O, with subsequent recovery."

However, the NO alone induced a more longlasting H2O change and furthermore the new analysis idea from the reviewers also helped us to detect also a long lasting, on average 9 months, PSD T2DF signal change over time. So not all changes are that temporary but have prolonged effect on slow protein/water reflecting DF signals,.

This actually helps to join in much previous literature on blood pressure and related damage as a preventable risk factor in neurodegenerative diseases, vascular damage explaining also why rigorous BP control prevents Alzheimer conversion.

Now added in Discussion: "Mechanistically, the combined vasomechanic intervention produced a spatially wide and age-dependent reorganization of neurofluid dynamics, reflected as coordinated changes in global brain tissue, CSF, and posterior PSD and venous sinus signals on dark-fluid MRI, which tracked both hemo and hydrodynamic signatures detected through simultaneous NIRS monitoring.

.. recruits complementary vascular and CSF transport mechanisms that seem to be capable of enhancing human brain solute clearance, corroborating previous research findings."

Furthermore, based on the quite insightful comments of the reviewers, we looked at the data more deeply and discovered that actually both dark fluid MRI intensity (reflecting the level of water bound by protein) of the posterior venous sinues correlated linearly with the venous blood both Aβ40 & Aβ42. Moreover, the even more clearly increasing plasma GFAP levels correlated inversely with the brain tissue intensity. So the more there is protein in the blood (increasing after the intervention), the brighter the venous sinus blood is due to increasing amount of protein affecting the water molecule spins. This indicates that the detected T2DF MRI signal is indeed reflecting venous blood protein levels and based on increasing values also the removal of brain tissue proteins to both CSF and blood.

We are unable to explain these correlating findings in a any other way than direct posterior sinus efflux from CSF into the posterior sinuses since such changes are unobservable in the anterior venous sinuses. Also T2DF signal in the parasagital dural space (that do not exist in small mammals like mice) seems to be affected in a way that actually last at least for on average 9 months.

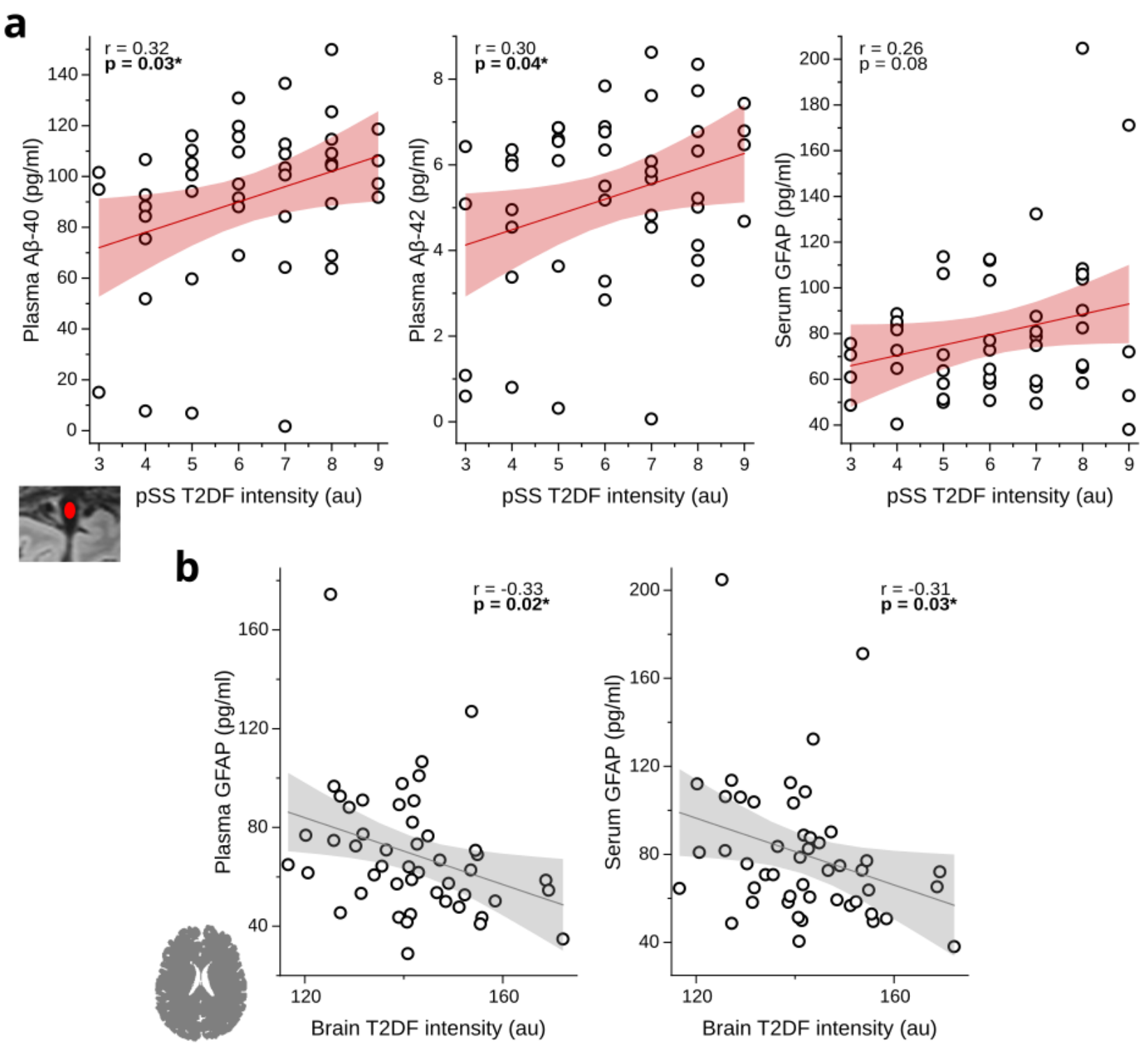

Figure.6 (i.e. Fig 2 shown here again for faster reference) a) Pooled analysis combining baseline pre and post vasomechanic intervention data of Aβ 40 and Aβ 42 plasma concentrations from Simoa HDX lab measurements vs. T2DF MRI sagital sinus image intensity showing linear correlation with both proteins and a trend with GFAP. b) The brain derived GFAP protein plasma and serum concentrations both are inversely correlated to whole brain T2df signal intensity indicating that the whole brain protein/water relation shifts is coupled to the GFAP that is being moved into the bloodstream in significant amounts during the vasomechanic intervention. Notably, the plasma protein levels normally should be lowered by the NO induced hemodilution (Dupuis et al., 1990), but in combined vasomechanic treatment the opposite occurs.

However, the reviewer is indeed correct (especially given the new information on CNS efflux areas by that the efflux can very well take multiple routes as it was uttered in the Oxford Glymphatics meeting on 2026; one route directly into blood and one of peripheral CSF efflux through perineurium all around the body[23]. Personal communication with Jeff Tichoff who is one of the leading solute flow modelling physicists in the same Oxford meeting indicates that such efflux routes can be about 50:50 between blood circulation and peripheral nerves in rodents at least. On the other hand, the Oslo human research pioneer Rignstad with Melin indicate that already 2/3 of Gd+ MRI tracer becomes removed in the spinal canal. Thus the whole body wibration with the Rzeplinski intra-neural solute effux in mind may serve a purpose worth looking into more deeply.

However, one might suspect that the CSF to peripheral nerve efflux route should be slow path and not cause major changes in 40 minutes especially in a person not moving and laying down. As the brain CSF has 80-100 x more $A\beta_{42}$ in the CSF than in blood in healthy controls, as seen from the measures below in Fig 7, it makes the brain CSF most likely source of $A\beta_{42}$ in our healthy controls rather than any peripheral system as in normal circumstances the liver usually degrades Ab from blood almost totally, as can be seem from the low levels (< 13 pg/ml, vs. CSF > 1000 pg(ml))in both cohorts of normal healthy control below in Fig. 7.

REDACTED

Table 1. Copied table from the article[25] Thijssen et al., Differential diagnostic performance of a panel of plasma biomarkers for different types of dementia,2022 Diagn Assess, DOI: 10.1002/dad2.12285 indicating both the Simoa HDX sensitivity vs. Mass spectrometry and also the marked difference in CSF vs. Plasma levels in normal controls in addition to AD, FTD and DLB patients.

2. The dark fluid MRI analysis is central to the interpretation of the study and directly informs the broader conclusions regarding clearance related physiology and brain protein redistribution. In this manuscript, the imaging findings serve not simply as supportive observations, but as the principal framework through which the post intervention biomarker changes are linked to altered fluid and macromolecular behavior within the brain and adjacent dural compartments. This aspect of the work therefore warrants a more fully developed methodological and conceptual discussion.

A: This is absolutely so as also the 1st reviewer pointed out; the previous version did not have enough commentary on this very subject. Now we added more in depth descriptions backed up by the additional analyses we did.

The use of a high resolution non contrast dark fluid, FLAIR type acquisition is an important strength, particularly in the context of human studies where repeated assessment may be required. At the same time, the interpretive scope of these signal changes should be defined with greater precision. Specifically, the Discussion would benefit from a more explicit account of how the observed dark fluid alterations should be understood in relation to changes in water and macromolecular distribution, rather than being carried too quickly toward route level mechanistic inference. Strengthening this section would materially

improve the manuscript by aligning the imaging results more closely with the biological claims being made and by clarifying the broader relevance of this MRI framework to the study of human brain clearance physiology.

A: We thank both reviewers for focusing on this key point: we did not explain all the issues in detailed way in the main text and now as we also found out that the venous blood T2DF MRI signal intensity and Aβ concentration are directly correlative, as it should be based on the DF/FLAIR image contrast mechanism, we add more in depth results and discussion on the matter.

We now do through the basics of the df image signal production principles and explain the detected results of sagittal sinus, PSD and brain tissue and their direct correlation to the quantified blood protein levels also referencing the in depth phantom experiment by Albayram & co-workers[17] here in reviewer response Fig 3 showing how protein concentration in a water phantom alters the the signal intensity; a relative increase in protein increases and a decrease drops the signal intensity.

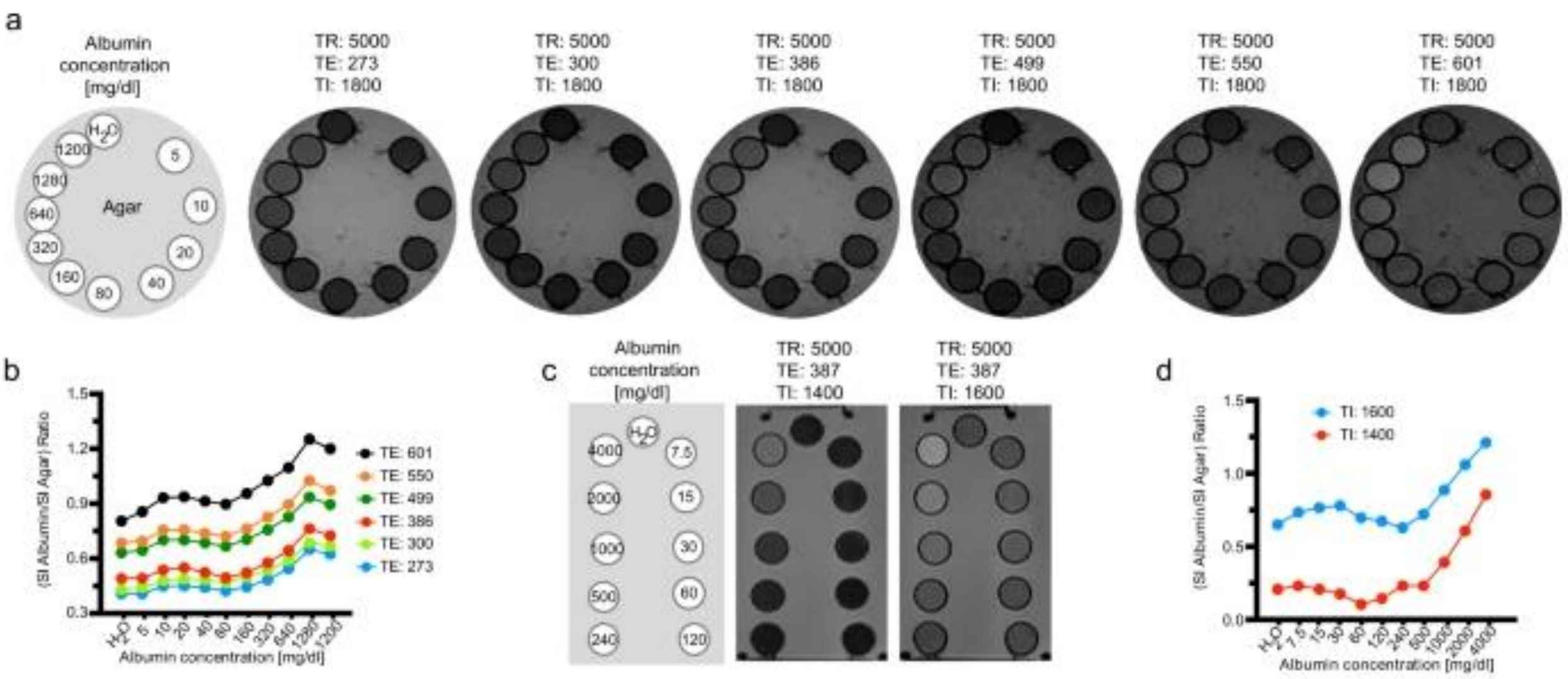

Figure 7 (same as Figure 3 above) Albayram protein concentration phantom study indicating how the amount of protein determines the T2DF intensity. Importantly, we used identical T2DF MRI sequence parameters (TR5000, TE 386, TI 1800 ms)[17], which verifies that the protein concentration in the fluids dictates the results also in our studies together with the verification of in vivo protein correlation to the T2DF MR image intensity above in Fig 2.

But as it is the relation between protein/water, also a change in water concentration influences the signal. These changes may either go hand in hand or in opposing directions in various tissue compartments, which may be the source of the detected changes depending on the anatomical location. The new text on the T2DF (FLAIR) image signal intensity spin dynamics now reads:

We used the dark-fluid (FLAIR-based) MRI signal intensity analysis implicated earlier by other groups for investigating brain solute transport within glymphatic PSD interfaces[22,23,58]. T2DF is a very important sequence in clinical neuroimaging, where tissue-bound water has high signal intensity due to proximity of water ^{1}H proximity to macromolecules like proteins. Less constricted water molecules distant from macromolecules in neurofluidic compartments such as blood and CSF are labelled dark in T2DF MRI by a precisely timed inversion pulse rendering freely precessing water ^{1}H signal dark.

In clinical imaging an acutely increased T2DF signal is often related to arising from brain tissue edema and darkening of T2DF signal typically reflects a reversal of such pathology after a

successful therapeutic interventions. The current T2DF MRI results need to the interpreted differentially based on the brain compartment at hand: while in tissue the darkening may reflect mostly reduced amount of bound water ^{1}H signal, while an increase in normally low signal neurofluidic compartments T2DF suggests increased amount of water molecules close to macromolecules such as proteins.

As shown by a previous protein phantom model verification study with a similar 3T scanner using identical TR, TI and TE sequence parameters, the measured T2DF darkening of reflects reduced protein/water ratio and vice versa with increasing signal in free water solutions[23]. One of the most prominent findings of the vasomechanic intervention was a whole-brain darkening of T2DF MRI signal extending into deep structures (Fig. 1f, 3f). Here, darkening of whole human T2DF image may in theory reflect either reduced amount of tissue bound water resulting in less ^{1}H protons to give MR signal, reduced amount of soluble proteins affecting the watermolecules less, or actually both. In light of the quantified hemodilution, an increase in soluble protein level increase in the blood laboratory results, in addition to the convergent fNIRS hydrodynamic brain sensing results, the most likely change in the brain is a combined redistribution of soluble proteins along with removed brain water, i.e. solutes, from the CSF compartment (Fig. 1f-h, 3h) into blood constituents (Fig 3l).

As normally the NO-induced hemodilution causes a drop in blood protein concentration (Dupuis et al., 1990), the opposing increase we detected in low molecular weight soluble brain derived proteins (< 68 kDa) in blood (Fig. 1d) strongly suggests an increased brain-derived solute efflux directly into the blood. This may well be accompanied with transient BBB related permeability effect, as also seen in the mouse model (Fig. S28b). The dcEEG level that reflects BBB permeability further suggests that BBB permeability could be increased (Kiviniemi et al., 2017). It is also of particular interest that two posterior venous sinus T2DF signal intensities showed a linear correlation with quantified circulating blood protein concentrations (Fig. 1m), linking the imaging findings more directly to blood biomarkers, suggesting a more posterior compartmentalized efflux mechanism in humans.

The low intensity DF compartments of posterior venous sinuses and adjacent PSD regions showed increased DF intensity following the vasomechanic intervention, suggesting the idea of enriched local protein concentration increasing the DF signal, as indicated by Albayram et al. in the PSD spaces(2022) (Fig. 1k-l) (Albayram et al., 2022). In contrast, the more frontally in the medial sagittal sinus and parasagittal/para-cavernous dural CSF regions displayed reduced, i.e. an even darker T2DF signal, consistent with simultaneous water dilution suggestive of either increased protein removal or increased dilution of CSF water (Fig. 1k, 4k).

To the best of our knowledge, this detection of differential solute efflux based on cranial position in humans is a novel finding and thus currently we do not have direct knowledge on the structural mechanical underpinnings of such differences in humans. There could be anatomical and cerebrofluidic pressure-dependent differences in the BBB and PSD permeability favouring dorsal area efflux. Mice have been shown to have differential basal meningeal lymphatic vessel structure and valves function that allows increased tracer efflux compared to dorsal meningeal vessels (Ahn et al., 2019). In the supine position there is also more hydrostatic pressure towards the posterior areas, which, together with the increased ICP during arterial vasodilation (Fig's 1 & 3) could in compartmentalized subarachnoid space CSF favor posterior solute drainage towards the posterior venous sinuses in human cranium (Eide et al. 2020, 2024; Møllgård et al., 2023).

Furthermore, the middle vertex PSD CSF-filled space showed quite a persistent DF signal alteration, with a mean interval between measurements of 9.4 months (range 0.5–22.3 months, Fig. S23), although majority of the T2DF signal intensity changes were transient over most parts of the brain. This seems to be in line with the notion that these vertical areas may have slowest solute

in/outflow processes as already suggested by Eide and co-workers in humans(Eide&Ringstad 2020). Here we show that once there is a change in the PSD solute content, the changes in these areas also seem to persist for months. This slow nature of solute exchange could be partially related to hydrostatic effects in the low pressure environment of venous drainage. Even in recumbent positions hydrostatic pressure of the vertical sagittal sinus is relatively elevated compared to lower venous sinuses, which in theory should direct the pressure driven solute flow towards be posterior areas as shown predominantly to occur in Fig's 1 & 3.

In theory, the T2DF changes could be due to technical Rician noise in low-intensity T2DF signal areas due to altered positioning between scans or T2 flow differences between scans. However, such noise artifacts should be present uniformly in a given area of the T2DF image. Here, however, the T2DF changes were highly compartment specific, and not present uniformly in the brain as should be in the Rician noise case. Neighboring supracellar cistern as well as other central CSF low intensity CSF areas show no changes, while adjacent sinus rectus dark fluid signal shows increases that are linearly correlating with the venous blood both Aβ's, which argues against the Rician noise artefact possibility (Fig, 1h&I, 3h, 4i, S7, S15, S17, S19, S21).

The increased CSF flow speed can also increase T2 DF signal intensity in narrowed CSF areas. However, most global brain changes indicate darkening of T2DF signal. The T2FD signal intensity increases in posterior venous sinuses and PSD areas after vasomechanic intervation do not overlap with any MREG pulsation power increases. We have previously shown that an increase in brain water flow speeds should coincide with simultaneous power increase in the brain tissue and CSF (Elabasy et al., 2025, Järvelä 2025, Väyrynen 2026). During the NO vasodilation the MREG power of especially vasomotor and respiratory pulsations was increased in wide areas; however in these areas the T2DF signal should increase instead of the detected darkening if the signal changes were due to increased flow. After the combined intervention, the pulsation power changes were mostly gone, and thus the T2DF signal increase posterior sinuses and other areas as well sound to be more related to increased blood protein content rather than flow speed increase, c.f Fig 1 m.

3. The conclusion that the combined intervention exerts a synergistic effect is currently stronger than the statistical evidence presented. Although the combined condition appears to produce larger effects than the individual intervention arms, descriptive comparison across conditions is not sufficient to establish synergy. This point should therefore be addressed more formally, either through an explicit interaction analysis or through more restrained wording that refers to a greater combined effect rather than confirmed synergy. As written, the current interpretation overstates what the comparative analysis can support.

A: This was actually absolutely true and in direct relation to the overly enthusiastic interpretations we had previously. A more realistic look at the data shows that indeed the effect size per voxel is not synergistic.

However, we performed a more quantitative analysis now on the spatial extent of the DF image effect: the WBV alone had no spatial changes in DF images, the NO did have a significant change but the combined No+WBVp intervention had 2-6 times larger effect compared to NO on the spatial extent of statistically significant DF signal change over the whole brain, c.f. the new Fig 5 below. The wording was also selected more cautiously here and we removed the word synergistic altogether (albeit 2-6 times higher than NO change volumetrically in the absence of any alterations in WBVp data is in practice the same as synergistic change based on volumetric radiological analytics similar to for instance cancer treatment results) :

“The intervention effect size in the DF MR intensity change was similar in both NO & combined vasomechanic treatment per DF image voxel. However, the spatial extent of the combined vasomechanic intervention was 2-6 times larger than the added volume of both interventions together Fig 5 a.”

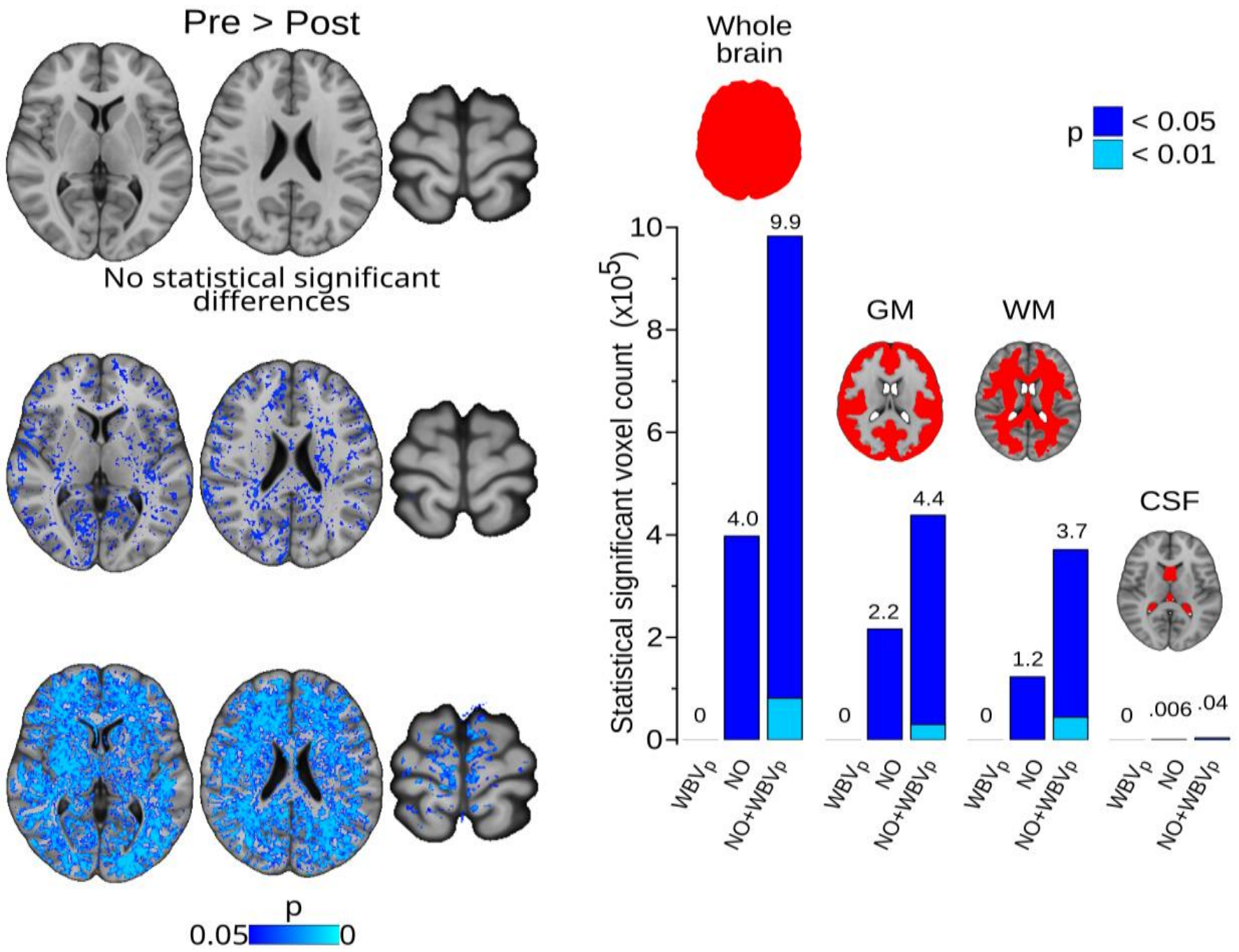

Article Figure 5 a.

Figure 5. Spatial extent of T2DF MRI signal changes and schematic representation mechanisms associated fluid, vascular, and protein redistribution processes across investigated interventions. *T2 dark fluid (T2DF) images from a representative subject before and after the intervention (Pre, Post), group averaged differential maps (Post – Pre, n = 12), and statistical difference maps (Pre > Post, n = 12, two-sided paired t-test, FWER p< 0.05) of* ***a.*** *WBV_p,* ***b.*** *NO and* ***c.*** *combined NO+WBV_p intervention, indicating that the combined intervention had a markedly greater spatial extent in reducing brain DF signal intensity. In NO and NO+WBV_p interventions, mean and difference maps were calculated from images of the same participants. Identical intensity ranges and scaling conventions were applied in all pre and post maps.* ***d.*** *Quantified spatial extent of the significantly altered DF intensity changes in whole brain and segmented compartments per intervention indicating at minimum 2-6 times higher spatial extent in combined NO+WBVp vs. other interventions.*

The statistical framework requires clearer definition in light of the exploratory, multimodal structure of the study. In particular, the manuscript would benefit from a more explicit specification of the primary endpoint, or primary endpoint family, together with a clearer account of how variation in sample size across modalities and analyses was handled. The study is presented within a pilot framework, yet the manuscript integrates multiple physiological, imaging, and biomarker readouts across partially overlapping cohorts. Under these conditions, a more clearly articulated analytical hierarchy is needed to distinguish hypothesis driven outcomes from broader exploratory observations and to improve the overall interpretability of the results.

A: We thank the reviewer for this important and insightful comment regarding the statistical

framework and analytical hierarchy of the study. We have revised the manuscript to improve clarity in study design, endpoint definition, and interpretation of results.

First of all, we have explicitly clarified the study design in the Methods section (Study design), which now reads as follows:

"Eligible participants completed two intervention visits in randomized order: (1) NO-mediated vasodilation with whole-body vibration (NO+WBVp) and (2) Piezo1-targeted whole-body vibration (WBVp). After the first visit, each participant crossed over to the alternate intervention, resulting in a randomized crossover design. A washout period of at least seven days separated the visits. The study flow is summarized in the CONSORT diagram (Fig. S1).

To investigate the acute effect of NO-mediated vasodilation (NO) alone in more detail (i.e., without WBVp), an additional NO-only session was conducted in a subset of participants (n = 12). This session was performed as an exploratory sub-study and was not part of the randomized crossover design. Accordingly, analyses involving this session were considered exploratory and interpreted separately from the primary endpoint family.

In addition, a separate supplementary cohort (n = 13) underwent a sham intervention without NO-mediated vasodilation or whole-body vibration. This cohort was used to assess changes in T2DF MRI signal intensity in the absence of active intervention and was analyzed in an exploratory manner, separately from the primary endpoint family.

Given the multimodal and exploratory design, we defined a primary endpoint family consisting of (i) circulating concentrations of brain-derived proteins, (ii) T2DF signal intensity changes, and (iii) brain pulsation changes due to the interventions. All other analyses were considered exploratory and interpreted accordingly."

Secondly, we have clarified in the manuscript the distinction between hypothesis-driven analyses (primary endpoint family) and exploratory analyses, particularly with respect to the NO-only sub-study and the sham cohort, which are now consistently described as exploratory throughout the text.

Intro changes at the end :

" Because both NO-mediated vasodilation and Piezo1 activation have been shown to enhance CSF efflux, we hypothesized that simultaneously augmenting these functions globally in humans could promote the removal of potentially harmful brain-derived proteins. Specifically, we hypothesized that the intervention would be associated with (i) increasing blood concentrations of brain-derived proteins, (ii) detectable changes in brain T2-weighted dark-fluid (T2DF) magnetic resonance imaging (MRI) signal intensity, and (iii) alterations in brain hydrovascular pulsation dynamics. To address this, we investigated the effects of combined NO-mediated vasodilation and Piezo1-targeted vasoconstrictive mechanical stimulation (WBV_p), as well as both interventions separately, and examined whether the combined intervention could influence CSF-to-blood solute transport in humans.

We exploited the joint capacities of advanced neurofluidic sensing technology linked to synchronous ultrafast brain pulsation magnetic resonance encephalography (MREG) imaging with previously established T2DF MRI to quantify the changes in neurofluidic drivers of protein-rich fluid shifts, as demonstrated in prior studies using to assess CSF protein burden[22,23], and to characterize physiological changes in brain water dynamics and macromolecule distributions. We

combined these measurements with clinical quantification of blood levels of brain-derived proteins that are relevant to neurodegeneration, including $A\beta_{40}$, $A\beta_{42}$, and glial fibrillary acidic protein (GFAP).”

Thirdly, variation in sample size across modalities and analyses has been made transparent by reporting the exact sample size for each analysis in the Results section and in figure legends. Fourth, the Supplementary Figure S21 legend has been expanded to explicitly describe modality-specific data availability within the context of the study design. Finally, the Discussion section has been rewritten. Together, these revisions provide a clearer specification of the analytical framework and improve the interpretability and transparency of the results.

5. The alignment between the clinical protocol and the final intervention description should be clarified more carefully, particularly with respect to nitroglycerin dosing and timing. The protocol describes a pilot design in which nitroglycerin was administered around imaging and treatment periods under blood pressure based constraints, and because the key physiological and biomarker findings are tightly linked to hypotensive responses, it would be important for the final manuscript to state explicitly how the analyzed NO and combined intervention arms were operationally implemented, whether the dosing structure was identical across participants, and whether any protocol refinements occurred during the course of the study.

A: Thank you for pointing out this important shortcoming in our previous approach.

We have revised the Methods section to provide a detailed description of nitroglycerin administration for each intervention condition and to clarify the timing of blood sampling and imaging procedures.

The *Study drug* section now states: “In the combined NO+WBVp intervention, nitroglycerin was administered as two sublingual 0.5 mg doses. The first dose was given during MRI acquisition, and the second dose was administered approximately 20–30 min later during the WBVp treatment period. In the NO-only intervention, a single sublingual 0.5 mg nitroglycerin dose was administered.”

To further clarify the temporal relationship between intervention, imaging, and biomarker sampling, we expanded the *Blood tests* section as follows: “Venous blood was drawn immediately before and after the NO+WBV_p intervention. Participants underwent the pre-intervention blood sampling prior to any active intervention procedures. The interval between the pre-blood sample and the pre-T2DF MRI acquisition was approximately 1 hour, during which participants were prepared for the measurements (e.g., instrumentation and setup) without exposure to active interventions. The post-intervention blood sample was collected approximately 10–15 minutes after the post-T2DF MRI acquisition, corresponding to approximately 1.5 hours after sublingual NO (0.5 mg) administration.

To evaluate the potential NO-induced hemodilution, a complete blood count (including hemoglobin, hematocrit, red and white blood cells and platelets) was obtained immediately before and after the NO intervention (n = 10). The pre-intervention blood sample was collected approximately 1 hour prior to imaging, during which no active intervention was performed and only preparatory procedures were carried out. The post-intervention blood sample was collected approximately 1 hour after sublingual NO (0.5 mg) administration. “

6. The safety and tolerability statements should be framed with greater precision. Although no serious adverse events were reported and the study appears feasible under monitored conditions, the manuscript also documents headache, nausea, and one discontinuation due to severe nausea. The wording should therefore be moderated accordingly, with the intervention described more specifically as feasible and acceptably tolerated in a closely supervised short term study of healthy volunteers. A clearer presentation of adverse events by intervention condition and severity would further improve the clinical transparency of the report.

A: Thank you pointing this out, we did not do a proper job on this originally. We have now revised the manuscript to provide a more detailed and accurate description of adverse events, including their severity, timing, and relation to the specific intervention. Specifically, the Results Treatment side effects section has been clarified to distinguish between NO-related and WBVp-related effects, and to explicitly report the nature and outcome of adverse events. The revised text now reads as follows:

“The interventions were feasible and acceptably tolerated under controlled experimental conditions, although transient adverse effects were observed: Mild adverse effects were reported following NO administration, with four participants reporting headache and three reporting nausea. In one case, the study was discontinued due to vomiting following NO administration. All reported symptoms resolved during the observation period within 30 minutes after active intervention. No participants reported discomfort associated with the whole-body vibration procedure. There were no serious adverse events.”

7. The murine experiments add useful biological support to the study, but their translational role should be defined with greater precision. In the current form of the manuscript, the animal data occasionally come close to being presented as mechanistic validation of the clearance route inferred from the human imaging findings. A more disciplined framing would be preferable. Specifically, the murine results are better positioned as complementary experimental support for the broader biological model rather than direct validation of the precise human pathway inferred from the present data. This distinction is important in the meningeal lymphatic and glymphatic field, where broad conceptual overlap across species coexists with substantial differences in anatomy, scaling, drainage predominance, and route visibility. Clarifying this point would strengthen the translational argument while preserving the importance of the animal work.

A: We thank the reviewer for pointing this out and it confirms what we were originally thinking of the positioning of the verificatory translational mice experiments. All the mice results are indeed in the supplementary information of the manuscript and thus they have :” **complementary experimental support for the broader biological model rather than direct validation “**

We now added to Results section: “We also conducted intrathecal tracer studies in mice to provide complementary biological support for the observed phenomena.”

The following has now been added to the discussion section: ”In rodents the CSF clearance is associated with the dura mater, which contains lymphatic vessels and blood-collecting venous sinuses. Substances present in CSF may be absorbed into dura mater, where they transit into

blood and lymph for drainage. We have recently shown that accumulation 40kDa fluorescent tracer from CSF into dorsal dura mater is significantly increased with blood pressure lowering in mice (Jukkola et al. 2024). Interestingly, combining the WBV with SNP-induced blood pressure lowering significantly reduced the amount of the same tracer in dura while increasing its transit to blood (Fig. S28), thus suggesting complementary action promoting dural clearance in posterior regions similar to the current results in human posterior venous sinuses (Fig 1l). A recent study indicated that solute efflux from the human brain can occur also partly through perineural routes, as seen in rodents[60], whereas paraspinal CSF pathways may represent major contributors to net efflux[61].“

In the second-to-last paragraph of the discussion, we also mention the following: “The murine experiments provide complementary biological support for the overall framework, rather than directly validating the specific mechanisms inferred from the human data. “

8. The age related findings are intriguing and potentially important, but I would suggest presenting them more clearly as exploratory unless their robustness can be further established. The cohort size is still modest, the age range is limited, and the main manuscript notes outlier masking in at least one correlation analysis. Under these conditions, the age dependent interpretation should be phrased carefully and ideally supported with fuller sensitivity analyses and transparent display of all data points.

A: Thank for pointing these important issues out, we have yet again looked at them with a more realistic approach.

In the age-related T2DF image (Fig. 1i-j) change analysis however, there were no outlier removals.

The outliers were removed from the in the blood pressure vs. serum protein level analysis in manuscript Fig. 1e, outliers (> 2.5SD, 3 subjects from Ab40 & -42 and 1 subject GFAP) were masked before the final correlation analysis. This is explained in the statistical analysis section. Now we have also added this same image with masked data (below) to supplementary information (Fig. S4).

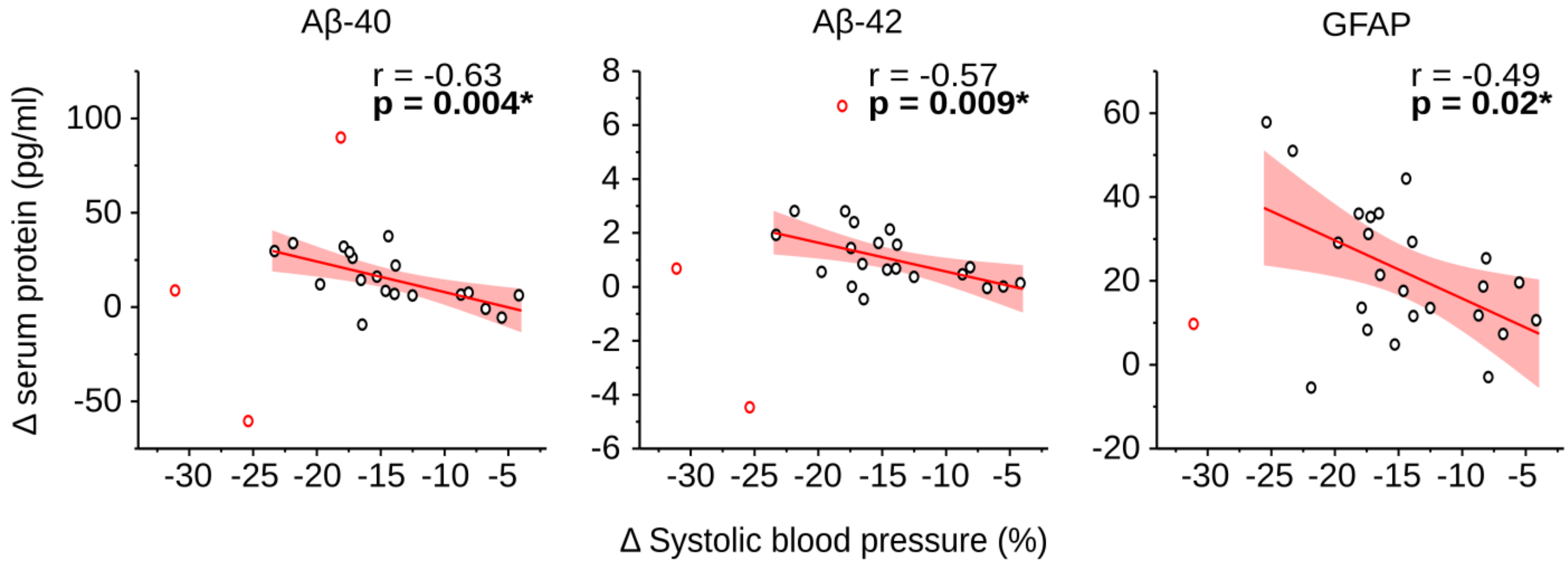

But indeed the number of subjects is still quite limited and age range is also quite wide albeit extending over decades prior to the usual emergence of neurodegenerative diseases against which the interventions are in part aimed against. Again due to these limitations we toned down the wording, even though the T2DF signal vs. age in Fig 1 j, and, GFAP change (manuscript S5) in serum vs. age all have do have a correlation r > 0.4, while as r > 0.3 is often considered statistically significant in literature.

We now toned down the conversation on the issue and added a sentence to discussion:

“These changes may help explain the age dependency observed in the present hydrodynamic responses (Fig 1i-j, S5), although given the modest sample size especially in the separate NO and WBVp studies, these age-related observations should be interpreted cautiously.

9. The central biological interpretation of the manuscript rests heavily on rapid post intervention changes in circulating biomarkers, and this aspect of the study would benefit from further analytical support.

If additional post intervention time points are available, these would be especially informative. If not, the mechanistic interpretation should be correspondingly moderated.

A: This is an important key validation point in the study, thank you for pointing it out.

Encouraged by this reviewer point, we went on to perform a long term surveillance analysis of the most distant DF ROI analysis results by comparing the 1st vs. last T2DF baseline, c.f. ms Figure S23 below.

There we found that there is a long term effect in the mid vertex PSD that last at least for an average 9 months between the NO and EAC treated subject n = 19. The parasagital dural space intensity reduces for months after the interventions suggesting at least in the presumably slowly removed dural areas that the effect is indeed long lasting. The CSF and blood do not change as it is understandable that there is a daily turnout in these brain compartments.

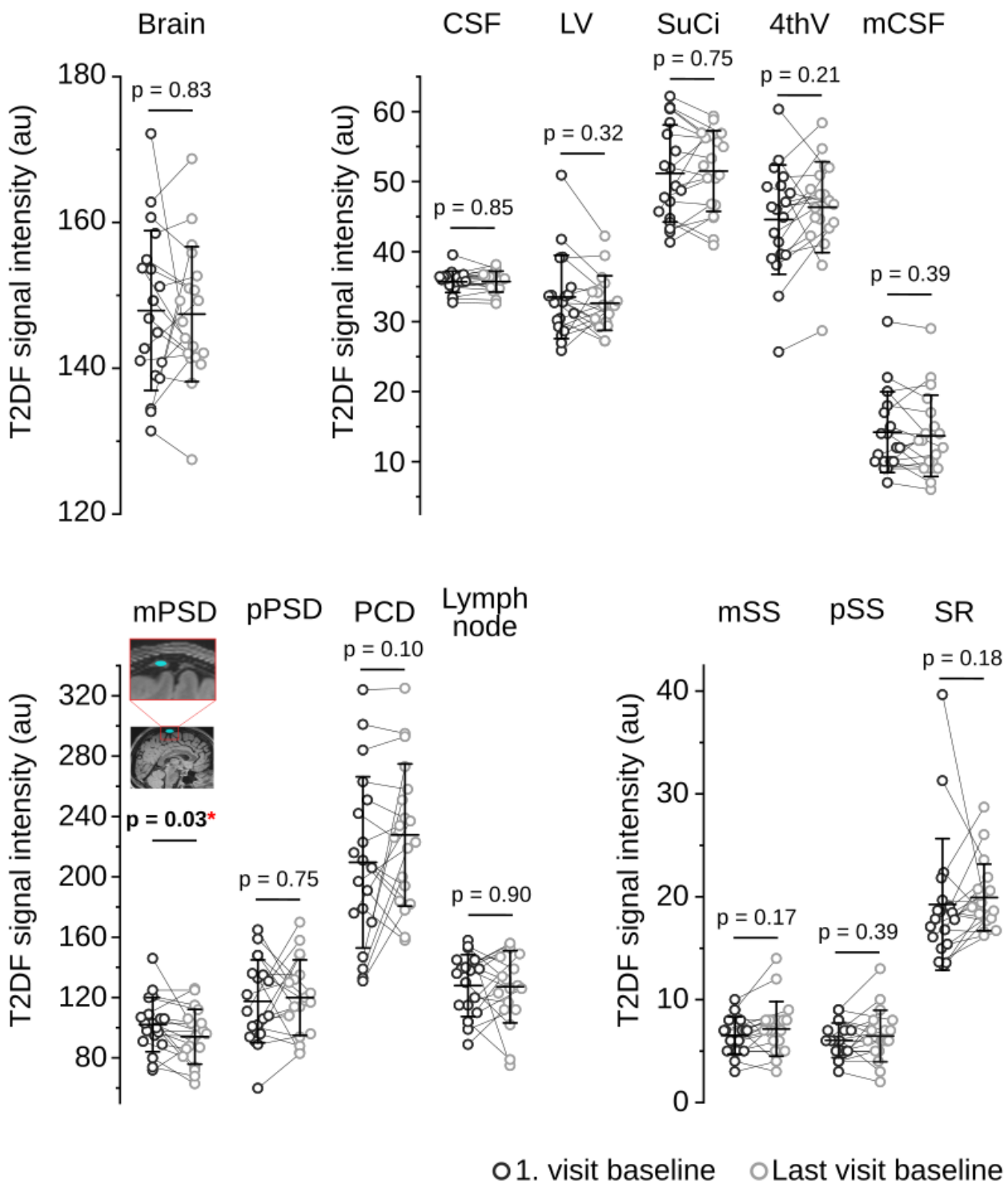

Manuscript supplementary Figure S23. Longitudinal stability of T2-weighted dark-fluid (T2DF) MRI signal intensity across study visits. T2DF signal intensity was quantified from regions of interest defined on the baseline image of the first visit and compared with the baseline image of the last visit (n = 19; mean interval 9.4 months, range 0.5–22.3 months). No significant changes were observed in whole-brain or cerebrospinal fluid (CSF) T2DF signal intensity between visits. Among individual regions, only the middle parasagittal dura CSF-filled space (mPSD) showed a significant

decrease in signal intensity (p = 0.03), whereas all other regions remained stable. Data are shown for the following regions: Segmented whole-brain tissue (Brain), global cerebrospinal fluid (CSF), lateral ventricles (LV#), superior cisterna (SuCi), fourth ventricle (4thV), middle CSF space (mCSF#), middle parasagittal dura CSF-filled space (mPSD), posterior parasagittal dura CSF-filled space (pPSD), para-cavernous dura CSF-filled space (PCD), lymph node), middle/vertex sagittal sinus (mSS), posterior sagittal sinus (pSS), and sinus rectus (SR#). All scatter plots show means, with whiskers extending to one standard deviation. Each line represents an individual subject. Comparisons were performed using two-sided paired t-tests. #Paired-sample Wilcoxon Signed-Rank test was used for comparison.

We went on to analyze the pooled pre+post Simoa HD-X lab findings in comparison to the segmented DF MRI data and found linear correlations between blood protein concentrations and venous sinus and total brain signal intensity, see also below, (Manuscript Fig. 1m, S8).

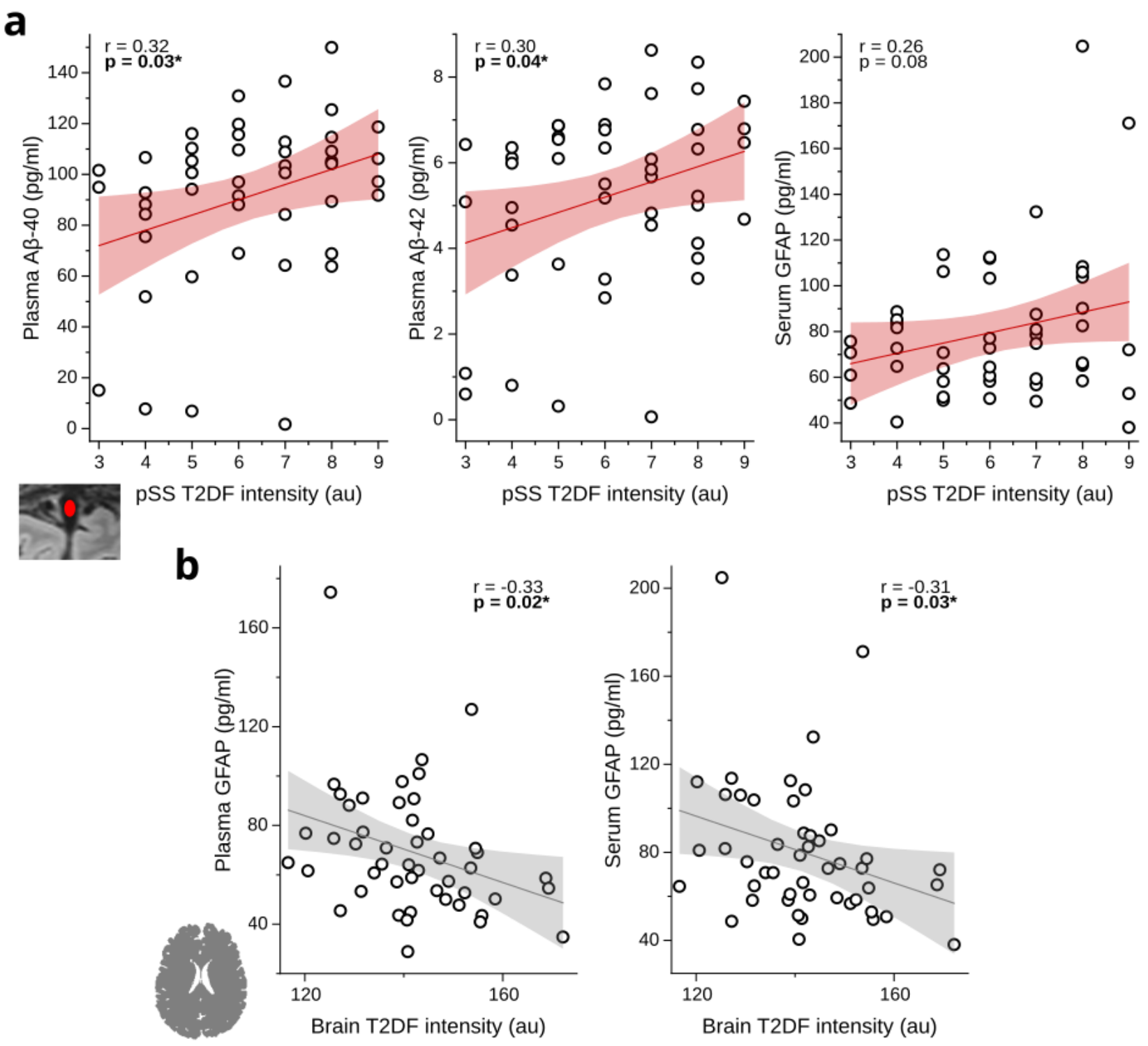

In particular, the interpretation would be strengthened by a more explicit assessment of biomarker kinetics and concordance, including closer integration of serum and plasma findings, fuller accounting for hemodilution related variables, and clearer subject level comparison between biomarker shifts and the corresponding physiological or imaging changes.

We thank the reviewer for this important key validation issue; previously we were not able to add some of our key articles as they were just published after the initial submission of the current manuscript. We now added several verification articles on the matter including Quanterix Simoa

HDX vs. Mass Spec golden standard data on healthy controls, Ad, FTD and DLB patients from 2 cohorts.

REDACTED

Table1 from Thijsse et al. study. *The concordance of the Simoa HDX plasma readings with respect to both CSF golden standard Ab42 measurements and plasma mass spectroscopy data indicating high correlation between the plasma readings in two cohorts*[25]. Thijssen et al., Differential diagnostic performance of a panel of plasma biomarkers for different types of dementia,2022 Diagn Assess, DOI: 10.1002/dad2.12285

Additional Simoa references:

Ashton, N. J., Janelidze, S., Al Khleifat, A., et al. (2021). A multicentre validation study of the diagnostic value of plasma neurofilament light. Nature Communications, 12, 3400.

Ashton, N. J., Janelidze, S., Al Khleifat, A., et al. (2021). A multicentre validation study of the diagnostic value of plasma neurofilament light. Nature Communications, 12, 3400.

Wilson DH, Rissin DM, Kan CW, et al. (2016). The Simoa HD-1 analyzer: a novel fully automated digital immunoassay analyzer with single-molecule sensitivity and multiplexing. J Lab Autom. 2016;21(4):533-547.

Wilson D., Chan D., Chang L., et al. (2023). Development and multi-center validation of a fully automated digital immunoassay for neurofilament light chain: toward a clinical blood test for neuronal injury. Clin Chem Lab Med. 2023 Sep 13;62(2):322-331.

Zhang, H., Tan, J., Zhang, N., & Lin, Z. J. (2024). Comparison of plasma pTau217 assays on different platforms. The Journal of Prevention of Alzheimer’s Disease, 11(5), 1206–1211.

All of the laboratory procedures were under approved standards Verberk et l., Alzheimers Dement . 2025 Sep 30;21(10):e70752. doi:10.1002/alz.70752

We were also able to reference now our recent brain electro/vaso/hydrodynamic sensing technology in our[7,8,26] PNAS & Adv Science papers where we illustrate a high concordance between simultaneously acquired hydrodynamic brain water fNIRS vs. water flow detection in ultrafast MREG vs. dcEEG (that reflects BBB permeability) was shown in measuring infraslow brain fluctuations in each measure simultaneously; we can now add this reference into the article as it was just published in PNAS. There increased power of waves was related to increase water movement speed of brain hydrodynamic water waves through the human brain tissue. Just after that it was shown that indeed the astrocytes do shrink in sleep allowing water to flow faster via tissue[24]

Images captured here are from Väyrynen et al., 2026 PNAS paper for faster and easier image access. The

REDACTED

results co-verify the sensitivity of the fNIRS and dcEEG signal detection of infraslow brain waves with all these techniques. Also the results show that vasomotor waves take over sensory areas in NREM sleep and start to drive electrophysiological signal and water hydrodynamics locally once the sleep induced solute flushing of the brain starts. The water flow through the brain speeds up in the areas of increased vasomotor power in sensory cortices (green areas on right up A images) and the solute dilution lowers EEG to delta power driving the ISF dcEEG potential along with water.

REDACTED

Plase also see commentary to the article:

https://www.pnas.org/doi/epub/10.1073/pnas.2608470123

The results of the PNAS paper show virtually identical infraslow (ISF) wave results as compared also to mice results at microscopical level where also NREM sleep increases ISF vasomotor waves drive both electrical and opposing brain water dynamics[6,7] in deep brain tissue in both humans and mice the water tends to lead as shown below but in human sensory areas also water follows vasomotor waves.

REDACTED

The PNAS article shows that also the infralow brain fluctuations and their directed coupling changes are robustly detected in all three measures of electrophysiology, hydrodynamics and vascular measures used also in the current reviewed article.

The MREG data is also precisely concordant with the faster cardiorespiratory physiology as shown in our article Järvelä in Nat Med Com[27].

REDACTED

The same close to 1:1 correlation was shown for MREG vs. cardiorespiratory data in Tuovinen et al., 2020; Kananen et al., 2022; Tuunanen et al., 2024. Haverinen et al., 2026.

Minor Comments

1. The presentation of participant numbers should be made more consistent across the manuscript. Although the supplement provides a CONSORT style overview and modality specific availability, the main text would be easier to follow if the reasons for moving from 29 enrolled participants to smaller analytic subsets were stated more explicitly at the first relevant point in the Results.

A: Indeed, thanks for this comment. We have revised the manuscript in total to improve clarity and consistency in the presentation of participant numbers. First, the Results section has been clarified so that the structure of the study population and the derivation of analytical subsets are explicitly described at the beginning of the section. This allows the reader to more easily follow how the initial cohort of 29 participants contributed to the different analyses. Second, we have provided more detailed statistical reporting throughout the manuscript, including the relevant test statistics (e.g., paired t-test t(DF), Wilcoxon W values) together with the corresponding sample sizes for each analysis. Thirdly, we have clarified the legend of modality specific availability figure (Fig. S27). This ensures that the number of participants included in each comparison is clearly indicated at all points.

2. The nomenclature of the intervention arms should be standardized throughout the manuscript and figure legends. At present, WBVp, WPVp, NO+WBVp, and NO+WPVp appear interchangeably. A uniform terminology would improve clarity, particularly when comparing the isolated and combined intervention conditions.

A: Deep apologies for this and thanks for pointing it out..we thought it should have been corrected after several re-corrections. Now the WBVp indeed corrected.

3. The manuscript would benefit from a careful language edit for precision and fluency. There are several small but noticeable issues in the current text, including "of who 15 females," "combined to Piezo1 targeted whole body vibration," "At 15 ans 45 min," "with the any of the whole body vibration procedure," and "not significantly unaffected."

A: The manuscript was indeed edited for language by a native english speaker and an editor or scientific journal NeuroImage. Despite this we here were able to miss these corrections for these obvious grammatical errors and now have re-proofread the article word by word more clearly. Apologies for these obvious lapses on attention from the writer's part.

4. The statistical terminology should be revised where "two sample paired t test" is used in the Methods and figure legends. If the analyses were conducted within subjects, they should simply be described as paired t tests.

A: Thanks for pointing this out, it was typo. "Two sample paired t-test" has to be "two-sided paired t-test". Now corrected.

5. The Methods would benefit from a more explicit account of the timing of blood sampling and MRI acquisition relative to the intervention period in each study arm. This is particularly important because the interpretation of both the biomarker and dark fluid MRI findings depends closely on temporal proximity to the physiological response.
A:

We thank the reviewer for this important comment regarding the temporal alignment of blood sampling and MRI acquisition. We have revised the Methods section to provide a more explicit description of the timing of blood sampling and imaging relative to the intervention.

Specifically, we now clarify that NO+WBVp pre-intervention blood sample was obtained prior to any active intervention, with an approximately 1-hour interval before the pre-intervention T2DF MRI during which only preparatory procedures were performed. We further specify that the post-intervention blood sample was collected approximately 10–15 minutes after the post-T2DF MRI acquisition, corresponding to approximately 1.5 hours after sublingual NO (0.5 mg) administration.

To evaluate the potential NO-induced hemodilution, blood test was obtained immediately before and after the NO intervention (n = 10). The pre-intervention blood sample was collected approximately 1 hour prior to imaging, during which no active intervention was performed and only preparatory procedures were carried out. The post-intervention blood sample was collected approximately 1 hour after sublingual NO (0.5 mg) administration.

6. A brief justification should be provided for treating serum as the principal matrix for the core biomarker analyses while presenting plasma data in the supplement. Since both are shown, this distinction should be clarified.

A: Thanks for pointing this out this important issue that falls into clinical chemistry that is a bit out of

our experience and knowledge fields. Also these superbly handy and important blood Alz biomarkers are quite new tools so experience is just accumulating.

Thanks to the reviewer again we now learned that indeed serum generally yields higher absolute concentrations of Amyloid-beta 40 (Aβ40) compared to plasma. This is because Aβ peptides are abundantly stored in platelets; during serum coagulation, platelets aggregate and release Aβ into the serum. Plasma, having been treated with anticoagulants (like EDTA or heparin), avoids this artificial spike. GFAP results are not affected by serum vs. plasma differences, however.

However, as shown below in manuscript supplementary S3 figure that both serum and plasma main results Aβ and GFAP results after intervention are highly congruent as literature states also. We chose to present serum results in Fig 1 as it also correlated with blood pressure. However, the plasma was shown in supplementary mainly to save space and text and in order to keep the Figure 1 clear.

The Figure S3 below shows all the Simoa results after NO+WBVp intervention indicating that the results are indeed present in plasma and serum alike.

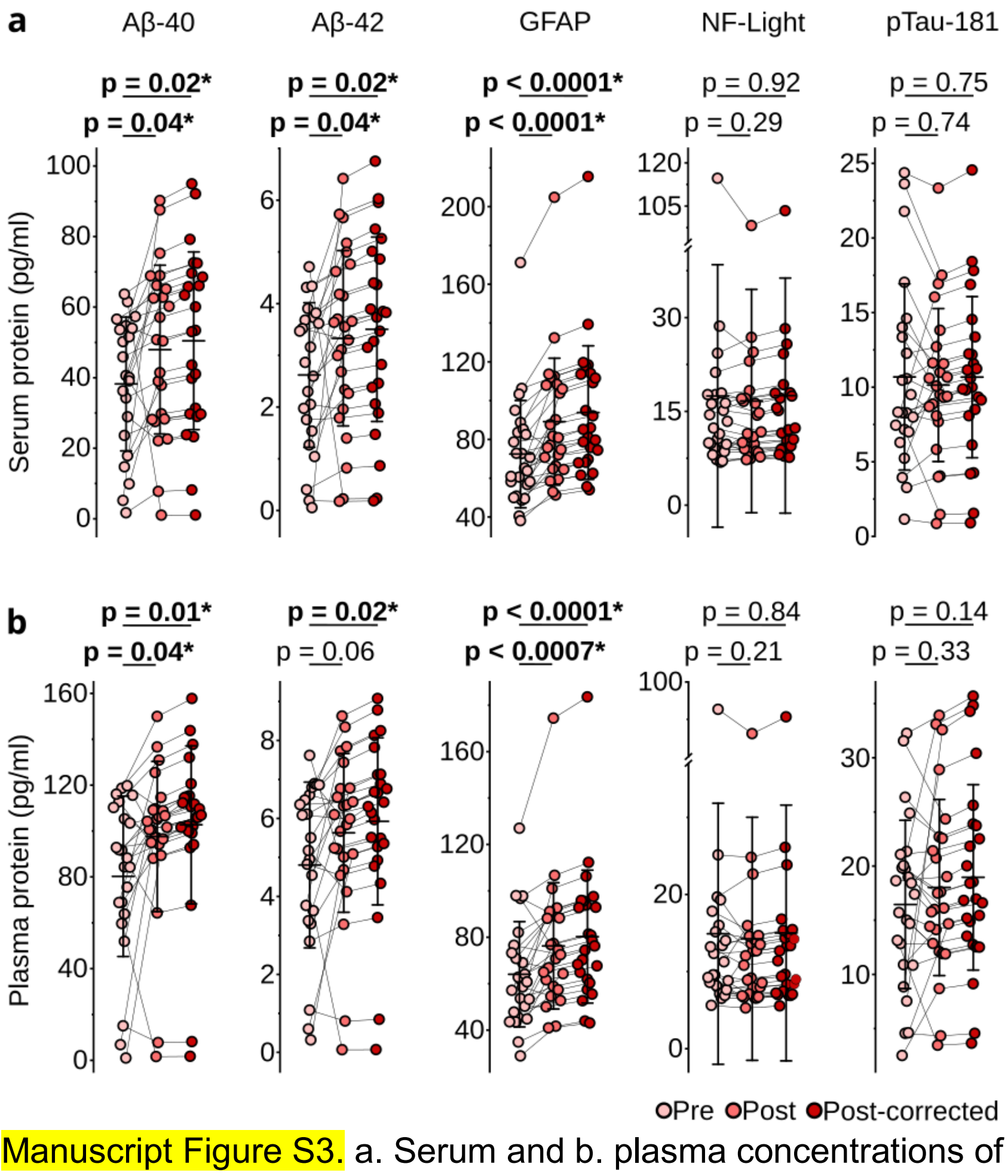

Manuscript Figure S3. a. Serum and b. plasma concentrations of brain-derived proteins before (Pre) and after (Post) NO+WBVp intervention, with post-intervention values additionally shown after hemodilution correction (Post-corrected). Mean (± standard deviation), two-sided paired t-test. Aβ40 & Aβ42 = beta-amyloid 40 & 42, GFAP = glial fibrillary acidic protein#, NF-Light = neurofilament-light#, pTau-181 = phosphorylated tau-181. All scatter plots show means, with whiskers extending to one standard deviation. Comparisons were performed using two-sided paired t-tests unless otherwise indicated. #Paired-sample Wilcoxon Signed-Rank test was used for comparison.

The further correlation to blood pressure occurs in serum, while the T2DF signal correlation is more related to plasma results and we chose to present only significant results as is. The reason for this could in theory be linked to NO co-effect on blood coagulation and thus on secondarily affect Ab concentrations in serum. Then again at the time of the venous blood sample the NO effect had most likely been gone. The plasma results show higher Ab concentrations that could be more directly then related to absolute venous blood concentrations in T2DF image intensity. However, the GFAP concentrations with also lienar T2DF correlation in brain tissue were related to both serum and plasma results and there we do not know the reason clearly. In any case, the laboratory vs. other biomarker issues are interesting topics for further research but current explanations are highly speculative and outside our expertise and the scope of this article.

Now added a sentence to Methods in Blood tests.

"Serum results are reported in the main Figure 1 and concordant plasma results in Supplementary figure S3. In further correlation analyses of blood samples data, only statistically significantly correlating sample data is illustrated in figures. "

7. Because hemodilution is central to the interpretation of the biomarker results, the Methods should state more explicitly how the correction factor was derived, whether it was applied uniformly across analytes, and whether the main conclusions were robust to uncorrected versus corrected analyses.

A: We thank the reviewer for this important comment. Because nitroglycerin is known to induce hemodilution[16,28], we considered it necessary to account for this effect when interpreting circulating biomarker concentrations. In the study by Arend et al., hematocrit decreased by approximately 11% and hemoglobin by 10% following nitroglycerin administration, with comparable reductions observed in serum protein and albumin concentrations. Dupuis et al. further demonstrated that a 9% hemodilution corresponded to an increase in total blood volume, most of which occurred during the first hour after nitroglycerin administration.

Importantly, as Dupuis et al. state in their 1990 study, the blood protein (and blood cell) concentration after NO medication usually drops in blood due to hemodilution by water; however we find both stable T2DF signal after solo use of NO and intriguingly even opposite protein and T2DF increasing values after combined NO+WBVp with the specific biomarkers of Ab and GFAP necessitating the correction. Yet, even without the correction, the increases in these proteins is statistically significant (supplementary figure S3 above).

As acute nitroglycerin-induced hemodilution has been only sparsely quantified in humans, we sought to determine the magnitude of this effect under the specific conditions used in the present study (0.5 mg nitroglycerin). To achieve this, complete blood counts were obtained from 10 participants immediately before and after NO intervention. Hemoglobin, hematocrit, red blood cell count, white blood cell count, and platelet count were measured, and the mean percentage decrease across these parameters was calculated. This yielded an average reduction of 5.2%, which was used as the hemodilution correction factor.

The correction factor was applied uniformly to all analyses and was used only in the analyses presented in Figures 1d–e. For transparency, the Supplementary Material (Fig. S3) also includes the corresponding uncorrected values. Importantly, the key findings remained unchanged, as the protein increase remained statistically significant even in the uncorrected analyses. We have revised the Methods section and Supplementary Information Text 2 to describe the derivation and application of the correction factor more explicitly.

Now added:

Supporting information Text 2

Hemodilution correction factor: To derive a study-specific hemodilution correction factor, the relative percentage changes in blood parameters (hemoglobin, hematocrit, erythrocytes, leukocytes, and thrombocytes) were calculated for each participant, and the mean percentage decrease across parameters was used as an estimate of blood dilution. This yielded an average reduction of 5.2%, which was applied as a global hemodilution correction factor.

The correction factor was applied uniformly to all analytes and was used only in the analyses presented in Figures 1d–e. Corresponding uncorrected values are provided in the Supplementary Figure S3. Importantly, the main findings remained qualitatively unchanged, as increases in circulating proteins were also observed in the uncorrected analyses. The blood test values are available in Source dataset S2.

Methods:

The pre-intervention blood sample was collected approximately 1 hour prior to imaging, during which no active intervention was performed and only preparatory procedures were carried out. The post-intervention blood sample was collected approximately 1 hour after sublingual NO (0.5 mg) administration. The analyses were performed by Nordlab (Oulu, Finland). The blood tests values are available in Source dataset S2. A detailed description of the derivation and calculation of the hemodilution correction factor is provided in Supplementary Information Text 2.

8. The ROI based dark fluid analyses would be easier to evaluate if the Methods provided slightly more detail regarding ROI delineation. In particular, it would be helpful to clarify whether raters were blinded to condition, whether ROIs were drawn independently on each scan or propagated across time points, and whether any internal consistency checks were performed.

A: Thank yout for the comment.

Raters were not blinded to the treatment; the focus was to avoid mixing blood vessels and focus on a single fluid compartment in each ROI based on additional T1 and T2w 3D images. Now more details has been written about the procedures.

ROI selection has now been clarified in the Methods, and a new Supplementary Figure (Fig. S25) illustrates ROI placement with enlarged examples.

All MRI datasets from each intervention were visually inspected and aligned to a consistent anatomical orientation and slice position prior to ROI delineation. ROI definition was performed on T2DF images using a diagnostic DICOM viewer, with simultaneous reference to T1- and T2-weighted anatomical images to ensure accurate identification of dural CSF spaces and lymphatic structures. ROIs were drawn individually for each subject and each scan to account for anatomical variability. For longitudinal comparisons, particular care was taken to ensure that ROIs were placed in identical anatomical slices and orientations between pre- and post-intervention images. Matching was based on anatomical landmarks and image geometry to ensure that the same structures were sampled across time points. For parasagittal regions, adjacent anatomical compartments were delineated within the same slice: posterior sagittal sinus (pSS) and posterior parasagittal dura CSF space (pPSD) were defined together, and similarly, middle/vertex sagittal sinus (mSS), middle parasagittal dura CSF space (mPSD), and medial CSF regions were defined within corresponding slices. This approach minimized spatial mismatches and ensured

comparability of signal changes across anatomically related regions. ROI size was maximized within anatomical boundaries to reduce the influence of single-voxel variability. Care was taken to avoid partial volume effects and inclusion of adjacent tissue types. For selected structures (lateral ventricles, fourth ventricle, superior cisterna, and sinus rectus), additional ROIs were generated using FSL-based thresholding tools. Subject-specific masks were created and applied identically to both pre- and post-intervention images to ensure consistency. This combined approach of anatomically guided manual delineation and standardized mask-based ROI extraction ensured high spatial consistency, reproducibility, and robustness of signal measurements across subjects and time points.

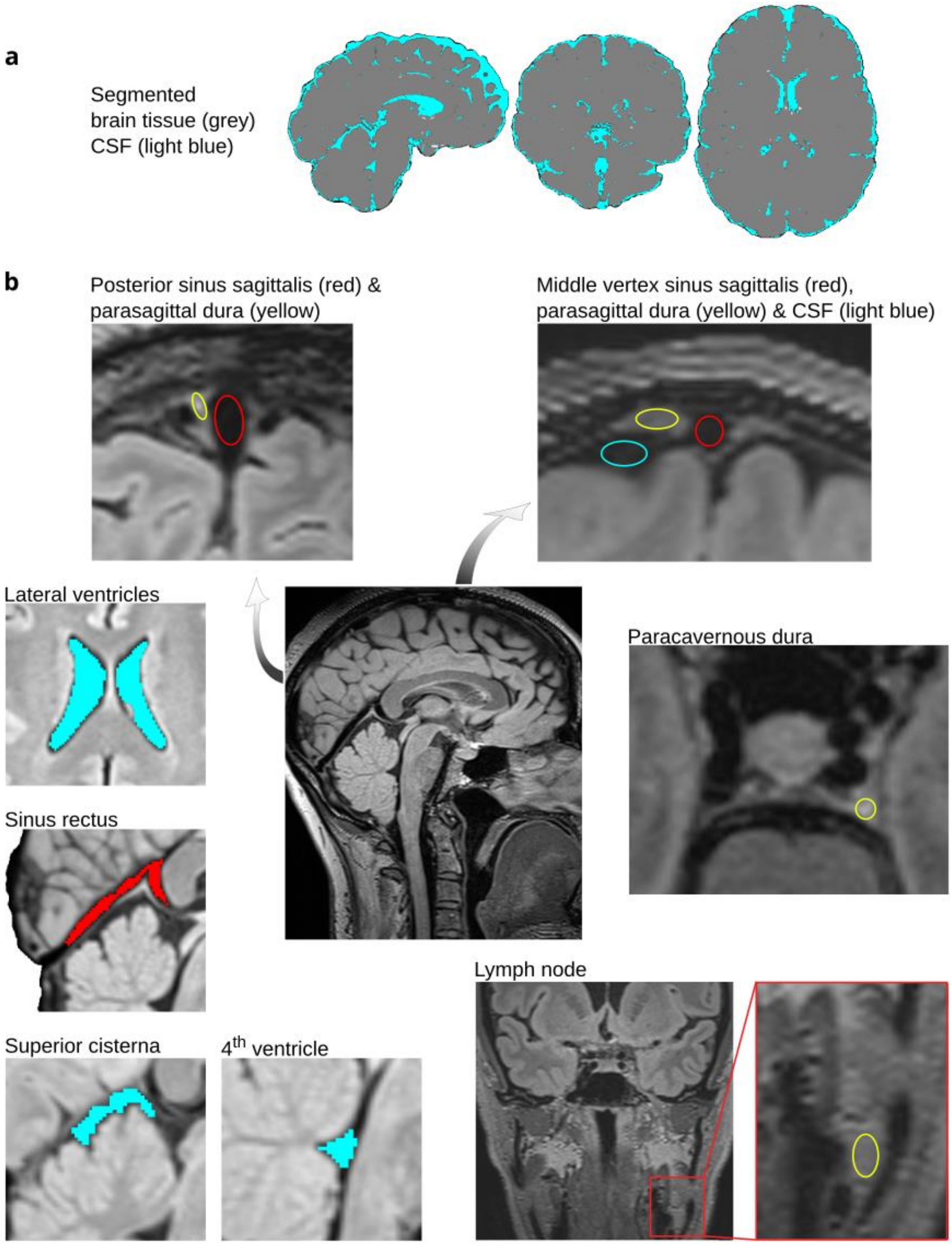

Figure S25. Segmentation and ROI definition. a. Representative segmentation of brain tissue (grey) and cerebrospinal fluid (CSF, light blue) from a single participant from T2-weighted dark fluid (T2DF) image. b. Representative examples of individually defined regions of interest (ROIs) from a single participant. Individual ROIs of posterior sinus sagittalis and parasagittal dura CSF-filled space, middle/vertex sinus sagittalis, parasagittal dura CSF-filled space and CSF, paracavernous dura CSF-filled space and lymph node were manually delineated on T2DF MRI images using a diagnostic DICOM viewer, guided by co-registered T1- and T2-weighted images to ensure accurate anatomical localization of CSF-filled dural spaces and lymphatic structures. For each subject and

scan, ROIs were drawn individually in matched anatomical slices and orientation to ensure direct comparability between pre- and post-intervention images. Anatomical regions were selected to maximize ROI size within each structure while avoiding partial volume effects, and identical ROI placement was maintained between time points within subjects. Adjacent structures (sinus sagittalis and parasagittal dura CSF spaces) were defined within the same slice. Individual ROIs of lateral ventricles, fourth ventricle, superior cisterna, and sinus rectus were generated using FSL-based thresholding and masking tools, and the same masks were applied to both pre- and post-intervention images for each subject.

9. The pre scan standardization procedures described in the protocol, including avoidance of coffee, energy drinks, and alcohol before study visits, appear relevant to the vascular and physiological readouts and should be reported in the main Methods section.

A: Indeed, we agree with the reviewer. This information has now been reported in the main Methods section: “participants were instructed to abstain from caffeinated beverages (e.g., coffee, energy drinks) and alcohol for at least 12 hours prior to each study visit.”

10. The protocol includes additional assessments such as the PAL memory test, sleep questionnaire, and BDI 21, whereas the manuscript focuses primarily on physiological, imaging, and blood biomarker outcomes. If these measures were prespecified but are outside the scope of the present report, a brief clarification in the Methods or supplement would help avoid uncertainty regarding their role in the overall study design.

A: We have clarified this point in the manuscript. Specifically, we now state in the method that, although the study protocol included additional assessments such as the PAL memory test, sleep questionnaire, and BDI-21, the present manuscript focuses on physiological, imaging, and blood biomarker outcomes. These additional assessments were not analyzed in the current report. We further clarify that these measures were used during the study to ensure that participants met general health criteria and did not present factors that could confound the physiological measurements.

Added text:

“The study protocol included additional assessments such as the PAL memory test, sleep questionnaire, and BDI-21, these measures were not analyzed in the present report. They were used to ensure general health status and suitability of participants, whereas the current manuscript focuses on physiological, imaging, and blood biomarker outcomes.“

11. The Results and Discussion would read more clearly if the distinction between global segmented dark fluid changes and ROI specific signal changes were maintained more consistently. At several points, the interpretation moves rapidly between whole brain, CSF, dural, and venous compartments, which makes the imaging narrative harder to follow.

A: Thank you for this helpful comment. We agree that the distinction between global segmented T2DF changes and ROI-specific findings should be presented as clearly as possible.

In response, we substantially revised Discussion sections to improve the logical flow of the imaging findings. The Results section now progresses more systematically through the intervention conditions, presenting the combined NO+WBVp intervention findings first, followed by the NO-only

and WBVp-only results. The Discussion section was also largely rewritten to provide a clearer structure and interpretation of the findings across the different anatomical compartments.

In addition, we believe the study is now easier to follow because the study design has been described in greater detail in the revised CONSORT diagram (Fig. S1), modality-specific data availability has been clarified in Fig. S27, and the exact sample size (*n*) is now reported for all statistical analyses in the Results section. Together, these revisions improve transparency and make it easier for readers to distinguish between whole-brain segmented analyses and ROI-based compartment-specific findings.

12. The figure legends should report sample size more consistently at the panel level. In several figures, different panels appear to derive from different analytic subsets, but the corresponding n is not always immediately apparent for each comparison.

A: Thank you for this suggestion. We agree that clear reporting of sample sizes is important, particularly in a multimodal study where participant availability differs across analyses. However, several figure legends are already over to the journal's practical length limits, and adding the sample size for every individual statistical test would have substantially increased their complexity and reduced readability.

To address this concern, we have instead revised the Results section to report the exact sample size for all statistical analyses and comparisons. We believe this makes it considerably easier for readers to follow the origin of each result while avoiding excessive repetition in the figure legends.

In addition, the figure legends now consistently state the relevant sample size when introducing each intervention condition or analysis panel. Together with the expanded study design and modality-availability information provided in the Supplementary Figures (Fig S27), these revisions improve transparency and facilitate interpretation of the figures and their associated analyses.

13. The figures are scientifically rich, but several panels are visually dense. In particular, figures that combine whole brain maps, ROI quantification, and physiological traces would benefit from a more orderly internal progression, so that the reader is guided from global observations to regional quantification and then to physiological support.

A: Thank you for this constructive comment. We agree that some of the figures are information-rich and therefore visually dense.

We start with the most relevant Figure 1, presenting (to us) the most impactful T2DF data with blood laboratory findings. Then in Fig's 2-4 we do start up with a whole brain global FFT power spectrogram over time followed by FFT power maps and if present statistical power change maps of the interventions. Then we show wearable sensing and T2DF results in opposed ways in Fig's 3 vs. Fig 4.We now altered this such that the dcEEG and NIRS results are in similar order after the T2DF data from more general to physiological support as mentioned by the reviewer.

This concern may relate most notably to Figure 3 where we put all NO-related data. Panels 3e–l were intentionally grouped together because they present results from the NO intervention sub-study, which was performed in a separate NO-effect cohort (n = 12), and were designed to provide a coherent presentation of the associated physiological and imaging findings within the same intervention condition. For this reason, we preferred to keep these results together rather than distribute them across multiple figures.

To further improve readability, we have revised the manuscript structure more broadly. The study design has been clarified substantially, including a revised CONSORT diagram (Fig. S1), a more detailed description of modality-specific data availability (Fig. S27), and explicit reporting of sample sizes throughout the Results section. In addition, the Results now follow a more systematic progression through the intervention conditions, and the Discussion has been reorganized to better separate the different findings and anatomical compartments.

We believe that these revisions provide a clearer framework for interpreting the whole-brain maps, ROI-based analyses, and physiological measurements while maintaining the logical grouping of related results.

14. In Figure 1, the representative anatomical insets and ROI illustrations are useful but relatively small given the interpretive importance of the parasagittal dural and venous regions. Enlarging these panels, if feasible, would improve anatomical clarity.

A: Thank you for this suggestion. Unfortunately, enlarging the anatomical inset panels within Figure 1 is only feasible to a very limited extent due to space limitations and figure layout constraints.

Thus, to improve anatomical clarity and accuracy, we have added an enlarged supplementary figure (S25) that provides a more detailed visualization of all defined ROIs, including the parasagittal dural and venous regions. We believe this additional figure facilitates interpretation while preserving the overall readability of the main figure.

15. Figure 5 is useful as a summary figure, but the legend should distinguish more explicitly between experimentally observed findings and interpretive schematic elements. This would allow the figure to function more effectively as a synthesis panel without conflating measured data with proposed mechanism.

A: We thank the reviewer for this important comment and agree that schematic summary figures should be clearly distinguished from experimentally observed findings. In response, we have revised Figure 5 and its presentation in several ways.

First, we added introductory text immediately before the figure to explicitly state that the schematic components are intended to illustrate potential mechanisms and physiological interpretations arising from the experimental observations, rather than to represent directly measured pathways.

Secondly, we revised the figure itself to improve the distinction between each modality and their measured findings and conceptual elements.

Thirdly, we expanded the figure legend substantially and now indicate which experimental findings support each major component of the schematic. Specifically, the proposed fluid redistribution, vascular responses, and protein-movement pathways shown in the illustration are now linked to the corresponding imaging, physiological, and blood biomarker findings presented in Figures 1–4 and the Supplementary Data.

Our intention was not to present the schematic as mechanistic proof, but rather as a visual synthesis of the multimodal observations obtained in this study. We agree with the reviewer that the field benefits from careful separation of data-driven observations and mechanistic interpretation, and we believe the revised text, figure, and legend now make this distinction much clearer.

16. The color scale conventions used for the pre, post, and difference maps should be clarified more explicitly in the figure legends. Where visual comparison across panels is central to interpretation, it should be immediately clear whether identical intensity ranges and scaling conventions were applied.

A: Thank you for this suggestion. We have revised the figure legends to clarify the color scale conventions used throughout the manuscript. Specifically, we now explicitly state when identical intensity ranges and scaling conventions were applied to the pre- and post-intervention maps, allowing direct visual comparison across panels. Statistical maps are separately identified as voxel-wise significance maps, thereby distinguishing them from the intensity-based images. We believe these revisions improve the transparency and interpretability of the figures.

17. Several figure legends are highly compressed relative to the complexity of the panels they describe. In the more data dense figures, particularly Figures 1, 3, and 4, a slightly more structured legend format would improve readability and reduce ambiguity regarding which statistical comparisons correspond to which panels.

A: WE do agree on this but given the instructions on text clarity improvement vs. limitations of word counts we were able to add only limited amounts of text to improve readability. We did however add text into the main text for further clarification of the work.

Reviewer #3

In the manuscript "Vasomechanic treatment increases efflux of human brain proteins" the authors Tuunanen et al study the combined delivery of nitric oxide (NO) and whole body vibrations (WBVp) in healthy adults (n = 29) on brain fluid dynamics and 27 CNS-to-blood protein efflux. The authors conclude that this intervention is a promising method for enhancing clearance of amyloid from the brain. Brain clearance in humans is still ill-understood and any study showing significant changes is therefore of importance. Unfortunately, this study has a sub-optimal design and the readout techniques can be

challenged. I would certainly more information to be able to fully appreciate their experiments.

My main concerns are:

1. The design of the experiments is sub-optimal. The NO condition was later tested in another cohort, the WBVp could not be done while in the MRI, and the study lacks a proper control condition. The lack of a control condition makes pre-post analysis difficult to interpret, as repeated MRI is known to induce changes in stress within participants. Or were all participants experienced MRI volunteers?

A: Thank you for these construtive and important comments that we totally agree on.

Based on these important points, we went on to improve the study design and performed the missing control SHAM study using the same T2 dark fluid scan with identical parameters, n =13 (39.7 ± 10.9 years, range 26 – 67 years, 6 female), where the subjects were scanned and laid down for 45 min resembling the WBVp treatment time on a Siemens VIDA 3T using identical T2DF sequence and parameters.

There result in the sham data indicates that there were no statistically significant changes in the DF images before vs. after the sham control state without any interventions (below, supplementary figure S22). However, this proved to be quite interesting and relatively informative new experiments as it can be noticed from the differential image below that the DF image intensity tended to increase (shown as red color) rather decrease as in all the interventions (lighter blue). In other words it seems that simply laying down for an extra while 45 min seems to increase brain water/protein content in the brain, albeit not significantly in 13 subjects.

We thought originally that as one is already laying supine in the scanner for at least 45 minutes, there should not be much changes but to our surprise there seems to be a slight change. But thanks to the precise reviewer comment, we now can conclude that indeed the laying down tends to increase, while the interventions decrease the brain signal intensity instead.

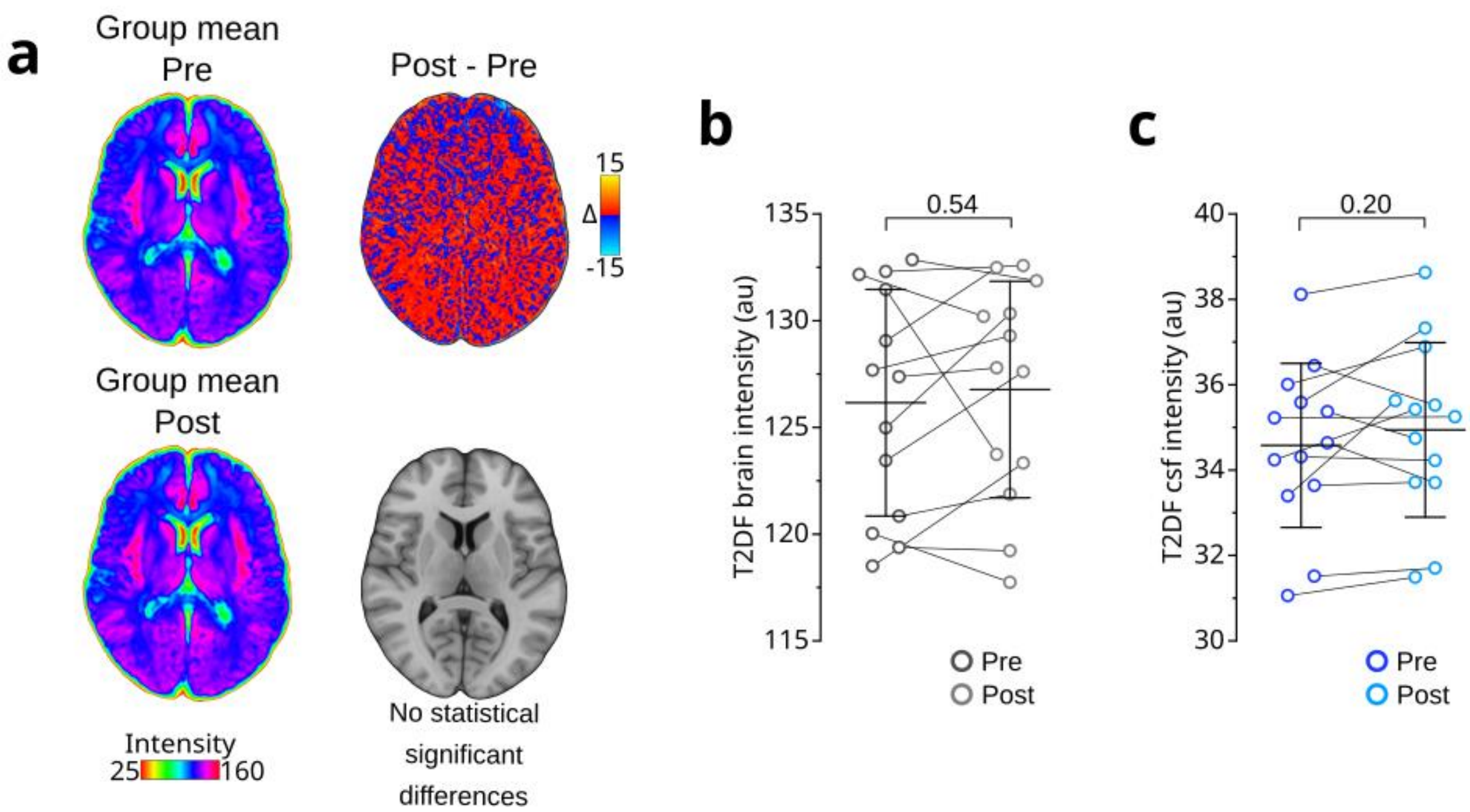

We also compared pre-intervention DF image baselines between different sessions on paired study within the same subjects, n=11, a) baseline group mean, subtraction and differences maps, b) Mean T2DF signal intensity of brain tissue, c) and CSF signal intensity. Again there was no significant differences between these baselines over the whole brain, and the subtraction map 2nd – 1st study showed a slight overall tendency towards reduced (blue) DF intensity in brain following any kind of intervention within the same subjects in contrast to the sham control state of increased signal tendency after just laying down (figure below).

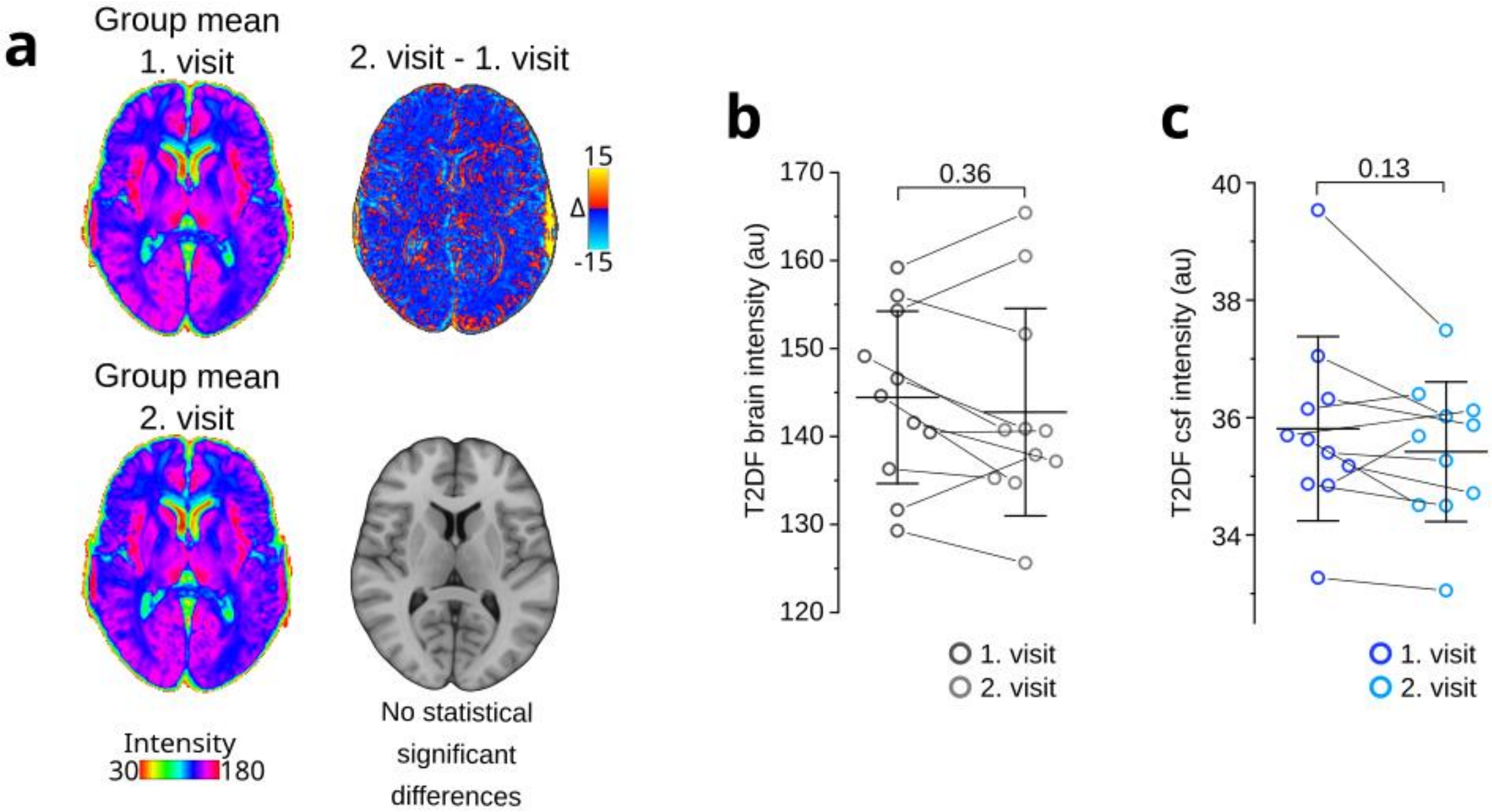

In addition, we have clarified the study design throughout the revised manuscript. The NO-only and sham-control experiments are now explicitly described as exploratory sub-studies conducted in separate cohorts and analyzed separately from the primary randomized crossover study. To improve transparency, we have updated the CONSORT flow diagram (Fig. S1), which now clearly illustrates the randomized crossover design of the main NO+WBVp and WBVp interventions, as well as the separate NO-only and sham-control sub-studies. We believe these revisions make the study structure easier to follow and help readers distinguish between the primary and exploratory components of the investigation.

2. The use of quantitative T2 dark fluid employed as by the authors, would need further validation. Signal intensities in MRI are easily influenced by e.g. preparation phases (power optimalization, frequency determination) as well as by post-processing (bias-field correction). Moreover, in the CSF the intensities are near zero, where Rician noise issues come into play. Without more convincing validation, I cannot be certain that the observed changes can indeed be interpreted as a changes in molecular content of CSF. This is a major point and would need more experiments.

A: We thank the reviewer for this important and insightful comment. Also the Rician noise issue was relatively new to us despite nearly 30 years of fMRI experience, so this also tought us new in depth knowledge on the sensitive aspects of the T2DF signal especially in the low intensity fluid areas.

We agree that quantitative interpretation of T2 dark-fluid signal intensity requires caution and that several technical factors, including image acquisition, preprocessing procedures, and noise characteristics, may influence the measured signal. We have therefore substantially expanded the Methods, Results and Discussion sections to address the technical and biological validity of the T2DF findings more thoroughly.

All MRI acquisition parameters, including field-of-view, were kept identical across all pre- and post-intervention scans and across all subjects using the Albayram et al. protocol to minimize confouds. No acquisition parameters were modified between conditions. To minimize processing-related effects, the same preprocessing pipeline was applied systematically to all datasets.

To further address the Reviewer's concern of potential influence of bias-field correction, we performed an additional analysis using non-bias-corrected images. Bias-field correction is important to remove low-frequency intensity inhomogeneities caused by radiofrequency coil sensitivity variations. Such correction is widely regarded as an essential preprocessing step because intensity non-uniformities can impair brain and csf segmentation, tissue classification, and quantitative image analysis. The correction procedure was applied identically and systematically to all datasets using the same processing pipeline.

Reassuringly, the overall findings remained highly similar. The figure below demonstrates the effects of bias-field correction on the resulting statistical maps (T2 dark-fluid imaging, n = 24, Pre > Post NO+WBVp intervention, main figure 1f). Blue voxels represent statistically significant effects ($p < 0.05$) in the bias-field-corrected data, whereas red voxels represent statistically significant effects in the non-bias-field-corrected data. These results indicate that the observed whole brain level findings are not driven by the bias-field correction procedure itself as there is a small result deficit in the non-biasfied corrected data area in the left sensorimotor cortex only, as shown below.

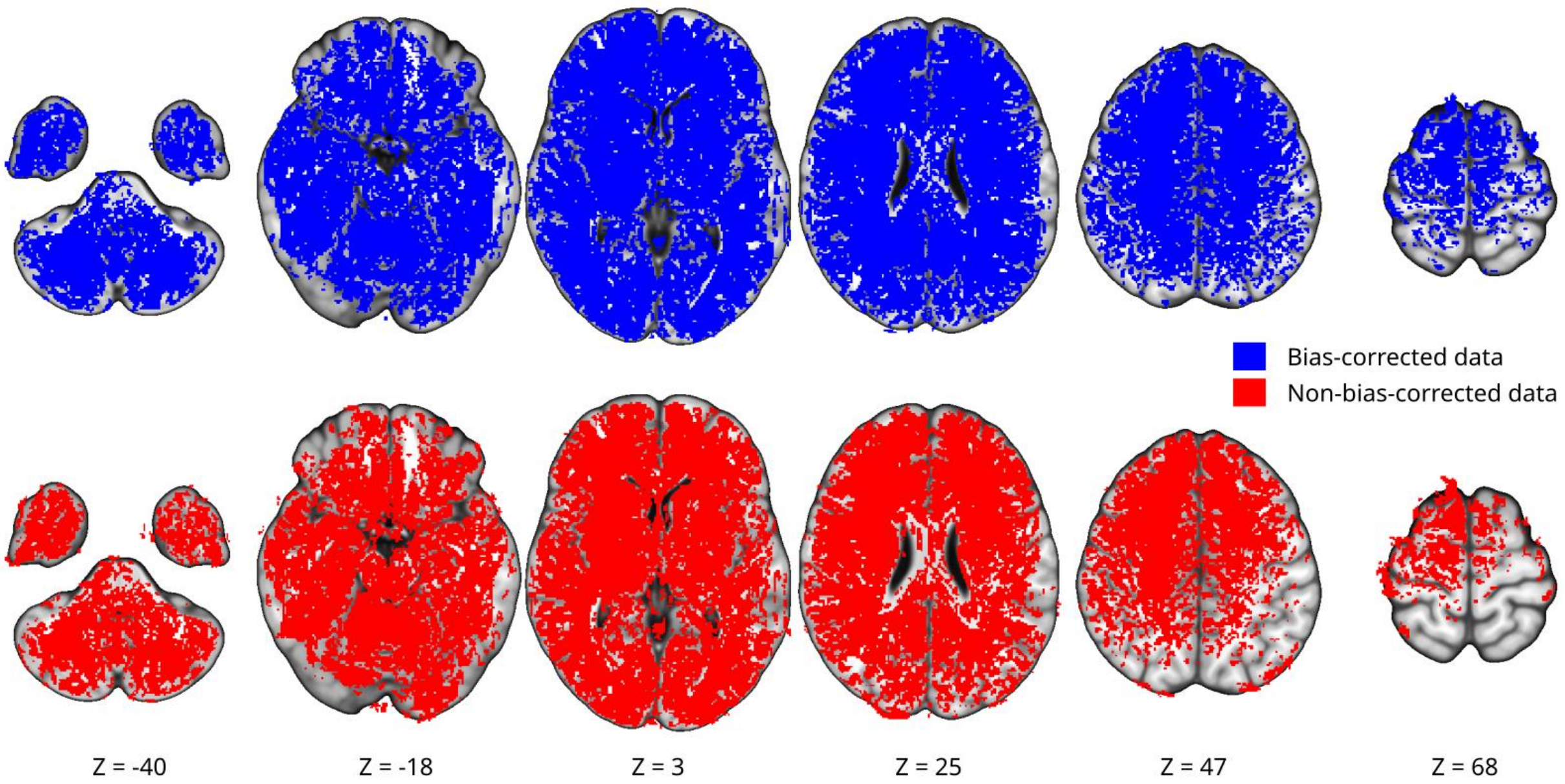

Importantly, omission of bias-field correction resulted in visibly reduced sensitivity in brain regions affected by strong magnetic susceptibility-related intensity inhomogeneities, particularly near peripheral brain boundaries where such artifacts are most prominent. These are precisely the areas that bias-field correction is designed to improve. Therefore, while the overall findings remained highly consistent between corrected and non-corrected analyses, bias-field correction improved anatomical uniformity and reduced the risk of regional underestimation of true effects.

Furthermore, all manually defined ROI analyses (posterior and middle sagittal sinus, parasagittal dura spaces, para-cavernous dura, lymph nodes, and related CSF compartments) were performed on the original T2DF image data using a diagnostic DICOM viewer and anatomical guidance from T1- and T2-weighted images. Thus, these ROI results were not dependent on bias-field correction. We have expanded the ROI methodology accordingly in the revised Methods section.

The reviewer also raises the important issue of Rician noise, particularly in low-intensity CSF regions. To reduce voxel-level instability, ROIs were deliberately defined as large as anatomically feasible while avoiding partial-volume tissue effects, and identical anatomical regions were sampled between pre- and post-intervention scans.

Importantly, if the findings were primarily explained by Rician noise, similar changes would be expected throughout low-intensity CSF compartments, especially the ones close to ROI's with changes between pre vs. post-intervention scans. However, this was not observed. Several neighboring low-intensity regions, including the lateral ventricles, fourth ventricle, superior cerebellar cisterna, middle parasagittal CSF and sagittal sinus, showed no significant signal changes (c.f. illustrative Fig below, Fig. S6 in the manuscript). In contrast, the observed effects were highly compartment-specific, with changes localized to selected posterior venous sinuses regions. We therefore believe that a generalized noise-related explanation is unlikely.

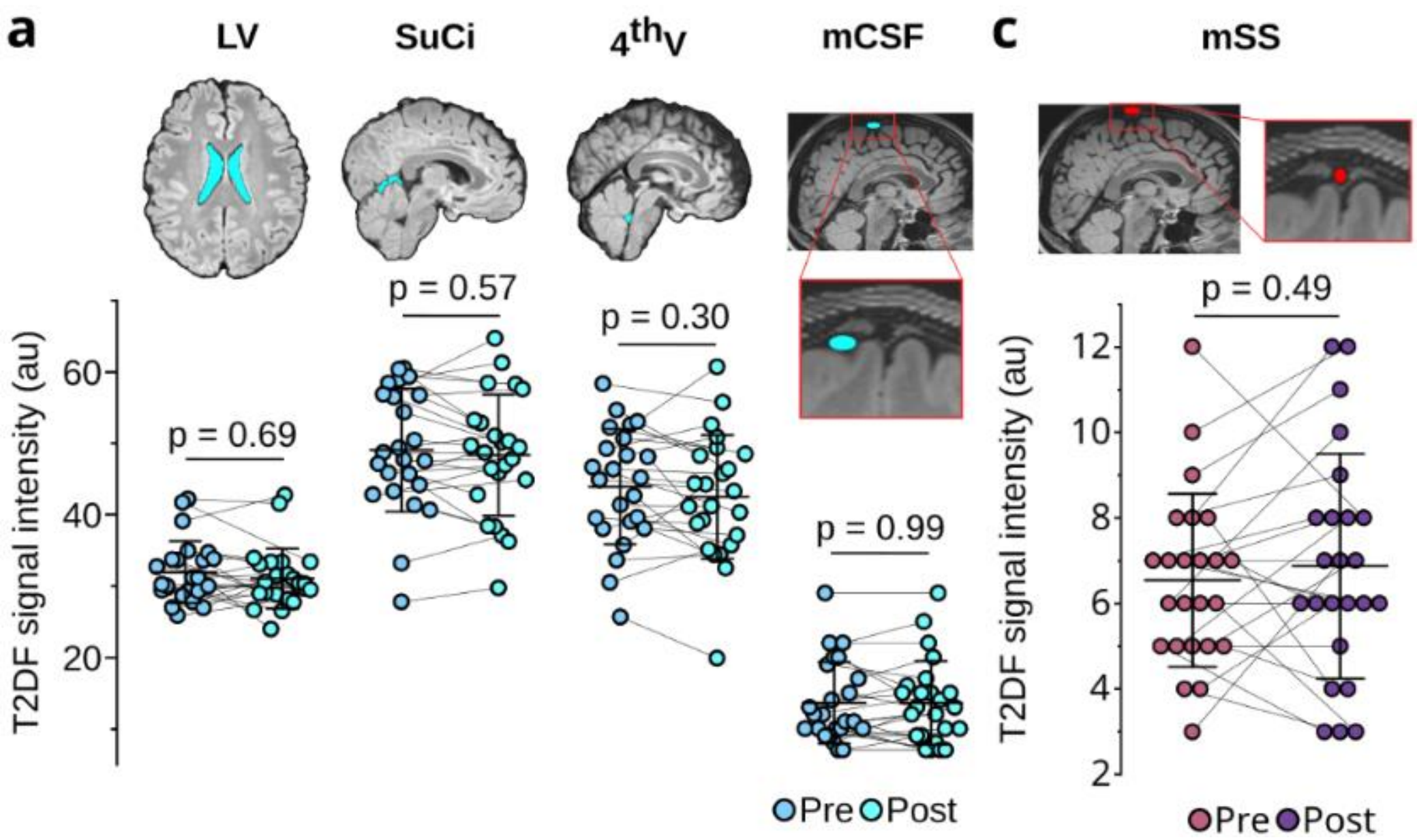

Encouraged and motivated by the several reviewers' comments, we additionally performed a pooled analysis combining baseline, (pre-), and post-intervention measurements and compared T2DF signal intensity with independently quantified blood biomarkers. We found that posterior sagittal sinus T2DF intensity correlated positively with plasma Aβ40 and Aβ42 concentrations, while whole-brain T2DF intensity correlated inversely with both circulating plasma and serum GFAP levels. These relationships provide independent biological evidence indicating that the measured T2DF signal changes in both tissue and blood are linked to physiological brain protein biomarker changes rather than imaging noise alone. The corresponding results have now been added to the Results section (c.f. Fig below, Fig. 1m, Fig. S8 in the manuscript) and discussed in greater detail.

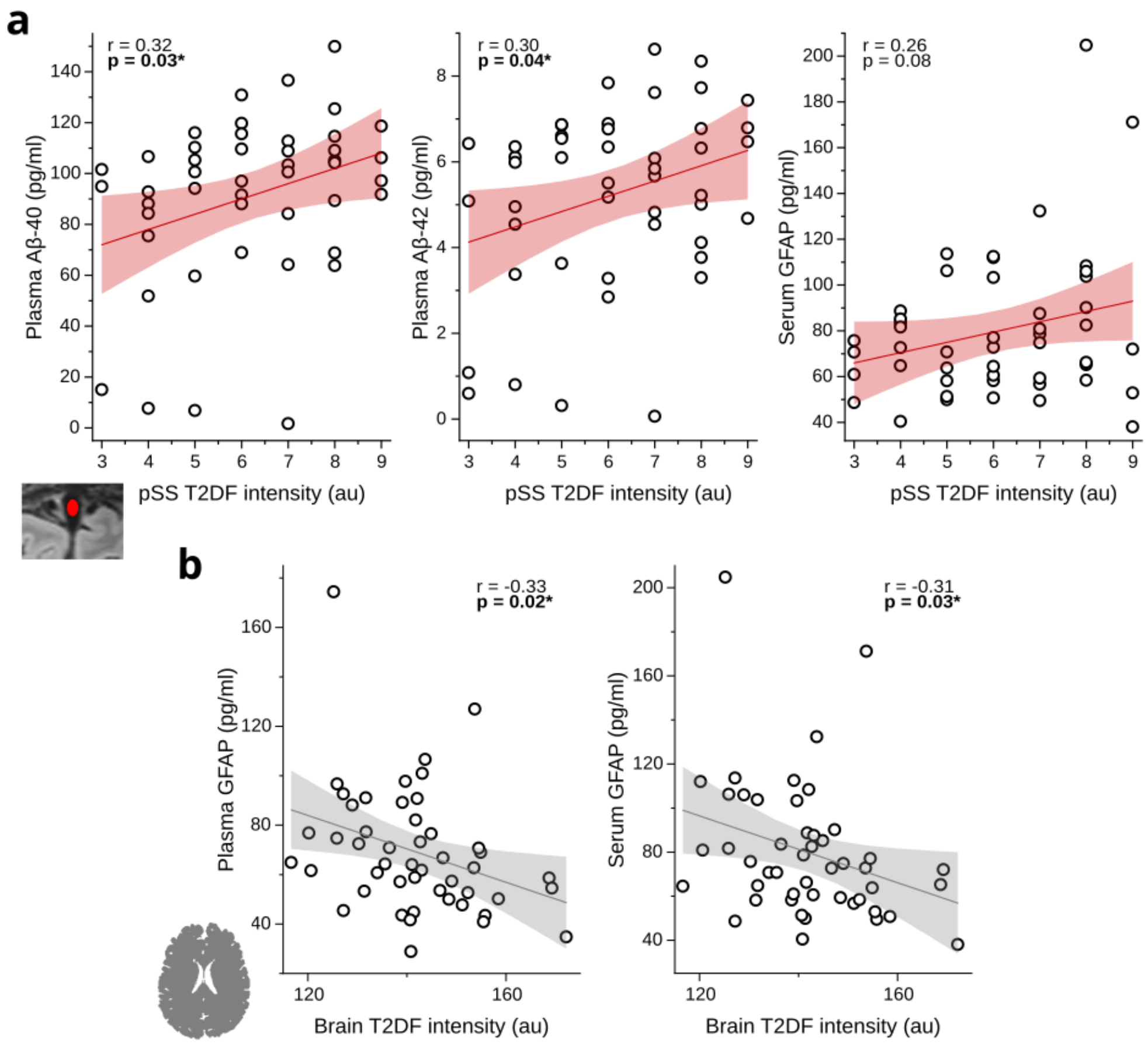

To further clarify interpretation of the T2DF signal, we substantially expanded the Discussion and Methods sections. We now describe the physical basis of the sequence in greater detail and explicitly discuss how T2DF signal reflects the ratio between water and macromolecules in different brain compartments, while acknowledging that changes in water content and macromolecular concentration cannot be completely separated by T2DF imaging alone.

Added text:

"We used the dark-fluid (FLAIR-based) MRI signal intensity analysis implicated earlier by other groups for investigating brain solute transport within glymphatic PSD interfaces[22,23,58]. T2DF is a very important sequence in clinical neuroimaging, where tissue-bound water has high signal intensity due to proximity of water ^{1}H proximity to macromolecules like proteins. Less constricted water molecules distant from macromolecules in neurofluidic compartments such as blood and CSF are labelled dark in T2DF MRI by a precisely timed inversion pulse rendering freely precessing water ^{1}H signal dark.

In clinical imaging an acutely increased T2DF signal is often related to arising from brain tissue edema and darkening of T2DF signal typically reflects a reversal of such pathology after a successful therapeutic interventions. The current T2DF MRI results need to the interpreted differentially based on the brain compartment at hand: while in tissue the darkening may reflect mostly reduced amount of bound water ^{1}H signal, while an increase in normally low signal neurofluidic compartments T2DF suggests increased amount of water molecules close to macromolecules such as proteins.

As shown by a previous protein phantom model verification study with a similar 3T scanner using identical TR, TI and TE sequence parameters, the measured T2DF darkening of reflects reduced protein/water ratio and vice versa with increasing signal in free water solutions[23]. One of the most

prominent findings of the vasomechanic intervention was a whole-brain darkening of T2DF MRI signal extending into deep structures (Fig. 1f, 3f). Here, darkening of whole human T2DF image may in theory reflect either reduced amount of tissue bound water resulting in less ^{1}H protons to give MR signal, reduced amount of soluble proteins affecting the watermolecules less, or actually both. In light of the quantified hemodilution, an increase in soluble protein level increase in the blood laboratory results, in addition to the convergent fNIRS hydrodynamic brain sensing results, the most likely change in the brain is a combined redistribution of soluble proteins along with removed brain water, i.e. solutes, from the CSF compartment (Fig. 1f-h, 3h) into blood constituents (Fig 3l).

As normally the NO-induced hemodilution causes a drop in blood protein concentration (Dupuis et al. 1990), the opposing increase we detected in low molecular weight soluble brain derived proteins (< 68 kDa) in blood (Fig. 1d) strongly suggests an increased brain-derived solute efflux directly into the blood. This may well be accompanied with transient BBB related permeability effect, as also seen in the mouse model (Fig. S28b). The dcEEG level that reflects BBB permeability further suggests that BBB permeability could be increased (Kiviniemi et al., 2017 Plos One). It is also of particular interest that two posterior venous sinus T2DF signal intensities showed a linear correlation with quantified circulating blood protein concentrations (Fig. 1m), linking the imaging findings more directly to blood biomarkers, suggesting a more posterior compartmentalized efflux mechanism in humans.

The low intensity DF compartments of posterior venous sinuses and adjacent PSD regions showed increased DF intensity following the vasomechanic intervention, suggesting the idea of enriched local protein concentration increasing the DF signal, as indicated by Albayram et al. in the PSD spaces(2022) (Fig. 1k-l) (Albayram et al. 2022). In contrast, the more frontally in the medial sagittal sinus and parasagittal/para-cavernous dural CSF regions displayed reduced, i.e. an even darker T2DF signal, consistent with simultaneous water dilution suggestive of either increased protein removal or increased dilution of CSF water (Fig. 1k, 4k).

To the best of our knowledge, this detection of differential solute efflux based on cranial position in humans is a novel finding and thus currently we do not have direct knowledge on the structural mechanical underpinnings of such differences in humans. There could be anatomical and cerebrofluidic pressure-dependent differences in the BBB and PSD permeability favouring dorsal area efflux. Mice have been shown to have differential basal meningeal lymphatic vessel structure and valves function that allows increased tracer efflux compared to dorsal meningeal vessels (Ahn et. al. 2019). In the supine position there is also more hydrostatic pressure towards the posterior areas, which, together with the increased ICP during arterial vasodilation (Fig's 1 & 3) could in compartmentalized subarachnoid space CSF favor posterior solute drainage towards the posterior venous sinuses in human cranium (Eide et al. Nat Com 2020; 2024, Møllgård et al., 2023).

Furthermore, the middle vertex PSD CSF-filled space showed quite a persistent DF signal alteration, with a mean interval between measurements of 9.4 months (range 0.5–22.3 months, Fig. S23), although majority of the T2DF signal intensity changes were transient over most parts of the brain. This seems to be in line with the notion that these vertical areas may have slowest solute in/outflow processes as already suggested by Eide and co-workers in humans(Eide&Ringstad 2020). Here we show that once there is a change in the PSD solute content, the changes in these areas also seem to persist for months. This slow nature of solute exchange could be partially related to hydrostatic effects in the low pressure environment of venous drainage. Even in recumbent positions hydrostatic pressure of the vertical sagittal sinus is relatively elevated compared to lower venous sinuses, which in theory should direct the pressure driven solute flow towards be posterior areas as shown predominantly to occur in Fig's 1 & 3.

In theory, the T2DF changes could be due to technical Rician noise in low-intensity T2DF signal areas due to altered positioning between scans or T2 flow differences between scans. However, such noise artifacts should be present uniformly in a given area of the T2DF image. Here, however, the T2DF changes were highly compartment specific, and not present uniformly in the brain as should be in the Rician noise case. Neighboring supracellar cistern as well as other central CSF low intensity CSF areas show no changes, while adjacent sinus rectus dark fluid signal shows increases that are linearly correlating with the venous blood both Aβ's, which argues against the Rician noise artefact possibility (Fig, 1h&l, 3h, 4i, S7, S15, S17, S19, S21).

The increased CSF flow speed can also increase T2 DF signal intensity in narrowed CSF areas. However, most global brain changes indicate darkening of T2DF signal. The T2FD signal intensity increases in posterior venous sinuses and PSD areas after vasomechanic intervation do not overlap with any MREG pulsation power increases. We have previously shown that an increase in brain water flow speeds should coincide with simultaneous power increase in the brain tissue and CSF (Elabasy et al., 2025, Järvelä 2025, Väyrynen 2026). During the NO vasodilation the MREG power of especially vasomotor and respiratory pulsations was increased in wide areas; however in these areas the T2DF signal should increase instead of the detected darkening if the signal changes were due to increased flow. After the combined intervention, the pulsation power changes were mostly gone, and thus the T2DF signal increase posterior sinuses and other areas as well sound to be more related to increased blood protein content rather than flow speed increase, c.f Fig 1 m.

Taken together, while we thankfully acknowledge the reviewer(s) comments and write that T2DF MRI results as a relative measure of protein/water concentrations cannot directly quantify protein concentration and thus interpretation must remain cautious, the convergence of anatomically localized effects, robustness to bias-field correction, ROI analyses performed on original image data, and independent biomarker correlations substantially increases our confidence that the observed findings reflect genuine physiological changes rather than technical artifacts alone."

3. In the MREG spectrograms, it is quite clear that cardiac and respiratory frequencies are quite drastically changed during the interventions. It is unclear whether their post-processing accurately corrects for this. The text is too vague (ranges and peaks were obtained from individual FFT spectrums; frequencies were double checked with the physiological recordings of the scanner), please explain exactly how this was done. Also, unclear why the scanner recordings were not used as an independent way to determine the frequency ranges. Now, the authors are double dipping: first use the spectrum to determine the frequency range and then integrate the same spectrum over that ranger. It is also unclear whether the person doing the post-processing was blinded towards the condition and whether pre- and post were scored immediately after each other. More information is needed and a second observer would be nice to prove objectivity of the measurements.

A: We thank the Reviewer for this important comment. We agree that the original description of frequency determination was too brief and potentially unclear. We have now expanded the methodological description in both the main manuscript and the Supplementary Information (Supplementary Text 1). In addition, we have included a new supplementary figure (below, S26) illustrating corresponding physiological recordings and MREG FFT spectra, together with the frequency-selection procedure.

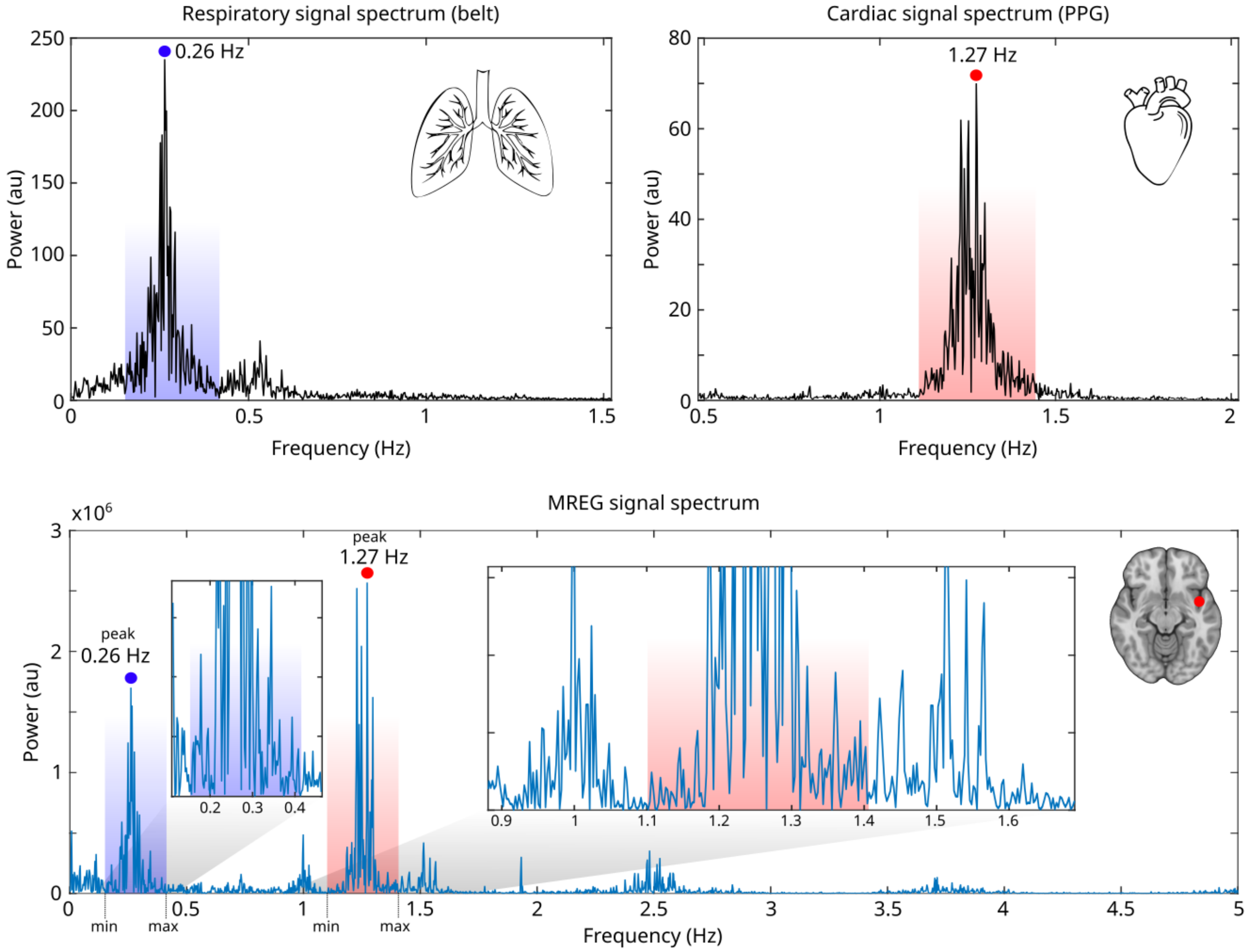

We have previously demonstrated in several publications that cardiorespiratory frequencies can be precisely identified from MREG quite and they correspond to simultaneously recorded physiological measurements in six separate articles[26,27,29–32].

REDACTED

Järvelä et al. Nat Med Com 2022 (Suppl Fig.1, doi:10.1038/s43856-022-00187-4)[27]*. Pulsation range estimation verification between MREG and physiological measurements. Individual minimum, maximum, and peak values of respiration (blue signal) and cardiac (red signal) pulse derived from end-tidal* CO_2 *and photoplethysmogram, respectively, correlate extremely well with corresponding values estimated from MREG data.*

Additionally, in particular our sleep study showed a correlation coefficient of approximately 0.9 between cardiac pulsations derived from MREG and simultaneously acquired fingertip photoplethysmography (PPG) during wakefulness. Interestingly, this correlation decreased substantially during sleep, likely reflecting differential physiological modulation of central and peripheral signals due increased brain vasomotor waves in sleep(below).

REDACTED

Tuunanen et al. Front NSci 2024 (Fig. 3, doi: 10.3389/fnins.2024.1457732)[30].

Accordingly, because the present analyses were performed directly on the MREG signal, and because the high temporal resolution of MREG enables highly accurate identification of physiological frequencies, the peak frequencies and ranges were determined from the MREG FFT spectra. The best way to separate these pulsations in high frequency MREG data is to utilize FFT bandfiltering since the TR is << 500 ms, as indicated by[33] Chen, Glover and Polimeni. Time domain methods such as RETROICOR cannot accurately filter these signals from the data, since these pulsations move within the brain and thus have a phase difference in each image voxel. Thus only FFT bandpass can be used based on individual cardiorespiratory frequencies, which always react to the current interventions.

As for the proper cardiorespiratory filtering, these pulsations are often regarded as noise and they are attempted to be removed often by time domain tools such as RETROICOR by Gary Glover[34] (Glover et al., MRM 2000). In his important study he writes correctly (and now very helpfully) by stating his original **assumptions** for time domain regression:

“The correction method assumes that the time series of intensities *y*(*t*) in a pixel is corrupted by additive noise resulting from cardiac and respiratory functions. The cardiac and respiratory states are monitored during the scan using a photoplethysmography and a pneumatic belt placed around the subject's abdomen, respectively. It is assumed that the physiological processes are quasi-periodic so that cardiac and respiratory phases can be uniquely assigned **for each image** in the time series…. the cardiac phase is **assumed to advance linearly** from 0 to 2π during each *R*–*R* interval and is reset to 0 for the next cycle….

…Accordingly, the physiological noise component $y_\delta(t)$ can be expressed as a low-order Fourier series expanded in terms of these phases:

$$y_\delta(t) = \sum_{m=1}^{M} a_m^c \cos(m\varphi_c) + b_m^c \sin(m\varphi_c) + a_m^r \cos(m\varphi_r) + b_m^r \sin(m\varphi_r) \quad (1)$$

where the superscript on coefficients *a* and *b* refers to cardiac or respiratory function, and $\phi_c(t)$ and $\phi_r(t)$ are the phases in the respective cardiac and respiratory cycles at time *t*. In this work $M = 2$ was used after preliminary studies showed that **little was gained by including higher-order** terms. With Eq. [1] evaluated, the data are corrected by subtracting $y_\delta(t)$ from $y(t)$.

Following Hu, the cardiac phase is defined as

$$\varphi_c(t) = 2\pi(t - t_1)/(t_2 - t_1) \quad (2)$$

Where t_1 is the time of the *R*-wave peak in the cardiac cycle, just preceding *t*, and t_2 is the time for the subsequent *R*-wave peak. Thus, the cardiac phase is **assumed to advance linearly from 0 to 2π during each *R–R* interval and is reset to 0 for the next cycle.”**

Below we show by actual ultrafast fMRI TR 100 measurements that these assumptions are wrong making time domain signal regression useless or even harmful for ultrafast fMRI data.

Below we used AFNI ***3dretroicor*** in an attempt to *remove* physiological noise: The green RETROICOR “corrected” signal overlaps completely with the underlying black original full band TR 100 ms MREG BOLD data without basically any removal of the physiological signals. The regressors were derived from the brain data directly (light blue color card in the middle and respiratory regressors on right box illustrated in MATLAB image windows right to the sagittal MRI image shown in FLSEYES).

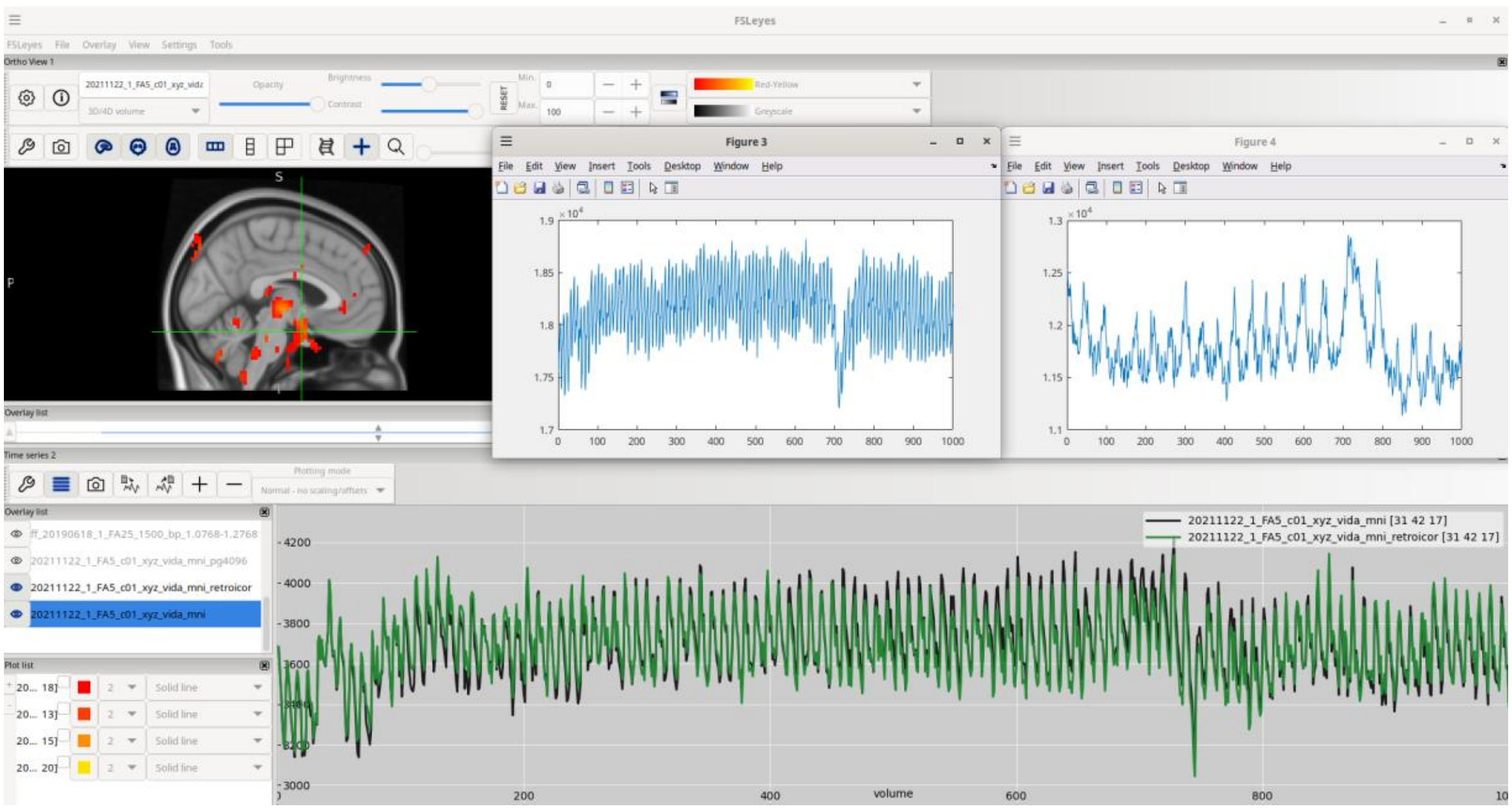

This nearly complete lack of physiological control even with RETROICOR was quite a shock to us, and this deserves to be published, and we aim to do so in the future. This was worse than we expected for ultrafast TR 100 ms data. Thus, we thank the reviewer for pointing this obvious shortcoming of the time regression.

We go on to show another subject's scan with original fMRI TR 100 ms signal having both a slow VLF vasomotor (baseline) waves and on top of it there is a strong cardiovascular pulsation: original full band signal in now in blue:

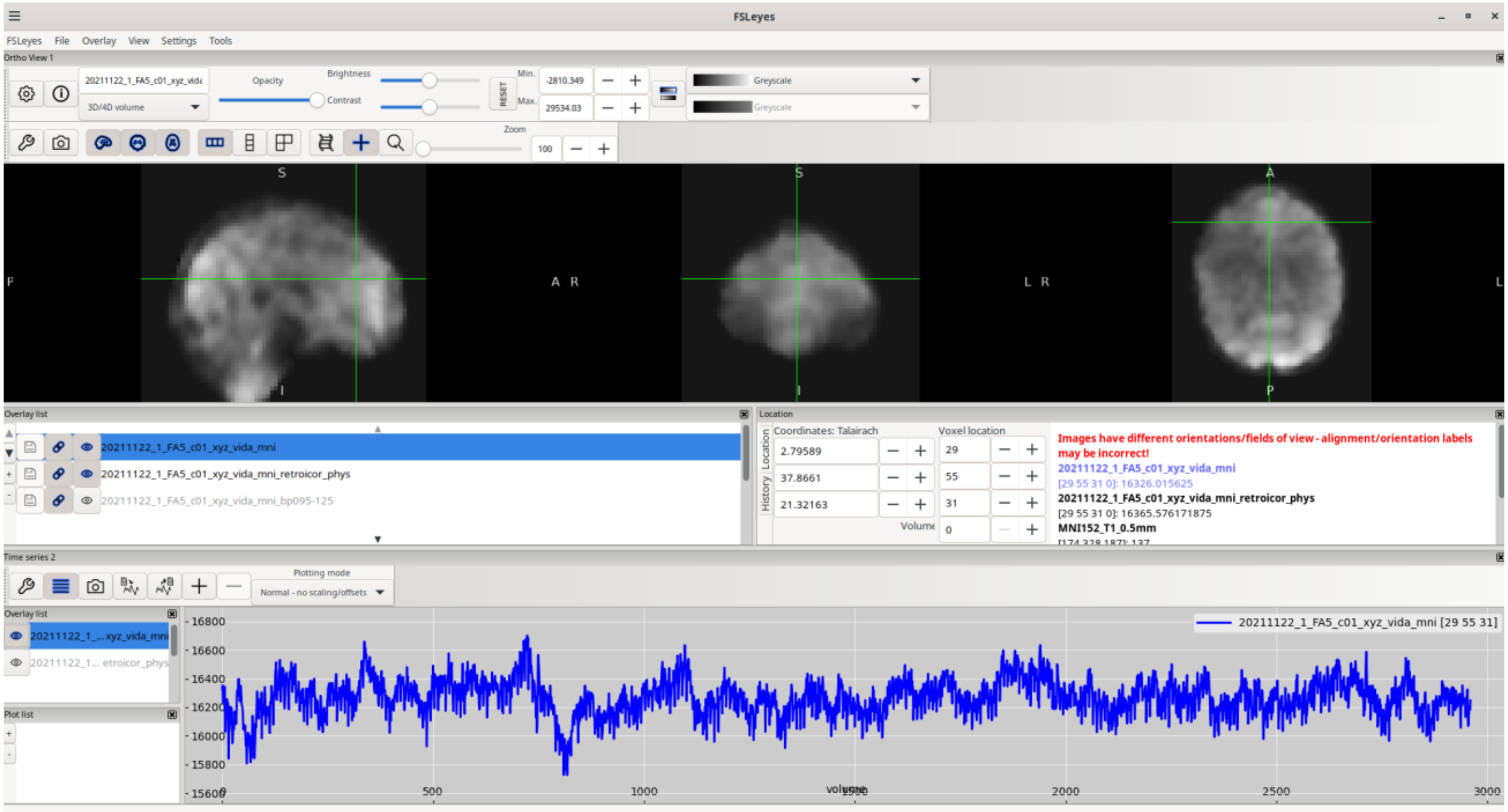

Underneath in another image the same voxel signal in black after ***3dretroicor*** using externally measured physiological SpO_2 and resp MR belt signals as regressors identical to what was proposed by Glover originally. After the "correction" the signal still has the overlaying faster cardiovascular pulsation: this black signal should be devoid of the fast cardiac pulsations completely, which it is not, as you can again see, very little was removed. And this repeats in all ultrafast data. Why?

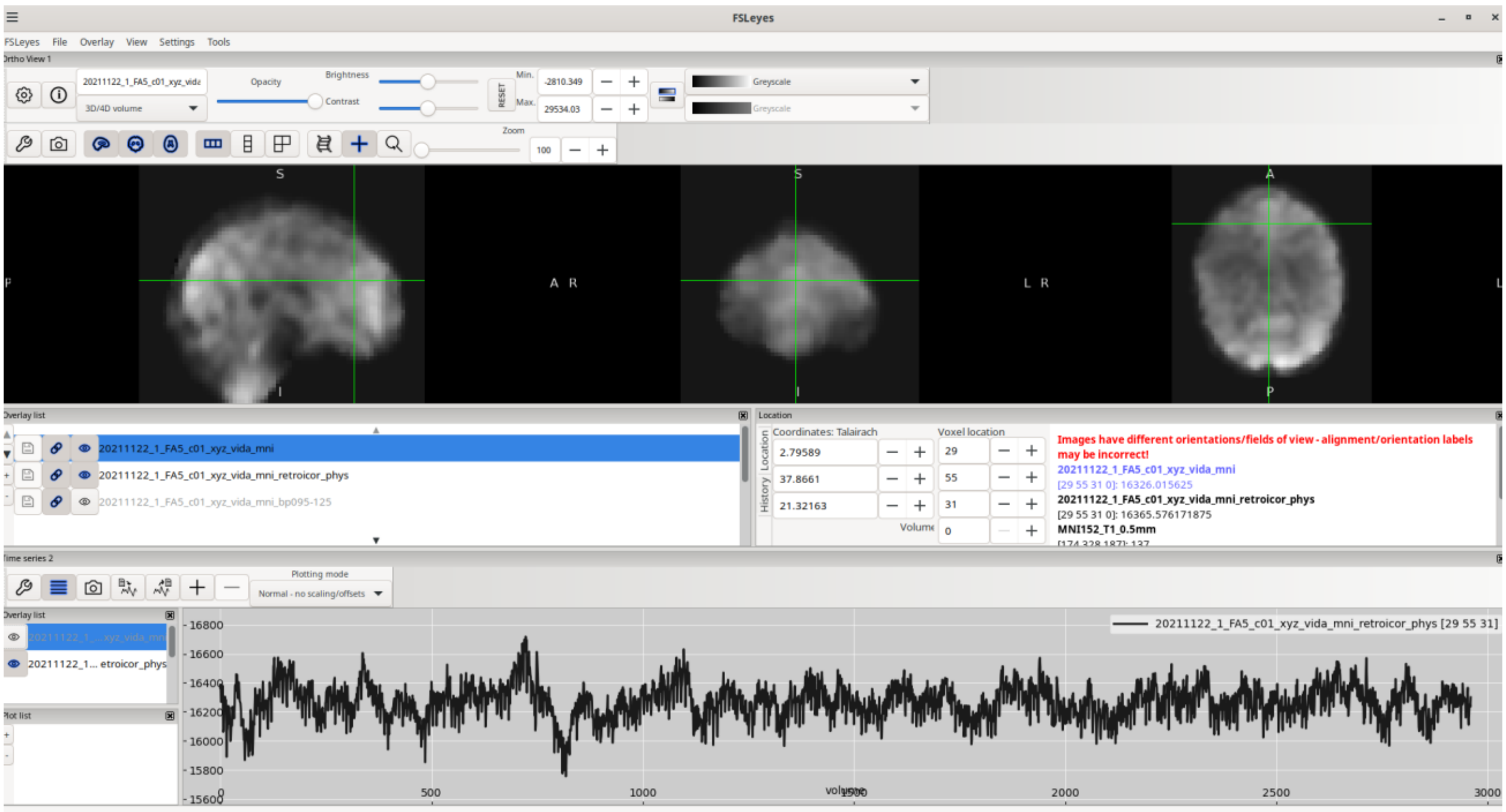

Below both original blue and black RETROICOR-corrected shown together indicating again how little RETROICOR can do to the moving physiological signals from ultrafast fMRI data.

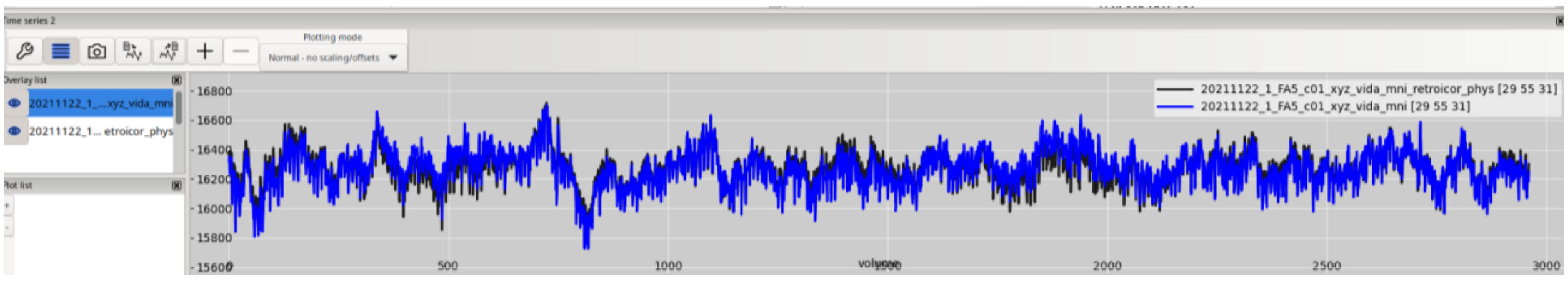

Fig 8.

Then we show bandpass effect: The same scan VLF signal after < 0.1 Hz bandpass with AFNI 3dTproject looks like this, which far superior to the RETROICOR done on the upper slide. We have used this approach since 2012 on critically sampled fMRI and fMREye data due to this obvious advantage over other methods as illustrated above.

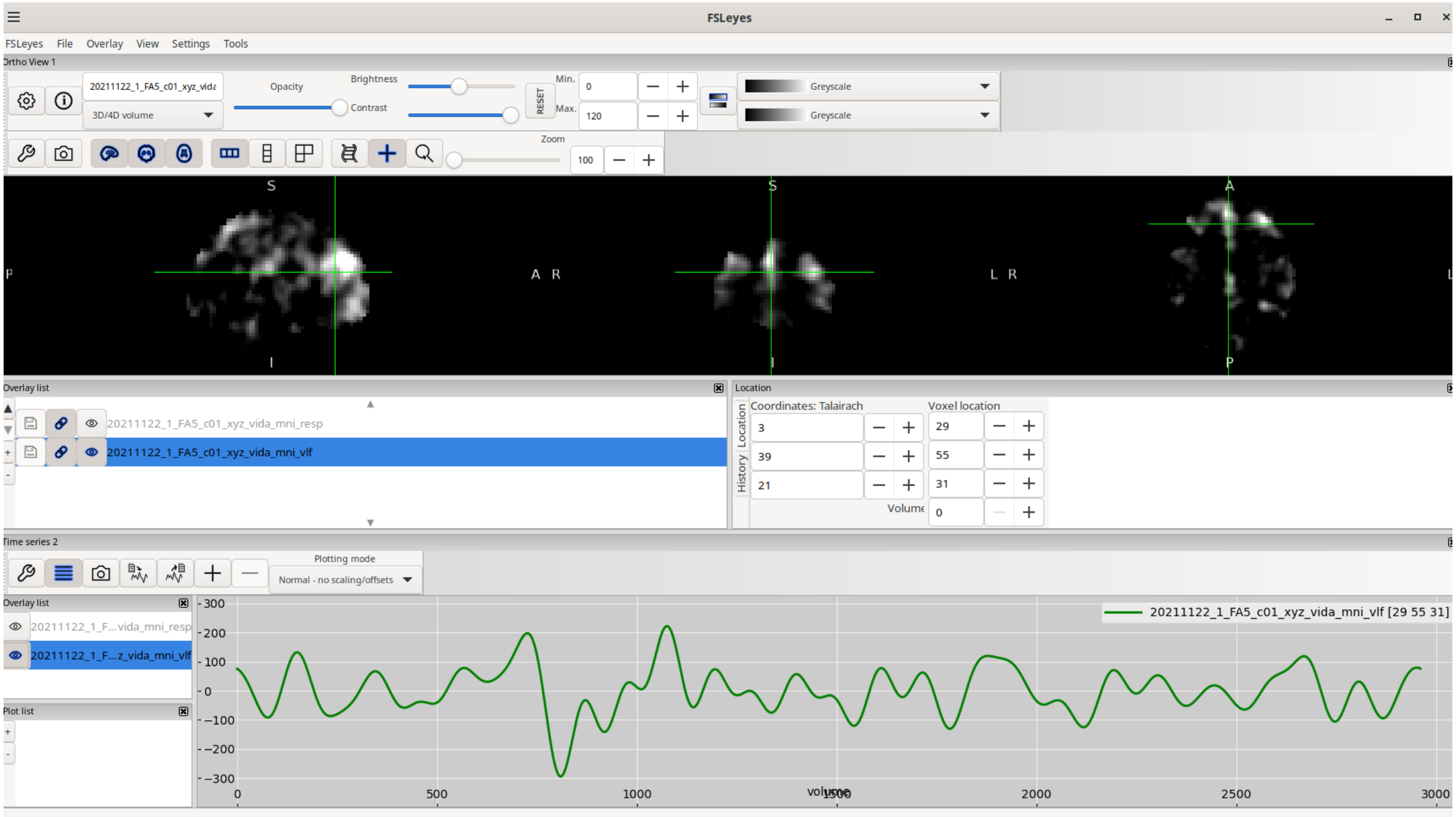

And below the same full band (FB) signal bandpassed to VLF, Resp(iratory) and Card(iovascular) pulsations on left with corresponding FFT power spectra on right. These represent isolated pulsations signals that are devoid of mixed signal effects like aliasing. And one can see that the FB FFT spectra is made of the physiological power peaks, only the y-axis scaling varies between the FFT spectra.

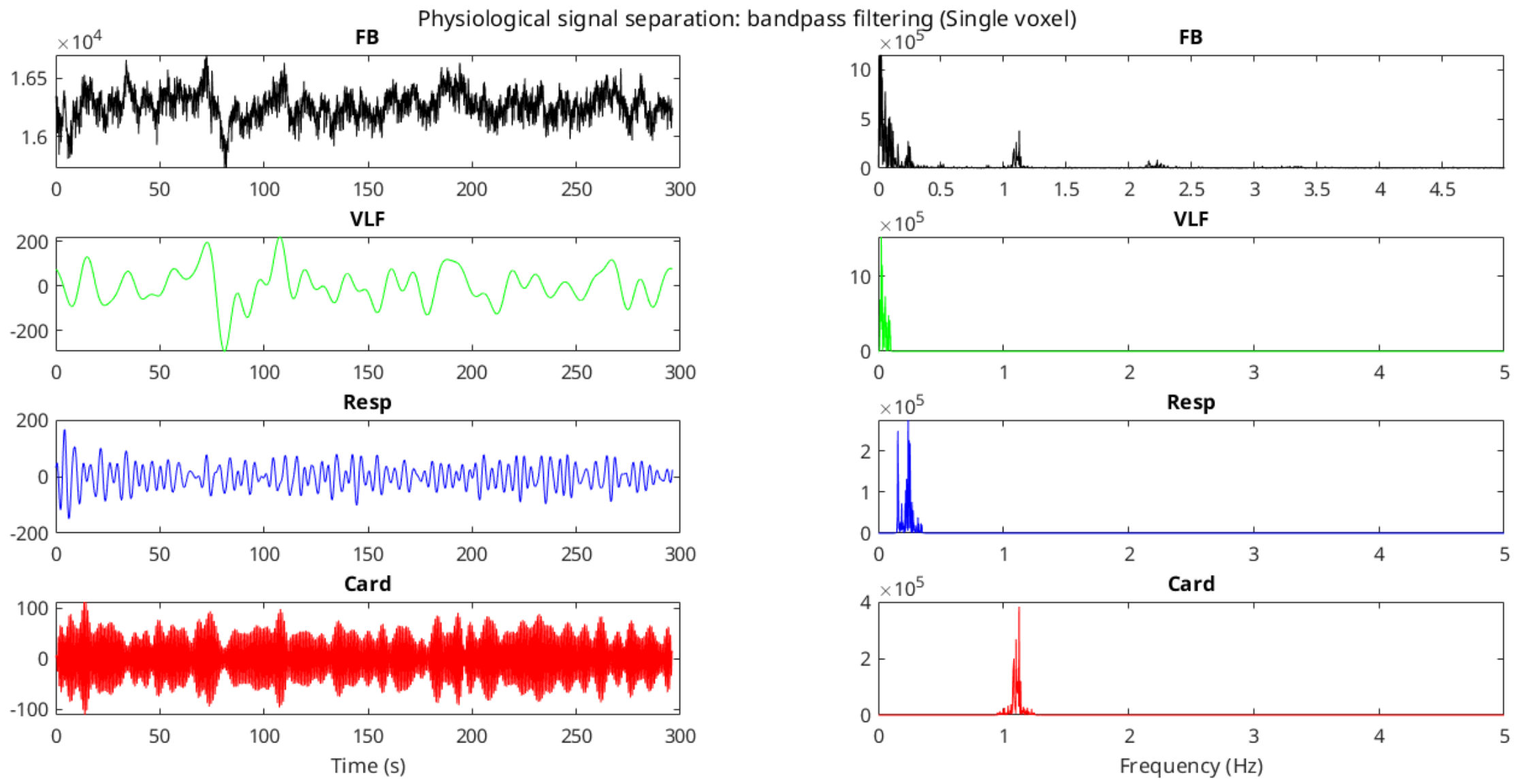

One can readily see the accuracy of the FFT band pass filtering in separating the green VLF vasomotor pulsation waves after FFT band pass filtering of 0,25 Hz respiratory and 1,1 Hz cardiac pulsation from the full band MREG data.

However, to ensure correct identification of the physiological frequencies, all frequency assignments were independently verified using simultaneously recorded PPG and respiratory belt signals just as we have done in our previous studies since 2012. This verification step was particularly important because I)interventions alter cardiorespiratory pulsations and vasomotor waves individually, and ii) respiratory modulation can generate heterodyne components in ultrafast MREG around the cardiac frequency[35]. The heterodynes on both sides of the modulated cardiac pulsation FFT peak can in MREG data sometimes exhibit greater spectral power like the true 1.14 Hz cardiac peak (c.f. the PPG vs. MREG FFT power spectra shown below) and could therefore be incorrectly identified as the cardiac frequency if physiological recordings are not performed in addition.

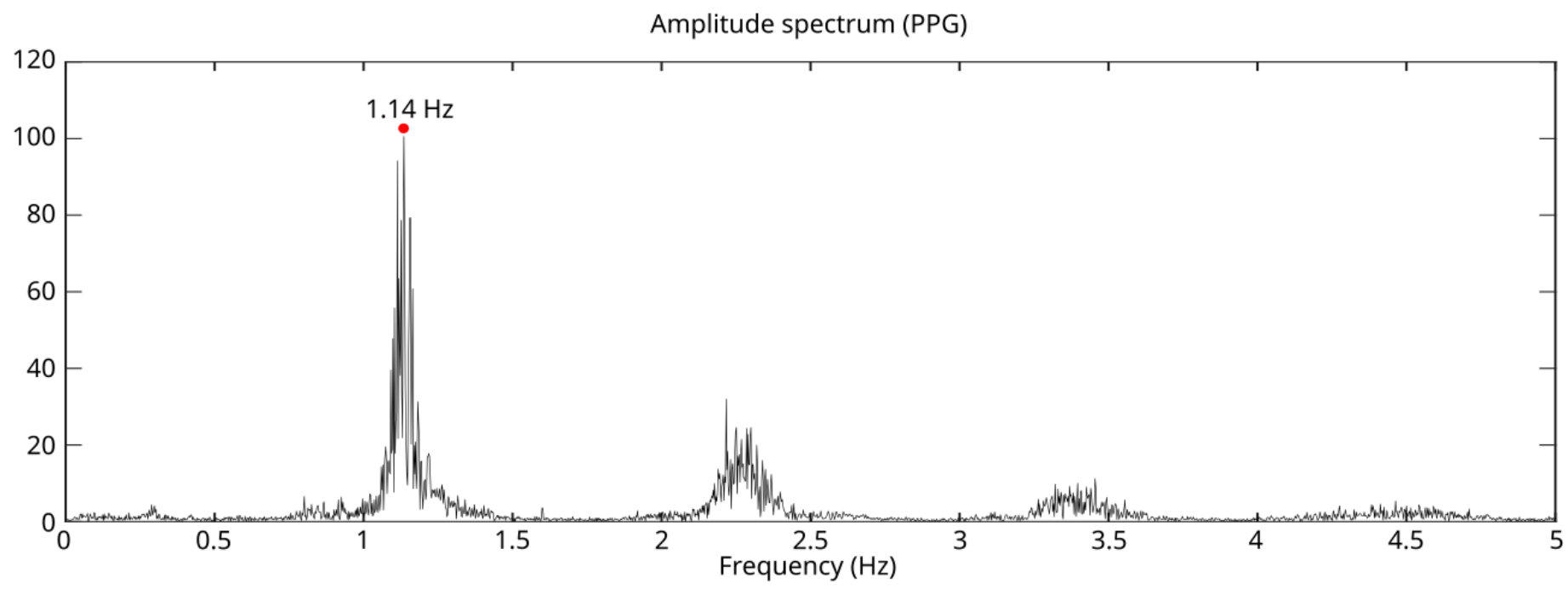

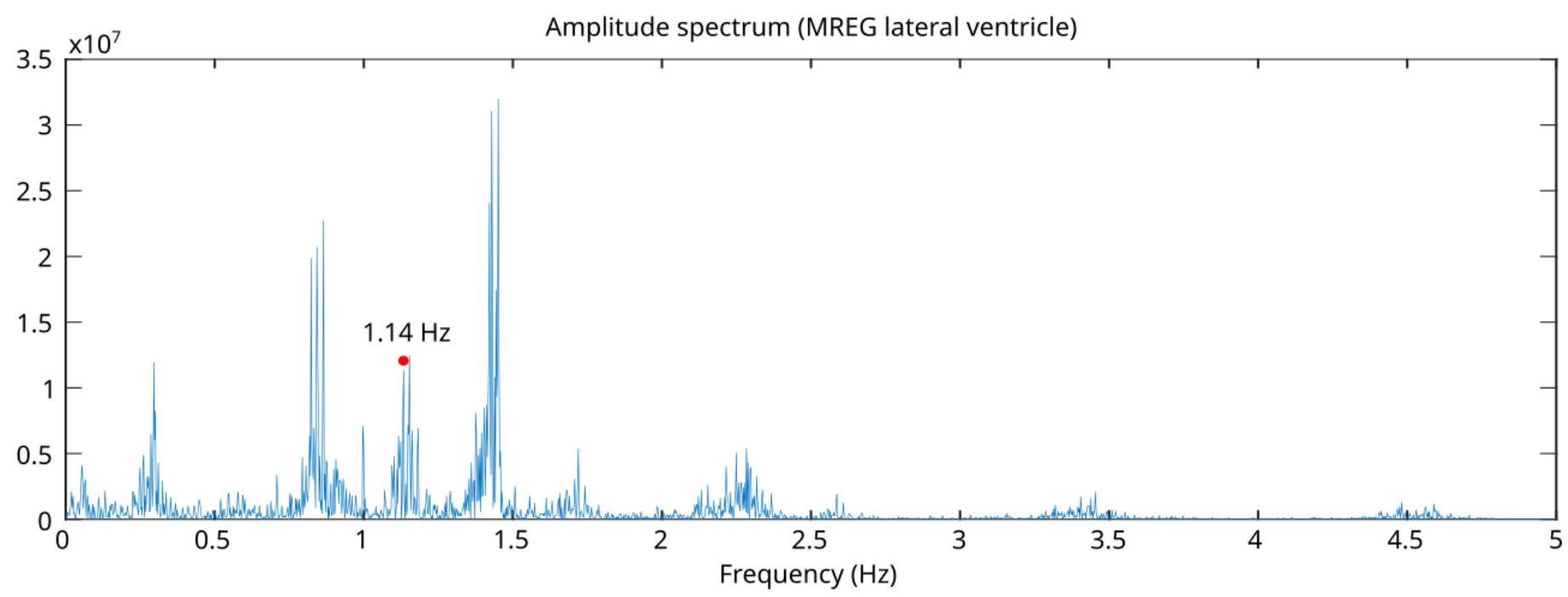

Likewise, verification against the respiratory belt signal was necessary to avoid misidentifying the prominent ~ LF 0.1 Hz fluctuation, which was substantially enhanced during the intervention, as the respiratory peak frequency.

The double dipping in fMRI literature refers to using data from a statistically significant result area again to do further secondary analysis: “In particular, 'double dipping', the use of the same dataset for selection and selective analysis” [36] by Kriegeskorte et al.

In our MREG analyses we similarly looked at the individual’s precise physiological pulsation frequencies from the brain together with physiological verification data in pre, during and post datasets. We then did the FFT power analysis comparing pre intervention baseline physiological power, as we have done in most of our recent apers, since it is the most accurate way.

To clarify the concern regarding potential "double dipping," the purpose of identifying individual peak frequencies was not to maximize intervention-related effects but to accurately account for inter-individual and intervention-related shifts in true physiological frequencies as they occur in the brain. As the NO and WBVp interventions do alter physiological cardiovascular status due to vasodulation and constriction respectively, as can be seen from the continuous independent brain sensing data in Fig’s 2-4, it would be wrong to take the baseline frequencies for instance since the interventions cause physiological changes that the autonomic nervous system reacts to by altering the cardiovascular physiology to adjust for the MAP changes for instance.

To further address the reviewer's concern, we performed an additional analysis using a fixed-width respiratory and cardiac frequency band for all subjects. As an example, we re-analyzed the Pre vs During Nitroglycerin respiratory and cardiac pulsation power data shown in Figures 3c–d. For each subject, the respiration peak was identified, and a symmetric bandwidth of ±0.13 Hz was applied around the peak, resulting in a total bandwidth of 0.26 Hz. Similarly, cardiac peak frequency was identified, and a symmetric bandwidth of ±0.075 Hz was applied around the peak, resulting in a total bandwidth of 0.15 Hz.

The overall findings were highly similar (figure below), which we believe should alleviate concerns regarding the robustness of the results. However, we do not consider the fixed-band approach to be physiologically optimal, as part of the true spectral power may fall outside the predefined frequency window, while in some subjects the fixed bandwidth may include non-physiological noise rather than relevant physiological signal. Also the heterodyne power peaks reflecting the respiratory driven modulation of the cardiovascular pulsation amplitudes can alter the wide band results also.

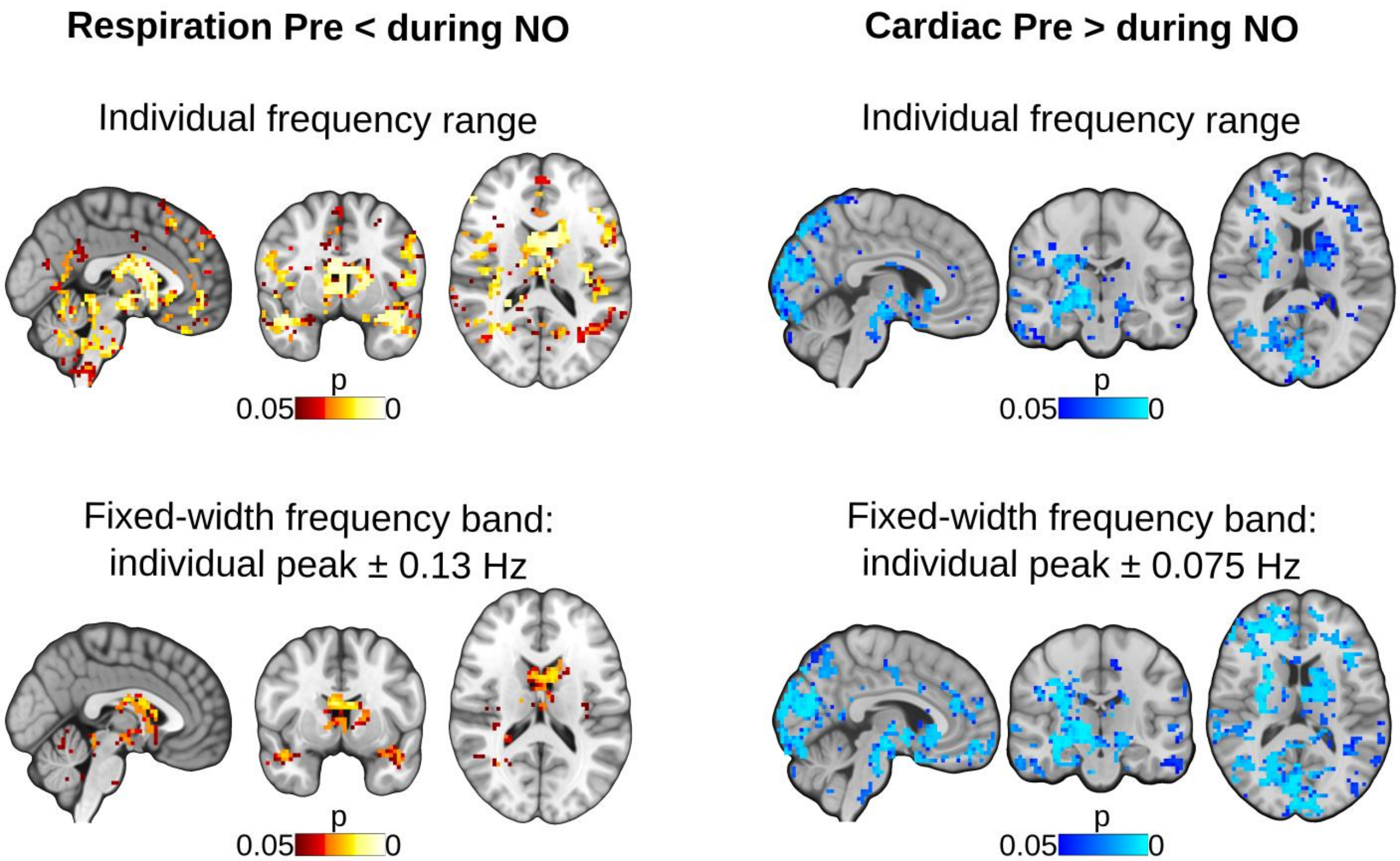

One of the major strengths of the MREG sequence is its exceptionally high temporal resolution, which enables accurate identification of individual physiological frequencies and allows these factors to be taken into account during analysis.

4. I find the cartoons of Figure 5 rather speculative and it would need more text to fully explain why certain arrows are drawn: i.e. for each process depicted in the cartoon, clearly indicate on what data/finding that is based. This field suffers from too many diagrams, with little data underlying the schemes.

A: We thank the reviewer for this important comment and agree that schematic summary figures should be clearly distinguished from experimentally observed findings. We also note that Reviewer #2 considered Figure 5 useful as a synthesis figure, while raising a similar concern regarding the distinction between experimentally observed results and interpretive elements.

In response, we have revised Figure 5 and its presentation in several ways.

First of all, we added introductory text immediately before the figure to explicitly state that the schematic components are intended to illustrate potential mechanisms and physiological interpretations arising from the experimental observations, rather than to represent directly measured pathways.

Secondly, we revised the figure itself to improve the distinction between measured findings per each modality and conceptual elements.

Thirdly, we expanded the figure legend substantially and now indicate which experimental findings support each major component of the schematic. Specifically, the proposed fluid redistribution, vascular responses, and protein-movement pathways shown in the illustration are now linked to

the corresponding imaging, physiological, and blood biomarker findings presented in Figures 1–4 and the Supplementary Data.

Our intention was not to present the schematic as mechanistic proof, but rather as a visual synthesis of the multimodal observations obtained in this study. We agree with the reviewer that the field benefits from careful separation of data-driven observations and mechanistic interpretation, and we believe the revised text, figure, and legend now make this distinction much clearer.

5. Please subtract pre- and post power maps in figure 3c and 4f and show these alongside the pre- and post maps.

A: Pre–post subtraction maps for Figures 3c and 4f have now been generated and included in the Supplementary Information (Figures S12–S14, S18).

Because the main figures already are, like reviewer #2 said "visually dense", we aimed to avoid excessive visual density in the figures while still providing the requested subtraction maps for detailed evaluation.

6. Please connect dots in all figures (pre - post) for each participant.

A: We thank the reviewer for this suggestion, this markedly improves the images and indeed helps to follow the trend and consistency of the physiological changes. All plots in both the main figures and the supplementary Information have now been updated to include participant-wise connected data points. This modification improved visualization of individual responses to the intervention and provided additional subject-level information alongside the group-level statistics.

7. In all figures, always display both positive and negative effects (e.g. Figure 2C).

A: Thank you for the suggestion. All statistically significant positive and negative effects are already presented in the figures whenever such effects were observed. Detailed lightbox visualizations are provided in the Supplementary Material. To improve clarity, we have revised the figure legends to explicitly state when no statistically significant effect was found in the opposite direction (e.g., Figure 2c shows no significant negative effect).

8. Please discuss in much more detail how CSF motion could also influence T2 dark fluid signal intensities.

A: Indeed the T2 based DF images are sensitive to phase effects due to flow; speeding up of the flow increases T2DF signal. Our MREG data did present some frontal changes in brain pulsations after the whole brain combined NO+WBVp intervention. The MREG pulsation changes were anatomically markedly smaller covering limited frontal areas not explaining the wide darkening by any means. Furthermore, the pulsation increases causes brain neurofluids flow to speed up which is the opposite the whole brain darkening.

During the NO-vasodilation effect there widespread vasomotor and respiratory increases and cardiovascular over the ROIS areas of the combined We have now added the discussion how the CSF motion in theory could influence the T2DF images as follows:

The increased CSF flow speed can also increase T2 DF signal intensity in narrowed CSF areas. However, most global brain changes indicate darkening of T2DF signal. The T2FD signal intensity increases in posterior venous sinuses and PSD areas after vasomechanic intervation do not overlap with any MREG pulsation power increases. We have previously shown that an increase in brain water flow speeds should coincide with simultaneous power increase in the brain tissue and CSF (Elabasy et al., 2025, Järvelä 2025, Väyrynen 2026). During the NO vasodilation the MREG power of especially vasomotor and respiratory pulsations was increased in wide areas; however in these areas the T2DF signal should increase instead of the detected darkening if the signal changes were due to increased flow. After the combined intervention, the pulsation power changes were mostly gone, and thus the T2DF signal increase posterior sinuses and other areas as well sound to be more related to increased blood protein content rather than flow speed increase, c.f Fig 1 m.

Review answer reference list:

1. Jukkola, J. *et al.* Blood pressure lowering enhances cerebrospinal fluid efflux to the systemic circulation primarily via the lymphatic vasculature. *Fluids Barriers CNS* **21**, 12 (2024).
2. Jukkola, J. *Investigation of Treatments to Enhance Cerebrospinal Fluid Flow in the Brain: Blood Pressure Lowering and Whole-Body Vibration*. (University of Oulu, Oulu, 2022).
3. Choi, D. *et al.* Piezo1 regulates meningeal lymphatic vessel drainage and alleviates excessive CSF accumulation. *Nat. Neurosci.* **27**, 913–926 (2024).
4. Lim, X. R. *et al.* Endothelial Piezo1 channel mediates mechano-feedback control of brain blood flow. *Nat. Commun.* **15**, 8686 (2024).
5. Chi, S. *et al.* Astrocytic Piezo1-mediated mechanotransduction determines adult neurogenesis and cognitive functions. *Neuron* **110**, 2984-2999.e8 (2022).
6. Hauglund, N. L. *et al.* Norepinephrine-mediated slow vasomotion drives glymphatic clearance during sleep. *Cell* **188**, 606-622.e17 (2025).
7. Väyrynen, T. *et al.* Sleep alters neurovascular and hydrodynamic coupling in the human brain. *Proc. Natl. Acad. Sci.* **123**, e2510731123 (2026).
8. Elabasy, A. *et al.* Sleep increases propagation speed of physiological brain pulsations. 2025.01.09.632145 Preprint at https://doi.org/10.1101/2025.01.09.632145 (2025).
9. Väyrynen, T. *et al.* Sleep-Induced Vasomotor Pulsation is a Driver of Cerebrospinal Fluid and Blood-Brain Barrier Dynamics in the Human Brain. 2024.10.16.618430 Preprint at https://doi.org/10.1101/2024.10.16.618430 (2024).

10. Hussain, R. *et al.* Potentiating glymphatic drainage minimizes post-traumatic cerebral oedema. *Nature* **623**, 992–1000 (2023).
11. Fujii, K., Heistad, D. D. & Faraci, F. M. Vasomotion of basilar arteries in vivo. *Am. J. Physiol.* **258**, 1829 (1990).
12. Kiviniemi, V. *et al.* Slow vasomotor fluctuation in fMRI of anesthetized child brain. *Magn. Reson. Med.* **44**, 373–378 (2000).
13. Helakari, H. *et al.* Human NREM Sleep Promotes Brain-Wide Vasomotor and Respiratory Pulsations. *J. Neurosci.* **42**, 2503–2515 (2022).
14. Helakari, H. *et al.* Effect of sleep deprivation and NREM sleep stage on physiological brain pulsations. *Front. Neurosci.* **17**, 1275184 (2023).
15. Helakari, H. Human brain pulsations during sleep. https://oulurepo.oulu.fi/handle/10024/50134 (2024).
16. AREND, S. M., BAX, J. J., HERMANS, J., VAN DER WALL, E. E. & SEDENEY, M. I. The short-term effect of intravenous nitroglycerin on haematocrit; an addifional benefit in patients with myocardial ischaemia? *Eur. Heart J.* **15**, 114–119 (1994).
17. Albayram, M. S. *et al.* Non-invasive MR imaging of human brain lymphatic networks with connections to cervical lymph nodes. *Nat. Commun.* **13**, 203 (2022).
18. Ahn, J. H. *et al.* Meningeal lymphatic vessels at the skull base drain cerebrospinal fluid. *Nature* **572**, 62–66 (2019).
19. Ringstad, G. & Eide, P. K. Cerebrospinal fluid tracer efflux to parasagittal dura in humans. *Nat. Commun.* **11**, 1–9 (2020).
20. Eide, P. K. & Ringstad, G. Functional analysis of the human perivascular subarachnoid space. *Nat. Commun.* **15**, 2001 (2024).
21. Møllgård, K. *et al.* A mesothelium divides the subarachnoid space into functional compartments. *Science* **379**, 84–88 (2023).
22. Lewis, A. H., Cui, A. F., McDonald, M. F. & Grandl, J. Transduction of Repetitive Mechanical Stimuli by Piezo1 and Piezo2 Ion Channels. *Cell Rep.* **19**, 2572–2585 (2017).

23. Rzepliński, R., Proulx, S. T., Tarka, S., Stępień, T. & Ciszek, B. CSF outflow from the human spinal canal: preliminary results from an anatomical specimen-based model. *Fluids Barriers CNS* **22**, 32 (2025).

24. Deng, S. *et al.* Astrocyte cell volume dynamics across cortical states and transitions. *Neuron* S0896627326002205 (2026) doi:10.1016/j.neuron.2026.03.029.

25. Thijssen, E. H. *et al.* Differential diagnostic performance of a panel of plasma biomarkers for different types of dementia. *Alzheimers Dement. Diagn. Assess. Dis. Monit.* **14**, e12285 (2022).

26. Järvelä, M. *et al.* Orexin effect on physiological pulsations of the human brain. *Proc. Natl. Acad. Sci.* **122**, e2501578122 (2025).

27. Järvelä, M. *et al.* Increased very low frequency pulsations and decreased cardiorespiratory pulsations suggest altered brain clearance in narcolepsy. *Commun. Med.* **2**, 1–13 (2022).

28. Dupuis, J., Lalonde, G., Lemieux, R. & Rouleau, J. L. Tolerance to intravenous nitroglycerin in patients with congestive heart failure: Role of increased intravascular volume, neurohumoral activation and lack of prevention with N-acetylcysteine. *J. Am. Coll. Cardiol.* **16**, 923–931 (1990).

29. Tuovinen, T. *et al.* Cardiorespiratory related brain signal variability is increased in Alzheimer's disease. *Sci. Rep.*

30. Tuunanen, J. *et al.* Cardiovascular and vasomotor pulsations in the brain and periphery during awake and NREM sleep in a multimodal fMRI study. *Front. Neurosci.* **18**, 1457732 (2024).

31. Kananen, J. *et al.* Increased interictal synchronicity of respiratory related brain pulsations in epilepsy. *J. Cereb. Blood Flow Metab.* 0271678X221099703 (2022) doi:10.1177/0271678X221099703.

32. Haverinen, J. *et al.* Heightened respiratory brain pulsations indicate fluid dynamic dysfunction in early multiple sclerosis. *J. Cereb. Blood Flow Metab.* 0271678X261453802 (2026) doi:10.1177/0271678X261453802.

33. Chen, J. E., Polimeni, J. R., Bollmann, S. & Glover, G. H. On the analysis of rapidly sampled fMRI data. *NeuroImage* **188**, 807–820 (2019).

34. Glover, G. H., Li, T.-Q. & Ress, D. Image-based method for retrospective correction of physiological motion effects in fMRI: RETROICOR. *Magn. Reson. Med.* **44**, 162–167 (2000).

35. Raitamaa, L. *et al.* Spectral analysis of physiological brain pulsations affecting the BOLD signal. *Hum. Brain Mapp.* (2021).

36. Kriegeskorte, N., Simmons, W. K., Bellgowan, P. S. & Baker, C. I. Circular analysis in systems neuroscience – the dangers of double dipping. *Nat. Neurosci.* **12**, 535–540 (2009).

Reviewer #1:

The authors have addressed all my questions. Mechanical stimulation provides a new and helpful way for brain modulation. I recommend acceptance of this paper.

Answer: We thank the reviewer for the careful evaluation of our manuscript and for the positive comments. We greatly appreciate the reviewer's constructive feedback throughout the review process, which has helped us improve the manuscript. We are pleased that the revised version has satisfactorily addressed all concerns.

Reviewer #2:

Thank you for the comprehensive and thoughtful revision. I have carefully reviewed the response letter and the revised manuscript. The authors have addressed the major conceptual, methodological, and reporting concerns in a substantive manner, and the manuscript has improved considerably.

The interpretation is now more appropriately aligned with the evidence. The removal of the synergy claim, the clearer distinction between the randomized crossover study and the exploratory cohorts, the definition of the primary endpoint family, and the improved reporting of sample sizes have strengthened the analytical framework. The expanded descriptions of intervention timing, hemodilution correction, serum and plasma findings, ROI delineation, adverse events, and the complementary role of the murine studies have also improved transparency and reproducibility. The additional analyses linking posterior venous sinus T2DF signals with circulating biomarkers provide valuable convergent support for the overall interpretation.

I am satisfied that my principal concerns have been addressed. Before publication, I recommend only two limited clarifications. First, the title and final conclusion remain somewhat more definitive than the careful interpretation presented throughout the revised manuscript. Slightly more cautious wording would improve consistency with the observational and exploratory nature of the human findings. Second, the statement that only statistically significant biomarker correlations are illustrated should be revised or accompanied by confirmation that all tested correlations are available in the Source Data or Supplementary Information.

These are minor points that do not require additional experiments. Overall, the authors have responded constructively and have produced a substantially stronger manuscript.

Answer: We thank the reviewer for the positive evaluation of the revised manuscript and for these constructive final suggestions. In response, we have adopted more cautious wording in both the title and the final conclusion to better reflect the observational and exploratory nature of the human findings.

The title has been revised to ”Vasomechanic treatment is associated with increased efflux of human brain proteins”

The final conclusion has been revised to ”We conclude that the multimodal findings are consistent with increased human brain solute efflux following combined NO-mediated vasodilation and Piezo1-targeted mechanotransductive stimulation, which was safely implemented under controlled

experimental conditions.” In addition, to address the comment regarding biomarker correlations, we revised the Methods section and added the following statement: “All tested correlations, including non-significant results, are provided in the Supplementary Information and Source Data file.” We hope these revisions fully address the reviewer's remaining concerns.

Reviewer #3:

I would like to thank the authors for this extensive resubmission: this was very informative. I have no further concerns.

Answer: We thank the reviewer for the careful evaluation of our revised manuscript and for the positive feedback. We greatly appreciate the time and effort invested in reviewing our work and the constructive comments provided throughout the review process. We are pleased that the revisions have satisfactorily addressed the reviewer's concerns.
